# Stereoselective Synthesis of Tropanes via a 6π‐Electrocyclic Ring‐Opening/ Huisgen [3+2]‐Cycloaddition Cascade of Monocyclopropanated Heterocycles

**DOI:** 10.1002/anie.202006030

**Published:** 2020-08-18

**Authors:** Carina M. Sonnleitner, Saerom Park, Robert Eckl, Thomas Ertl, Oliver Reiser

**Affiliations:** ^1^ Institut für Organische Chemie Universität Regensburg Universitätsstrasse 31 93053 Regensburg Germany

**Keywords:** bicyclo[3.2.1]octanes, cocaine analogues, furans and pyrroles, isoquinuclidines, microwave-assisted [3+2]-cycloaddition, tropanes

## Abstract

The synthesis of tropanes via a microwave‐assisted, stereoselective 6π‐electrocyclic ring‐opening/ Huisgen [3+2]‐cycloaddition cascade of cyclopropanated pyrrole and furan derivatives with electron‐deficient dipolarophiles is demonstrated. Starting from furans or pyrroles, 8‐aza‐ and 8‐oxabicyclo[3.2.1]octanes are accessible in two steps in dia‐ and enantioselective pure form, being versatile building blocks for the synthesis of pharmaceutically relevant targets, especially for new cocaine analogues bearing various substituents at the C‐6/C‐7 positions of the tropane ring system. Moreover, the 2‐azabicyclo[2.2.2]octane core (isoquinuclidines), being prominently represented in many natural and pharmaceutical products, is accessible via this approach.

## Graphical abstract

**Cyclopropanated furans** and pyrroles can be converted into tropanes and isoquinuclidines, featuring a 6π‐electrocyclic ring‐opening/ Huisgen [3+2]‐cycloaddition cascade of 2‐oxo‐ and 2‐aza‐bicyclo[2.1.0]‐hex‐3‐enes.

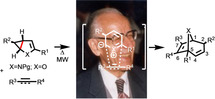

Tropane alkaloids, being characterized by an 8‐azabicyclo‐[3.2.1]octane core, serve as key motifs in drug design due to their unique biological activities.^1^ Important representatives of this class are Atropine (**1 a**), Scopolamine (**1 b**), Calystegine C2 (**1 c**) and (*R*)‐(−)‐cocaine (**2**) (Figure 1). Although these compounds have the same core structure, they differ greatly in their chemical as well as pharmacological properties. For instance, (*R*)‐(−)‐Cocaine (**2**) is a reuptake inhibitor of the three monoamine transporters serotonin, dopamine and noradrenalin,^2^ whereas atropine (**1 a**) and scopolamine (**1 b**) are competitive muscarinic receptor antagonists.^3^ Alterations of these transporter functions may play a role in diseases like Parkinson and Alzheimer.3c, 4

**Figure 1:**
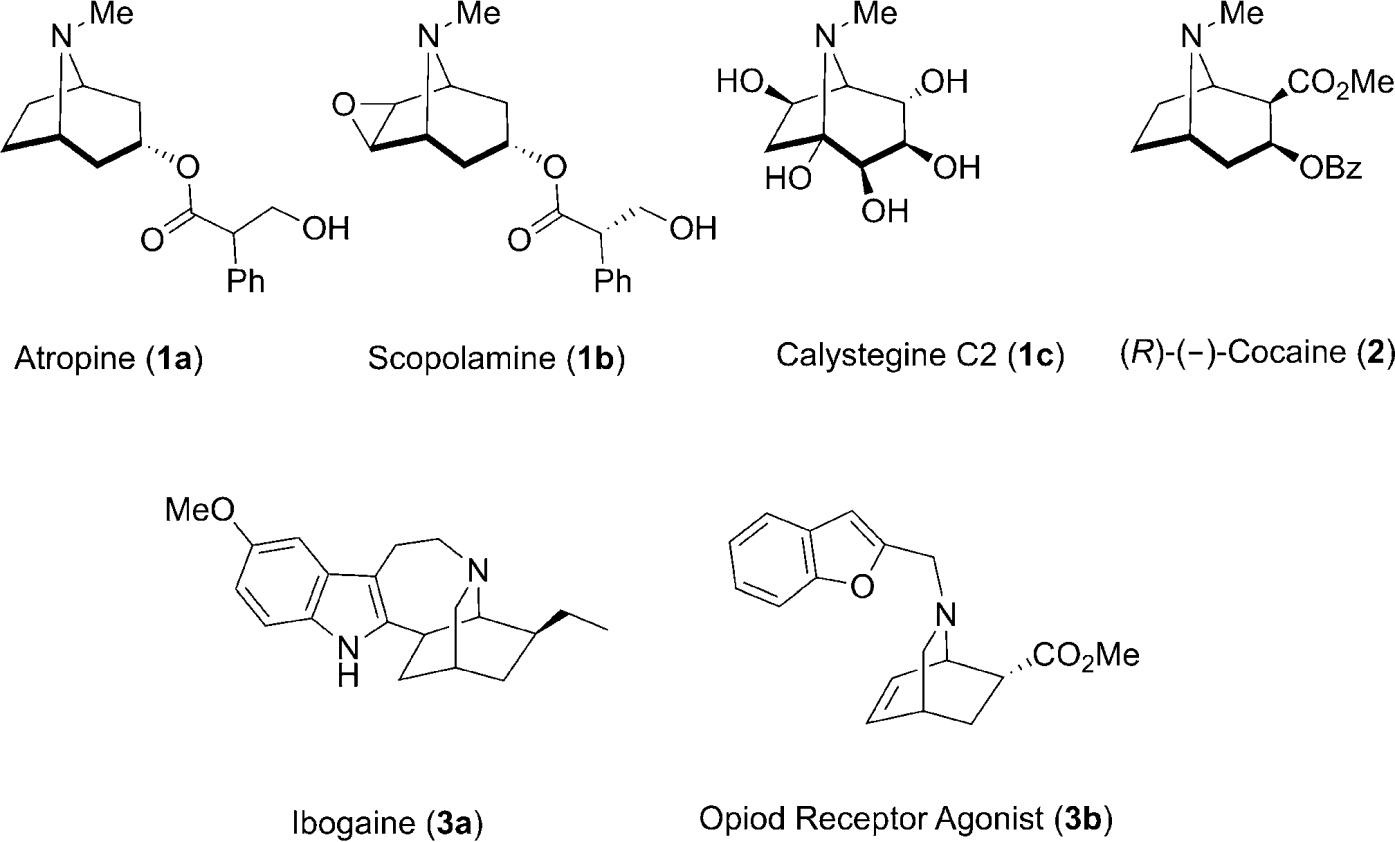
Important representatives of biological active tropane and isoquinuclidine alkaloids.

Calystegines such as **1 c** belong to the class of glycoalkaloids and impact rhizosphere ecology as nutritional sources for soil microorganisms and as glycosidase inhibitors.^5^ Furthermore, 8‐oxabicyclo[3.2.1]octanes have attracted great attention in the design of medications for cocaine abuse due to their promising dopamine transporter inhibitor properties.^6^ Likewise, the 2‐azabicyclo[2.2.2]octane core (isoquinuclidines) is prominently represented in many natural and pharmaceutical products: a representative example is ibogaine (**3 a**), being an important lead structure in the development of analgesia with anti‐addictive properties.^7^ The key step for the efficient synthesis of tropane alkaloids and analogues is the construction of bridged seven‐membered rings containing the appropriate functionalities, and a number of elegant solutions have been presented to this challenging problem.2e, 8 In the 1970s Fowler et al. reported that homo‐pyrrole can undergo cycloaddition reactions under thermal activation with suitable dipolarophiles to form bicyclic seven‐membered ring systems.^9^ Thereafter, Herges and Ugi^10^ described an analogous cycloaddition reaction of homo‐furan with activated dipolarophiles, followed by elegant mechanistic studies by Klärner^11^ and Yu.^12^ Nevertheless, given the limited substrate scope and low or unreported product yields, this type of reaction is still an underexplored area in organic synthesis.^13^ Herein, we report the microwave‐assisted 6π‐electrocyclic ring‐opening/ [3+2]‐cycloaddition cascade of monocyclopropanated pyrroles **4** and furans **5** towards the synthesis of 8‐azabicyclo[3.2.1]octanes **7** and 8‐oxabicyclo[3.2.1]octanes **8** (Scheme 1a).

**Scheme 1:**
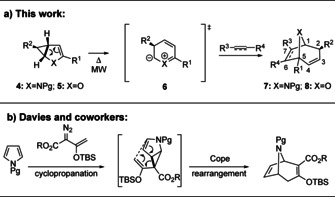
Strategies to tropanes via cyclopropanated pyrroles and furans.

Monocyclopropanated heteroarenes **4** and **5** (R^2^=CO_2_R) are readily available in diastereo‐ and enantiopure form by the cyclopropanation of pyrroles and furans with diazoacetates,^14^ all representing inexpensive, renewable resources. Such compounds have been proven to be of great synthetic value, being associated with the facile cleavage of the activated, *exocyclic*, donor‐acceptor substituted cyclopropane bonds.14e, 14f, 14g, 14h, 15 In the context of the synthesis of tropanes, this reactivity was most elegantly exploited by Davies and co‐workers with the cyclopropanation of pyrroles by vinyldiazoacetates followed by a Cope rearrangement (Scheme 1 b).8e, 8g, 16 In contrast, examples for the ring‐opening of the unactivated, *endocyclic* cyclopropane bond in **4** or **5** are rare.^17^ Nevertheless, we reasoned that the aromatic transition state of a 6π‐electrocyclic reaction leading to a transient 1,3‐dipol **6** could outcompete the typical ring‐opening pathway of the *exocyclic* cyclopropane bond via the push‐pull system present through the heteroatom donor and the ester group on the cyclopropane moiety. Trapping of **6** with dipolarophiles in a Huisgen [3+2]‐cycloaddition^18^ would then give direct access to 8‐azabicyclo[3.2.1]octanes **7** and 8‐oxabicyclo[3.2.1]octanes **8** (Scheme 1 a).

Cyclopropane **4 a** and dimethylacetylene dicarboxylate (DMAD) were used to test the validity of the envisioned 6π‐electrocyclic ring‐opening/ [3+2]‐cycloaddition cascade, which indeed proved to be possible under thermal conditions (Table 1). Appreciable reaction rates were only observed well above 100 °C (Table 1, Entry 1), giving rise to the cycloadduct **7 a** as single, *exo*‐diastereomer in 70 % yield upon conventional heating at 150 °C for 24 hours (Table 1, Entry 2). A dramatic acceleration of the reaction rate was observed upon changing to microwave heating, affording **7 a** in 77 % yield in a reaction time of only one hour (Table 1, Entry 3), which could be further shortened to 0.5 h/ 81 % yield when running the reaction neat (Table 1, Entry 4).

**Table 1:** Optimization of the reaction conditions. 
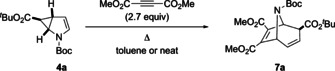

Entry	Solvent	*T* [°C]	Time [h]	Yield [%]
1	toluene	100	24	no conversion
2	toluene	150	24	70
3^[a]^	toluene	150	1	77
4^[a]^	neat	150	0.5	81

[a] Microwave irradiation was used.

With the optimal reaction conditions established, we investigated the scope of the reaction (Scheme 2, Scheme 3). Depending on the aggregation state of the dipolarophile the reaction was performed in minimal amounts of toluene (**A**, solid dipolarophile) or in the absence of solvent (**B**, liquid dipolarophile). Keeping subsequent functionalizations of adducts of type **7 a** in mind, we established with the synthesis of **7 a**–**f** that different combinations of ester groups stemming from **4** or the acetylenedicarboxylate as well as common nitrogen protecting groups (Ts, Boc) are amenable for this process. Enantiomerically pure **4 a** and **4 c** gave rise to (+)‐**7 a** and (−)‐**7 l** without any observable erosion of enantiopurity. Furthermore, the cycloadducts **7 a**, **7 c** and **7 d** were prepared on gram scale demonstrating the value of the developed protocol for synthetic applications. Starting from **4 d** in which the ester moiety on C‐2 was changed to an alcohol was also well tolerated giving rise to cycloadduct **7 g**, again as single, *exo*‐diastereomer in 75 % yield. Notably, in this case the reaction proceeded already at 100 °C, which could be either due to reduced steric congestion in the ring‐opening/ cycloaddition process or to more favorable electronics in the cycloaddition with an electron‐deficient dipolarophile. Switching from alkyne to alkene based cycloaddition partners was also well tolerated: Starting from pyrrole **4 e**, maleic anhydride afforded **7 h** and *N*‐phenylmaleimide **7 i** in 79 % and 64 % yield, respectively, both with perfect *endo*‐control of the approaching dipolarophile and *exo*‐placement of the ester moiety, which was unambiguously established by X‐ray structure analysis of **7 h**. Moreover, maleonitrile was successfully used as a dipolarophile in the cycloaddition with **4 e** and afforded *endo* cycloadduct **7 k** exclusively. The reaction with unsymmetrical dipolarophiles such as fumaronitrile or tosylacetylene gave the corresponding cycloadducts **7 j** and **7 m** in a *ratio* of 4.5:1 and 4:1, however, the latter was obtained in only low yields, pointing to the requirement of strongly electron‐poor dipolarophiles for the title reaction. Indeed, electron neutral or electron rich dipolarophiles failed to undergo the reaction sequence (see supporting information).

**Scheme 2:**
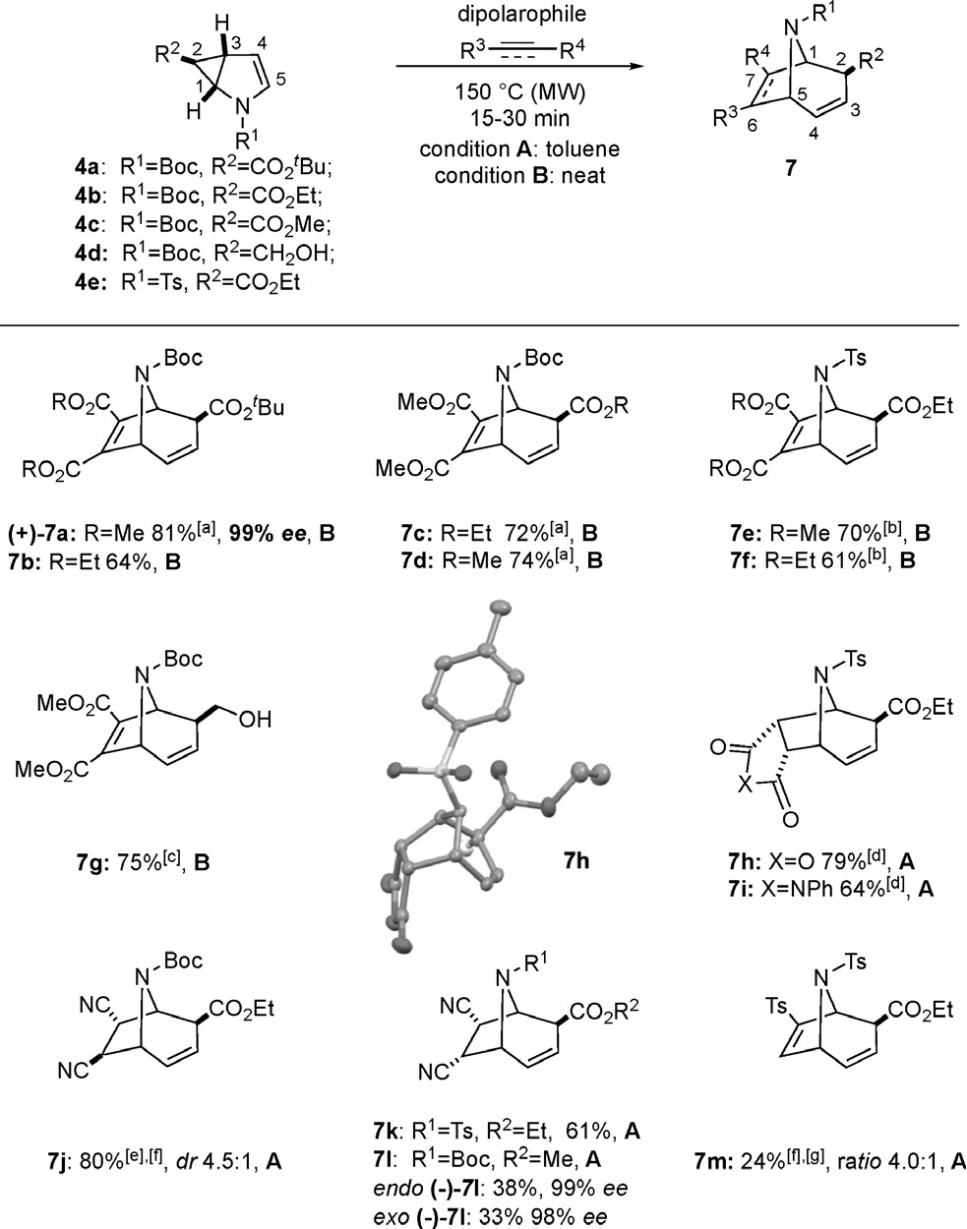
Substrate scope of [3+2]‐cycloadditions for the synthesis of 8‐azabicyclo[3.2.1]octanes **7**: 0.3–1 mmol **4**, dipolarophile (2.7 equiv); [a] Scale‐up: 4.39 mmol **4 a** were employed to yield 1.54 g of **7 a**; 4.03 mmol **4 b** were employed to yield 1.15 g of **7 c**; 4.18 mmol **4 c** were employed to yield 1.18 g of **7 d**; [b] 170 °C; [c] 100 °C; [d] 1 h, 1.1 equiv of dipolarophile; [e] Major diastereomer shown; [f] Combined isolated yield of two diastereomers; [g] Major regioisomer shown.

**Scheme 3:**
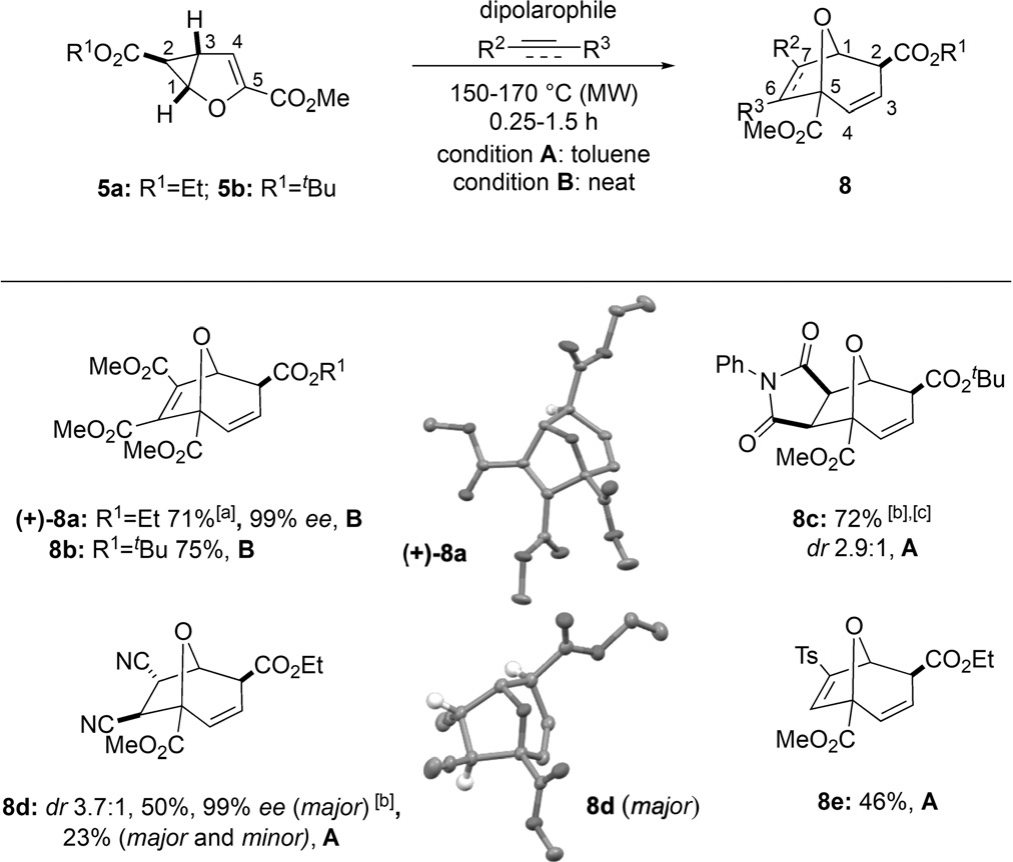
Substrate scope of [3+2]‐cycloadditions for the synthesis of 8‐oxabicyclo[3.2.1]octanes **8**: 0.4–2.7 mmol **5**, dipolarophile (2.7 equiv); [a] Scale‐up: 7.87 mmol (−)‐**5 a** were employed to yield 1.98 g of **(+)‐8 a**; [b] Major diastereomer shown. [c] Combined isolated yield of two diastereomers.

The tropane skeleton in **7 j** could be fully assigned by ^1^H NMR and 2D NMR. The coupling constants for protons H‐1 and H‐5 adjacent to the bridging nitrogen were indicative of the *exo* and *endo* relationship to H‐6 and H‐7. The spectroscopic data of **7 j** are in agreement with the data of the related tropane **8 d** (vide infra), for which an X‐Ray structure was obtained. The structure assignment for cycloadduct **7 m** was based on ^1^H NMR and 2D NMR.

Further expanding the scope to cyclopropanated furans **5** provides access to 8‐oxabicyclo[3.2.1]octanes **8** (Scheme 3),^6^, ^19^ being also key motifs in biological active compounds. DMAD was again identified as a suitable dipolarophile, resulting in the corresponding cycloadducts **8 a** and **8 b** as single, *exo* diastereomers in 71 % and 75 % yield, respectively. Enantiomerically pure cycloadduct (+)‐**8 a** (>99 % *ee*, confirmed by X‐ray analysis) could be obtained as a single stereoisomer in gram quantities using (−)‐**5 a**, highlighting once more the scalability of the developed protocol. Additionally, enantiomerically pure (−)‐**5 a** gave access to (−)‐**8 d** (major). The reaction between cyclopropanated furan derivative **5 b** and *N*‐phenylmaleimide gave rise to **8 c** (72 %, *dr* 2.9:1) from which *major* diastereomer could be isolated in pure form. Structure assignments for *exo*/*endo* **8 c** were based on ^1^H NMR and 2D NMR spectra. The *endo* and *exo* stereochemistry can be deduced from differences in the coupling constant of the bridgehead proton H‐1 to the *exo* or *endo* proton H‐7. Coupling of **5 a** and fumaronitrile resulted in **8 d** (73 %, *dr* 3.7:1) from which the *major*, presumably sterically favored diastereomer was isolated in pure form (50 % yield). Finally, tosylacetylene produced in the reaction with **5 a** single regioisomer **8 e** in 46 % yield most likely a consequence to the ester group in the bridgehead position, which was unambiguously assigned by ^1^H NMR and 2D NMR spectroscopy.

The [3+2]‐cycloaddition reactions proceeded in all cases with complete facial selectivity in that the incoming dipolarophile and the ester group at C‐2 orient *anti* to each other (TS‐1, Scheme 4) resulting in the *exo*‐orientation of the latter. In case of alkene dipolarophiles high *endo*‐control of the latter was observed in the pyrrole series (**7 h**, **7 i** and **7 k**), while in the furan series the *exo*‐orientation was preferred (**8 c**), being a consequence of the different steric demand of the heteroatom bridge (O vs. NTs). The approach reported here allows the introduction of various substituents with stereoselective control at the tropane skeleton, in particular at C‐6/C‐7 positions, comparing well to other approaches which often require multistep synthesis.8j, 8k, 19

**Scheme 4:**
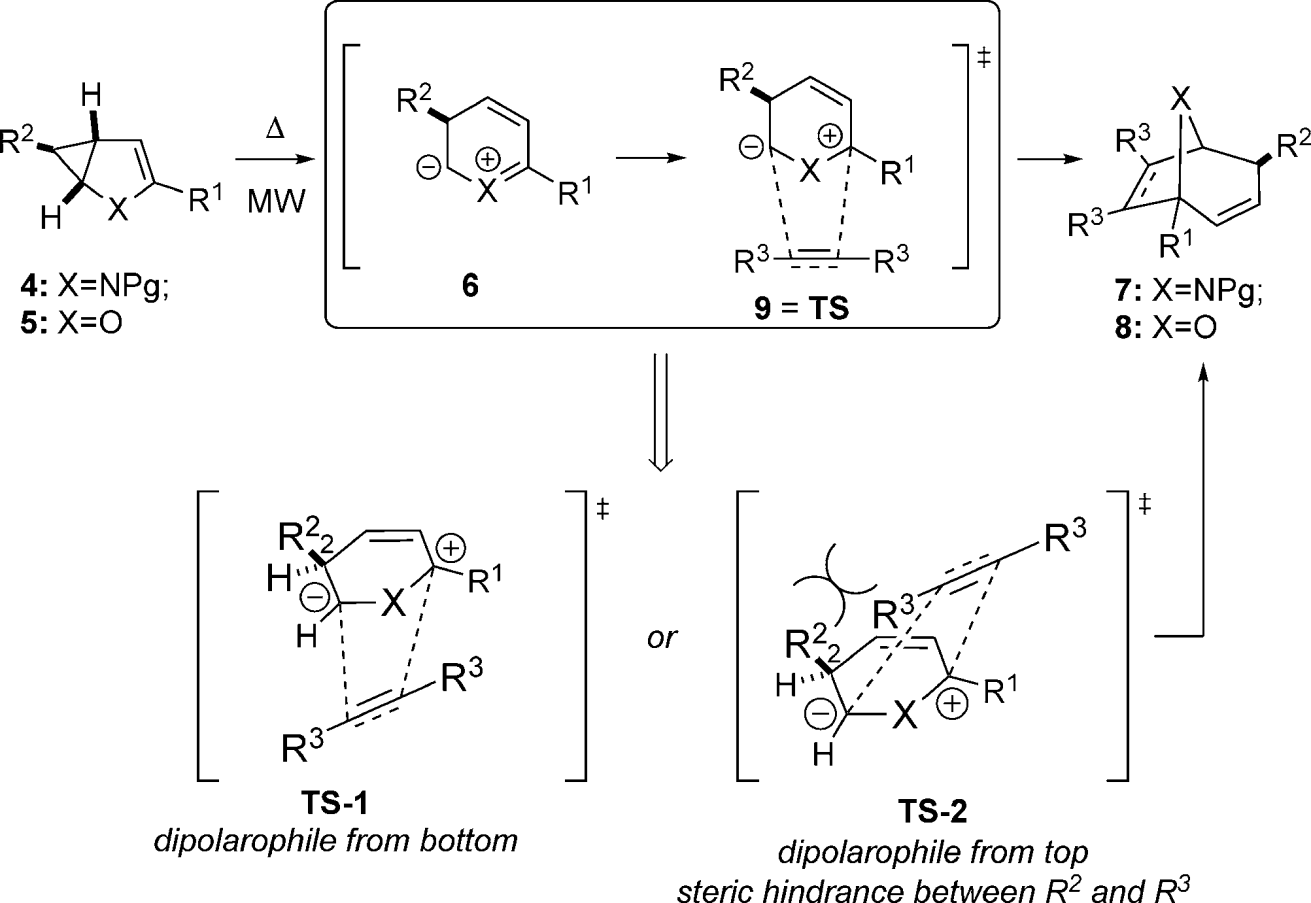
Stereochemical model for the [3+2]‐cycloaddition of **4** and **5**.

Furthermore, postfunctionalization of the scaffolds obtained is possible (Scheme 5, Scheme 6) allowing its further diversification.

**Scheme 5:**
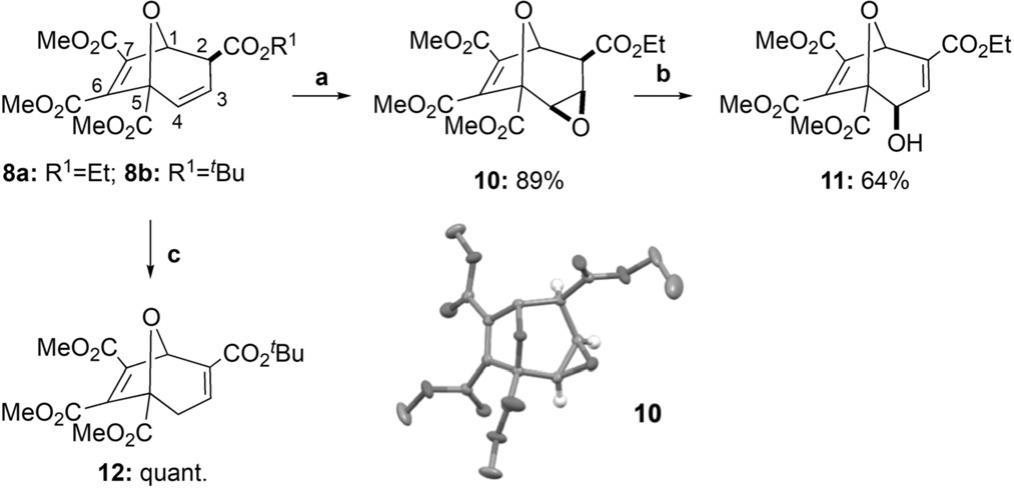
Derivatization reactions of the 8‐oxabicyclo[3.2.1]octane framework **8 a**,**b**. Conditions: **8 a**: a) *m*CPBA (3.5 equiv), CH_2_Cl_2_, 50 °C, 18 h, 89 %; b) flash chromatography, 1 % triethylamine (TEA), 64 %; **8 b**: c) TEA (1.3 equiv), CH_2_Cl_2_, 25 °C, 30 min, quant.

**Scheme 6:**
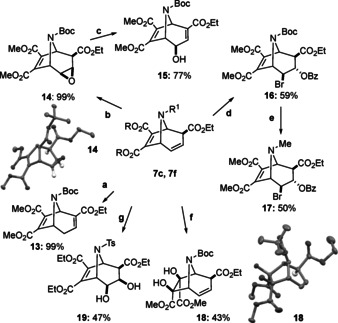
Derivatization reactions of the 8‐azabicyclo[3.2.1]octane framework **7 c** and **7 f**. Conditions: a) TEA (1.3 equiv), CH_2_Cl_2_, 2 h, 99 %; b) *m*CPBA (4.0 equiv), CH_2_Cl_2_, 25 °C, 3 days, 99 %; c) flash chromatography, 1 % TEA, 77 %; d) (i) NBS (2.0 equiv), acetone/H_2_O (3:1 v/v), 0 to 25 °C, 21 h; (ii) BzCl (1.5 equiv), DMAP (0.5 equiv), TEA (5.0 equiv), CH_2_Cl_2_, 25 °C, 8 h, 59 %; e) (i) TFA (33 equiv), CH_2_Cl_2_, 25 °C, 1.5 h, (ii) 37 % aq CH_2_O (6.0 equiv), NaBH_3_CN (3.0 equiv), MeCN, 25 °C, 1 h, 50 %; f) K_2_OsO_4_⋅2 H_2_O (0.05 equiv), NMO (2.0 equiv), H_2_O, acetone, 0 °C, 12 h, 43 %; g) RuCl_3_⋅3 H_2_O (6 mol %), NaIO_4_ (1.6 equiv), MeCN, H_2_O, 0 to 25 °C, 2 days, 47 %.

Epoxidation of 8‐oxabicyclo[3.2.1]octane **8 a** with *meta*‐chloroperoxybenzoic acid (*m*CPBA) proceeded selectively from the less‐hindered convex side to give *exo*‐epoxide **10** in 89 % yield, which was further converted under basic conditions to allylic alcohol **11** in 64 % yield. In turn, the isomerization of the C‐3,4 double bond in **8 b** to the thermodynamically favored enone **12** can be quantitatively achieved under basic conditions with TEA.

In an analogous way, derivatives **13**–**15** were obtained in the nitrogen series in excellent yields (Scheme 6). Bromohydrin formation of **7 c** with *N*‐bromosuccinimide (NBS) in the presence of water (confirmed by X‐ray structure analysis; see supporting information) followed by esterification with BzCl proceeded with remarkable diastereoselectivity to **16**. Finally, deprotection and reductive amination of **16** in one pot8i to **17** demonstrates the exchange of the *N*‐Boc protecting group to *N*‐Me as typically found in natural products (see Scheme 1). Dihydroxylation of **7 c** in the presence of K_2_OsO_4_/*N*‐methylmorpholine *N*‐oxide (NMO) in acetone‐water (3:1) proceeded in moderate yield but surprisingly, furnished exclusively *exo*‐diol **18**, as single diastereomer. In contrast, the dihydroxylation of **7 f** with RuCl_3_⋅3 H_2_O, and NaIO_4_ resulted selectively in diol **19**. These alcohols have potential for the synthesis of calystegines analogues being polyhydroxy bicyclic nortropane alkaloids (see Scheme 1).

The tropane scaffold can also be readily rearranged to the isoquinuclidine scaffold (Scheme 7) by subjecting bromo compounds such as **20** to AIBN/Bu_3_SnH upon which a homoallylic radical rearrangement to **21** takes place.^20^

**Scheme 7:**
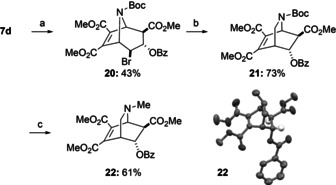
Isoquinuclidine synthesis by homoallylic radical rearrangement. Conditions: (a) (i) NBS (4.0 equiv), acetone/H_2_O (4:1 v/v), 0 to 25 °C, 3 days; (ii) BzCl (1.5 equiv), DMAP (0.5 equiv), TEA (5.0 equiv), CH_2_Cl_2_, 25 °C, 15 h, 43 %; (b) azobisisobutyronitrile (AIBN) (0.1 equiv), Bu_3_SnH (1.6 equiv), benzene, reflux, 5 h, 73 %; (c) (i) TFA (33 equiv), CH_2_Cl_2_, 25 °C, 1 h, (ii) 37 % aq CH_2_O (11 equiv), NaBH_3_CN (8.2 equiv), MeCN, 25 °C, 1.5 h, 61 %.

Finally, addressing the lack of using electron neutral or donating dipolarophiles that can be used in the reaction cascade, initial results on manipulating the methyl ester groups in **7 a** were obtained (Scheme 8). Hydrogenation followed by saponification gave rise to **24** in excellent yield, however, an equilibration to the sterically less hindered *trans*‐arrangement of the carboxylic acid groups had taken place as well. Subsequent lead tetraacetate degradation of **24** gave rise to **25**, moreover, the carboxylic acids can be reduced to alcohols and further transformed to the corresponding bromides (see supporting information for details). Direct saponification of **7 a** gave rise to 6‐azatricyclo[3.2.1.0^2,7^]octane **23**, a scaffold that is prominently found in its carbocyclic version in several natural products.^21^

**Scheme 8:**
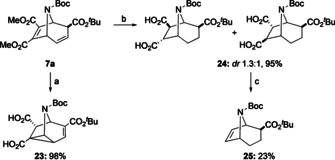
Base‐induced formation of 6‐azatricyclo[3.2.1.0^2, 7^]octane **23** (a) and oxidative degradation of diacid **24** to **25** (b,c). Conditions: a) NaOH (2.0 equiv), THF, 0 to 25 °C, 5.5 h, then HCl, 0 °C, 98 %; b) (i) H_2_, Pd/C (10 mol %), EtOH/THF (1:4 v/v), 60 bar, 25 °C, 4 h, (ii) NaOH (2.0 equiv), THF, 0 to 25 °C, 4 h, then HCl, 0 °C, 95 %; c) Pb(OAc)_4_ (2.4 equiv), C_5_H_5_N, 67 °C, 6 h, 23 %.

In conclusion, starting from commercially available furans and pyrroles, a short sequence was developed which offers the stereoselective assembly of oxo‐ and aza‐bicyclo‐[3.2.1] and [2.2.2]‐scaffolds, being highly relevant for natural products and drugs. As the key step, a transient 1,3‐dipol is generated by a electrocyclic 6π ring‐opening reaction of 2‐oxa‐ or 2 aza‐bicyclo[2.1.0]‐hex‐3‐enes, can be efficiently trapped with various dipolarophiles, demonstrating once again that the Huisgen [3+2]‐cycloaddition is one of the most powerful synthetic transformation for the construction of heterocycles in our time.

## Conflict of interest

The authors declare no conflict of interest.

## Supporting information

As a service to our authors and readers, this journal provides supporting information supplied by the authors. Such materials are peer reviewed and may be re‐organized for online delivery, but are not copy‐edited or typeset. Technical support issues arising from supporting information (other than missing files) should be addressed to the authors.

Supplementary
