# Supplementary material for: Stereoselective Synthesis of Tropanes via a 6π‐Electrocyclic Ring‐Opening/ Huisgen [3+2]‐Cycloaddition Cascade of Monocyclopropanated Heterocycles
Source: Angew Chem Int Ed Engl. 2020 Aug 18;59(41):18110–5. doi: 10.1002/anie.202006030 (PMC7589232; doi:10.1002/anie.202006030)
Supplement: Supplementary file 1 — Supplementary [file ANIE-59-18110-s001.pdf]

Angewandte Chemie
Eine Zeitschrift der Gesellschaft Deutscher Chemiker

Supporting Information

Stereoselective Synthesis of Tropanes via a 6π-Electrocyclic Ring-Opening/ Huisgen [3+2]-Cycloaddition Cascade of Monocyclopropanated Heterocycles

*Carina M. Sonnleitner, Saerom Park, Robert Eckl, Thomas Ertl, and Oliver Reiser**

anie_202006030_sm_miscellaneous_information.pdf

Table of Contents

1. General information 3
2. Experimental procedures and analytical data 4
   2.1. Synthesis of cyclopropanes 4
   2.2. [3+2]-Cycloaddition reactions 6
   2.3. Derivatization reactions 25
3. NMR spectra 42
4. X-Ray structures 85
5. HPLC chromatograms 96
6. References 101

1. General information

All moisture sensitive reactions were performed in flame-dried glassware under nitrogen atmosphere. Commercially available chemicals were used as purchased. Anhydrous solvents were prepared according to standard procedures. Analytical thin layer chromatography was performed on Silica gel 60 F254 aluminium plates (Merck). Visualization was accomplished using UV-irradiation (λ = 254 nm), vanillin/sulfuric acid solution, potassium permanganate solution, bromocresol green or Seebach's stain. Column chromatography was carried out on silica gel Merck Geduran 60 (0.063-0.200 mm) and flash silica gel Merck Geduran Si 60 (0.040-0.063 mm). Furthermore, purification by flash system was performed with silica gel (Merck, 0.040-0.063 mm) on a Reveleris® X2 Flash System (Büchi). Melting points were recorded on Stanford Research Systems OptiMelt MPA 100 Automated melting point system. ^{1}H NMR and ^{13}C NMR spectra were recorded on Bruker Avance 300 MHz and Bruker Avance 400 MHz spectrometers. Chemical shifts for ^{1}H NMR were reported as δ, parts per million, relative to the signal of $CHCl_3$ at 7.26 ppm. Chemical shifts for ^{13}C NMR were reported as δ, parts per million, relative to the signal of $CHCl_3$ at 77.2 ppm and TMS as an internal standard. Coupling constants (*J*) are given in Hertz (Hz). The following notations indicate the multiplicity of the signals: s = singlet, bs = broad singlet, d = doublet, t = triplet, q = quartet, dd = doublet of doublets, dt = doublet of triplets, dq = doublet of quartet, td = triplet of doublets, ddd = doublet of doublet of doublets, ddt = doublet of doublet of triplets, dddd = doublet of doublet of doublet of doublets and m = multiplet. ^{13}C NMR: (+) = primary/tertiary, (−) = secondary, (q) = quaternary carbon. The assignment resulted from DEPT, COSY, HSQC, HMBC and NOESY experiments. FTIR was carried out on a spectrometer equipped with a Diamon Single Reflection ATR-SYSTEM. The samples were prepared as thin films. Mass spectra were recorded at the Central Analytical Laboratory at the Department of Chemistry of the University of Regensburg on Agilent Technologies 6540 UHD Accurate-Mass Q-TOF LC/MS. Optical rotations [α] were determined using Perkin Elmer 241 polarimeter at λ = 589 nm (sodium-*d*-line) in a 1.0 dm measuring cell and the specified solvent. X-ray analysis was performed on Agilent Technologies SuperNova and Agilent Technologies GV 1000. Analytical high-performance liquid chromatography (HPLC) was conducted on a Varian 920-LC chromatograph equipped with Diode Array detector. Phenomenex Lux Cellulose-1, Phenomenex Lux Cellulose-2, Chiracel AS-H, Chiracel OJ-H and Chiralpak AS-H served as chiral stationary phase and mixtures of *n*-heptane and *i*-PrOH were used for elution. Microwave irradiation experiments were carried out using an Anton Paar Monowave 300 reactor.

2. Experimental procedures and analytical data

Following compounds were synthesized according to literature procedures and spectroscopic data matched well with those reported:

Tert-butyl diazoacetate[1], furan-2-carboxylic acid methyl ester[2], 2,2-bis((4*S*)-(–)-4-isopropyloxazoline) propane[3], ethyl 2-diazoacetate[4], 1-tosyl-1*H*-pyrrole[5], *tert*-butyl-1*H*-pyrrole-1-carboxylate[6], ethynyl *p*-tolyl sulfone[7], compounds **4a**[8], **(−)-4a**[8], **4c**[8], **(−)-4c**[8], **4d**[9], **5a**[10], **(−)-5a**[10], **5b**[10].

2.1. Synthesis of cyclopropanes

2-(*Tert*-butyl) 6-ethyl (1*S*,5*S*,6*S*)-2-azabicyclo[3.1.0]hex-3-ene-2,6-dicarboxylate (4b)

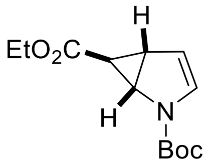

According to literature procedure[8], a flame-dried schlenk flask was charged with $Cu(OTf)_2$ (482 mg, 1.33 mmol, 0.01 equiv) and dry CH_2Cl_2 (5 mL) under N_2-atmosphere. *Tert*-butyl-1*H*-pyrrole-1-carboxylate (22.3 g, 22.3 mL, 133 mmol, 1.0 equiv) was dissolved in dry CH_2Cl_2 (42 mL) and the $Cu(OTf)_2$ solution was added at 25 °C. Subsequently, phenylhydrazine (144 mg, 131 µL, 1.33 mmol, 0.01 equiv) was added. Afterwards, ethyl 2-diazoacetate (22.8 g, 9.73 wt%, 200 mmol, 1.5 equiv) in CH_2Cl_2 was added dropwise to the reaction mixture *via* syringe pump (addition rate: 1 drop/10 s). The reaction mixture was filtered through a plug of basic Al_2O_3 and washed with CH_2Cl_2 (800 mL). The solvent was removed under reduced pressure and the crude product was purified by silica gel column chromatography (17% EA:PE) to obtain cyclopropane **4b** (14.0 g, 55.3 mmol, 42%) as a yellow oil. Recrystallization from pentane yielded pure cyclopropane **4b** (10.9 g, 43.1 mmol, 32%) as a colorless solid.

R_f = 0.78 (PE:EA = 2:1; $KMnO_4$, vanillin); **m.p.** = 43 °C; **^{1}H NMR** (400 MHz, $CDCl_3$): δ = 6.71 – 6.28 (m, 1H), 5.45 – 5.23 (m, 1H), 4.44 – 4.21 (m, 1H), 4.16 – 4.04 (m, 2H), 2.88 – 2.62 (m, 1H), 1.48 (s, 9H), 1.26 – 1.20 (m, 3H), 0.97 – 0.85 (m, 1H) (signal broadening and doubling due to rotamers); **^{13}C NMR** (101 MHz, $CDCl_3$): δ = 173.2 (q), 172.9 (q), 151.3 (q), 151.0 (q), 129.8 (+), 129.6 (+), 109.9 (+), 81.7 (q), 60.6 (–), 44.3 (+), 44.1 (+), 32.2 (+), 31.0 (+), 28.3 (+), 23.1 (+), 23.0 (+), 14.3 (+) (signal broadening and doubling due to rotamers); **IR** $\tilde{\nu}$ [cm^{-1}]: 3097, 3049, 2982, 2940, 2904, 1700, 1588, 1474, 1461, 1398, 1342, 1262, 1249, 1167, 1141, 1044,

1013, 939, 937, 902, 831, 814, 764, 729, 719; **HRMS** (ESI): calcd. for $C_{13}H_{19}NO_4$ $(M+H)^+$, m/z = 254.1387; found 254.1391.

Ethyl (1*S*,5*S*,6*S*)-2-tosyl-2-azabicyclo[3.1.0]hex-3-ene-6-carboxylate (4e)

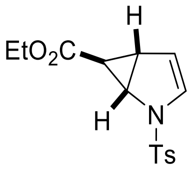

According to literature procedure[8], a flame-dried schlenk flask was charged with $Cu(OTf)_2$ (361 mg, 998 µmol, 0.01 equiv) and dry CH_2Cl_2 (10 mL) under N_2-atmosphere. 1-Tosyl-1*H*-pyrrole (22.1 g, 99.8 mmol, 1.0 equiv) was dissolved in dry CH_2Cl_2 (60 mL) and the $Cu(OTf)_2$ solution was added at 25 °C. Subsequently, phenylhydrazine (108 mg, 98 µL, 998 µmol, 0.01 equiv) was added. Afterwards, ethyl 2-diazoacetate (17.1 g, 10.7 wt%, 150 mmol, 1.5 equiv) in CH_2Cl_2 was added dropwise to the reaction mixture *via* syringe pump (addition rate: 1 drop/10 s). The reaction mixture was filtered through a plug of basic Al_2O_3 and washed with CH_2Cl_2 (800 mL). The solvent was removed under reduced pressure and the crude product was purified by silica gel column chromatography (10 → 17% EA:PE) to obtain cyclopropane **4e** (10.5 g, 34.2 mmol, 34%) as a colorless solid.

$\mathbf{R_f}$ = 0.36 (PE:EA = 5:1; $KMnO_4$, vanillin); **m.p.** = 66 °C; ^{1}**H NMR** (400 MHz, $CDCl_3$): δ = 7.66 (d, *J* = 8.3 Hz, 2H), 7.32 (d, *J* = 8.1 Hz, 2H), 6.33 (d, *J* = 3.9 Hz, 1H), 5.45 (dd, *J* = 3.9, 2.7 Hz, 1H), 4.22 – 3.98 (m, 3H), 2.70 (dt, *J* = 6.1, 2.6 Hz, 1H), 2.44 (s, 3H), 1.24 (t, *J* = 7.1 Hz, 3H), 0.48 – 0.43 (m, 1H); ^{13}**C NMR** (101 MHz, $CDCl_3$): δ = 172.2 (q), 144.5 (q), 133.7 (q), 130.5 (+), 130.1 (+), 127.6 (+), 113.8 (+), 61.0 (−), 45.4 (+), 31.6 (+), 21.8 (+), 21.1 (+), 14.4 (+); **IR** $\tilde{\nu}$ [cm^{-1}]: 3124, 2986, 2907, 1715, 1588, 1495, 1446, 1402, 1379, 1346, 1290, 1163; **HRMS** (ESI): calcd. for $C_{15}H_{17}NO_4S$ $(M+H)^+$, m/z = 308.0951; found 308.0957.

2.2. [3+2]-Cycloaddition reactions

General procedures for dipolar cycloaddition (GP-1a, GP-1b):

General procedure 1a (GP-1a): Conditions I

A microwave vial equipped with a magnetic stirring bar was charged with cyclopropane **4a**, **4b**, **4c**, **4d**, **4e**, **5a**, **5b** (1.0 equiv) and dipolarophile (2.7 equiv). The mixture was stirred for 15-30 min at 150-170 °C under microwave irradiation. The solution was concentrated under reduced pressure and crude product was purified by column chromatography using EA:PE as eluent.

General procedure 1b (GP-1b): Conditions II

A microwave vial equipped with a magnetic stirring bar was charged with cyclopropane **4b**, **4c, 4e, 5a, 5b** (1.0 equiv), dipolarophile (1.1-2.7 equiv) and toluene. The mixture was stirred for 0.25-1.5 h at 150-170 °C under microwave irradiation. The solution was concentrated under reduced pressure and crude product was purified by column chromatography using EA:PE as eluent.

2,8-Di-*tert*-butyl 6,7-dimethyl (1*S*,2*S*,5*R*)-8-azabicyclo[3.2.1]octa-3,6-diene-2,6,7,8-tetracarboxylate ((+)-7a)

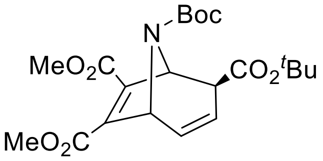

Following GP-1a-Conditions I, **(+)-7a** was prepared from cyclopropane **(−)-4a** (103 mg, 365 µmol, 1.0 equiv) and DMAD (140 mg, 120 µL, 987 µmol, 2.7 equiv). The mixture was heated for 30 min at 150 °C under microwave irradiation. The crude product was purified by flash system (5 → 13% EA:PE) to afford cycloadduct **(+)-7a** (125 mg, 295 µmol, 81%, 99% *ee*) as a colorless solid.

R_f = 0.58 (PE:EA = 3:1; UV, $KMnO_4$); **m.p.** = 51 °C; **HPLC analysis:** 99% *ee* (Phenomenex Lux Cellulose-2, *n*-heptane/*i*-propanol 90:10, 1.0 mL/min, 215 nm): t_r = 7.82 min; $[\alpha]_D^{20}$ = +73.2 (c = 1.0 in $CHCl_3$); **^{1}H NMR** (300 MHz, $CDCl_3$): δ = 6.35 (ddd, *J* = 9.6, 5.3, 2.1 Hz, 1H), 5.67 (ddd, *J* = 9.6, 3.9, 1.8 Hz, 1H), 5.53 (dt, *J* = 1.8, 0.9 Hz, 1H), 5.19 – 4.70 (m, 1H), 3.82 (s, 3H), 3.82 (s, 3H), 3.09 (ddd, *J* = 3.8, 2.1, 1.0 Hz, 1H), 1.49 (s, 9H), 1.45 (s, 9H) (signal broadening

due to rotamers); **^{13}C NMR** (101 MHz, $CDCl_3$): δ = 169.0 (q), 163.5 (q), 162.8 (q), 152.6 (q), 148.7 (q), 138.3 (q), 130.6 (+), 124.7 (+), 82.0 (q), 80.9 (q), 62.0 (+), 58.8 (+), 52.57 (+), 52.55 (+), 43.3 (+), 28.3 (+), 28.1 (+) (signal broadening due to rotamers); **IR** $\tilde{\nu}$ [cm^{-1}]: 2982, 1707, 1640, 1435, 1312, 1256, 1156, 1081, 1025, 947, 846, 783; **HRMS** (ESI): calcd. for $C_{21}H_{29}NO_8$ $(M+H)^+$, m/z = 424.1966, found 424.1969.

2,8-Di-*tert*-butyl 6,7-dimethyl (1*S*,2*S*,5*R*)-8-azabicyclo[3.2.1]octa-3,6-diene-2,6,7,8-tetracarboxylate (7a)

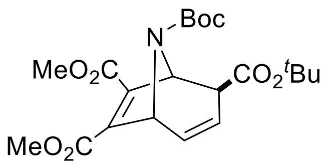

Following GP-1a-Conditions I, **7a** was prepared from cyclopropane **4a** (1.24 g, 4.39 mmol, 1.0 equiv) and DMAD (1.68 g, 1.45 mL, 11.9 mmol, 2.7 equiv). The mixture was heated for 30 min at 150 °C under microwave irradiation. The crude product was purified by flash system (5 → 13% EA:PE) to afford cycloadduct **7a** (1.54 g, 3.64 mmol, 83%) as a colorless solid.

NMR, m.p. and IR data were identical with those reported for the enantiomer **(+)-7a**.

2,8-Di-*tert*-butyl 6,7-diethyl (1*S*,2*S*,5*R*)-8-azabicyclo[3.2.1]octa-3,6-diene-2,6,7,8-tetracarboxylate (7b)

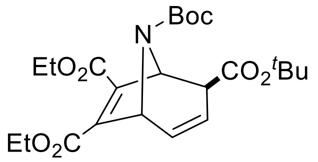

Following GP-1a-Conditions I, **7b** was prepared from cyclopropane **4a** (56.0 mg, 199 µmol, 1.0 equiv) and DEAD (140 mg, 86 µL, 537 µmol, 2.7 equiv). The mixture was heated for 30 min at 150 °C under microwave irradiation. The crude product was purified by flash column chromatography (5 → 13% EA:PE) to afford cycloadduct **7b** (57.9 mg, 130 µmol, 64%) as a colorless oil.

R_f = 0.30 (PE:EA = 5:1; UV, $KMnO_4$); **^{1}H NMR** (400 MHz, $CDCl_3$): δ = 6.34 (ddd, *J* = 9.6, 5.3, 2.1 Hz, 1H), 5.66 (ddd, *J* = 9.6, 3.9, 1.8 Hz, 1H), 5.50 (dt, *J* = 1.8, 0.9 Hz, 1H), 5.17 – 4.70 (m, 1H), 4.40 – 4.16 (m, 4H), 3.09 (ddd, *J* = 3.4, 2.2, 0.9 Hz, 1H), 1.48 (s, 9H), 1.44 (s, 9H), 1.35 – 1.27 (m, 6H) (signal broadening due to rotamers); **^{13}C NMR** (75 MHz, $CDCl_3$) δ = 169.0 (q), 163.1 (q), 162.5 (q), 152.4 (q), 138.1 (q), 135.0 (q), 130.7 (+), 124.6 (+), 81.8 (q), 80.8 (q), 62.0 (+), 61.6 (–), 61.5 (–), 58.6 (+), 43.2 (+), 28.2 (+), 28.0 (+), 14.0 (+) (signal broadening due to rotamers); **IR** ṽ [cm^{-1}]: 2982, 2937, 1707, 1640, 1476, 1457, 1392, 1368, 1305, 1252, 1159, 1115, 1077, 1033, 869, 844, 775; **HRMS** (ESI): calcd. for $C_{23}H_{33}NO_8$ $(M+H)^+$, m/z = 452.2279, found 452.2281.

8-(*Tert*-butyl) 2-ethyl 6,7-dimethyl (1*S*,2*S*,5*R*)-8-azabicyclo[3.2.1]octa-3,6-diene-2,6,7,8-tetracarboxylate (7c)

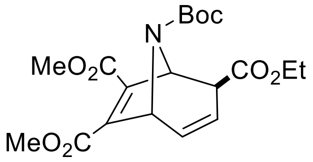

Following GP-1a-Conditions I, **7c** was prepared from cyclopropane **4b** (1.02 g, 4.03 mmol, 1.0 equiv) and DMAD (1.55 g, 1.33 mL, 10.9 mmol, 2.7 equiv). The mixture was heated for 30 min at 150 °C under microwave irradiation. The crude product was purified by flash system (6 → 15% EA:PE) to afford cycloadduct **7c** (1.15 g, 2.91 mmol, 72%) as a yellowish oil.

R_f = 0.62 (PE:EA = 2:1; $KMnO_4$); **^{1}H NMR** (300 MHz, $CDCl_3$): δ = 6.38 (ddd, *J* = 9.7, 5.2, 2.1 Hz, 1H), 5.69 (ddd, *J* = 9.6, 3.7, 1.8 Hz, 1H), 5.55 – 5.42 (m, 1H), 5.13 – 4.72 (m, 1H), 4.26 – 4.15 (m, 2H), 3.81 (s, 3H), 3.80 (s, 3H), 3.14 (ddd, *J* = 3.5, 2.2, 0.9 Hz, 1H), 1.42 (s, 9H), 1.28 (t, *J* = 7.1 Hz, 3H) (signal broadening due to rotamers); **^{13}C NMR** (101 MHz, $CDCl_3$): δ = 169.9 (q), 163.2 (q), 162.6 (q), 152.3 (q), 149.0 (q), 137.8 (q), 131.1 (+), 124.1 (+), 80.9 (q), 61.7 (+), 61.5 (–), 58.4 (+), 52.5 (+), 42.2 (+), 28.1 (+), 14.1 (+) (signal broadening due to rotamers); **IR** ṽ [cm^{-1}]: 2982, 1703, 1640, 1435, 1390, 1249, 1163, 1118, 1077, 1028, 947, 887, 857, 760; **HRMS** (ESI): calcd. for $C_{19}H_{25}NO_8$ $(M+H)^+$, m/z = 418.1472; found 418.1474.

8-(*Tert*-butyl) 2,6,7-trimethyl (1*S*,2*S*,5*R*)-8-azabicyclo[3.2.1]octa-3,6-diene-2,6,7,8-tetracarboxylate (7d)

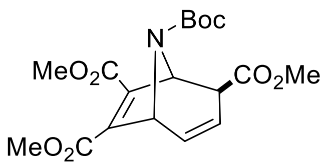

Following GP-1a-Conditions I, **7d** was prepared from cyclopropane **4c** (1.00 g, 4.18 mmol, 1.0 equiv) and DMAD (1.60 g, 1.38 mL, 11.3 mmol, 2.7 equiv). The mixture was heated for 30 min at 150 °C under microwave irradiation. The crude product was purified by flash system (6 → 20% EA:PE) to afford cycloadduct **7d** (1.18 g, 3.09 mmol, 74%) as a yellowish oil.

R_f = 0.56 (PE:EA = 2:1; UV, $KMnO_4$); **^{1}H NMR** (400 MHz, $CDCl_3$): δ = 6.37 (ddd, *J* = 10.1, 5.3, 2.0 Hz, 1H), 5.66 (ddd, *J* = 9.7, 3.8, 1.8 Hz, 1H), 5.47 – 5.41 (m, 1H), 5.11 – 4.71 (m, 1H), 3.79 (s, 3H), 3.78 (s, 3H), 3.73 (s, 3H), 3.22 – 3.05 (m, 1H), 1.40 (s, 9H) (signal broadening due to rotamers); **^{13}C NMR** (101 MHz, $CDCl_3$): δ = 170.5 (q), 163.1 (q), 162.6 q), 152.2 (q), 148.6 (q), 137.6 (q), 131.3 (+), 124.0 (+), 81.0 (q), 61.8 (+), 58.3 (+), 52.5 (+), 42.0 (+), 28.2 (+) (signal broadening due to rotamers); **IR** ṽ [cm^{-1}]: 2956, 1700, 1640, 2435, 1435, 1394, 1312, 1249, 1163, 1118, 1077, 1025, 947, 857, 760; **HRMS** (ESI): calcd. for $C_{18}H_{23}NO_8$ $(M+NH_4)^+$, m/z = 399.1762, found 399.1762.

2-Ethyl 6,7-dimethyl (1*S*,2*S*,5*R*)-8-tosyl-8-azabicyclo[3.2.1]octa-3,6-diene-2,6,7-tricarboxylate (7e)

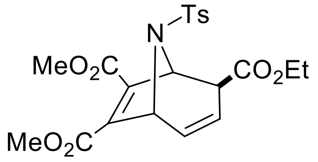

Following GP-1a-Conditions I, **7e** was prepared from cyclopropane **4e** (102 mg, 332 µmol, 1.0 equiv) and DMAD (127 mg, 110 µL, 896 µmol, 2.7 equiv). The mixture was heated for 15 min at 170 °C under microwave irradiation. The crude product was purified by flash system (16 → 41% EA:PE) to afford cycloadduct **7e** (105 mg, 234 µmol, 70%) as a colorless oil.

R_f = 0.43 (PE:EA = 2:1; UV, $KMnO_4$); **^{1}H NMR** (400 MHz, $CDCl_3$): δ = 7.64 – 7.60 (m, 2H), 7.24 (d, *J* = 8.0 Hz, 2H), 6.35 (ddd, *J* = 9.5, 5.8, 2.1 Hz, 1H), 5.71 (ddd, *J* = 9.5, 4.0, 1.6 Hz, 1H), 5.44 – 5.40 (m, 1H), 4.85 (dd, *J* = 5.8, 1.1 Hz, 1H), 4.26 – 4.14 (m, 2H), 3.69 (s, 3H), 3.68 (s,

3H), 3.16 (ddd, J = 3.7, 2.1, 1.0 Hz, 1H), 2.38 (s, 3H), 1.29 (t, J = 7.1 Hz, 3H); **^{13}C NMR** (101 MHz, $CDCl_3$): δ = 169.0 (q), 162.6 (q), 161.8 (q), 147.8 (q), 144.2 (q), 135.4 (q), 134.3 (q), 130.2 (+), 129.9 (+), 128.0 (+), 124.4 (+), 64.5 (+), 61.9 (–), 61.5 (+), 52.41 (+), 52.36 (+), 43.5 (+), 21.6 (+), 14.1 (+); **IR** $\tilde{\nu}$ [cm^{-1}]: 2982, 2956, 2931, 2856, 1711, 1644, 1599, 1439, 1349, 1287, 1245, 1163, 1126, 1088, 1022, 1021, 965, 936, 857, 816, 782, 753, 706, 690; **HRMS** (ESI): calcd. for $C_{21}H_{23}NO_8S$ $(M+H)^+$, m/z = 450.1217, found 450.1211.

Triethyl (1*S*,2*S*,5*R*)-8-tosyl-8-azabicyclo[3.2.1]octa-3,6-diene-2,6,7-tricarboxylate (7f)

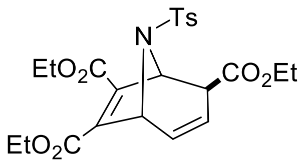

Following GP-1b-Conditions II, **7f** was prepared from cyclopropane **4e** (123 mg, 400 µmol, 1.0 equiv) and diethyl acetylenedicarboxylate (200 µL, 212 mg, 1.25 mmol, 3.0 equiv) in toluene (0.4 mL). The mixture was heated for 30 min at 150 °C under microwave irradiation. The crude product was purified by flash system (5 → 13% EA:PE) to afford cycloadduct **7f** (115 mg, 240 µmol, 61%) as a colorless oil.

R_f = 0.38 (PE:EA = 2:1; UV; $KMnO_4$); **^{1}H NMR** (300 MHz, $CDCl_3$): δ = 7.66 – 7.57 (m, 2H), 7.22 (d, J = 8.0 Hz, 2H), 6.35 (ddd, J = 9.5, 5.8, 2.1 Hz, 1H), 5.71 (ddd, J = 9.5, 3.9, 1.6 Hz, 1H), 5.41 (q, J = 1.3 Hz, 1H), 4.83 (dd, J = 5.8, 1.1 Hz, 1H), 4.27 – 4.06 (m, 6H), 3.16 (ddd, J = 3.5, 2.2, 1.1 Hz, 1H), 2.36 (s, 3H), 1.29 (t, J = 7.1 Hz, 3H), 1.24 – 1.18 (m, 6H); **^{13}C NMR** (75 MHz, $CDCl_3$): δ = 170.0 (q), 162.3 (q), 161.4 (q), 147.4 (q), 143.9 (q), 135.2 (q), 134.3 (q), 130.2 (+), 129.9 (+), 127.9 (+), 124.3 (+), 64.6 (+), 61.9 (–), 61.54 (+), 61.47 (–), 61.4 (–), 43.5 (+), 21.6 (+), 14.1 (+), 14.0 (+); **IR** $\tilde{\nu}$ [cm^{-1}]: 2982, 2933, 2908, 1711, 1640, 1599, 1447, 1446, 1394, 1370, 1353, 1286, 1241, 1163, 1091, 1081, 1036, 965, 938, 910, 855, 816, 735, 705, 687; **HRMS** (ESI): calcd. for $C_{23}H_{27}NO_8S$ $(M+Na)^+$, m/z = 500.1349; found 500.1350.

8-(*Tert*-butyl) 6,7-dimethyl (1*R*,4*S*,5*S*)-4-(hydroxymethyl)-8-azabicyclo[3.2.1]octa-2,6-diene-6,7,8-tricarboxylate (7g)

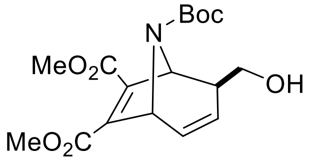

Following GP-1a-Conditions I, **7g** was prepared from cyclopropane **4d** (96.0 mg, 454 µmol, 1.0 equiv) and DMAD (174 mg, 150 µL, 1.23 mmol, 2.7 equiv). The mixture was heated for 30 min at 100 °C under microwave irradiation. The crude product was purified by flash system (3 → 40% EA:PE) to afford cycloadduct **7g** (120 mg, 340 µmol, 75%) as a colorless oil.

R*f* = 0.31 (PE:EA = 1:1; $KMnO_4$); **^{1}H NMR** (300 MHz, $CDCl_3$): δ = 6.33 (ddd, *J* = 9.7, 5.1, 1.9 Hz, 1H), 5.46 (ddd, *J* = 9.6, 3.6, 1.8 Hz, 1H), 5.18 – 5.07 (m, 1H), 4.97 – 4.74 (m, 1H), 3.82 (s, 3H), 3.81 (s, 3H), 3.71 – 3.58 (m, 1H), 2.53 – 2.33 (m, 2H), 1.46 (s, 9H) (signal broadening due to rotamers); **^{13}C NMR** (101 MHz, $CDCl_3$): δ = 163.3 (q), 162.7 (q), 152.3 (q), 147.3 (q), 138.1 (q), 131.0 (+), 127.5 (+), 81.1 (q), 63.6 (–), 60.4 (+), 58.4 (+), 52.5 (+), 52.4 (+), 40.4 (+), 28.3 (+) (signal broadening due to rotamers); **IR** $\tilde{\nu}$ [cm^{-1}]: 3437, 2978, 1700, 1640, 1431, 1367, 1327, 1260, 1163, 1118, 1077, 1033, 943, 861, 757, 731; **HRMS** (ESI): calcd. for $C_{17}H_{23}NO_7$ $(M+H)^+$, m/z = 354.1547; found 354.1547.

Ethyl (3*aS*,4*S*,5*S*,8*R*,8*aR*)-1,3-dioxo-9-tosyl-3,3a,4,5,8,8a-hexahydro-1*H*-4,8-epiminocyclohepta[c]furan-5-carboxylate (7h)

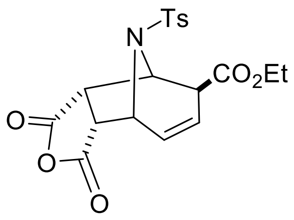

Following GP-1b-Conditions II, **7h** was prepared from cyclopropane **4e** (307 mg, 1.00 mmol, 1.0 equiv) and maleic anhydride (108 mg, 1.10 mmol, 1.1 equiv) in toluene (2 mL). The mixture was heated for 1 h at 150 °C under microwave irradiation. The crude mixture was filtered through a short plug of silica gel and the solvent was removed under reduced pressure to afford the desired cycloaddition product **7h** (320 mg, 789 µmol, 79%) as a colorless solid.

R*f* = 0.10 (PE:EA = 3:1, UV); **m.p.** = 155 °C; **^{1}H NMR** (300 MHz, $CDCl_3$): δ = 7.77 – 7.70 (m,

2H), 7.36 – 7.28 (m, 2H), 6.13 (ddd, *J* = 9.6, 5.8, 1.8 Hz, 1H), 5.94 (ddd, *J* = 9.6, 4.5, 1.4 Hz, 1H), 5.14 (dq, *J* = 8.4, 1.6 Hz, 1H), 4.82 (ddd, *J* = 7.2, 5.9, 1.6 Hz, 1H), 4.18 (dd, *J* = 10.0, 8.4 Hz, 1H), 4.10 – 3.90 (m, 2H), 3.83 (dq, *J* = 10.8, 7.1 Hz, 1H), 3.49 (dt, *J* = 4.5, 1.8 Hz, 1H), 2.44 (s, 3H), 1.13 (t, *J* = 7.1 Hz, 3H); **^{13}C NMR** (75 MHz, $CDCl_3$): δ = 169.6 (q), 168.8 (q), 167.5 (q), 145.0 (q), 134.9 (q), 129.9 (+), 128.5 (+), 128.1 (+), 126.3 (+), 61.7 (–), 58.7 (+), 56.02 (+), 55.99 (+), 50.4 (+), 45.1 (+), 21.7 (+), 13.9 (+); **IR ṽ** [cm^{-1}]: 2989, 1864, 1774, 1722, 1595, 1446, 1368, 1325, 1308, 1259, 1242, 1221, 1208, 1185, 1156, 1088, 1021, 1006, 987, 917, 902, 857, 813, 763, 719, 667; **HRMS** (ESI): calcd. for $C_{19}H_{19}NO_7S$ $(M+H)^+$, m/z = 406.0955; found 406.0955.

Ethyl (3a*S*,4*R*,5*S*,8*R*,8a*R*)-1,3-dioxo-2-phenyl-9-tosyl-1,2,3,3a,4,5,8,8a-octahydro-4,8-epiminocyclohepta[c]pyrrole-5-carboxylate (7i)

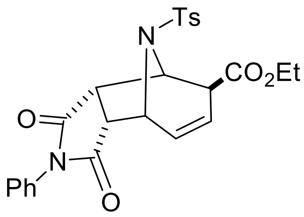

Following GP-1b-Conditions II, **7i** was prepared from cyclopropane **4e** (154 mg, 501 µmol, 1.0 equiv) and *N*-phenylmaleimide (95.4 mg, 551 µmol, 1.1 equiv) in toluene (0.5 mL). The mixture was heated for 30 min at 150 °C under microwave irradiation. The crude product was purified by flash column chromatography (33% EA:PE) to afford cycloadduct **7i** (154 mg, 320 µmol, 64%) as a colorless solid.

R_f = 0.29 (PE:EA = 2:1; UV); **m.p.** = 71 °C; **^{1}H NMR** (300 MHz, $CDCl_3$): δ = 7.83 – 7.74 (m, 2H), 7.52 – 7.36 (m, 3H), 7.36 – 7.29 (m, 2H), 7.19 – 7.09 (m, 2H), 6.13 (ddd, *J* = 9.7, 5.8, 1.8 Hz, 1H), 5.95 (ddd, *J* = 9.6, 4.4, 1.4 Hz, 1H), 5.19 (dq, *J* = 8.3, 1.5 Hz, 1H), 4.97 – 4.79 (m, 1H), 4.11 – 3.78 (m, 4H), 3.53 (dt, *J* = 4.3, 1.7 Hz, 1H), 2.44 (s, 3H), 1.15 (t, *J* = 7.1 Hz, 3H); **^{13}C NMR** (75 MHz, $CDCl_3$): δ = 174.7 (q), 173.2 (q), 169.3 (q), 144.6 (q), 135.6 (q), 131.3 (q), 129.8 (+), 129.3 (+), 129.1 (+), 128.6 (+), 128.0 (+), 126.3 (+), 125.7 (+), 61.6 (–), 58.3 (+), 56.0 (+), 54.4 (+), 49.5 (+), 45.1 (+), 21.7 (+), 14.0 (+); **IR ṽ** [cm^{-1}]: 2982, 1707, 1595, 1498, 1457, 1579, 1353, 1327, 1304, 1185, 1156, 1088, 1049, 1029, 932, 906, 862, 817, 735, 717, 691, 664; **HRMS** (ESI): calcd. for $C_{25}H_{24}N_2O_6S$ $(M+H)^+$, m/z = 481.1428; found 481.1432.

8-(*Tert*-butyl) 2-ethyl (1*R*,2*S*,5*R*,6*S*,7*S*)-6,7-dicyano-8-azabicyclo[3.2.1]oct-3-ene-2,8-dicarboxylate (*major* 7j)

8-(*Tert*-butyl) 2-ethyl (1*R*,2*S*,5*R*,6*R*,7*R*)-6,7-dicyano-8-azabicyclo[3.2.1]oct-3-ene-2,8-dicarboxylate (*minor* 7j)

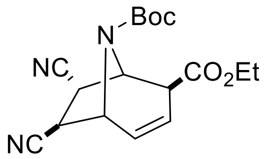

major **7j**

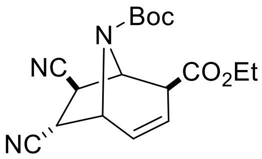

minor **7j**

Following GP-1b-Conditions II, **7j** was prepared from cyclopropane **4b** (102 mg, 403 µmol, 1.0 equiv) and fumaronitrile (85.0 mg, 1.09 mmol, 2.7 equiv) in toluene (0.3 mL). The mixture was heated for 1 h at 150 °C under microwave irradiation. The crude product (diastereomeric ratio of *dr* 4.5:1) was purified by flash system (12 → 19% EA:PE) to afford *major* **7j** (25.4 mg, 76.7 µmol, 19%) and inseparable mixture of *major* and *minor* **7j** (81.2 mg, 245 µmol, 61%, *dr* 3.2:1) both as a colorless oil.

R_f = 0.45 (PE:EA = 3:1; $KMnO_4$); **^{1}H NMR** (400 MHz, $CDCl_3$): δ (*major* **7j**) = 6.20 (ddd, *J* = 9.8, 5.5, 1.9 Hz, 1H), 5.96 (ddd, *J* = 9.6, 4.3, 1.5 Hz, 1H), 5.50 – 5.30 (m, 1H), 5.01 – 4.69 (m, 1H), 4.32 – 4.10 (m, 2H), 3.65 (dd, *J* = 7.8, 3.6 Hz, 1H), 3.46 (dt, *J* = 4.5, 1.5 Hz, 1H), 3.34 (d, *J* = 3.6 Hz, 1H), 1.46 (s, 9H), 1.29 (t, *J* = 7.2 Hz, 3H) (signal broadening due to rotamers); **^{13}C NMR** (101 MHz, $CDCl_3$): δ (*major* **7j**) = 169.3 (q), 152.2 (q), 129.8 (+), 125.1 (+), 118.0 (q), 117.2 (q), 82.6 (q), 62.0 (–), 57.1 (+), 55.9 (+), 45.1 (+), 41.4 (+), 37.1 (+), 28.1 (+), 14.2 (+) (signal broadening due to rotamers); *major* **7j**: **IR** ṽ [cm^{-1}]: 2982, 2937, 2248, 1707, 1476, 1394, 1372, 1334, 1260, 1163, 1118, 1085, 1036, 977, 910, 760, 723; **HRMS** (ESI): calcd. for $C_{17}H_{21}N_3O_4$ $(M+Na)^+$, m/z = 354.1424; found 354.1424 (*major* **7j** t_r = 2.424-2.474 min); **^{1}H NMR** (400 MHz, $CDCl_3$): δ (*minor* **7j**) = 6.26 (dddd, *J* = 9.8, 5.5, 2.1, 0.9 Hz, 1H), 5.98 – 5.91 (m, 1H)*, 5.28 – 5.24 (m, 1H), 4.96 – 4.75 (m, 1H)*, 4.25 – 4.13 (m, 2H)*, 3.29 (dd, *J* = 7.0, 5.6 Hz, 1H), 3.18 (dd, *J* = 7.0, 1.4 Hz, 1H), 3.08 (dt, *J* = 3.8, 1.6 Hz, 1H), 1.44 (s, 9H)*, 1.29 – 1.25 (m, 3H)* (signal broadening due to rotamers); **^{13}C NMR** (101 MHz, $CDCl_3$): δ (*minor* **7j**) = 168.8 (q), 152.4 (q), 129.0 (+), 125.0 (+), 118.2 (q), 116.2 (q), 82.5 (q), 61.9 (–), 59.7 (+), 54.4 (+), 48.8 (+), 42.0 (+), 38.3 (+), 28.0 (+)*, 14.1 (+)* (signal broadening due to rotamers; *these signals are overlapping with major diastereomer); **HRMS** (ESI): calcd. for $C_{17}H_{21}N_3O_4$ $(M+Na)^+$, m/z = 354.1424; found 354.1423 (*minor* **7j** t_r = 2.483-2.520 min).

Ethyl (1*R*,2*S*,5*R*,6*R*,7*S*)-6,7-dicyano-8-tosyl-8-azabicyclo[3.2.1]oct-3-ene-2-carboxylate (7k)

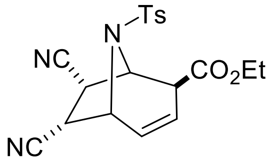

Following GP-1b-Conditions II, **7k** was prepared from cyclopropane **4e** (144 mg, 469 µmol, 1.0 equiv) and maleonitrile (98.8 mg, 1.26 mmol, 2.7 equiv) in toluene (0.3 mL). The mixture was heated for 30 min at 150 °C under microwave irradiation. The crude product was purified by flash system (5 → 21% EA:PE) to afford *endo* **7k** (110 mg, 285 µmol, 61%) as a colorless solid.

$\mathbf{R_f}$ = 0.52 (PE:EA = 3:2; UV, $KMNO_4$); **m.p.** = 174 °C; **^{1}H NMR** (400 MHz, $CDCl_3$): δ = 7.77 – 7.65 (m, 2H), 7.40 – 7.27 (m, 2H), 6.28 (ddd, *J* = 9.6, 5.9, 1.9 Hz, 1H), 6.05 (ddd, *J* = 9.6, 4.5, 1.4 Hz, 1H), 5.12 (dq, *J* = 7.5, 1.5 Hz, 1H), 4.73 (td, *J* = 5.7, 1.5 Hz, 1H), 3.97 (ddd, *J* = 13.6, 10.8, 7.3 Hz, 2H), 3.84 (dq, *J* = 10.8, 7.1 Hz, 1H), 3.75 (dd, *J* = 10.9, 5.5 Hz, 1H), 3.61 (dd, *J* = 4.2, 2.0 Hz, 1H), 2.44 (s, 3H), 1.13 (t, *J* = 7.1 Hz, 3H); **^{13}C NMR** (101 MHz, $CDCl_3$): δ = 168.6 (q), 145.3 (q), 134.7 (q), 129.9 (+), 128.7 (+), 128.0 (+), 125.5 (+), 115.9 (q), 115.0 (q), 61.8 (–), 58.8 (+), 56.3 (+), 46.5 (+), 40.7 (+), 35.3 (+), 21.7 (+), 13.9 (+); **IR** ṽ [cm^{-1}]: 2960, 2248, 1730, 1595, 1446, 1327, 1293, 1208, 1159, 1088, 1029, 980, 939, 898, 816, 760, 708, 664; **HRMS** (ESI): calcd. for $C_{19}H_{19}N_3O_4S$ $(M+Na)^+$, m/z = 408.0988; found 408.0987.

8-(*Tert*-butyl) 2-methyl (1*R*,2*S*,5*R*,6*R*,7*S*)-6,7-dicyano-8-azabicyclo[3.2.1]oct-3-ene-2,8-dicarboxylate (*endo* (–)-7l)

8-(*Tert*-butyl) 2-methyl (1*R*,2*S*,5*R*,6*S*,7*R*)-6,7-dicyano-8-azabicyclo[3.2.1]oct-3-ene-2,8-dicarboxylate (*exo* (–)-7l)

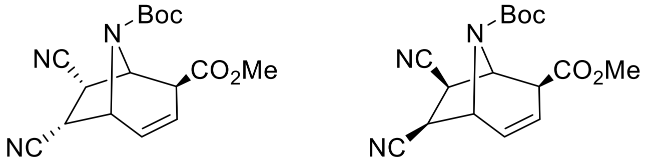

endo **(-)-7l** *exo* **(-)-7l**

Following GP-1b-Conditions II, **(–)-7l** was prepared from cyclopropane **(−)-4c** (150 mg, 627 µmol, 1.0 equiv) and maleonitrile (132 mg, 1.69 mmol, 2.7 equiv) in toluene (0.3 mL). The

mixture was heated for 15 min at 150 °C under microwave irradiation. The crude product (diastereomeric ratio of *dr* 1:1) was purified by flash system (4 → 20% EA:PE) to afford *endo* **(–)-7l** (74.8 mg, 236 µmol, 38%, 99% *ee*) as a colorless solid and *exo* **(–)-7l** (66.1 mg, 208 µmol, 33%, 98% *ee*) as a colorless oil.

endo* (–)-7l: R*f = 0.52 (PE:EA = 3:2; $KMnO_4$); **m.p.** = 153 °C; **HPLC analysis:** 99% *ee* (Chiralcel OJ-H, *n*-heptane/*i*-propanol 70:30, 0.5 mL/min, 215 nm): t_r = 31.61 min; $[\alpha]_D^{20}$ = -132.3 (c = 1.0 in $CHCl_3$); **^{1}H NMR** (400 MHz, $CDCl_3$): δ = 6.31 (ddd, *J* = 9.8, 5.4, 1.9 Hz, 1H), 6.07 (ddd, *J* = 9.8, 4.3, 1.5 Hz, 1H), 5.22 (dq, *J* = 7.6, 1.3 Hz, 1H), 4.79 (t, *J* = 5.6 Hz, 1H), 3.78 (dd, *J* = 10.9, 7.7 Hz, 1H), 3.75 (s, 3H), 3.55 (dt, *J* = 4.4, 1.5 Hz, 1H), 3.42 (dd, *J* = 10.8, 5.6 Hz, 1H), 1.42 (s, 9H); **^{13}C NMR** (101 MHz, $CDCl_3$): δ = 169.9 (q), 152.0 (q), 129.2 (+), 125.5 (+), 116.0 (q), 115.2 (q), 82.3 (q), 56.0 (+), 53.7 (+), 52.7 (+), 45.1 (+), 39.6 (+), 35.4 (+), 28.1 (+) (signal broadening due to rotamers); **IR $\tilde{\nu}$** [cm^{-1}]: 2978, 2251, 1737, 1700, 1394, 1342, 1260, 1163, 1118, 731; **HRMS** (ESI): calcd. for $C_{16}H_{19}N_3O_4$ $(M+Na)^+$, m/z = 340.1268; found 340.1271.

exo* (–)-7l*:* R*f = 0.29 (PE:EA = 3:2; $KMnO_4$); **HPLC analysis:** 98% *ee* (Phenomenex Lux Cellulose-1, *n*-heptane/*i*-propanol 70:30, 0.5 mL/min, 215 nm): t_r (*major*) = 42.56 min, t_r (*minor*) = 24.21 min; $[\alpha]_D^{20}$ = -60.0 (c = 1.0 in $CHCl_3$); **^{1}H NMR** (300 MHz, $CDCl_3$): δ = 6.23 – 6.08 (m, 1H), 5.82 (dd, *J* = 9.6, 4.1 Hz, 1H), 5.42 – 5.23 (m, 1H), 5.16 – 4.77 (m, 1H), 3.74 (s, 3H), 3.54 (d, *J* = 8.5 Hz, 1H), 3.36 (d, *J* = 8.3 Hz, 1H), 3.12 – 3.01 (m, 1H), 1.47 (s, 9H) (signal doubling and broadening due to rotamers); **^{13}C NMR** (101 MHz, $CDCl_3$): δ = 169.3 (q), 152.1 (q), 129.9 (+), 124.2 (+), 123.9 (+), 117.2 (q), 116.6 (q), 82.3 (q), 59.4 (+), 58.6 (+), 57.4 (+), 56.4 (+), 52.7 (+), 48.6 (+), 48.3 (+), 41.4 (+), 38.4 (+), 37.5 (+), 28.0 (+) (signal doubling and broadening due to rotamers); **IR $\tilde{\nu}$** [cm^{-1}]: 2960, 2930, 2244, 1733, 1670, 1394, 1338, 1238, 1156, 1118, 1066; **HRMS** (ESI): calcd. for $C_{16}H_{19}N_3O_4$ $(M+Na)^+$, m/z = 340.1268; found 340.1268.

8-(*Tert*-butyl) 2-methyl (1*R*,2*S*,5*R*,6*R*,7*S*)-6,7-dicyano-8-azabicyclo[3.2.1]oct-3-ene-2,8-dicarboxylate (*endo* 7l)

8-(*Tert*-butyl) 2-methyl (1*R*,2*S*,5*R*,6*S*,7*R*)-6,7-dicyano-8-azabicyclo[3.2.1]oct-3-ene-2,8-dicarboxylate (*exo* 7l)

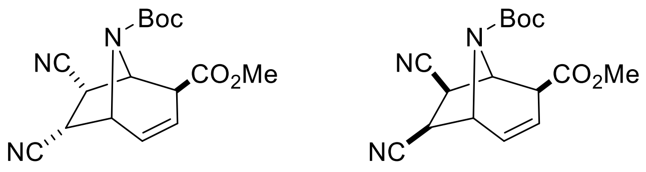

endo **7l** *exo* **7l**

Following GP-1b-Conditions II, **7l** was prepared from cyclopropane **4c** (158 mg, 660 µmol, 1.0 equiv) and maleonitrile (139 mg, 1.78 mmol, 2.7 equiv) in toluene (0.3 mL). The mixture was heated for 15 min at 150 °C under microwave irradiation. The crude product (diastereomeric ratio of *dr* 1:1) was purified by flash system (4 → 20% EA:PE) to afford *endo* **7l** (76.4 mg, 241 µmol, 36%) as a colorless solid and *exo* **7l** (70.0 mg, 221 µmol, 33%) as a colorless oil.

NMR, m.p. and IR data were identical with those reported for the enantiomers **(–)-7l**.

Ethyl (1*S*,2*S*,5*R*)-7,8-ditosyl-8-azabicyclo[3.2.1]octa-3,6-diene-2-carboxylate (*major* 7m)

Ethyl (1*S*,2*S*,5*R*)-6,8-ditosyl-8-azabicyclo[3.2.1]octa-3,6-diene-2-carboxylate (*minor* 7m)

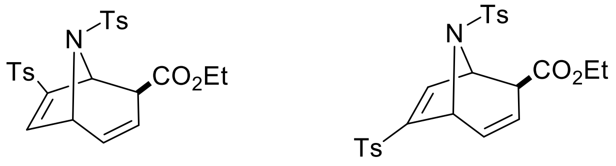

major **7m** *minor* **7m**

Following GP-1b-Conditions II, **7m** was prepared from cyclopropane **4e** (252 mg, 818 µmol, 1.0 equiv) and tosylacetylene (399 mg, 2.21 mmol, 2.7 equiv) in toluene (0.3 mL). The mixture was heated for 30 min at 150 °C under microwave irradiation. The crude product (diastereomeric ratio of *dr* 4.0:1) was purified by flash system (17 → 25% EA:PE) to afford *major* **7m** (30.0 mg, 61.5 µmol, 8%) as a colorless solid and mixture of *major* and *minor* **7m** (62.3 mg, 128 µmol, 16%, *dr* 1.7:1) as a colorless oil.

R_f = 0.46 (PE:EA = 2:1; $KMnO_4$); **m.p.** = 49 °C; **^{1}H NMR** (400 MHz, $CDCl_3$): δ (*major* **7m**) = 7.64 (d, *J* = 8.3 Hz, 2H), 7.44 (d, *J* = 8.3 Hz, 2H), 7.37 (d, *J* = 8.1 Hz, 2H), 7.15 (d, *J* = 8.1 Hz, 2H), 6.89 (d, *J* = 2.3 Hz, 1H), 6.23 (ddd, *J* = 9.5, 5.7, 2.1 Hz, 1H), 5.71 (ddd, *J* = 9.5, 4.0, 1.6 Hz, 1H), 5.35 (bs, 1H), 4.73 (dd, *J* = 5.8, 2.3 Hz, 1H), 4.17 (q, *J* = 7.1 Hz, 2H), 3.27 (ddd, *J* = 3.5, 2.1, 1.0 Hz, 1H), 2.49 (s, 3H), 2.41 (s, 3H), 1.27 (t, *J* = 7.2 Hz, 3H); **^{13}C NMR** (101 MHz, $CDCl_3$): δ (*major* **7m**) = 168.8 (q), 148.0 (+), 145.3 (q), 143.9 (q), 142.3 (q), 135.8 (q), 134.7 (q), 130.04 (+), 129.8 (+), 128.9 (+), 128.2 (+), 127.8 (+), 125.6 (+), 62.2 (+), 61.8 (–), 60.0 (+), 44.7 (+), 21.8 (+), 21.6 (+), 14.1(+); *major* **7m**: **IR** $\tilde{\nu}$ [cm^{-1}]: 3060, 2982, 2926, 1737, 1595, 1450, 1353, 1305, 1230, 1152, 1096, 1040, 969, 731, 645; **HRMS** (ESI): calcd. for $C_{24}H_{25}NO_6S_2$ $(M+Na)^+$, m/z = 510.1016; found 510.1015 (*major* **7m** t_r = 2.926-2.972 min);**^{1}H NMR** (400 MHz, $CDCl_3$): δ (*minor* **7m**) = 7.53 – 7.49 (m, 2H), 7.35 – 7.31 (m, 4H)*, 7.18 (d, *J* = 8.1 Hz, 2H), 6.25 (d, *J* = 2.5 Hz, 1H), 6.14 (ddd, *J* = 9.5, 5.7, 2.1 Hz, 1H), 5.54 (ddd, *J* = 9.5, 3.9, 1.5 Hz, 1H), 5.20 (d, *J* = 1.1 Hz, 1H), 4.81 (d, *J* = 5.7 Hz, 1H), 4.21 – 4.11 (m, 2H)*, 2.88 (ddd, *J* = 3.5, 2.1, 0.9 Hz, 1H), 2.46 (s, 3H), 2.40 (s, 3H)*, 1.29 – 1.22 (m, 3H)*; **^{13}C NMR** (101 MHz, $CDCl_3$): δ (*minor* **7m**) = 169.0 (q), 155.5 (q), 145.4 (q), 144.0 (q), 135.6 (q), 134.9 (+), 134.4 (q), 131.7 (+), 130.02 (+), 129.9 (+), 128.3 (+), 127.9 (+), 122.5 (+), 62.7 (+), 62.0 (–), 58.9 (+), 42.9 (+), 21.8 (+)*, 21.6 (+)*, 14.1 (+)* (*these signals are overlapping with *major* diastereomer); **HRMS** (ESI): calcd. for $C_{24}H_{25}NO_6S_2$ $(M+Na)^+$, m/z = 510.1016; found 510.1015 (*major* and *minor* **7m** t_r = 2.926-2.972 min).

4-Ethyl 1,6,7-trimethyl (1*R*,4*S*,5*S*)-8-oxabicyclo[3.2.1]octa-2,6-diene-1,4,6,7-tetracarboxylate ((+)-8a)

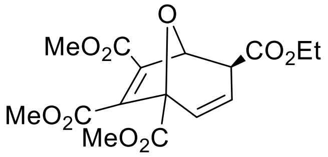

Following GP-1a-Conditions I, **(+)-8a** was prepared from cyclopropane **(−)-5a** (1.67 g, 7.87 mmol, 1.0 equiv) and DMAD (3.02 g, 2.60 mL, 21.3 mmol, 2.7 equiv). The mixture was heated for 30 min at 170 °C under microwave irradiation. The crude product was purified by flash system (6 → 37% EA:PE) to afford cycloadduct **(+)-8a** (1.98 g, 5.59 mmol, 71%, 99% *ee*) as a colorless solid.

R_f = 0.21 (PE:EA = 3:1; $KMnO_4$); **m.p.** = 64 °C; **HPLC analysis:** 99% *ee* (Chiralcel AS-H, *n*-heptane/*i*-propanol 90:10, 1.0 mL/min, 215 nm): t_r (*major*) = 18.96 min, t_r (*minor*) = 24.37 min; **$[\alpha]_D^{20}$** = +128.7 (c = 1.0 in $CHCl_3$); **^{1}H NMR** (300 MHz, $CDCl_3$): δ = 6.59 (dd, *J* = 9.8, 2.3 Hz, 1H), 5.89 (ddd, *J* = 9.8, 4.2, 1.9 Hz, 1H), 5.76 (d, *J* = 1.8 Hz, 1H), 4.24 (q, *J* = 7.2 Hz, 2H), 3.83

(s, 3H), 3.82 (s, 3H), 3.80 (s, 3H), 3.02 (dd, *J* = 4.0, 2.1 Hz, 1H), 1.29 (t, *J* = 7.2 Hz, 3H). **^{13}C NMR** (75 MHz, $CDCl_3$): δ = 169.5 (q), 166.7 (q), 162.2 (q), 162.0 (q), 150.2 (q), 134.7 (q), 130.7 (+), 124.1 (+), 86.1 (q), 80.7 (+), 61.9 (–), 53.2 (+), 52.9 (+), 52.7 (+), 40.6 (+), 14.2 (+); **IR** $\tilde{\nu}$ [cm^{-1}]: 2989, 2960, 1722, 1655, 1439, 1368, 1260, 1193, 1144, 1029, 850, 727, 667; **HRMS** (ESI): calcd. for $C_{16}H_{18}O_9$ $(M+H)^+$, m/z = 355.1024; found 355.1032.

4-Ethyl 1,6,7-trimethyl (1*R*,4*S*,5*S*)-8-oxabicyclo[3.2.1]octa-2,6-diene-1,4,6,7-tetracarboxylate (8a)

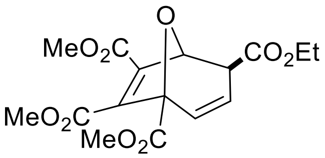

Following GP-1a-Conditions I, **8a** was prepared from cyclopropane **5a** (215 mg, 1.01 mmol, 1.0 equiv) and DMAD (389 mg, 335 µL, 2.74 mmol, 2.7 equiv). The mixture was heated for 30 min at 170 °C under microwave irradiation. The crude product was purified by flash system (6 → 37% EA:PE) to afford cycloadduct **8a** (250 mg, 706 µmol, 70%) as a colorless solid.

NMR, m.p. and IR data were identical with those reported for the enantiomer **(+)-8a**.

4-(*Tert*-butyl) 1,6,7-trimethyl (1*R*,4*S*,5*S*)-8-oxabicyclo[3.2.1]octa-2,6-diene-1,4,6,7-tetracarboxylate (8b)

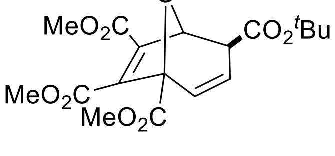

Following GP-1a-Conditions I, **8b** was prepared from cyclopropane **5b** (607 mg, 2.53 mmol, 1.0 equiv) and DMAD (969 mg, 836 µL, 6.82 mmol, 2.7 equiv). The mixture was heated for 30 min at 170 °C under microwave irradiation. The crude product was purified by flash system (6 → 37% EA:PE) to afford cycloadduct **8b** (726 mg, 1.90 mmol, 75%) as a colorless oil.

R$_f$ = 0.62 (PE:EA = 2:1; $KMnO_4$); **^{1}H NMR** (300 MHz, $CDCl_3$): δ = 6.57 (dd, *J* = 9.8, 2.3 Hz, 1H), 5.84 (ddd, *J* = 9.8, 4.1, 1.9 Hz, 1H), 5.70 (d, *J* = 1.8 Hz, 1H), 3.81 (s, 6H), 3.79 (s, 3H), 2.93 (ddd, *J* = 4.1, 2.3, 0.7 Hz, 1H), 1.47 (s, 9H); **^{13}C NMR** (75 MHz, $CDCl_3$): δ = 168.4 (q), 166.8 (q), 162.11 (q), 162.08 (q), 149.6 (q), 135.2 (q), 130.3 (+), 124.5 (+), 85.9 (q), 82.4 (q), 80.7 (+), 53.1 (+), 52.7 (+), 52.6 (+), 41.3 (+), 28.0 (+); **IR** ṽ [cm^{-1}]: 2960, 1722, 1651, 1439, 1245, 1200, 1148, 1077, 1006, 731; **HRMS** (ESI): calcd. for $C_{18}H_{22}O_9$ $(M+H)^+$, m/z = 383.1337; found 383.1336.

7-(*Tert*-butyl) 4-methyl (3a*S*,4*R*,7*S*,8*R*,8a*R*)-1,3-dioxo-2-phenyl-2,3,3a,7,8,8a-hexahydro-4,8-epoxycyclohepta[c]pyrrole-4,7(1*H*)-dicarboxylate (*major* 8c)

7-(*Tert*-butyl) 4-methyl (3*aR*,4*R*,7*S*,8*R*,8*aS*)-1,3-dioxo-2-phenyl-2,3,3a,7,8,8a-hexahydro-4,8-epoxycyclohepta[c]pyrrole-4,7(1*H*)-dicarboxylate (*minor* 8c)

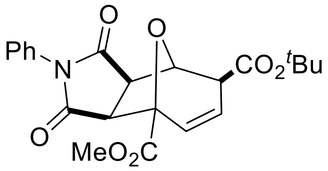

major **8c**

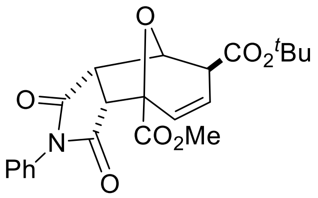

minor **8c**

Following GP-1b-Conditions II, **8c** was prepared from cyclopropane **5b** (100 mg, 416 µmol, 1.0 equiv) and *N*-phenylmaleimide (195 mg, 1.12 mmol, 2.7 equiv) in toluene (1 mL). The mixture was heated for 1.5 h at 150 °C under microwave irradiation. The crude product (diastereomeric ratio of *dr* 2.9:1) was purified by flash column chromatography (33% EA:PE) to afford inseparable mixture of *major* and *minor* **8c** (124 mg, 299 µmol, 72%, *dr* 2.8:1) as a colorless solid. Recrystallization from toluene led to separation of pure *major* **8c** (47 mg, 114 µmol, 27%) as a colorless solid.

R$_f$ = 0.23 (PE:EA = 1:1; $KMnO_4$); **m.p.** = 144 °C; **^{1}H NMR** (400 MHz, $CDCl_3$): δ (*major* **8c**) = 7.51 – 7.36 (m, 3H), 7.29 – 7.27 (m, 2H, overlapping with solvent signal), 6.55 (dd, *J* = 9.9, 2.1 Hz, 1H), 5.98 (ddd, *J* = 9.9, 4.5, 1.6 Hz, 1H), 5.49 (bs, 1H), 3.86 (s, 3H), 3.79 (d, *J* = 7.5 Hz, 1H), 3.36 (dd, *J* = 7.5, 1.0 Hz, 1H), 2.93 (ddd, *J* = 4.5, 2.1, 0.7 Hz, 1H), 1.48 (s, 9H); **^{13}C NMR** (101 MHz, $CDCl_3$): δ (*major* **8c**) = 175.3 (q), 172.8 (q), 168.2 (q), 167.4 (q), 131.41 (+), 131.36 (q), 129.2 (+), 129.0 (+), 126.4 (+), 123.8 (+), 83.0 (q), 82.5 (q), 79.5 (+), 57.5 (+), 53.1 (+), 50.3 (+), 47.1 (+), 28.1 (+); *major* **8c**: **IR** ṽ [cm^{-1}]: 2974, 1711, 1372, 1204, 1156, 1096, 1070, 846, 731; **HRMS** (ESI): calcd. for $C_{22}H_{23}NO_7$ $(M+H)^+$, m/z = 414.1547; found 414.1548

(*major* **8c** t_r = 2.636-2.644 min); **^{1}H NMR** (400 MHz, $CDCl_3$): δ (*minor* **8c**) = 7.44 – 7.36 (m, 3H)*, 7.22 – 7.17 (m, 2H), 6.41 (dd, *J* = 10.0, 2.1 Hz, 1H), 6.11 (ddd, *J* = 10.0, 4.6, 1.7 Hz, 1H), 5.48 (dd, *J* = 9.1, 1.4 Hz, 1H)*, 4.11 (dd, *J* = 9.6, 8.6 Hz, 1H), 3.96 (d, *J* = 9.5 Hz, 1H), 3.91 (s, 3H), 3.27 (dd, *J* = 4.7, 2.1 Hz, 1H), 1.46 (s, 9H); **^{13}C NMR** (101 MHz, $CDCl_3$): δ (*minor* **8c**) = 174.4 (q), 171.5 (q), 168.5 (q), 168.0 (q), 131.36 (q)*, 129.3 (+), 129.1 (+), 127.9 (+), 126.32 (+), 126.30 (+), 82.3 (q), 80.7 (q), 76.7 (+), 57.8 (+), 53.4 (+), 49.7 (+), 43.2 (+), 28.0 (+)* (*these signals are overlapping with major diastereomer); **HRMS** (ESI): calcd. for $C_{22}H_{23}NO_7$ $(M+H)^+$, m/z = 414.1547; found 414.1552 (*minor* **8c** t_r = 2.686-2.732 min).

4-Ethyl 1-methyl (1*R*,4*S*,5*R*,6*R*,7*R*)-6,7-dicyano-8-oxabicyclo[3.2.1]oct-2-ene-1,4-dicarboxylate (*major* (–)-8d)

4-Ethyl 1-methyl (1*R*,4*S*,5*R*,6*S*,7*S*)-6,7-dicyano-8-oxabicyclo[3.2.1]oct-2-ene-1,4-dicarboxylate (*minor* 8d)

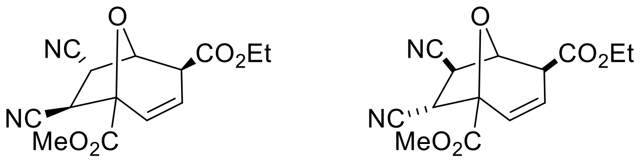

major (-)-**8d** *minor* **8d**

Following GP-1b-Conditions II, **8d** was prepared from cyclopropane **(−)-5a** (566 mg, 2.67 mmol, 1.0 equiv) and fumaronitrile (562 mg, 7.20 mmol, 2.7 equiv) in toluene (0.5 mL). The mixture was heated for 15 min at 170 °C under microwave irradiation. The crude mixture (diastereomeric ratio of *dr* 3.7:1) was recrystallized from toluene to obtain pure *major* **(–)-8d** (390 mg, 1.34 mmol, 50%, 99% *ee*) as a colorless solid. The filtrate was concentrated under reduced pressure. The residue was purified by flash system (17 → 20% EA:PE) to afford inseparable mixture of *major* and *minor* **8d** (181 mg, 624 µmol, 23%, *dr* 1:1) as a colorless solid.

R_f = 0.47 (PE:EA = 2:1, $KMnO_4$); **m.p.** = 165 °C; **HPLC analysis:** 99% *ee* (Chiralpak AS-H, *n*-heptane/*i*-propanol 50:50, 0.5 mL/min, 215 nm): t_r = 25.61 min; **$[\alpha]_D^{20}$** = -31.4 (c = 1.0 in $CHCl_3$); **^{1}H NMR** (300 MHz, $CDCl_3$): δ (*major* **(–)-8d**) = 6.33 (dd, *J* = 9.8, 2.0 Hz, 1H), 6.19 (ddd, *J* = 9.8, 4.7, 1.6 Hz, 1H), 5.57 (d, *J* = 7.9 Hz, 1H), 4.23 (q, *J* = 7.1 Hz, 2H), 3.93 (s, 3H), 3.87 (dd, *J* = 8.0, 3.5 Hz, 1H), 3.75 (d, *J* = 3.5 Hz, 1H), 3.35 (dd, *J* = 4.7, 1.8 Hz, 1H), 1.29 (t, *J* = 7.1 Hz, 3H); **^{13}C NMR** (75 MHz, $CDCl_3$): δ (*major* **(–)-8d**) = 168.5 (q), 166.2 (q), 128.9 (+), 125.5 (+), 116.4 (q), 115.9 (q), 84.0 (q), 76.3 (+), 62.16 (–), 53.8 (+), 45.0 (+), 43.3 (+), 36.6

(+), 14.1 (+); *major* **(–)-8d**: **IR** ṽ [cm^{-1}]: 3086, 2993, 2251, 1759, 1722, 1439, 1327, 1264, 1193, 1111, 1085, 1028, 839, 887, 731; **HRMS** (ESI): calcd. for $C_{14}H_{14}N_2O_5$ $(M+Na)^+$, m/z = 313.0795; found 313.0798 (*major* **(–)-8d** t_r = 1.866-1.870 min); **^{1}H NMR** (400 MHz, $CDCl_3$): δ (*minor* **8d**) = 6.42 (dd, *J* = 9.9, 2.0 Hz, 1H), 6.23 – 6.14 (m, 1H)*, 5.48 – 5.42 (m, 1H), 4.31 – 4.18 (m, 2H)*, 3.91 (s, 3H), 3.62 (d, *J* = 7.4 Hz, 1H), 3.38 – 3.31 (m, 1H)*, 3.01 (dd, *J* = 4.8, 2.0 Hz, 1H), 1.32 – 1.26 (m, 3H)*; **^{13}C NMR** (101 MHz, $CDCl_3$): δ (*minor* **8d**) = 168.0 (q), 166.1 (q), 127.9 (+), 125.1 (+), 117.1 (q), 114.8 (q), 81.9 (q), 79.5 (+), 62.22 (–), 53.9 (+), 46.9 (+), 45.9 (+), 38.1 (+), 14.1 (+)* (*these signals are overlapping with major diastereomer); **HRMS** (ESI): calcd. for $C_{14}H_{14}N_2O_5$ $(M+Na)^+$, m/z = 313.0795; found 313.0796 (*minor* **8d** t_r = 1.913-2.005 min).

4-Ethyl 1-methyl (1*R*,4*S*,5*R*,6*R*,7*R*)-6,7-dicyano-8-oxabicyclo[3.2.1]oct-2-ene-1,4-dicarboxylate (*majo*r 8d)

4-Ethyl 1-methyl (1*R*,4*S*,5*R*,6*S*,7*S*)-6,7-dicyano-8-oxabicyclo[3.2.1]oct-2-ene-1,4-dicarboxylate (*minor* 8d)

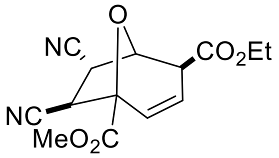

major **8d**

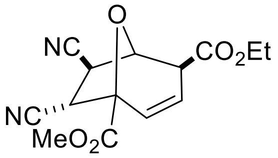

minor **8d**

Following GP-1b-Conditions II, **8d** was prepared from cyclopropane **5a** (268 mg, 1.26 mmol, 1.0 equiv) and fumaronitrile (266 mg, 3.41 mmol, 2.7 equiv) in toluene (0.25 mL). The mixture was heated for 15 min at 170 °C under microwave irradiation. The crude mixture (diastereomeric ratio of *dr* 3.6:1) was recrystallized from toluene to obtain pure *major* **8d** (179 mg, 1.26 mmol, 49%) as a colorless solid. The filtrate was concentrated under reduced pressure. The residue was purified by flash system (17 → 20% EA:PE) to afford inseparable mixture of *major* and *minor* **8d** (80.7 mg, 278 µmol, 22%, *dr* 1:1) as a colorless solid.

NMR, m.p. and IR data were identical with those reported for the enantiomer **(–)-8d**.

4-Ethyl 1-methyl (1*R*,4*S*,5*S*)-6-tosyl-8-oxabicyclo[3.2.1]octa-2,6-diene-1,4-dicarboxylate (8e)

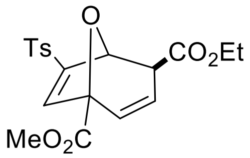

Following GP-1b-Conditions II, **8e** was prepared from cyclopropane **5a** (109 mg, 514 µmol, 1.0 equiv) and tosylacetylene (250 mg, 1.39 mmol, 2.7 equiv) in toluene (0.2 mL). The mixture was heated for 30 min at 160 °C under microwave irradiation. The crude product was purified by flash system (8 → 35% EA:PE) to afford cycloadduct **8e** (92.0 mg, 234 µmol, 46%) as a colorless oil.

R*f* = 0.50 (PE:EA = 3:2; $KMnO_4$); **^{1}H NMR** (300 MHz, $CDCl_3$): δ = 7.78 (d, *J* = 8.3 Hz, 2H), 7.36 (d, *J* = 8.5 Hz, 2H), 7.30 (s, 1H), 6.37 (dd, *J* = 9.7, 2.2 Hz, 1H), 5.89 (dddd, *J* = 9.7, 4.1, 2.0, 0.8 Hz, 1H), 5.54 (d, *J* = 1.8 Hz, 1H), 4.29 – 4.14 (m, 2H), 3.81 (s, 3H), 3.22 (dd, *J* = 3.9, 2.4 Hz, 1H), 2.45 (s, 3H), 1.28 (t, *J* = 7.1 Hz, 3H); **^{13}C NMR** (75 MHz, $CDCl_3$): δ = 169.3 (q), 167.4 (q), 147.2 (+), 145.7 (q), 144.0 (q), 135.6 (q), 130.3 (+), 130.1 (+), 128.1 (+), 125.1 (+), 85.5 (q), 79.5 (+), 61.9 (–), 53.2 (+), 41.5 (+), 21.7 (+), 14.1 (+); **IR** $\tilde{\nu}$ [cm^{-1}]: 3086, 2982, 2926, 1733, 1595, 1439, 1402, 1368, 1305, 1215, 1148, 1118, 1025, 969, 943, 880, 816, 671; **HRMS** (ESI): calcd. for $C_{19}H_{20}O_7S$ $(M+H)^+$, m/z = 393.1003; found 393.1003.

Screening of various dipolarophiles with different electronic distributions

Table 1. Screening of cycloaddition with various dipolarophiles and monocyclopropanated furan **5a**.

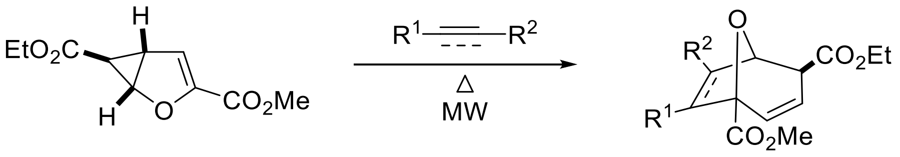

5a → **8**

Entry	R^1—R^2	T [°C]	Time [h]	Product
1	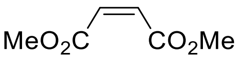	170	1	partial conversion
2	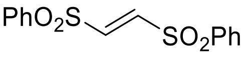	150	0.5	-
3	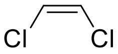	150 → 170	2.5	complex mixture
4	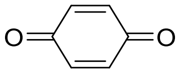	150	1.5	complex mixture
5	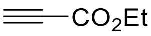	170	1	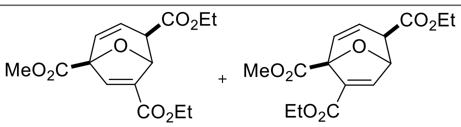 12% 4%
6	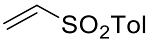	150	3	no conversion
7	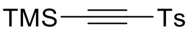	150 → 170	2	complex mixture
8	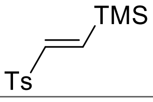	170	0.5	complex mixture
9	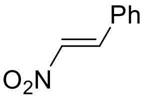	150 → 170	1 → 1	decomposition
10	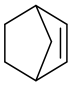	150 → 160	1 → 2	no conversion
11	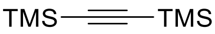	170	1.5	no conversion
12	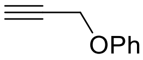	150	3	no conversion
13	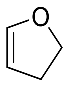	170	1	decomposition

Table 2. Screening of cycloaddition with various dipolarophiles and monocyclopropanated pyrrole **4e**.

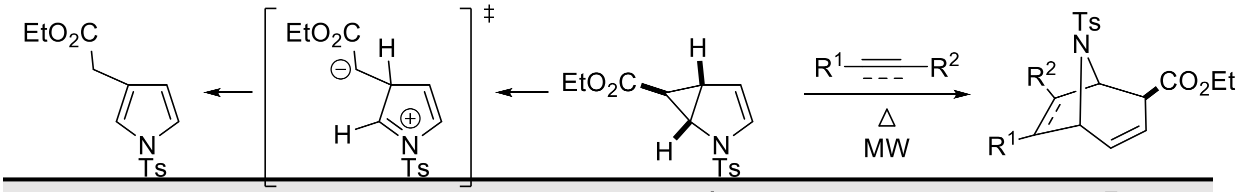

4e **7**

Entry	R^1—R^2	T [°C]	Time [h]	Product
1	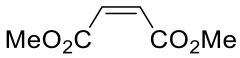	180	0.25	rearomatization
2	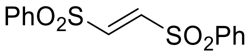	200	1	rearomatization
3	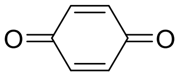	135	0.25	-
4	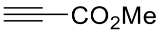	150	1	rearomatization
5	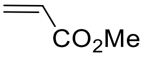	100 → 130	0.25 → 1	no conversion
6	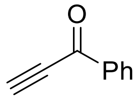	150	1	-
7	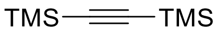	180	1	-
8	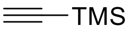	150	1	rearomatization
9	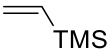	150	0.5	rearomatization
10	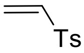	150 → 200	1	rearomatization

2.3. Derivatization reactions

5-Ethyl 1,7,8-trimethyl (1*S*,2*R*,4*R*,5*R*,6*S*)-3,9-dioxatricyclo[4.2.1.0^{2,4}]non-7-ene-1,5,7,8-tetracarboxylate (10)

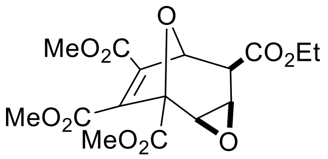

Cycloadduct **8a** (113 mg, 318 µmol, 1.0 equiv) was dissolved in CH_2Cl_2 (4 mL) and *m*-CPBA (193 mg, 1.12 mmol, 3.5 equiv) was added at 25 °C. The reaction mixture was stirred for 18 h at 50 °C. The reaction was quenched with saturated aqueous sodium thiosulfate and the aqueous layer was extracted with CH_2Cl_2 (3 x 5 mL). The combined organic phases were washed with saturated $NaHCO_3$ (2 x 20 mL), brine (2 x 20 mL) and dried over Na_2SO_4. The solvent was removed under reduced pressure and the crude product (117 mg, 316 µmol, 99%) was obtained as a yellowish oil. Recrystallization from diethyl ether afforded pure product **10** (105 mg, 283 µmol, 89%) as a colorless solid.

R*f* = 0.32 (PE:EA = 3:2; UV, $KMnO_4$); **m.p.** = 91 °C; **^{1}H NMR** (300 MHz, $CDCl_3$): δ = 5.59 (s, 1H), 4.40 – 4.21 (m, 2H), 3.86 (s, 3H), 3.83 (s, 3H), 3.81 (s, 3H), 3.78 (d, *J* = 3.9 Hz, 1H), 3.53 (ddt, *J* = 5.3, 3.9, 1.2 Hz, 1H), 2.79 (d, *J* = 5.2 Hz, 1H), 1.32 (t, *J* = 7.1 Hz, 3H); **^{13}C NMR** (75 MHz, $CDCl_3$): δ = 168.7 (q), 166.2 (q), 162.1 (q), 161.6 (q), 143.8 (q), 138.9 (q), 86.1 (q), 80.2 (+), 62.0 (–), 53.4 (+), 53.1 (+), 52.9 (+), 50.3 (+), 47.6 (+), 38.2 (+), 14.2 (+); **IR ṽ** [cm^{-1}]: 2960, 1722, 1655, 1435, 1297, 1260, 1193, 1141, 1081, 1029, 980, 910, 842, 790, 712; **HRMS** (ESI): calcd. for $C_{16}H_{18}O_{10}$ $(M+H)^+$, m/z = 371.0973; found 371.0973.

4-Ethyl 1,6,7-trimethyl (1*S*,2*R*,5*S*)-2-hydroxy-8-oxabicyclo[3.2.1]octa-3,6-diene-1,4,6,7-tetracarboxylate (11)

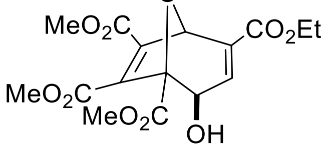

Flash column chromatography (33 → 50% EA:PE + 1% TEA) of epoxide **10** (87.0 mg, 246 µmol, 1.0 equiv) resulted in allylic alcohol **11** (58.0 mg, 157 µmol, 64%) as a colorless oil.

R$_f$ = 0.28 (PE:EA = 1:1; UV, $KMnO_4$); **^{1}H NMR** (400 MHz, $CDCl_3$): δ = 6.70 (dd, *J* = 3.7, 0.8 Hz, 1H), 5.70 (d, *J* = 0.8 Hz, 1H), 4.60 – 4.44 (m, 1H), 4.37 – 4.16 (m, 2H), 3.87 (s, 3H), 3.85 (s, 3H), 3.76 (s, 3H), 2.73 (s, 1H), 1.31 (t, *J* = 7.1 Hz, 3H); **^{13}C NMR** (101 MHz, $CDCl_3$): δ = 166.4 (q), 163.12 (q), 163.05 (q), 160.8 (q), 147.1 (q), 139.6 (q), 137.8 (q), 135.6 (+), 92.1 (q), 77.9 (+), 63.5 (+), 61.4 (–), 53.2 (+), 53.0 (+), 52.7 (+), 14.2 (+); **IR** $\tilde{\nu}$ [cm^{-1}]: 3478, 2960, 1726, 1651, 1439, 1275, 1162, 1059, 1021; **HRMS** (ESI): calcd. for $C_{16}H_{18}O_{10}$ $(M+H)^+$, m/z = 371.0973; found 371.0973.

4-(*Tert*-butyl) 1,6,7-trimethyl (1*R*,5*S*)-8-oxabicyclo[3.2.1]octa-3,6-diene-1,4,6,7-tetracarboxylate (12)

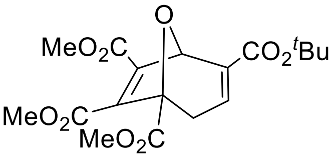

8-oxatropane **8b** (104 mg, 273 µmol, 1.0 equiv) was dissolved in CH_2Cl_2 (0.4 mL). Then TEA (36.5 mg, 50 µL, 361 µmol, 1.3 equiv) was added and the reaction mixture was stirred for 30 min at 25 °C. The solvent was removed under reduced pressure and product **12** (104 mg, 273 µmol, quant.) was obtained as a yellowish oil.

R$_f$ = 0.32 (PE:EA = 3:1; UV, $KMnO_4$); **^{1}H NMR** (400 MHz, $CDCl_3$) δ = 6.70 (ddd, *J* = 4.0, 3.3, 0.8 Hz, 1H), 5.51 (t, *J* = 1.0 Hz, 1H), 3.79 (s, 3H), 3.77 (s, 3H), 3.74 (s, 3H), 3.04 (ddd, *J* = 20.0, 3.3, 1.4 Hz, 1H), 2.50 (dd, *J* = 20.1, 4.0 Hz, 1H), 1.44 (s, 9H); **^{13}C NMR** (101 MHz, $CDCl_3$) δ = 168.1 (q), 162.6 (q), 162.3 (q), 162.0 (q), 147.9 (q), 135.9 (+), 135.6 (q), 134.4 (q), 86.6 (q), 81.4 (q), 78.3 (+), 53.2 (+), 52.62 (+), 52.59 (+), 28.6 (–), 28.0 (+); **IR** $\tilde{\nu}$ [cm^{-1}]: 2982, 2956, 1722, 1640, 1439, 1368, 1252, 1152, 1092, 1010, 943, 876, 846, 753; **HRMS** (ESI): calcd. for $C_{18}H_{22}O_9$ $(M+NH_4)^+$, m/z = 400.1602; found 400.1600.

8-(*Tert*-butyl) 2-ethyl 6,7-dimethyl (1*S*,5*R*)-8-azabicyclo[3.2.1]octa-2,6-diene-2,6,7,8-tetracarboxylate (13)

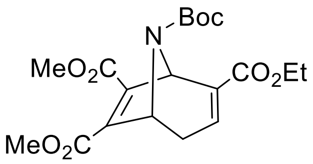

8-azatropane **7c** (283 mg, 716 µmol, 1.0 equiv) was dissolved in CH_2Cl_2 (2 mL). Then TEA (94.2 mg, 130 µL, 930 µmol, 1.3 equiv) was added and the reaction mixture was stirred for 2 h at 25 °C. The solvent was removed under reduced pressure and product **13** (280 mg, 709 µmol, 99%) was obtained as a yellowish oil.

R*f* = 0.62 (PE:EA = 2:1; $KMnO_4$); **^{1}H NMR** (400 MHz, $CDCl_3$): δ = 6.70 – 6.61 (m, 1H), 5.40 – 5.25 (m, 1H), 4.93 (d, *J* = 5.5 Hz, 1H), 4.23 – 4.13 (m, 2H), 3.76 (s, 3H), 3.75 (s, 3H), 3.01 – 2.72 (m, 1H), 2.23 (dd, *J* = 20.3, 3.8 Hz, 1H), 1.40 (s, 9H), 1.25 (t, *J* = 7.1 Hz, 3H) (signal broadening due to rotamers); **^{13}C NMR** (101 MHz, $CDCl_3$) δ = 163.8 (q), 162.9 (q), 162.6 (q), 152.8 (q), 147.8 (q), 137.1 (+), 135.6 (q), 134.8 (q), 81.0 (q), 60.8 (–), 58.6 (+), 57.9 (+), 52.43 (+), 52.36 (+), 28.2 (+), 25.9 (–), 14.2 (+) (signal broadening due to rotamers); **IR ṽ** [cm^{-1}]: 2960, 1707, 1640, 1439, 1368, 1252, 1118, 1156, 1080, 1029, 943, 857, 783; **HRMS** (ESI): calcd. for $C_{19}H_{25}NO_8$ $(M+Na)^+$, m/z = 418.1472; found 418.1475.

9-(*Tert*-butyl) 5-ethyl 7,8-dimethyl (1*S*,2*S*,4*R*,5*R*,6*S*)-3-oxa-9-azatricyclo[4.2.1.0^{2,4}]non-7-ene-5,7,8,9-tetracarboxylate (14)

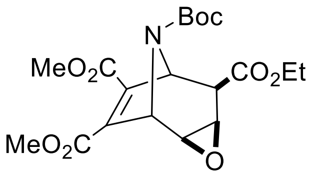

Cycloadduct **7c** (327 mg, 826 µmol, 1.0 equiv) was dissolved in CH_2Cl_2 (15 mL). Then *m*-CPBA (285 mg, 1.65 mmol, 2.0 equiv) was added at 25 °C. After stirring for 1 d, additional *m*-CPBA (285 mg, 1.65 mmol, 2.0 equiv) was added and the reaction was stirred for further 2 d at 25 °C. The reaction was quenched with saturated aqueous sodium thiosulfate. Then the aqueous layer was extracted with CH_2Cl_2 (3 x 20 mL). The combined organic phases were washed with saturated aqueous $NaHCO_3$ (3 x 30 mL), brine (2 x 30 mL) and were dried over

Na_2SO_4. The solvent was removed under reduced pressure and epoxide **14** (336 mg, 816 µmol, 99%) was obtained as a colorless solid.

R*f* = 0.39 (PE:EA = 3:2; UV, $KMnO_4$); **m.p.** = 122 °C; **^{1}H NMR** (300 MHz, $CDCl_3$): δ = 5.40 – 5.28 (m, 1H), 5.23 – 4.89 (m, 1H), 4.40 – 4.16 (m, 2H), 3.85 (s, 3H), 3.83 (s, 3H), 3.52 – 3.34 (m, 1H), 3.35 – 3.24 (m, 1H), 2.77 (d, *J* = 4.5 Hz, 1H), 1.43 (s, 9H), 1.32 (t, *J* = 7.1 Hz, 3H) (signal broadening and doubling due to rotamers); **^{13}C NMR** (101 MHz, $CDCl_3$): δ = 168.9 (q), 168.8 (q), 163.0 (q), 162.4 (q), 152.6 (q), 152.1 (q), 142.2 (q) 142.1 (q), 141.7 (q), 140.6 (q), 80.8 (q), 61.5 (–), 59.8 (+), 59.7 (+), 58.8 (+), 58.0 (+), 52.7 (+), 49.2 (+), 46.9 (+), 38.8 (+), 38.0 (+), 28.1 (+), 14.1 (+) (signal broadening and doubling due to rotamers); **IR** $\tilde{\nu}$ [cm^{-1}]: 2989, 2956, 2904, 1730, 1696, 1651, 1416, 1349, 1267, 1223, 1197, 1118, 1036, 954, 891, 764, 727, 686; **HRMS** (ESI): calcd. for $C_{19}H_{25}NO_9$ $(M+H)^+$, m/z = 412.1602; found 412.1610.

8-(*Tert*-butyl) 2-ethyl 6,7-dimethyl (1*S*,4*R*,5*S*)-4-hydroxy-8-azabicyclo[3.2.1]octa-2,6-diene-2,6,7,8-tetracarboxylate (15)

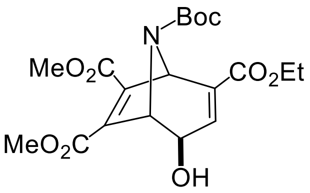

Flash column chromatography (25% EA:PE + 1% TEA) of epoxide **14** (336 mg, 816 µmol, 1.0 equiv) resulted in allylic alcohol **15** (259 mg, 630 µmol, 77%) as a colorless oil.

R*f* = 0.41 (PE:EA = 3:2; UV, $KMnO_4$); **^{1}H NMR** (300 MHz, $CDCl_3$): δ = 6.71 – 6.60 (m, 1H), 5.56 – 5.33 (m, 1H), 5.13 – 5.04 (m, 1H), 4.40 – 4.15 (m, 3H), 3.81 (s, 3H), 3.80 (s, 3H), 1.44 (s, 9H), 1.31 (t, *J* = 7.1 Hz, 3H) (signal broadening due to rotamers); **^{13}C NMR** (101 MHz, $CDCl_3$): δ = 163.7 (q), 162.6 (q), 162.3 (q), 155.5 (q), 150.0 (q), 137.7 (+), 137.1 (q), 135.8 (q), 82.3 (q), 65.2 (+), 64.9 (+), 61.3 (–), 59.8 (+), 52.58 (+), 52.56 (+), 28.2 (+), 14.2 (+) (signal broadening due to rotamers); **IR** $\tilde{\nu}$ [cm^{-1}]: 3440, 2982, 1711, 1435, 1394, 1372, 1323, 1252, 1163, 1118, 1088, 1040, 951, 921, 775; **HRMS** (ESI): calcd. for $C_{19}H_{25}NO_9$ $(M+H)^+$, m/z = 412.1602; found 412.1600.

8-(*Tert*-butyl) 2-ethyl 6,7-dimethyl (1*S*,2*R*,3*S*,4*S*,5*S*)-3-(benzoyloxy)-4-bromo-8-azabicyclo[3.2.1]oct-6-ene-2,6,7,8-tetracarboxylate (16)

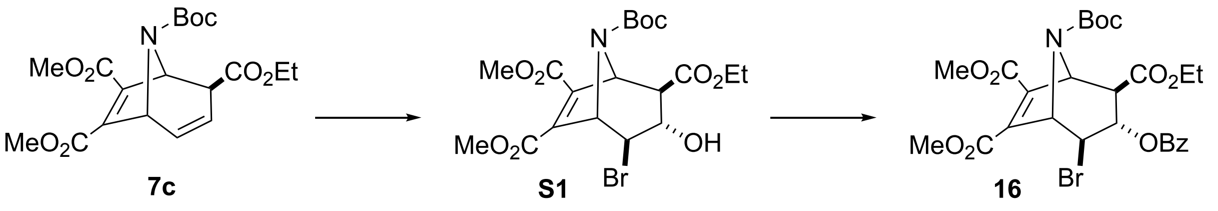

To a stirred solution of cycloadduct **7c** (641 mg, 1.62 mmol, 1.0 equiv) in acetone:H_2O (3 mL; 3:1 v/v) NBS (577 mg, 3.24 mmol, 2.0 equiv) was added in portions within 45 min at 0 °C under exclusion of light. The reaction mixture was allowed to warm up to 25 °C and stirred for 21 h at 25 °C. The reaction was quenched with saturated aqueous sodium metabisulfite until the initial yellow color had faded. Acetone was removed under reduced pressure. Then the residue was redissolved in diethyl ether and the aqueous phase was extracted with diethyl ether (3 x 20 mL). The combined organic phases were washed with H_2O (2 x 30 mL), brine (2 x 30 mL) and dried over Na_2SO_4. Removal of the solvent under reduced pressure afforded halohydrin **S1** (confirmed by X-ray analysis, *vide infra*) as a colorless oil. To a solution of halohydrin in CH_2Cl_2 (9 mL) benzoyl chloride (342 mg, 280 µL, 2.43 mmol, 1.5 equiv), TEA (820 mg, 1.12 mL, 8.10 mmol, 5.0 equiv) and DMAP (89.1 mg, 729 µmol, 0.5 equiv) were added. After stirring for 8 h at 25 °C, the reaction mixture was diluted with CH_2Cl_2 (5 mL). The reaction mixture was washed with saturated aqueous $NaHCO_3$ (5 x 10 mL) and brine (10 mL). The combined organic phases were dried over Na_2SO_4 and the solvent was removed under reduced pressure. Purification of the crude product by flash system (10 → 15% EA:PE) yielded compound **16** (571 mg, 957 µmol, 59%) as a colorless oil.

R_f = 0.35 (PE:EA = 3:1; UV, $KMnO_4$); **^{1}H NMR** (400 MHz, $CDCl_3$): δ = 7.94 – 7.81 (m, 2H), 7.61 – 7.49 (m, 1H), 7.43 – 7.37 (m, 2H), 6.13 – 6.01 (m, 1H), 5.77 – 5.45 (m, 1H), 5.45 – 5.04 (m, 1H), 4.45 – 4.35 (m, 1H), 4.33 – 4.13 (m, 2H), 3.73 (s, 3H), 3.64 (s, 3H), 3.23 – 3.03 (m, 1H), 1.47 (s, 9H), 1.33 (t, *J* = 7.0 Hz, 3H) (signal broadening due to rotamers); **^{13}C NMR** (101 MHz, $CDCl_3$): δ = 168.3 (q), 164.5 (q), 162.8 (q), 162.5 (q), 152.8 (q), 143.4 (q), 140.6 (q), 133.5 (+), 129.7 (+), 129.0 (q), 128.4 (+), 81.5 (q), 73.3 (+), 65.3 (+), 62.0 (–), 60.0 (+), 52.54 (+), 52.50 (+), 45.2 (+), 43.2 (+), 28.2 (+), 14.2 (+) (signal broadening due to rotamers); **IR** ṽ [cm^{-1}]: 2982, 1711, 1651, 1439, 1368, 1323, 1241, 1159, 1085, 1025, 992, 947, 857, 767, 712; **HRMS** (ESI): calcd. for $C_{26}H_{30}BrNO_{10}$ $(M+H)^+$, m/z = 596.1126; found 596.1129.

2-Ethyl 6,7-dimethyl (1*S*,2*R*,3*S*,4*S*,5*S*)-3-(benzoyloxy)-4-bromo-8-methyl-8-azabicyclo[3.2.1]oct-6-ene-2,6,7-tricarboxylate (17)

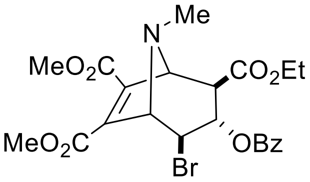

Cycloadduct **16** (229 mg, 384 µmol, 1.0 equiv) was dissolved in CH_2Cl_2 (4 mL) and TFA (1.44 g, 970 µL, 12.7 mmol, 33 equiv) was added dropwise at 25 °C. After stirring for 1.5 h at 25 °C, the reaction mixture was diluted with CH_2Cl_2 (5 mL) and saturated aqueous $NaHCO_3$ was added dropwise. The phases were separated, and the aqueous layer was extracted with CH_2Cl_2 (3 x 10 mL). The combined organic layers were washed with brine (25 mL) and dried over Na_2SO_4. Then the solvent was removed under reduced pressure and the crude amine was obtained as a yellowish oil. To a solution of crude amine and formaldehyde (70.3 mg, 176 µL, 2.34 mmol, 6.0 equiv) in MeCN 5 mL) was added $NaBH_3CN$ (71.4 mg, 1.14 mmol, 3.0 equiv) and the reaction mixture was stirred for 1 h at 25 °C. The solution was acidified to pH 6 with HOAc and stirred for 1.5 h. After neutralization to pH 9 with NH_3 (25%), the mixture was diluted with CH_2Cl_2 (10 mL) and saturated aqueous $NaHCO_3$ (10 mL). The phases were separated, and the aqueous layer was extracted with CH_2Cl_2 (5 x 10 mL). The combined organic layers were washed with brine (30 mL) and dried over Na_2SO_4. After removal of the solvent under reduced pressure, the crude product was purified by flash system (18 → 39% EA:PE) to afford cycloadduct **17** (98.9 mg, 194 µmol, 50%) as a colorless oil.

R_f = 0.74 (PE:EA = 1:1; UV, $KMnO_4$); **^{1}H NMR** (300 MHz, $CDCl_3$): δ = 7.97 – 7.84 (m, 2H), 7.60 – 7.48 (m, 1H), 7.45 – 7.33 (m, 2H), 6.09 (t, *J* = 0.9 Hz, 1H), 4.43 – 4.24 (m, 4H), 3.96 (dt, *J* = 2.3, 0.8 Hz, 1H), 3.71 (s, 3H), 3.64 (s, 3H), 3.04 (dd, *J* = 2.3, 1.1 Hz, 1H), 2.43 (s, 3H), 1.33 (t, *J* = 7.1 Hz, 3H); **^{13}C NMR** (101 MHz, $CDCl_3$): δ = 169.2 (q), 164.7 (q), 164.2 (q), 163.9 (q), 141.0 (q), 139.9 (q), 133.3 (+), 129.8 (+), 129.3 (q), 128.3 (+), 74.6 (+), 72.8 (+), 69.6 (+), 61.8 (–), 52.3 (+), 46.1 (+), 44.6 (+), 41.8 (+), 14.2 (+); **IR** $\tilde{\nu}$ [cm^{-1}]: 2952, 1715, 1648, 1435, 1372, 1320, 1245, 1088, 1025, 943, 790, 865, 790, 708; **HRMS** (ESI): calcd. for $C_{22}H_{24}BrNO_8$ $(M+H)^+$, m/z = 510.0758; found 510.0762.

8-(*Tert*-butyl) 2-ethyl 6,7-dimethyl (1*S*,2*S*,5*R*,6*R*,7*S*)-6,7-dihydroxy-8-azabicyclo[3.2.1]oct-3-ene-2,6,7,8-tetracarboxylate (18)

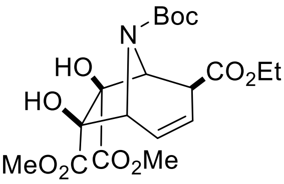

To a solution of cycloadduct **7c** (121 mg, 306 µmol, 1.0 equiv) in acetone (1.5 mL) and H_2O (1.2 mL) was added NMO (82.7 mg, 612 µmol, 2.0 equiv) followed by $K_2OsO_4{\cdot}2H_2O$ (6 mg, 15 µmol, 5 mol%) at 0 °C. The reaction mixture was stirred for 12 h at 0 °C. Then the reaction mixture was filtered through a short plug of silica and the solvent was removed under reduced pressure. Recrystallization from diethyl ether yielded diol **18** (57.0 mg, 137 µmol, 43%) as a colorless solid.

R_f = 0.55 (PE:EA = 1:2; Seebach's stain, $KMnO_4$); **m.p.** = 131 °C; **^{1}H NMR** (400 MHz, $CDCl_3$): δ = 6.30 – 6.19 (m, 1H), 5.85 – 5.73 (m, 1H), 5.16 – 4.99 (m, 1H), 4.97 – 4.84 (m, 1H), 4.77 – 4.54 (m, 2H), 4.22 – 4.09 (m, 2H), 3.76 (s, 3H), 3.67 (s, 3H), 3.15 – 2.93 (m, 1H), 1.43 (s, 9H), 1.28 – 1.22 (m, 3H) (signal broadening and doubling due to rotamers); **^{13}C NMR** (101 MHz, $CDCl_3$): δ = 170.9 (q), 170.8 (q), 170.3 (q), 169.7 (q), 169.4 (q), 154.3 (q), 128.6 (+), 128.5 (+), 124.5 (+), 124.4 (+), 89.1 (q), 88.4 (q), 85.6 (q), 84.8 (q), 81.0 (q), 80.9 (q), 67.2 (+), 65.7 (+), 62.1 (+), 61.6 (–), 61.4 (–), 60.7 (+), 53.3 (+), 53.2 (+), 52.4 (+), 52.3 (+), 45.2 (+), 45.0 (+), 28.1 (+), 14.2 (+), 14.1 (+) (signal doubling due to rotamers); **IR** $\tilde{\nu}$ [cm^{-1}]: 3295, 2978, 1744, 1670, 1413, 1368, 1334, 1279, 1163, 1126, 1059, 1025, 962, 921, 861, 701, 675; **HRMS** (ESI): calcd. for $C_{19}H_{27}NO_{10}$ $(M+H)^+$, m/z = 430.1708; found 430.1713.

Triethyl (1*S*,2*R*,3*R*,4*S*,5*S*)-3,4-dihydroxy-8-tosyl-8-azabicyclo[3.2.1]oct-6-ene-2,6,7-tricarboxylate (19)

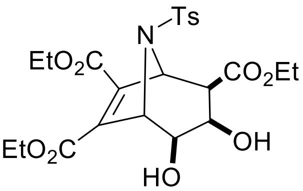

According to literature procedure[11], to a vigorously stirred solution of the cycloaddition product **7f** (137 mg, 287 µmol, 1.0 equiv) in MeCN (1.7 mL) was added a solution of $RuCl_3{\cdot}3H_2O$ (4 mg, 17.7 µmol, 6 mol%) and $NaIO_4$ (95.0 mg, 444 µmol, 1.6 equiv) in H_2O (0.3 mL) at 0 °C. The mixture was allowed to warm to 25 °C and stirred for 2 d. Then the suspension was filtered

through a short plug of silica gel, which was washed with EA. Concentration of the filtrate and purification by flash column chromatography (33% EA:PE) yielded diol **19** (69.0 mg, 135 µmol, 47%) as a colorless oil.

R*f* = 0.25 (PE:EA = 2:1; UV); **^{1}H NMR** (300 MHz, $CDCl_3$): δ = 7.66 – 7.57 (m, 2H), 7.29 – 7.22 (m, 2H, overlapping with solvent signal), 5.03 (dd, *J* = 2.6, 1.1 Hz, 1H), 4.86 (s, 1H), 4.32 – 4.11 (m, 6H), 3.98 (bs, 1H), 3.85 (bs, 1H), 3.32 (dd, *J* = 6.3, 2.6 Hz, 1H), 2.39 (s, 3H), 1.36 (t, *J* = 7.1 Hz, 3H), 1.26 – 1.20 (m, 6H); **^{13}C NMR** (75 MHz, $CDCl_3$): δ = 172.1 (q), 161.4 (q), 161.1 (q), 144.5 (q), 138.9 (q), 137.3 (q), 133.9 (q), 130.1 (+), 127.7 (+), 68.8 (+), 65.8 (+), 65.1 (+), 64.9 (+), 62.6 (–), 61.9 (–), 61.8 (–), 46.3 (+), 21.6 (+), 14.1 (+), 14.02 (+), 13.99 (+); **IR** $\tilde{\nu}$ [cm^{-1}]: 3440, 2982, 1700, 1394, 1364, 1256, 1163, 1025, 931, 880, 723; **HRMS** (ESI): calcd. for $C_{23}H_{29}NO_{10}$ $(M+Na)^+$, m/z = 534.1404; found 534.1400.

8-(*Tert*-butyl) 2,6,7-trimethyl (1*S*,2*R*,3*S*,4*S*,5*S*)-3-(benzoyloxy)-4-bromo-8-azabicyclo[3.2.1]oct-6-ene-2,6,7,8-tetracarboxylate (20)

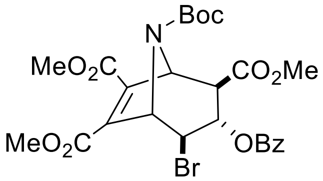

To a stirred solution of cycloadduct **7d** (620 mg, 1.63 mmol, 1.0 equiv) in acetone:H_2O (8 mL; 4:1 v/v) NBS (1.16 g, 6.52 mmol, 4.0 equiv) was added in portions within 45 min at 0 °C under exclusion of light. Subsequently, the ice bath was removed, and the reaction mixture was stirred for 3 d at 25 °C. The reaction was quenched with saturated aqueous sodium metabisulfite until the initial yellow color had faded. Acetone was removed under reduced pressure. Then the residue was redissolved in diethyl ether and the aqueous phase was extracted with diethyl ether (3 x 15 mL). The combined organic phases were washed with H_2O (2 x 30 mL), brine (2 x 30 mL) and dried over Na_2SO_4. The solvent was removed under reduced pressure and the crude halohydrin was obtained as a colorless oil. To a solution of halohydrin in CH_2Cl_2 (8 mL) benzoyl chloride (344 mg, 282 µL, 2.45 mmol, 1.5 equiv), TEA (825 mg, 1.13 mL, 8.15 mmol, 5.0 equiv) and DMAP (89.6 mg, 734 µmol, 0.5 equiv) were added. After stirring for 15 h at 25 °C, the reaction mixture was diluted with CH_2Cl_2 (15 mL). The mixture was washed with saturated aqueous $NaHCO_3$ (5 x 20 mL) and brine (30 mL). The combined organic phases were dried over Na_2SO_4 and the solvent was removed under

reduced pressure. Purification of the crude product by flash system (10 → 18% EA:PE) yielded compound **20** (407 mg, 700 µmol, 43%) as a colorless oil.

R*f* = 0.58 (PE:EA = 3:2; UV, $KMnO_4$); **^{1}H NMR** (300 MHz, $CDCl_3$): δ = 7.94 – 7.83 (m, 2H), 7.60 – 7.50 (m, 1H), 7.44 – 7.35 (m, 2H), 6.17 – 6.00 (m, 1H), 5.73 – 5.51 (m, 1H), 5.38 – 5.08 (m, 1H), 4.53 – 4.25 (m, 1H), 3.83 (s, 3H), 3.73 (s, 3H), 3.66 (s, 3H), 3.22 – 3.05 (m, 1H), 1.48 (s, 9H) (signal broadening due to rotamers); **^{13}C NMR** (75 MHz, $CDCl_3$): δ = 168.8 (q), 164.5 (q), 162.8 (q), 162.5 (q), 152.5 (q), 147.0 (q), 141.8 (q), 133.5 (+), 129.7 (+), 129.0 (q), 128.4 (+), 81.6 (q), 73.3 (+), 64.5 (+), 60.1 (+), 52.8 (+), 52.59 (+), 52.56 (+), 45.0 (+), 43.1 (+), 28.2 (+) (signal broadening due to rotamers); **IR** $\tilde{\nu}$ [cm^{-1}]: 2978, 1707, 1651, 1435, 1394, 1323, 1241, 1163, 1085, 1025, 988, 857, 760, 712; **HRMS** (ESI): calcd. for $C_{25}H_{28}BrNO_{10}$ $(M+H)^+$, m/z = 582.0969; found 582.0972.

2-(*Tert*-butyl) 5,6,7-trimethyl (1*S*,4*R*,7*R*,8*R*)-8-(benzoyloxy)-2-azabicyclo[2.2.2]oct-5-ene-2,5,6,7-tetracarboxylate (21)

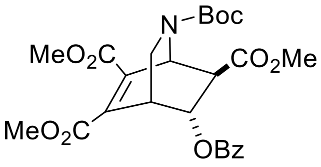

Tributyltin hydride (80.0 mg, 75 µL, 275 µmol, 1.6 equiv) and AIBN (3 mg, 19.7 µmol, 12 mol%) in dry benzene (4 mL) were added to a solution of cycloadduct **20** (98.0 mg, 168 µmol, 1.0 equiv) in dry benzene (7 mL) at reflux under N_2-atmosphere. The reaction mixture was stirred for 5 h at reflux and the solvent was removed under reduced pressure. Then the residue was redissolved in EA (5 mL) and a saturated solution of KF (5 mL) was added. After stirring for 18 h at 25 °C, the white precipitate was removed by filtration. The phases were separated, and the aqueous phase was extracted with EA (3 x 10 mL). The combined organic phases were washed with brine (30 mL) and the solvent was removed under reduced pressure. The crude product was purified by flash column chromatography (flash silica gel + 10% KF, 10% → 25% EA:PE) to afford cycloadduct **21** (62.0 mg, 123 µmol, 73%) as a colorless oil.

R*f* = 0.35 (PE:EA = 3:1; UV, $KMnO_4$); **^{1}H NMR** (400 MHz, $CDCl_3$): δ = 8.01 – 7.92 (m, 2H), 7.59 – 7.51 (m, 1H), 7.44 – 7.38 (m, 2H), 5.68 – 5.61 (m, 1H), 5.59 – 5.40 (m, 1H), 3.84 (s, 3H), 3.77 (s, 6H), 3.62 – 3.44 (m, 2H), 3.25 – 3.16 (m, 1H), 2.76 – 2.64 (m, 1H), 1.42 (s, 9H)

(signal broadening and doubling due to rotamers); ^{13}C **NMR** (101 MHz, $CDCl_3$): δ = 170.7 (q), 170.2 (q), 165.9 (q), 165.7 (q), 163.4 (q), 163.2 (q), 154.1 (q), 153.6 (q), 141.1 (q), 140.7 (q), 136.7 (q), 136.4 (q), 133.4 (+), 129.8 (+), 129.3 (q), 128.4 (+), 80.6 (q), 71.7 (+), 71.4 (+), 52.73 (+), 52.65 (+), 52.52 (+), 52.48 (+), 49.9 (+), 48.7 (+), 42.6 (–), 42.2 (–), 38.6 (+), 38.3 (+), 28.3 (+) (signal broadening and doubling due to rotamers); **IR** ṽ [cm^{-1}]: 2956, 1722, 1651, 1439, 1394, 1267, 1174, 1111, 1029, 865, 757, 716; **HRMS** (ESI): calcd. for $C_{25}H_{29}NO_{10}$ $(M+Na)^+$, m/z = 526.1684; found 526.1686.

Trimethyl (1*S*,4*R*,7*R*,8*R*)-8-(benzoyloxy)-2-methyl-2-azabicyclo[2.2.2]oct-5-ene-5,6,7-tricarboxylate (22)

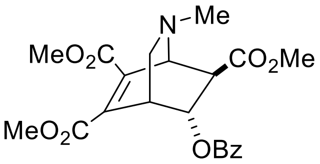

Cycloadduct **21** (59.0 mg, 117 µmol, 1.0 equiv) was dissolved in CH_2Cl_2 (4 mL) and TFA (441 mg, 296 µL, 3.87 mmol, 33 equiv) was added dropwise at 25 °C. After stirring for 1 h at 25 °C, the reaction mixture was diluted with CH_2Cl_2 (5 mL) and saturated aqueous $NaHCO_3$ was added dropwise. The phases were separated, and the aqueous layer was extracted with CH_2Cl_2 (3 x 10 mL). The combined organic layers were washed with brine (30 mL) and dried over Na_2SO_4. Then the solvent was removed under reduced pressure and the crude amine was obtained as colorless oil. To a solution of crude amine and formaldehyde (39.6 mg, 99 µL, 1.32 mmol, 11 equiv) in MeCN (5 mL) $NaBH_3CN$ (60.0 mg, 955 µmol, 8.2 equiv) was added and the reaction mixture was stirred for 1 h at 25 °C. The solution was acidified to pH 6 with HOAc and stirred for 1.5 h at 25 °C. After neutralization to pH 9 with NH_3 (25%), the mixture was diluted with CH_2Cl_2 (10 mL) and saturated aqueous $NaHCO_3$ (10 mL). The phases were separated, and the aqueous layer was extracted with CH_2Cl_2 (5 x 10 mL). The combined organic layers were washed with brine (30 mL) and dried over Na_2SO_4. After removal of the solvent under reduced pressure, the crude product was purified by flash column chromatography (40% EA:PE + 1% TEA) to afford cycloadduct **22** (29.6 mg, 70.9 µmol, 61%) as a colorless oil.

$\mathbf{R_f}$ = 0.48 (PE:EA 1:1; UV, $KMnO_4$); ^{1}H **NMR** (400 MHz, $CDCl_3$): δ = 7.98 – 7.91 (m, 2H), 7.58 – 7.49 (m, 1H), 7.45 – 7.36 (m, 2H), 5.73 (t, *J* = 3.0 Hz, 1H), 4.19 (d, *J* = 2.9 Hz, 1H), 3.85 (s, 3H), 3.80 (s, 3H), 3.71 (s, 3H), 3.49 (q, *J* = 2.8 Hz, 1H), 3.34 (dd, *J* = 10.9, 2.0 Hz, 1H), 2.63

(t, *J* = 2.8 Hz, 1H), 2.25 (s, 3H), 2.00 (dd, *J* = 10.9, 3.0 Hz, 1H); **^{13}C NMR** (101 MHz, $CDCl_3$): δ = 171.0 (q), 165.8 (q), 165.6 (q), 165.4 (q), 137.8 (q), 137.3 (q), 133.1 (+), 129.74 (q), 129.67 (+), 128.3 (+), 70.8 (+), 58.6 (+), 52.6 (+), 52.52 (+), 52.50 (+), 52.3 (+), 50.8 (–), 44.8 (+), 37.7 (+); **IR** $\tilde{\nu}$ [cm^{-1}]: 3004, 2952, 2855, 2803, 1718, 1644, 1435, 1260, 1200, 1144, 1111, 1074, 958, 887, 861, 753, 716; **HRMS** (ESI): calcd. for $C_{21}H_{23}NO_8$ $(M+H)^+$, m/z = 418.1496; found 418.1499.

Derivatizations of the dimethyl maleate moiety in 7a

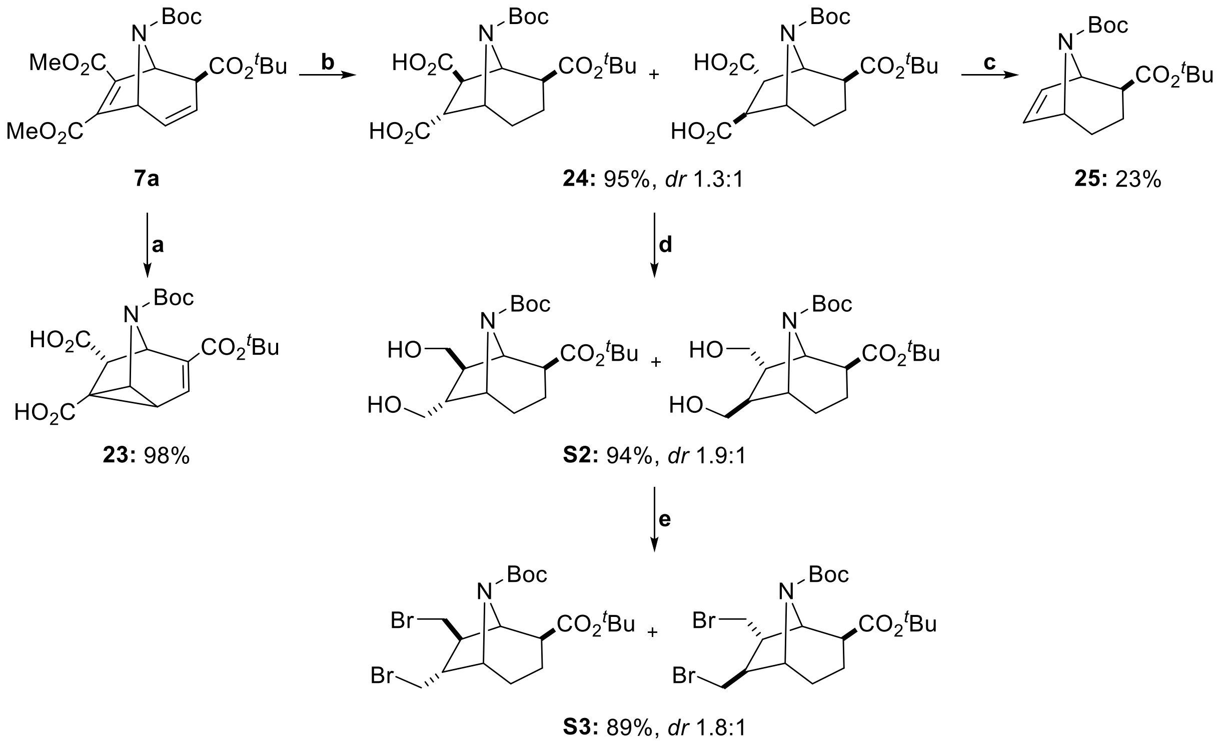

Scheme 1. Derivatizations of **7a**. Conditions: a) NaOH (2.0 equiv), THF, 0 to 25 °C, 5.5 h, then HCl, 0 °C, 98%; b) *(i)* H_2, Pd/C (10 mol%), EtOH/THF (1:4 v/v), 60 bar, 25 °C, 4 h, *(ii)* NaOH (2.0 equiv), THF, 0 to 25°C, 4 h, then HCl, 0 °C, 95%; c) $Pb(OAc)_4$ (2.4 equiv), C_5H_5N, 67 °C, 6 h, 23%; d) BH_3·THF (6.5 equiv), THF, 0 to 25 °C, 24 h, 94%; e) *(i)* MsCl (2.2 equiv), TEA (4.0 equiv), CH_2Cl_2, 0 °C, 2 h, *(ii)* LiBr (13 equiv), THF, reflux, 9 h, 89%.

(5*R*,7*R*, 8*S*)-4,6-Bis(*tert*-butoxycarbonyl)-6-azatricyclo[3.2.1.0^{2,7}]oct-3-ene-1,8-dicarboxylic acid (23)

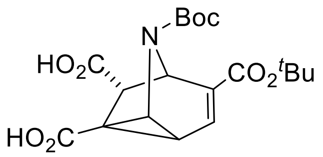

1M NaOH (510 µL, 20.4 mg, 510 µmol, 2.0 equiv) was added dropwise to a solution of cycloadduct **7a** (108 mg, 255 µmol, 1.0 equiv) in THF (2 mL) at 0 °C. Subsequently, the cooling bath was removed and the mixture was stirred for 5.5 h at 25 °C. Then the solvent was removed under reduced pressure and the salt was redissolved in EA and water. The aqueous

layer was extracted with EA (3 x 15 mL). Afterwards the pH of the aqueous layer was adjusted to pH 2 by addition of 1M HCl solution. Then the aqueous layer was extracted with EA (3 x 15 mL) and the combined organic phases were washed with brine (40 mL) and dried over Na_2SO_4. Removal of the solvent under reduced pressure yielded cycloadduct **23** (99.0 mg, 250 µmol, 98%) as a colorless solid.

R*f* = 0.40 (PE:EA = 1:2; bromocresol green, $KMNO_4$); **m.p.** = 127 °C; **^{1}H NMR** (400 MHz, $CDCl_3$): δ = 7.16 (dd, *J* = 6.5, 2.0 Hz, 1H), 5.38 – 5.25 (m, 1H), 4.19 (d, *J* = 5.9 Hz, 1H), 3.51 – 3.48 (m, 1H), 3.03 – 2.95 (m, 1H), 1.47 (s, 9H), 1.41 (s, 9H) (signal broadening due to rotamers); **^{13}C NMR** (101 MHz, $CDCl_3$): δ = 175.4 (q), 173.8 (q), 162.0 (q), 153.9 (q), 134.0 (+), 129.2 (q), 81.8 (q), 81.2 (q), 52.8 (+), 43.7 (+), 42.5 (+), 31.0 (+), 28.11 (q), 28.06 (+), 24.9 (+) (signal broadening due to rotamers); **IR** ṽ [cm^{-1}]: 3474, 2978, 2937, 1703, 1621, 1368, 1312, 1282, 1252, 1159, 1111, 1033, 995, 921, 820, 772; **HRMS** (ESI): calcd. for $C_{19}H_{25}NO_8$ $(M+Na)^+$, m/z = 418.1472; found 418.1475.

(1*S*,2*S*,5*R*,6*R*,7*R*)-2,8-Bis(*tert*-butoxycarbonyl)-8-azabicyclo[3.2.1]octane-6,7-dicarboxylic acid (*major 24*)

(1*S*,2*S*,5*R*,6*S*,7*S*)-2,8-Bis(*tert*-butoxycarbonyl)-8-azabicyclo[3.2.1]octane-6,7-dicarboxylic acid (*minor 24*)

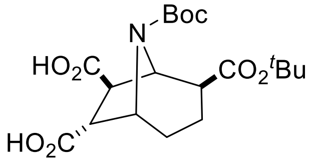

major **24**

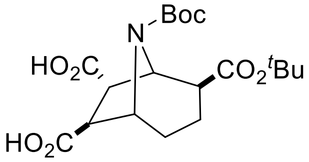

minor **24**

Cycloadduct **7a** (128 mg, 302 µmol, 1.0 equiv) was dissolved in EtOH:THF (3 mL; 1:4 v/v). Then Pd/C (3 mg, 30.2 µmol, 10 mol%, 10w% Pd on charcoal) was added and the reaction mixture was transferred in a vial which was placed in an autoclave and sealed. Then the autoclave was pressurized to 60 bar with H_2 and the solution was stirred for 4 h at 25 °C. The reaction mixture was filtered and the solvent was removed under reduced pressure. The crude product was dissolved in THF (2 mL) and a 1M NaOH (604 µL, 24.2 mg, 605 µmol, 2.0 equiv) was added dropwise at 0 °C. Subsequently, the cooling bath was removed and the mixture was stirred for 4 h h at 25 °C. After removal of the solvent, the residue was redissolved in EA and water and the phases were separated. The aqueous layer was extracted with EA (3 x 15 mL). Afterwards the pH of the aqueous layer was adjusted to pH 2 by addition of 1M HCl solution. Then the aqueous layer was extracted with EA (3 x 15 mL) and the combined

organic phases were washed with brine (30 mL) and dried over Na_2SO_4. Removal of the solvent under reduced pressure yielded diastereomeric mixture of compound **24** (115 mg, 287 µmol, 95%, *dr* 1.3:1) as a colorless solid.

In the proton and carbon NMR the signals of both diastereomers are overlapping. Characteristic signals of the minor diastereomer are marked.

R_f = 0.68 (CH_2Cl_2:MeOH = 9:1; bromocresol green, $KMNO_4$); **m.p.** = 173 °C; **^{1}H NMR** (400 MHz, $CDCl_3$): δ = 11.09 (s, 4H), 5.15 – 5.05 ^{minor} (m, 1H), 5.06 – 4.99 (m, 1.32H), 4.83 – 4.47 (m, 2.32H), 3.78 – 3.73 ^{minor} (m, 1H), 3.70 – 3.63 (m, 1.38H), 3.34 (d, *J* = 6.2 Hz, 1.34H), 3.29 ^{minor} (d, *J* = 6.1 Hz, 1H), 2.77 ^{minor} (dd, *J* = 6.0, 2.8 Hz, 1H), 2.65 (dd, *J* = 6.1, 2.9 Hz, 1.31H), 2.06 – 1.91 (m, 4.30H), 1.77 – 1.51 (m, 5.30H), 1.47 – 1.36 (m, 41.91H) (signal broadening due to rotamers); **^{13}C NMR** (101 MHz, $CDCl_3$): δ = 178.7 (q), 177.3 (q), 175.9 (q), 175.44 (q), 171.39 (q), 171.0 (q), 153.4 (q), 81.3 (q), 81.2 (q), 80.9 (q), 59.0 (+); 58.8 (+), 57.7 (+), 56.6 (+), 49.0 (+), 48.8 (+), 48.6 (+), 48.2 (+), 45.9 (+), 41.9 (+), 28.7 (–), 28.2 (+), 27.9 (+), 24.51 (–), 18.12 (–), 18.01 (–) (signal broadening due to rotamers); **IR** $\tilde{\nu}$ [cm^{-1}]: 3440, 2978, 2937, 1700, 1394, 1368, 1312, 1252, 1156, 1051, 1010, 943, 850, 757, 705; **HRMS** (ESI): calcd. for $C_{19}H_{29}NO_8$ $(M+H)^+$, m/z = 400.1966; found 400.1969 (*major* **24** t_r = 5.763-5.829 min); **HRMS** (ESI): calcd. for $C_{19}H_{29}NO_8$ $(M+H)^+$, m/z = 400.1966; found 400.1967 (*minor* **24** t_r = 5.829-5.908 min).

Di-*tert*-butyl (1*R*,2*S*,5*R*)-8-azabicyclo[3.2.1]oct-6-ene-2,8-dicarboxylate (25)

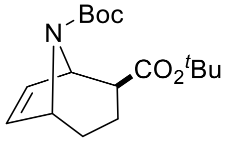

To a solution of compound **24** (99.7 mg, 227 µmol, 1.0 equiv) in dry pyridine (2.5 mL) was added $Pb(OAc)_4$ (141 mg, 318 µmol, 1.4 equiv) at 0 °C under N_2-atmosphere. Then the reaction temperature was gradually raised to 67 °C. After 3 h, additional $Pb(OAc)_4$ (101 mg, 227 µmol, 1.0 equiv) was added at 0 °C and the reaction mixture was stirred for 3 h at 67 °C. At 0 °C water (5 mL) was added and the mixture was filtrated through a short plug of celite. Then the aqueous phase was extracted with diethyl ether (3 x 15 mL). The combined organic phases were washed with 5% HCl (30 mL), saturated aqueous $NaHCO_3$ (30 mL), brine (30 mL) and dried over Na_2SO_4. The solvent was removed under reduced pressure and the crude product was purified by flash system (2 → 7% EA:PE) to afford cycloadduct **25** (16.3 mg, 52.7 µmol, 23%) as a colorless oil.

R$_f$ = 0.70 (PE:EA = 3:1; Vanillin, $KMNO_4$); **^{1}H NMR** (400 MHz, DMF-*d*$_7$, 333 K): δ = 6.29 (dd, *J* = 5.9, 2.3 Hz, 1H), 6.21 (dd, *J* = 5.9, 2.2 Hz, 1H), 4.98 (t, *J* = 2.4 Hz, 1H), 4.75 – 4.58 (m, 1H), 2.41 (ddd, *J* = 6.3, 2.5, 1.0 Hz, 1H), 1.91 – 1.82 (m, 2H), 1.77 – 1.68 (m, 1H), 1.49 (s, 9H), 1.44 (s, 9H), 1.35 – 1.33 (m, 1H) (signal broadening due to rotamers); **^{13}C NMR** (101 MHz, DMF-*d*$_7$, 333 K): δ = 172.3 (q), 153.7 (q), 133.7 (+), 132.4 (+), 80.3 (q), 79.3 (q), 61.1 (+), 59.7 (+), 40.8 (+), 28.4 (+), 28.1 (+), 22.8 (–), 18.8 (–) (signal broadening due to rotamers); **IR** ṽ [cm^{-1}]: 2975, 2930, 1730, 1700, 1368, 1312, 1252, 1163, 1051, 1013, 857; **HRMS** (ESI): calcd. for $C_{17}H_{27}NO_4$ $(M+H)^+$, m/z = 310.2013; found 310.2015.

Di-*tert*-butyl (1*R*,2*S*,5*R*,6*R*,7*R*)-6,7-bis(hydroxymethyl)-8-azabicyclo[3.2.1]octane-2,8-dicarboxylate (*major* S2)

Di-tert-butyl (1*R*,2*S*,5*R*,6*S*,7*S*)-6,7-bis(hydroxymethyl)-8-azabicyclo[3.2.1]octane-2,8-dicarboxylate (*minor* S2)

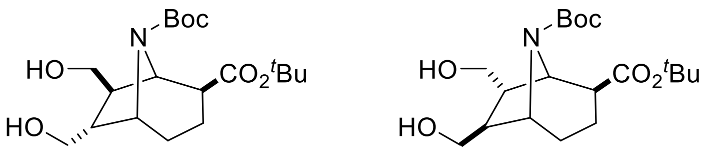

major **S2** *minor* **S2**

BH_3·THF (1.61 mL, 139 mg, 1.61 mmol, 3.5 equiv) was added dropwise to a solution of compound **24** (184 mg, 461 µmol, 1.0 equiv) in dry THF (4 mL) at 0 °C under N_2-atmosphere. The mixture was then warmed to 25 °C and after 8 h additional BH_3·THF (1.38 mL, 119 mg, 1.38 mmol, 3.0 equiv) was added at 0 °C, then the mixture was stirred for 16 h at 25 °C. Afterwards the reaction mixture was treated with MeOH and the solvent was removed under reduced pressure. The residue was redissolved in MeOH and evaporated in vacuo. This process was repeated three times and purification of the crude product by flash system (1 → 8% MeOH: CH_2Cl_2) afforded diastereomeric mixture of compound **S2** (160 mg, 431 µmol, 94%, *dr* 1.9:1) as a colorless oil.

In the proton and carbon NMR the signals of both diastereomers are overlapping. Characteristic signals of the minor diastereomer are marked.

R$_f$ = 0.57 (CH_2Cl_2:MeOH = 9:1; $KMNO_4$); **^{1}H NMR** (400 MHz, $CDCl_3$): δ = 4.68 – 4.58 ^{minor}(m, 1H), 4.45 – 4.27 (m, 2.93H), 4.23 – 4.09 (m, 2H), 3.82 – 3.61 (m, 13.62H), 3.36 – 3.20 (m, 3.81H), 2.50 – 2.41 (m, 1.94H), 2.37 – 2.29 ^{minor}(m, 1H), 2.21 – 1.85 (m, 12.66H), 1.65 – 1.51 (m, 4.35H), 1.43 – 1.36 (m, 53.88H) (signal broadening and doubling due to rotamers); **^{13}C NMR** (101 MHz, $CDCl_3$): δ = 172.4 (q), 172.1 (q), 171.9 (q), 171.6 (q), 154.9 (q), 153.8 (q),

153.3 (q), 81.0 (q), 80.9 (q), 80.64 (q), 80.60 (q), 80.1 (q), 80.0 (q), 79.6 (q), 79.5 (q), 65.8 (–), 65.61 (–), 65.55 (–), 65.5 (–), 61.1 (–), 60.8 (–), 60.6 (–), 59.3 (+), 59.0 (+), 57.5 (+), 57.3 (+), 57.2 (+), 56.7 (+), 56.2 (+), 55.9 (+), 49.3 (+), 45.7 (+), 45.6 (+), 41.6 (+), 28.4 (+), 28.1 (+), 27.9 (+), 23.7 (–), 19.3 (–), 19.2 (–), 19.1 (–), 19.0 (–) (signal broadening and doubling due to rotamers); **IR** ṽ [cm^{-1}]: 3370, 2974, 2930, 2874, 1726, 1662, 1476, 1420, 1364, 1252, 1163, 1115, 1062, 1021, 861, 753; **HRMS** (ESI): calcd. for $C_{19}H_{33}NO_6$ $(M+Na)^+$, m/z = 394.2200; found 394.2201 (*major* **S2** t_r = 5.481-5.518 min); **HRMS** (ESI): calcd. for $C_{19}H_{33}NO_6$ $(M+Na)^+$, m/z = 394.2200; found 394.2201 (*minor* **S2** t_r = 5.576-5.659 min).

Di-*tert*-butyl (1*S*,2*S*,5*R*,6*R*,7*R*)-6,7-bis(bromomethyl)-8-azabicyclo[3.2.1]octane-2,8-dicarboxylate (*major* S3)

Di-*tert*-butyl (1*S*,2*S*,5*R*,6*S*,7*S*)-6,7-bis(bromomethyl)-8-azabicyclo[3.2.1]octane-2,8-dicarboxylate (*minor* S3)

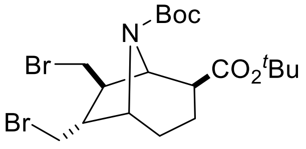

major **S3**

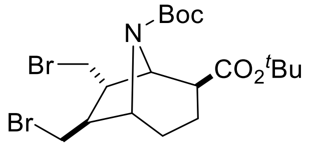

minor **S3**

MsCl (50 µL, 74.6 mg, 651 µmol, 2.2 equiv) was added dropwise to a solution of TEA (165 µL, 120 mg, 1.18 mmol, 4.0 equiv) and diol **S2** (110 mg, 296 µmol, 1.0 equiv) in CH_2Cl_2 (2.5 mL) at 0 °C. After stirring for 2 h at 0 °C, 1M HCl (5 mL) was added and the layers were separated. The organic layer was washed with 1M HCl (10 mL), 2M NaOH (10 mL) and brine (10 mL). The combined organic layers were dried over Na_2SO_4 and the solvent was removed under reduced pressure to obtain the crude mesylate as yellowish solid. The mesylate was dissolved in dry THF (4 mL) and LiBr (334 mg, 3.85 mmol, 13 equiv) was added in one portion at 25 °C under N_2-atmosphere. Then the reaction mixture was heated at reflux for 9 h and was concentrated in vacuo. The residue was redissolved in CH_2Cl_2 (10 mL) and washed with water (4 x 15 mL), brine (15 mL) and dried over Na_2SO_4. Removal of the solvent and purification by flash system (2 → 6% EA:PE) yielded diastereomeric mixture of *major* and *minor* cycloadduct **S3** (131 mg, 264 µmol, 89%, *dr* 1.8:1) as a colorless oil.

In the proton and carbon NMR the signals of both diastereomers are overlapping. Characteristic signals of the minor diastereomer are marked.

R_f = 0.63 (PE:EA = 5:1; UV, $KMnO_4$); **^{1}H NMR** (400 MHz, $CDCl_3$): δ = 4.84 – 4.57 (m, 2.59H), 4.51 – 4.14 (m, 3.26H), 3.76 – 3.61 (m, 2.66H), 3.52 – 3.25 (m, 9.30H), 2.58 (d, *J* = 6.0 Hz, 1H), 2.47^{minor} (dd, *J* = 6.4, 2.7 Hz, 1.79H), 2.39 – 2.15 (m, 3.38H), 2.16 – 1.92 (m, 7.96H), 1.71 – 1.54 (m, 3.83H), 1.47 – 1.40 (m, 51.58H) (signal broadening and doubling due to rotamers);

^{13}C NMR (101 MHz, $CDCl_3$): δ = 171.8 (q), 171.3 (q), 171.2 (q), 171.0 (q), 154.4 (q), 153.6 (q), 153.2 (q), 152.9 (q), 81.1 (q), 80.8 (q), 80.3 (q), 79.7 (q), 61.5 (+), 60.4 (+), 59.7 (+), 58.3 (+), 57.4 (+), 56.9 (+), 56.2 (+), 51.5 (+), 51.0 (+), 50.8 (+), 50.2 (+), 50.1 (+), 45.3 (+), 40.3 (+), 36.7(–), 36.5 (–), 36.3 (–), 35.9 (–), 30.7 (–), 30.3 (–), 30.0 (–), 29.8 (–), 29.7 (–), 28.3 (+), 28.0 (+), 22.6 (–), 22.4 (–), 21.9 (–), 21.6 (–), 19.2 (–), 19.1 (–) (signal broadening and doubling due to rotamers); **IR ṽ** [cm^{-1}]: 2974, 2928, 1730, 1696, 1394, 1368, 1320, 1256, 1170, 1115, 1036, 865; **HRMS** (ESI): calcd. for $C_{19}H_{31}Br_2NO_4$ $(M+Na)^+$, m/z = 496.0693; found 496.0706 (*major* and *minor* **S3** t_r = 7.565-7.660 min).

3. NMR spectra

Compound **4b**, ^{1}H NMR and ^{13}C NMR ($CDCl_3$):

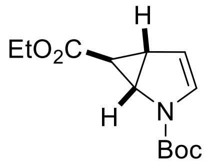

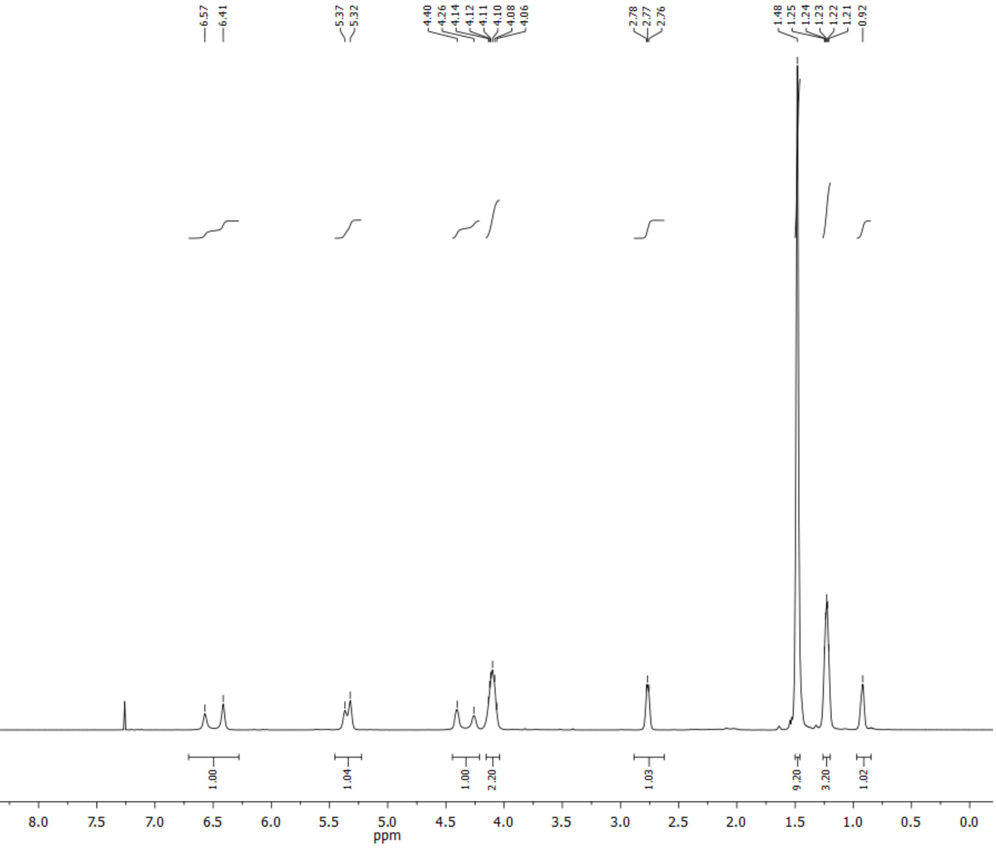

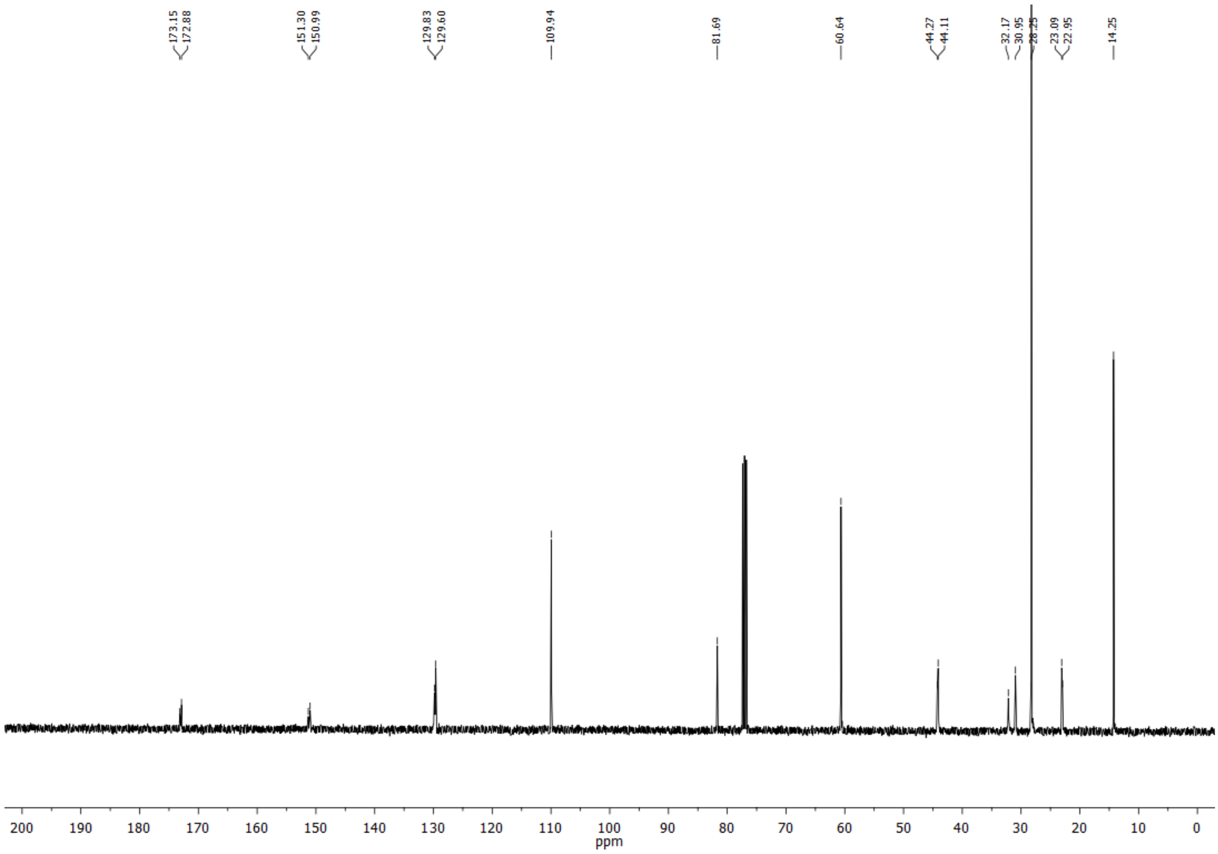

Compound **4e**, ^{1}H NMR and ^{13}C NMR ($CDCl_3$):

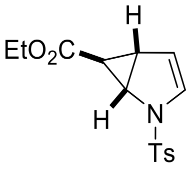

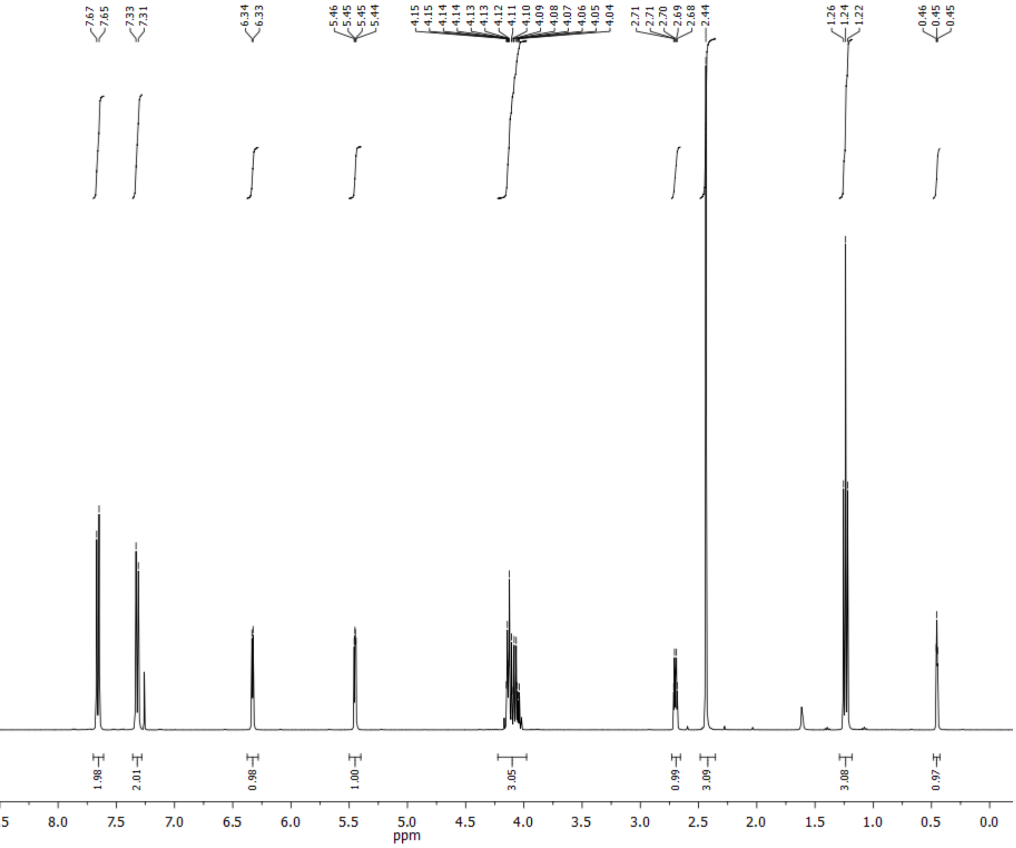

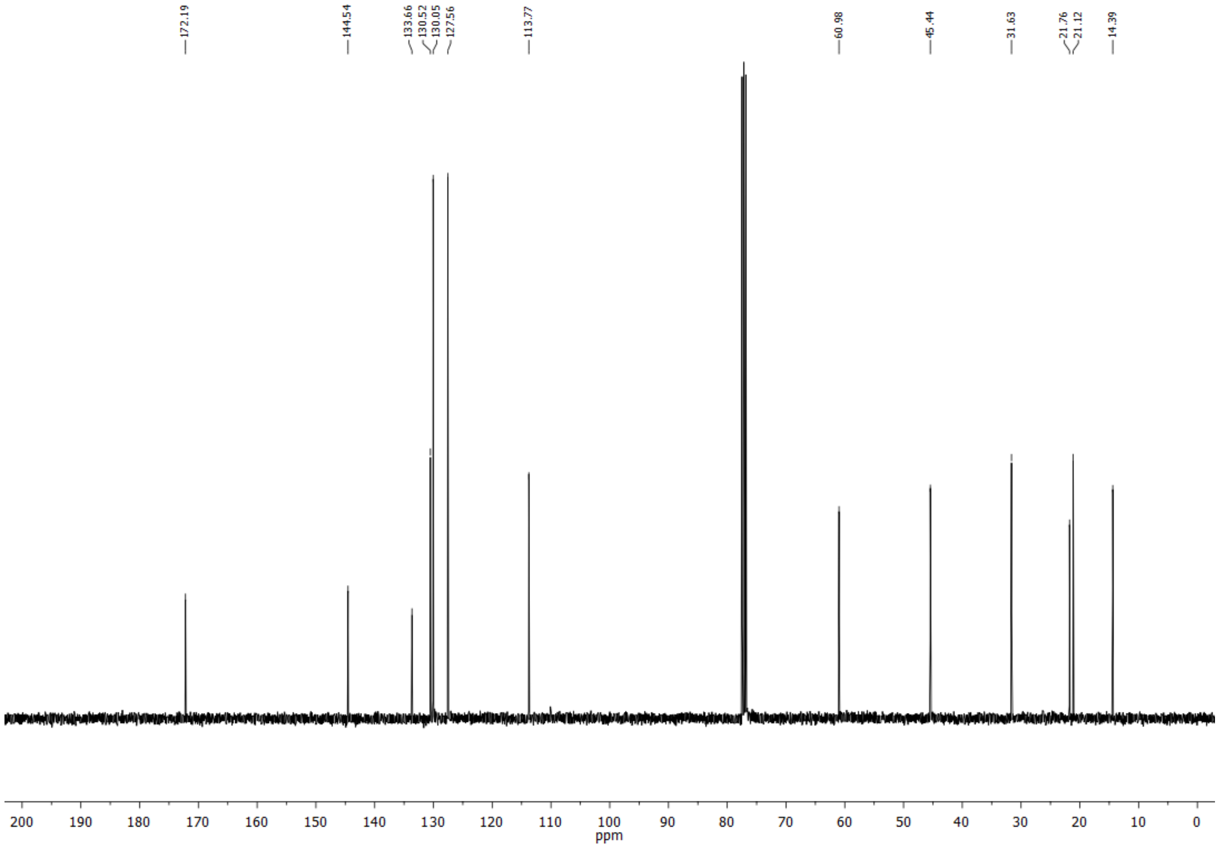

Compound **(+)-7a**, ^{1}H NMR and ^{13}C NMR ($CDCl_3$):

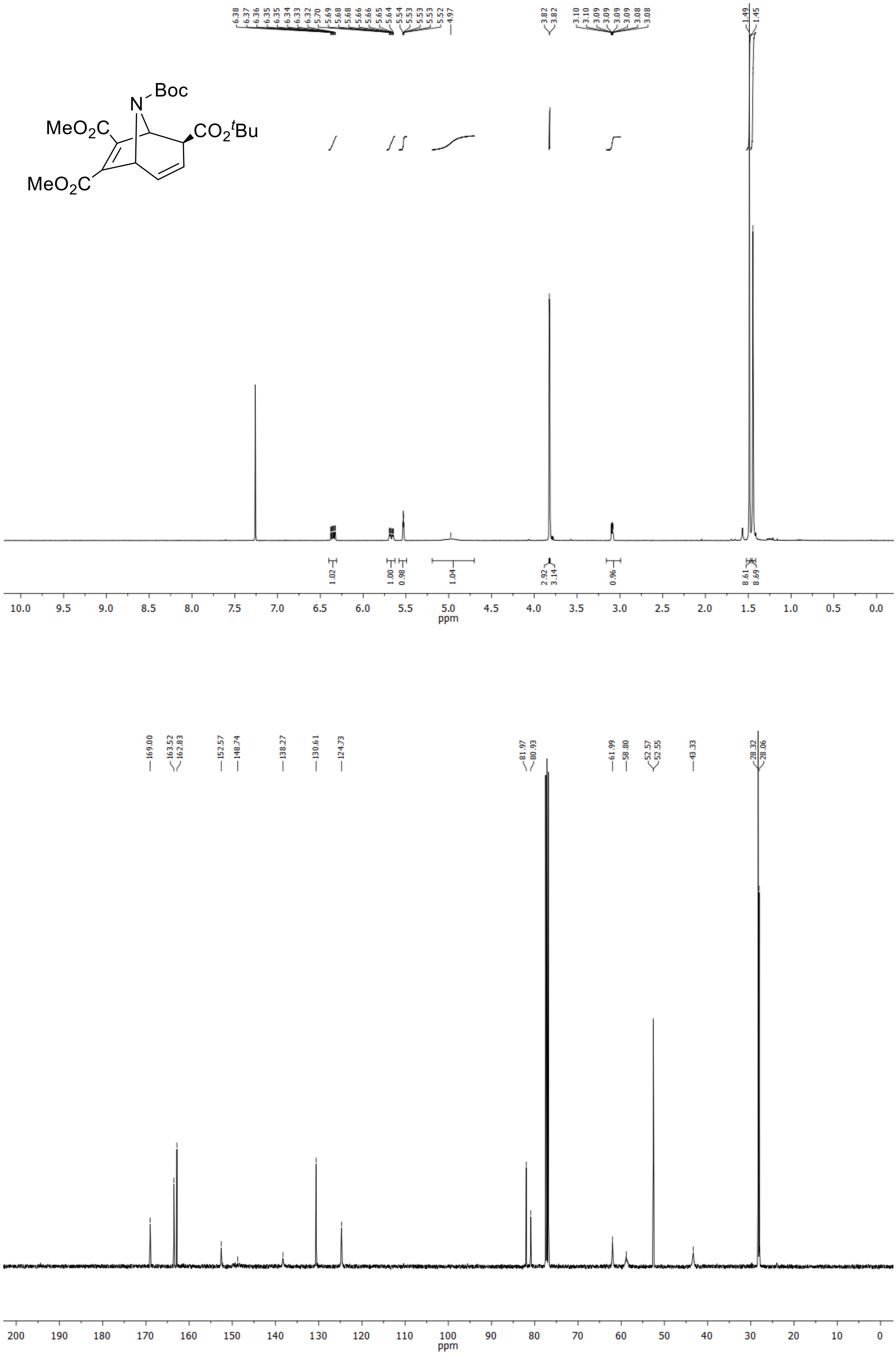

Compound **7b**, ^{1}H NMR and ^{13}C NMR ($CDCl_3$):

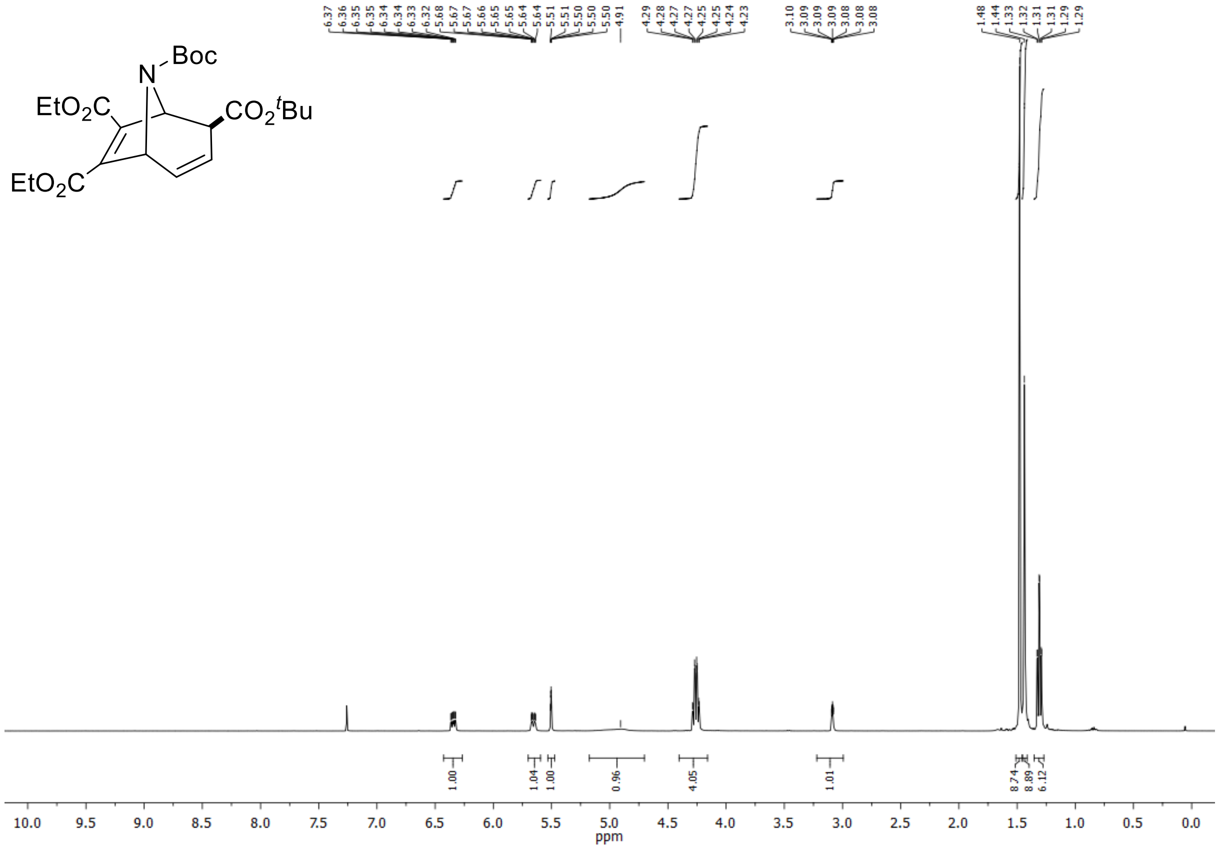

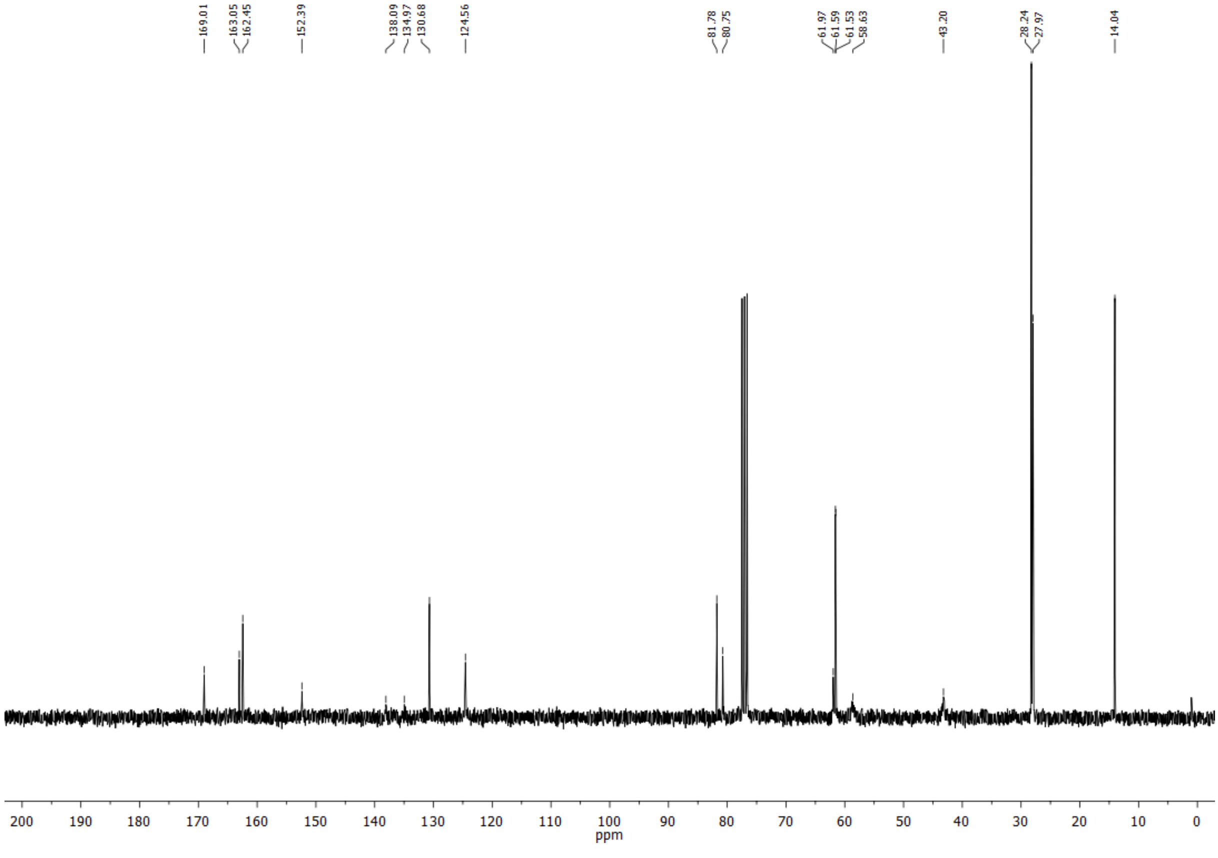

Compound **7c**, ^{1}H NMR and ^{13}C NMR ($CDCl_3$):

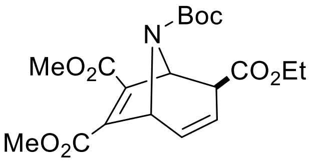

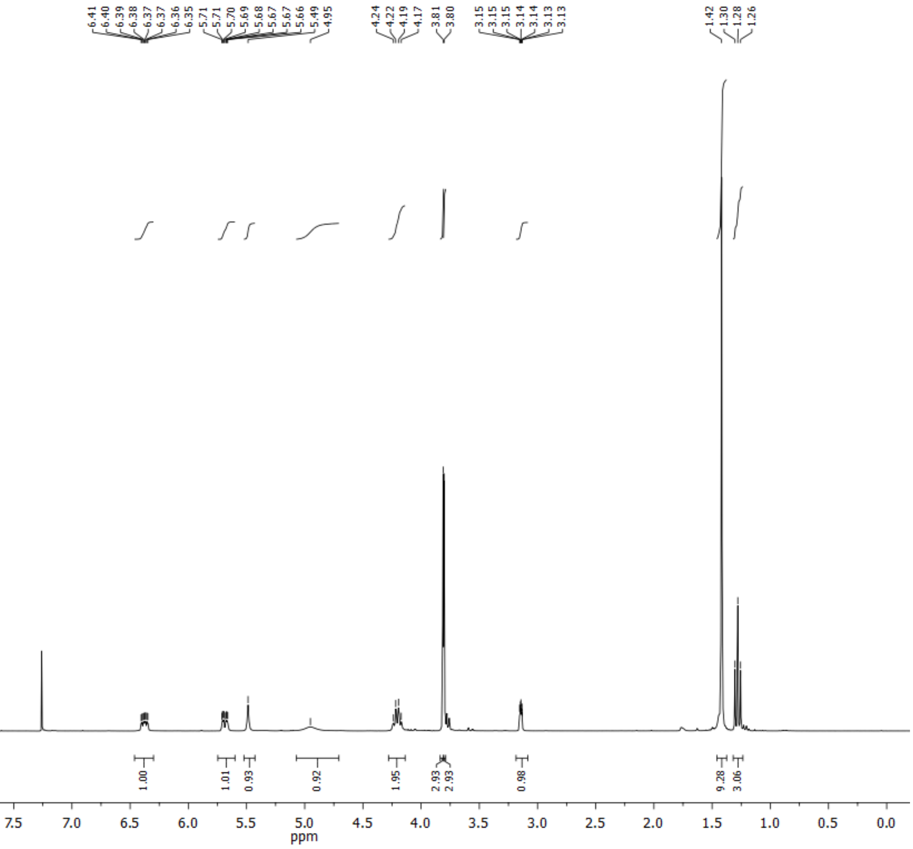

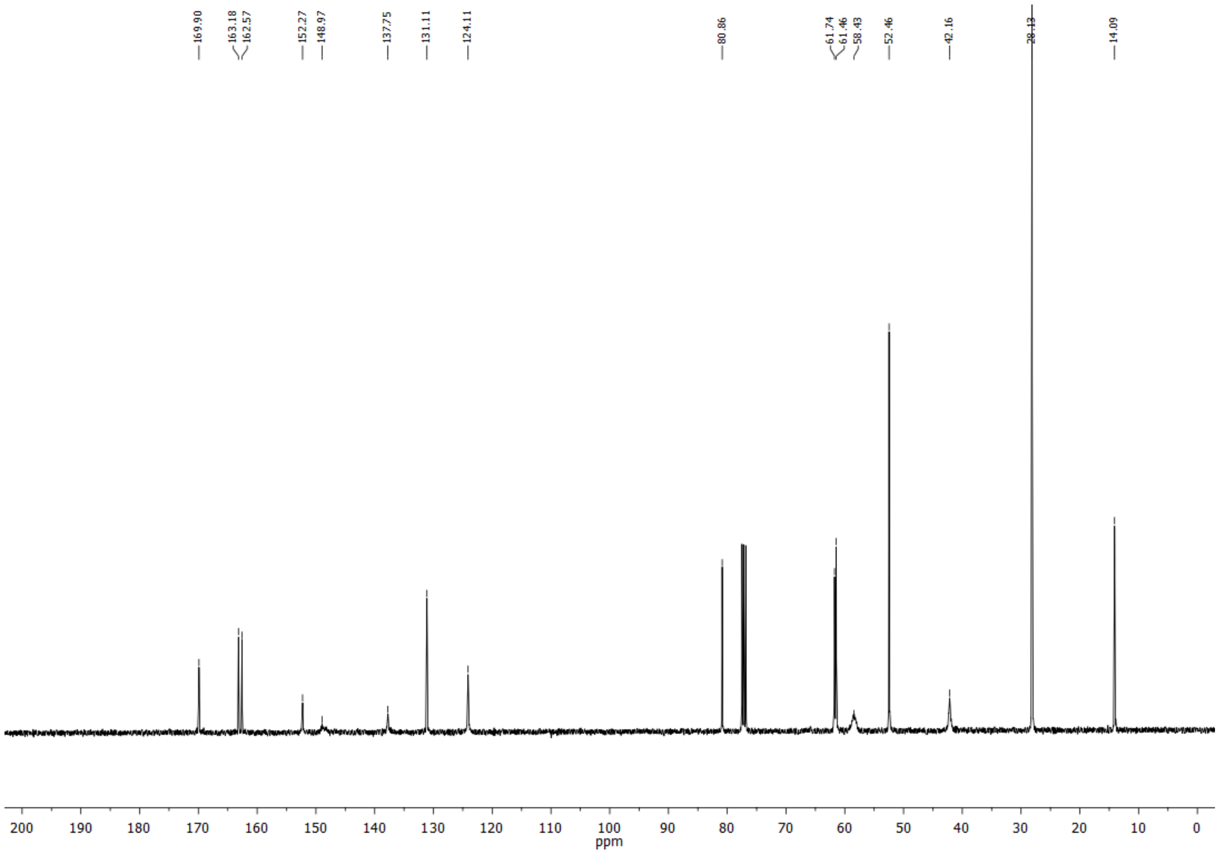

Compound **7d**, ^{1}H NMR and ^{13}C NMR ($CDCl_3$):

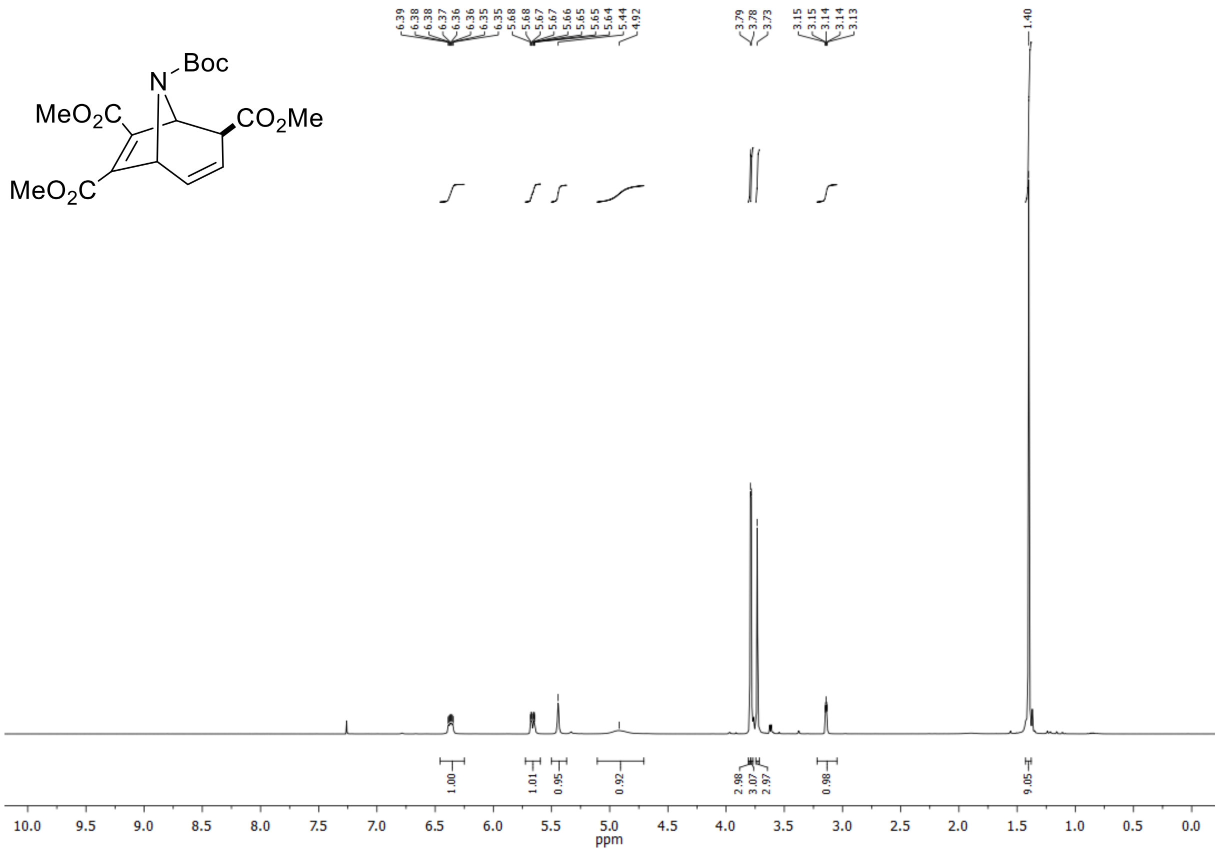

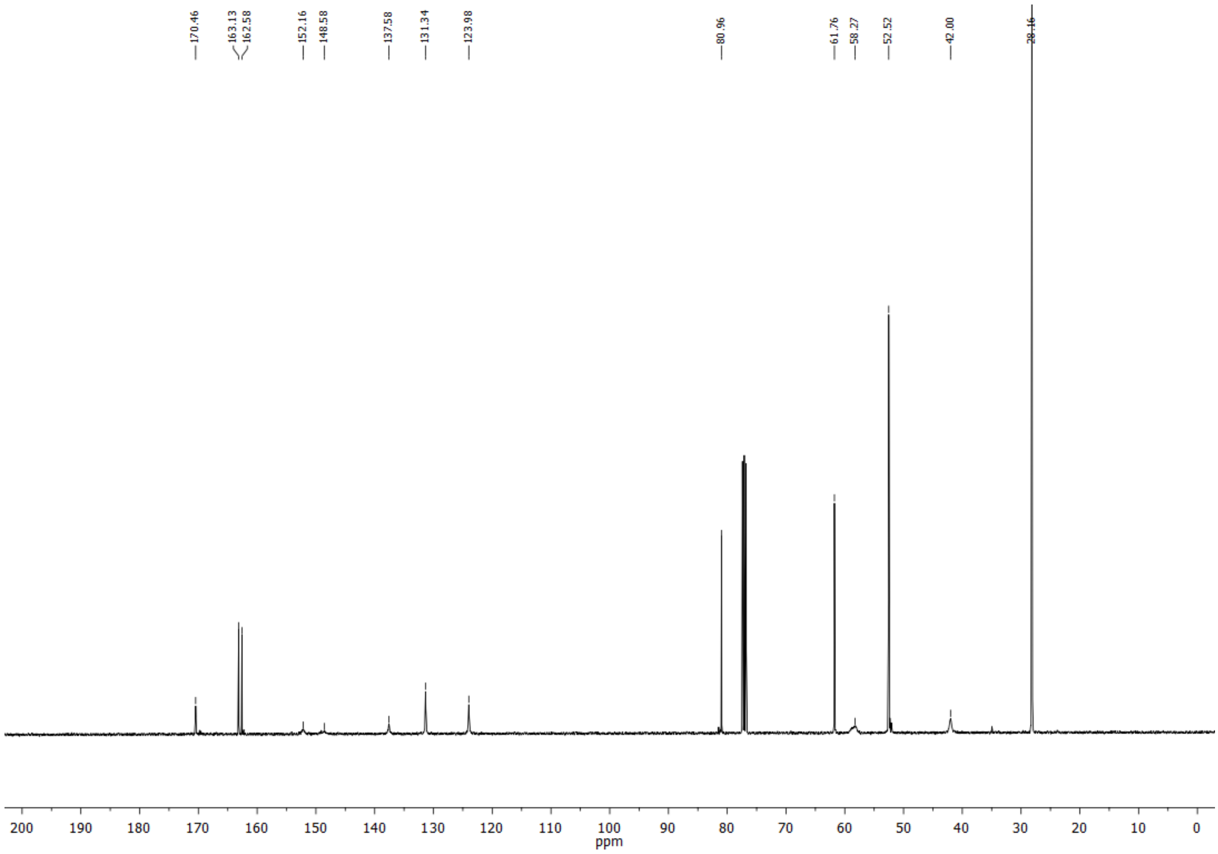

Compound **7e**, ^{1}H NMR and ^{13}C NMR ($CDCl_3$):

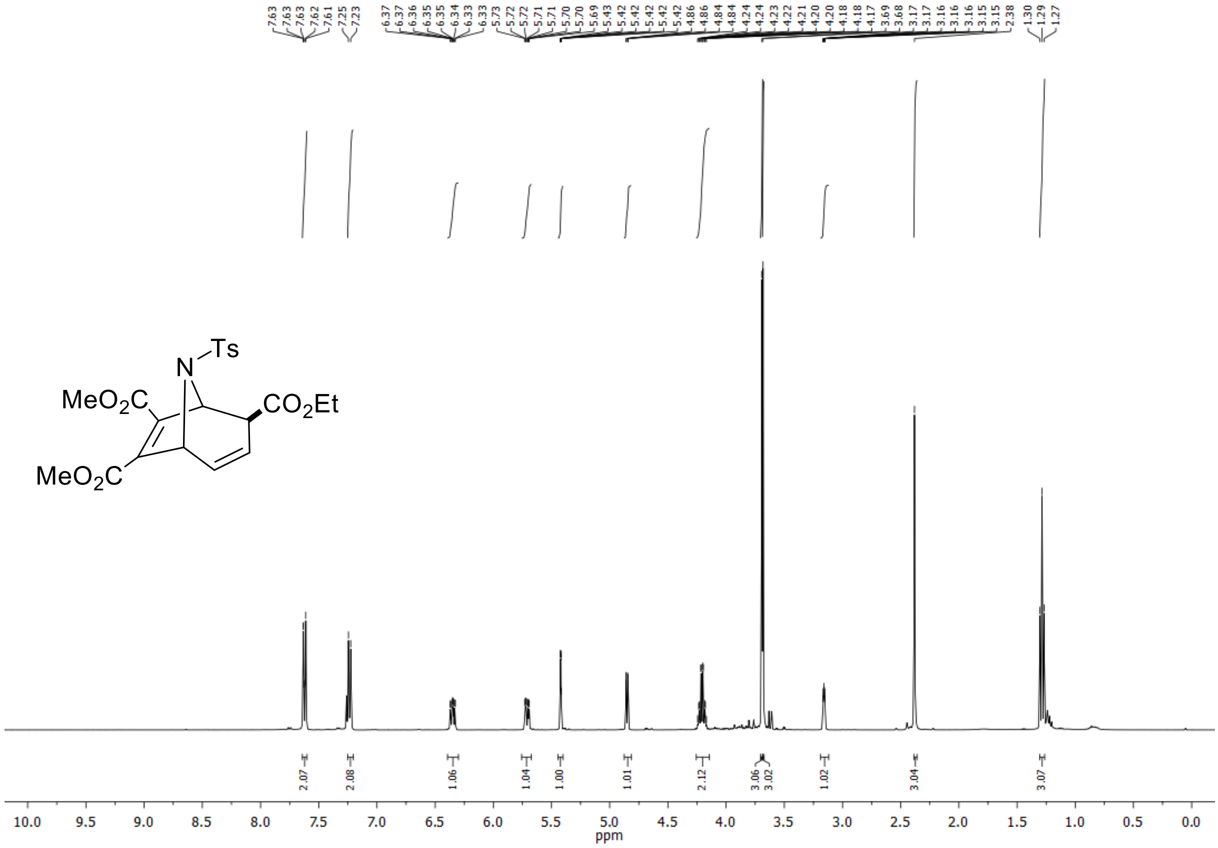

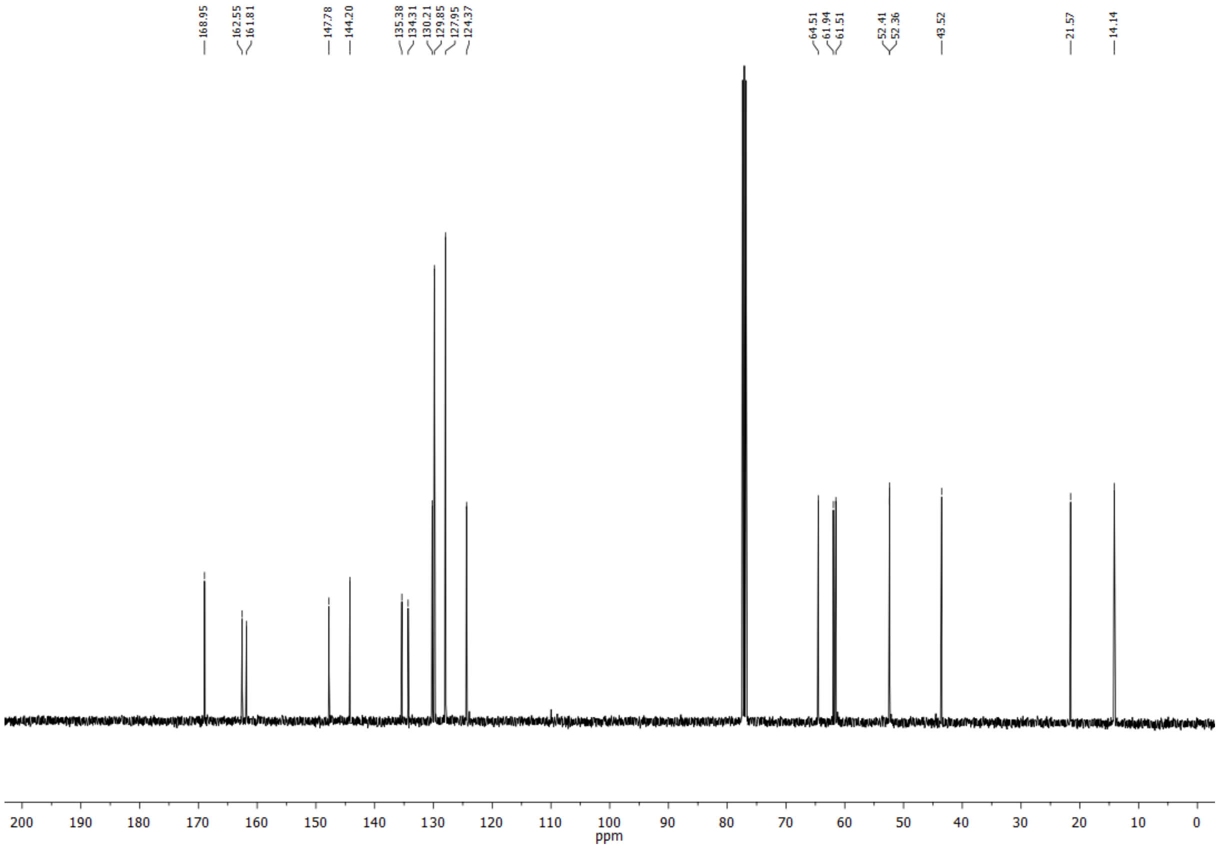

Compound **7f**, ^{1}H NMR and ^{13}C NMR ($CDCl_3$):

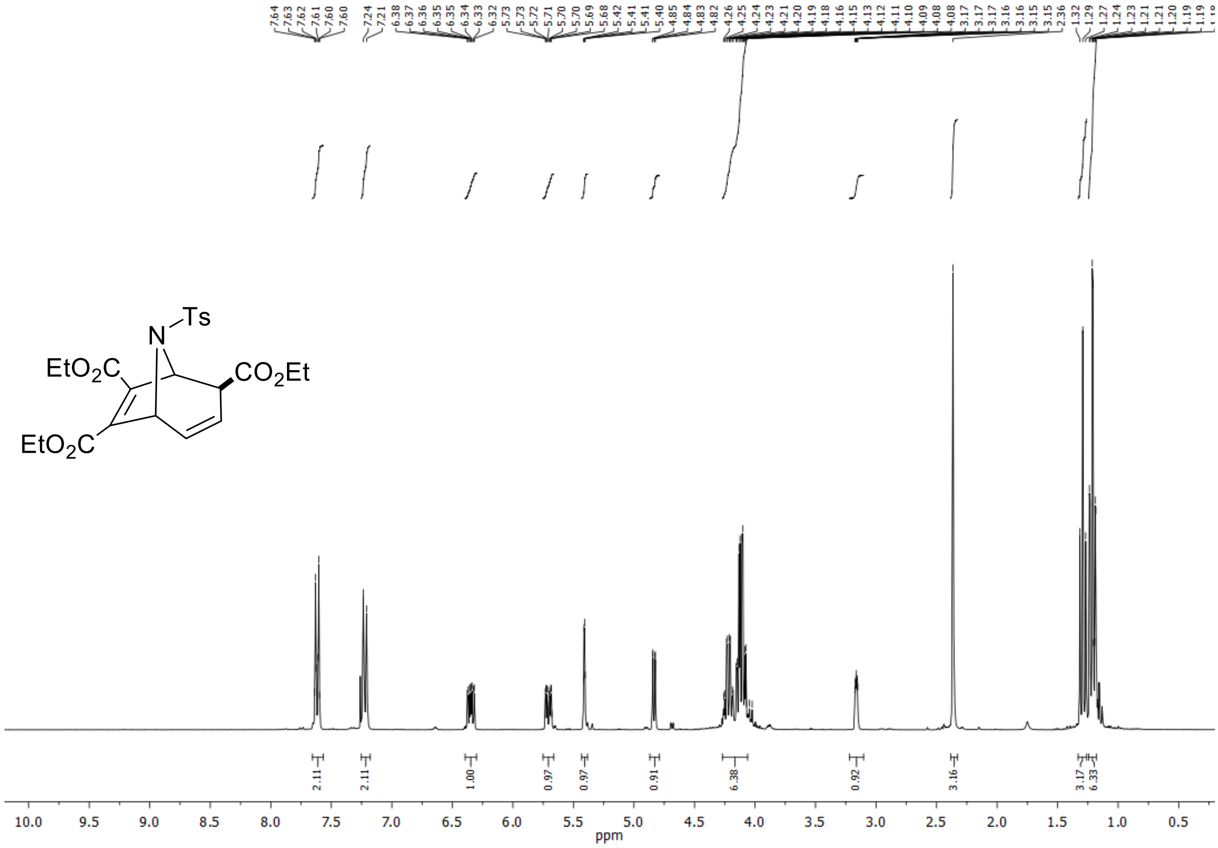

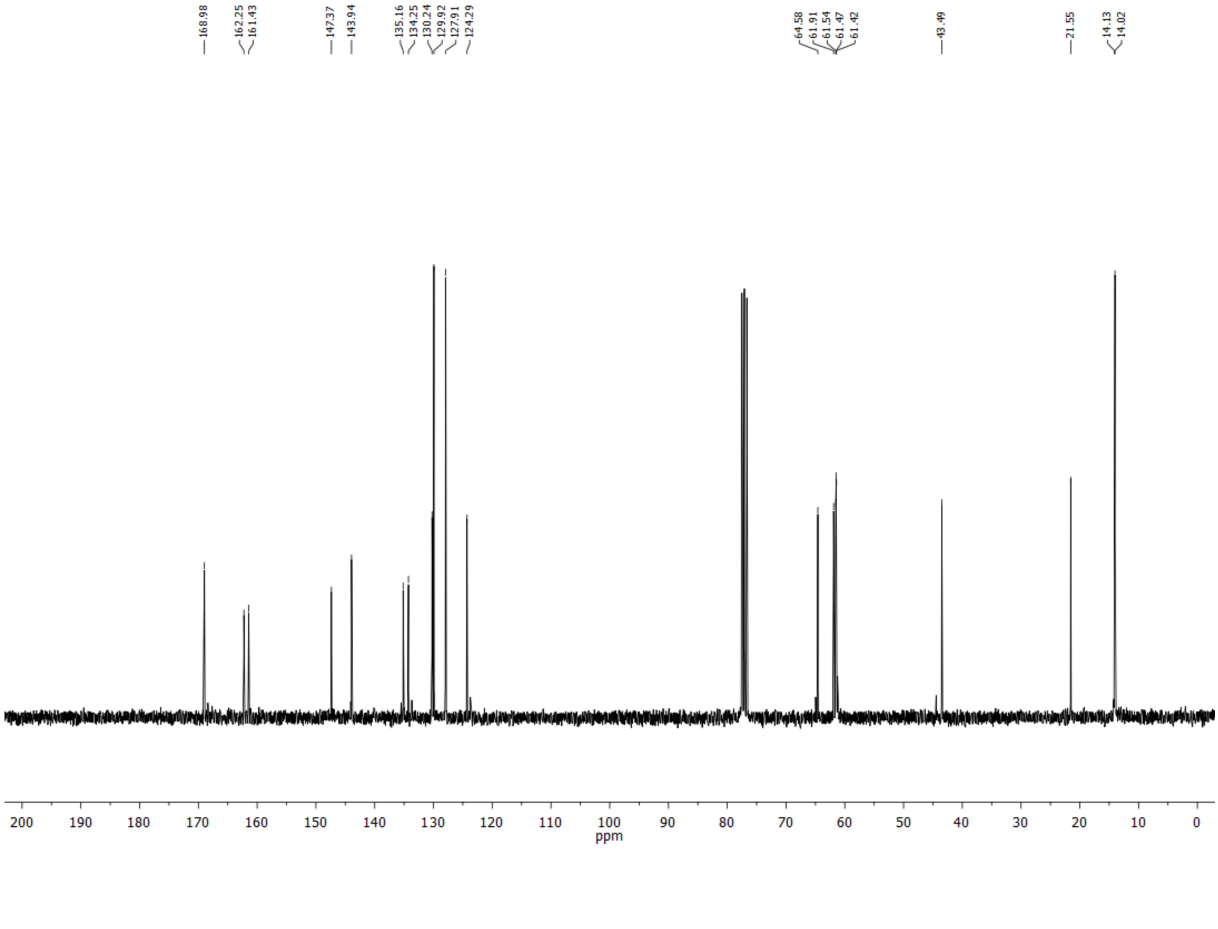

Compound **7g**, ^{1}H NMR and ^{13}C NMR ($CDCl_3$):

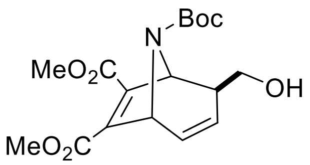

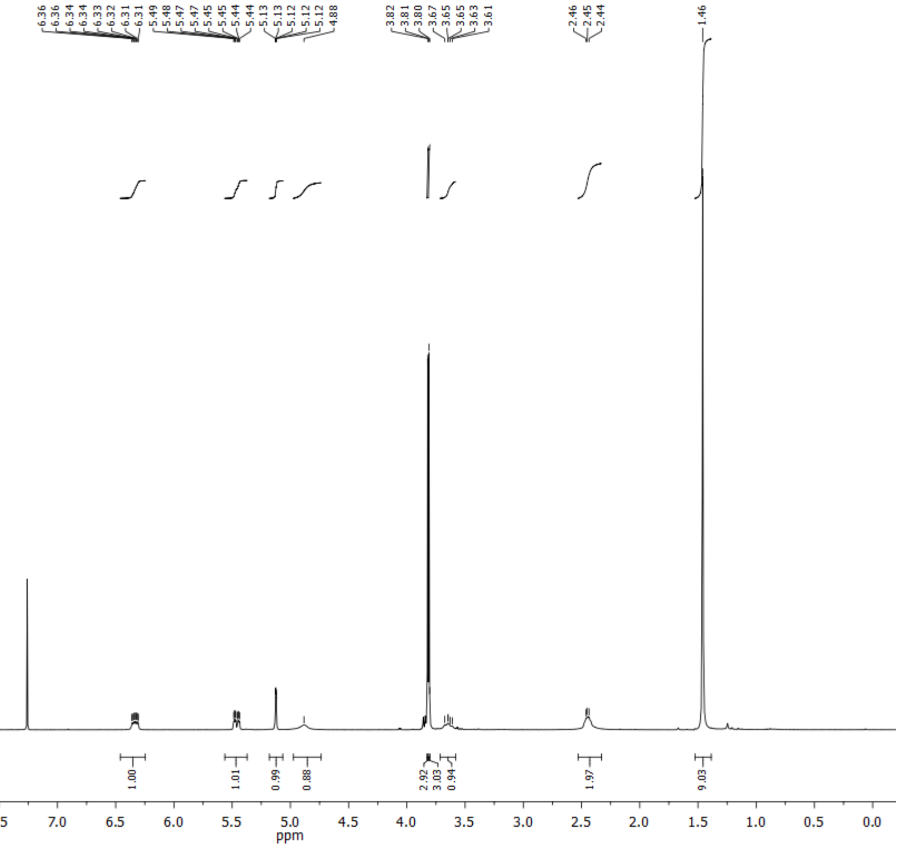

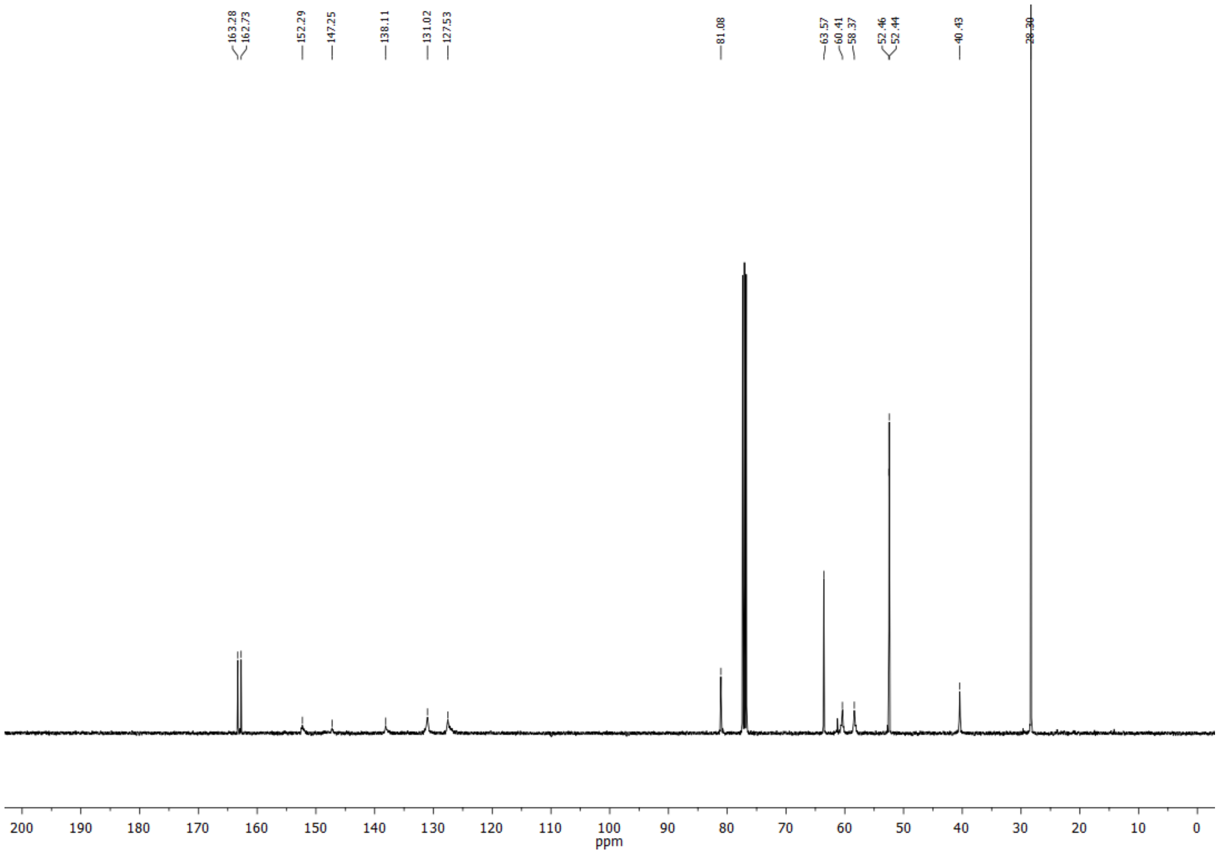

Compound **7h**, ^{1}H NMR and ^{13}C NMR ($CDCl_3$):

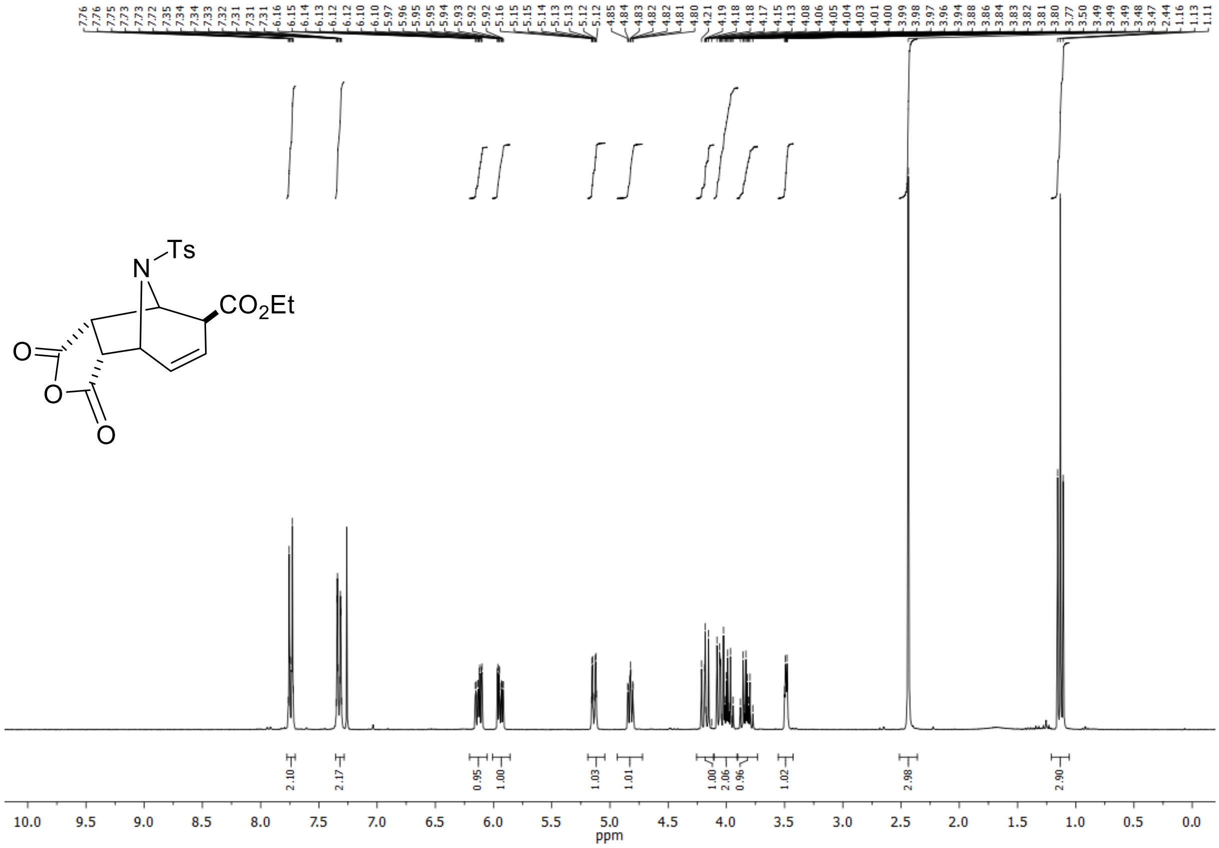

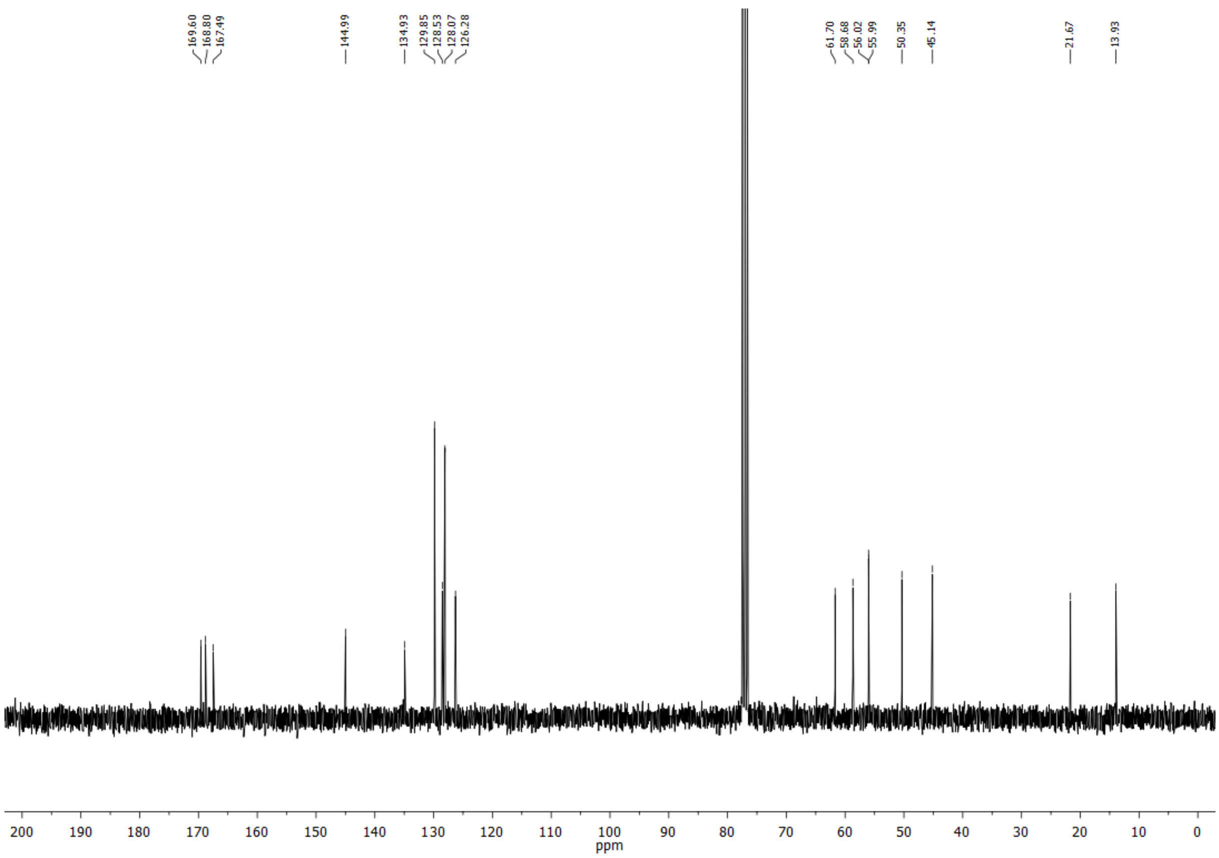

Compound **7i**, ^{1}H NMR and ^{13}C NMR ($CDCl_3$):

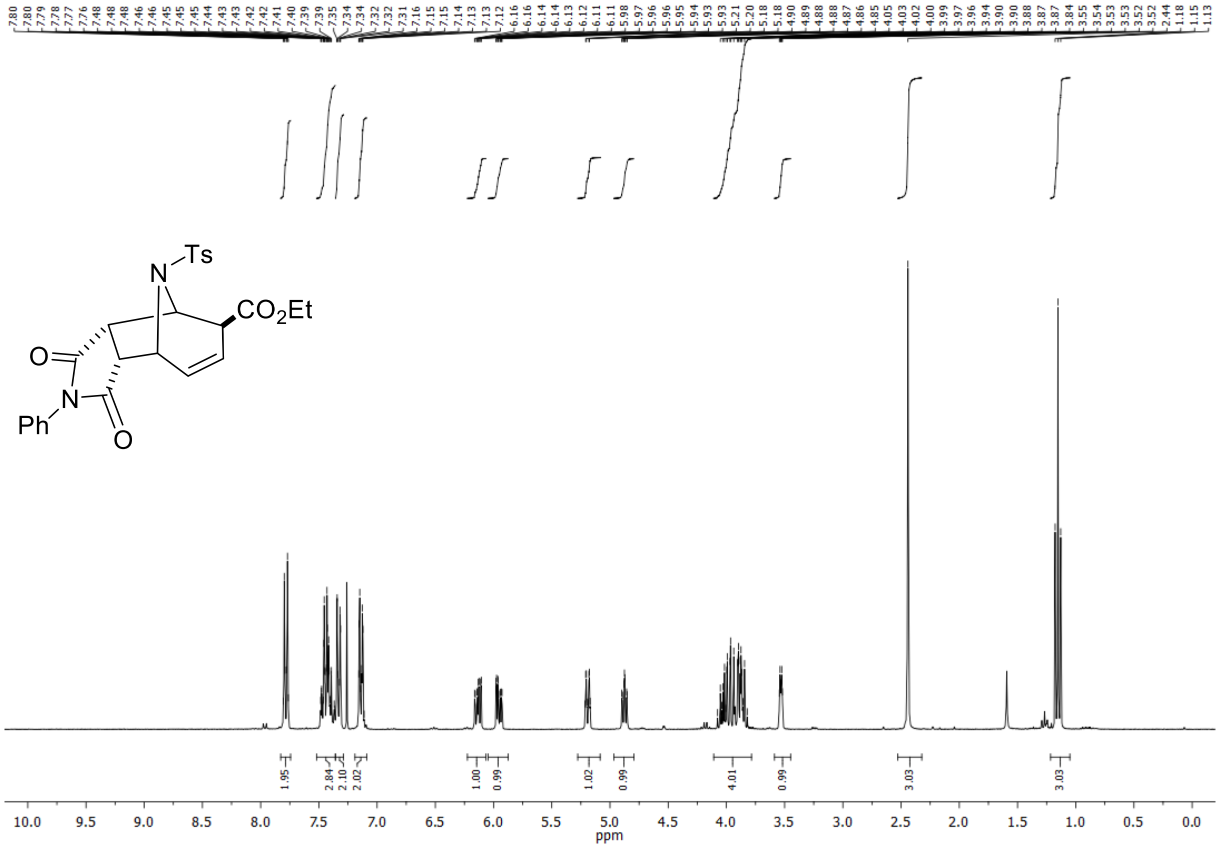

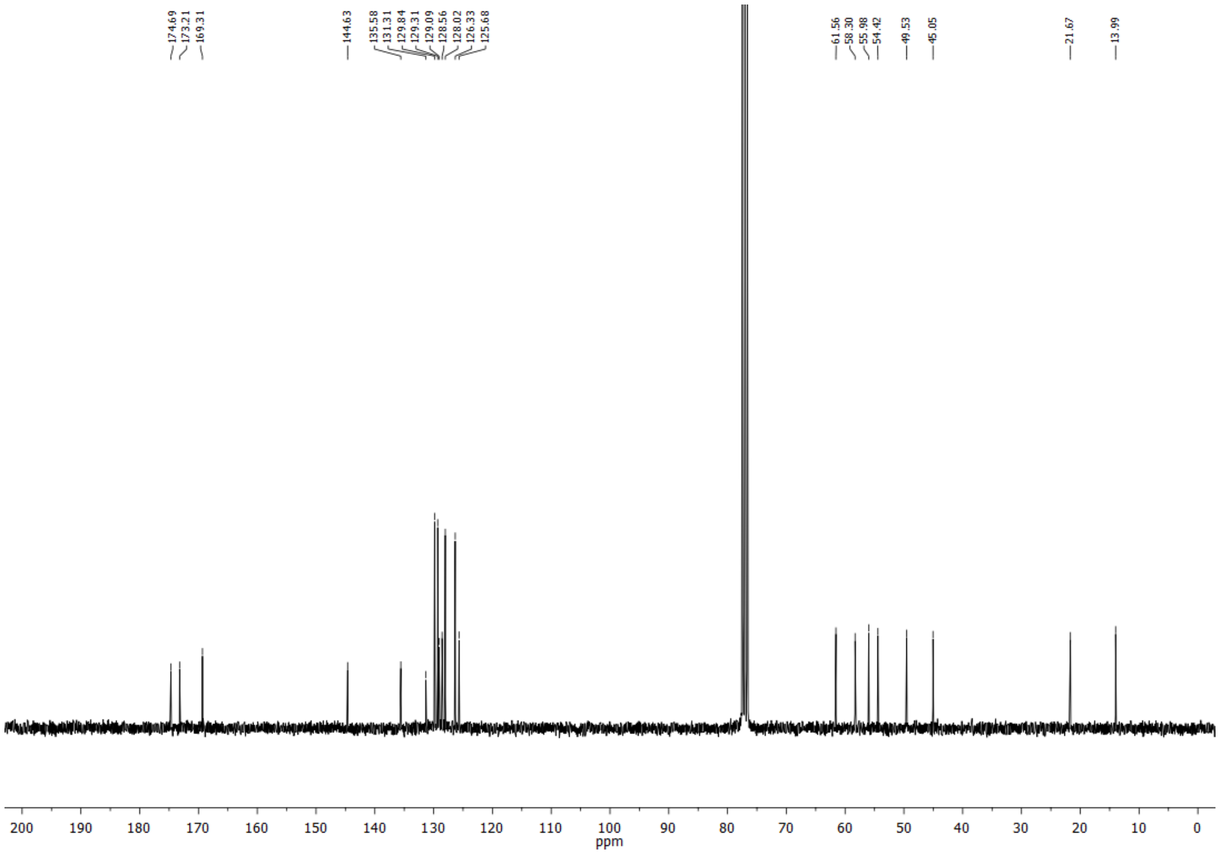

Compound *major* **7j**, ^{1}H NMR and ^{13}C NMR ($CDCl_3$):

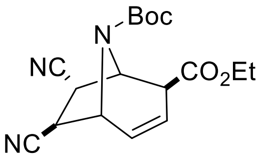

major **7j**

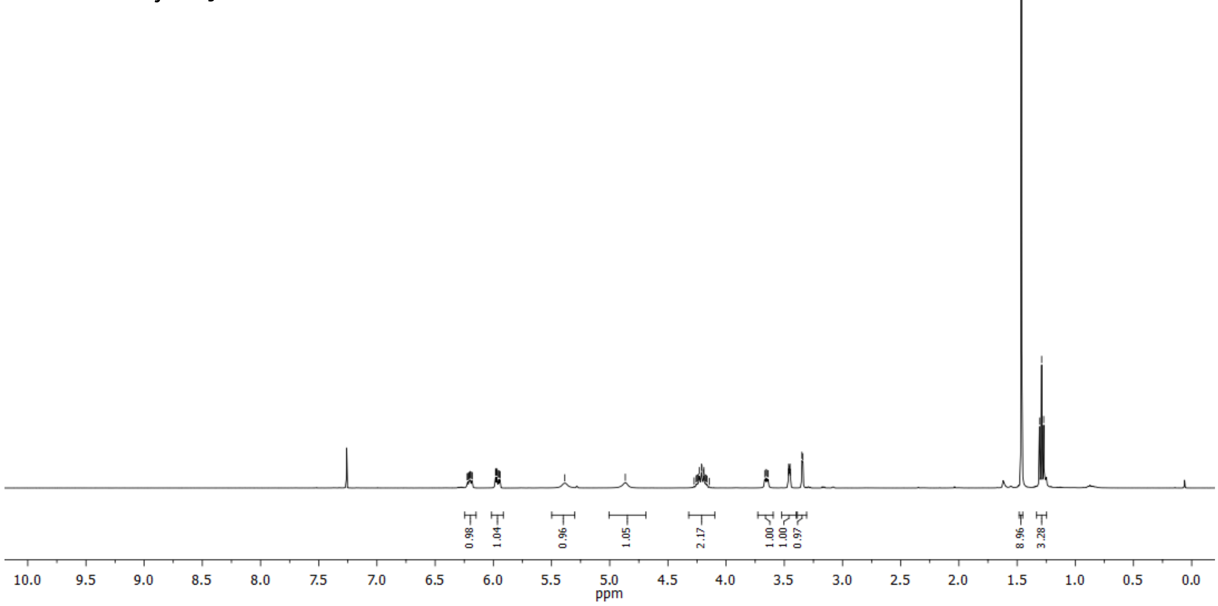

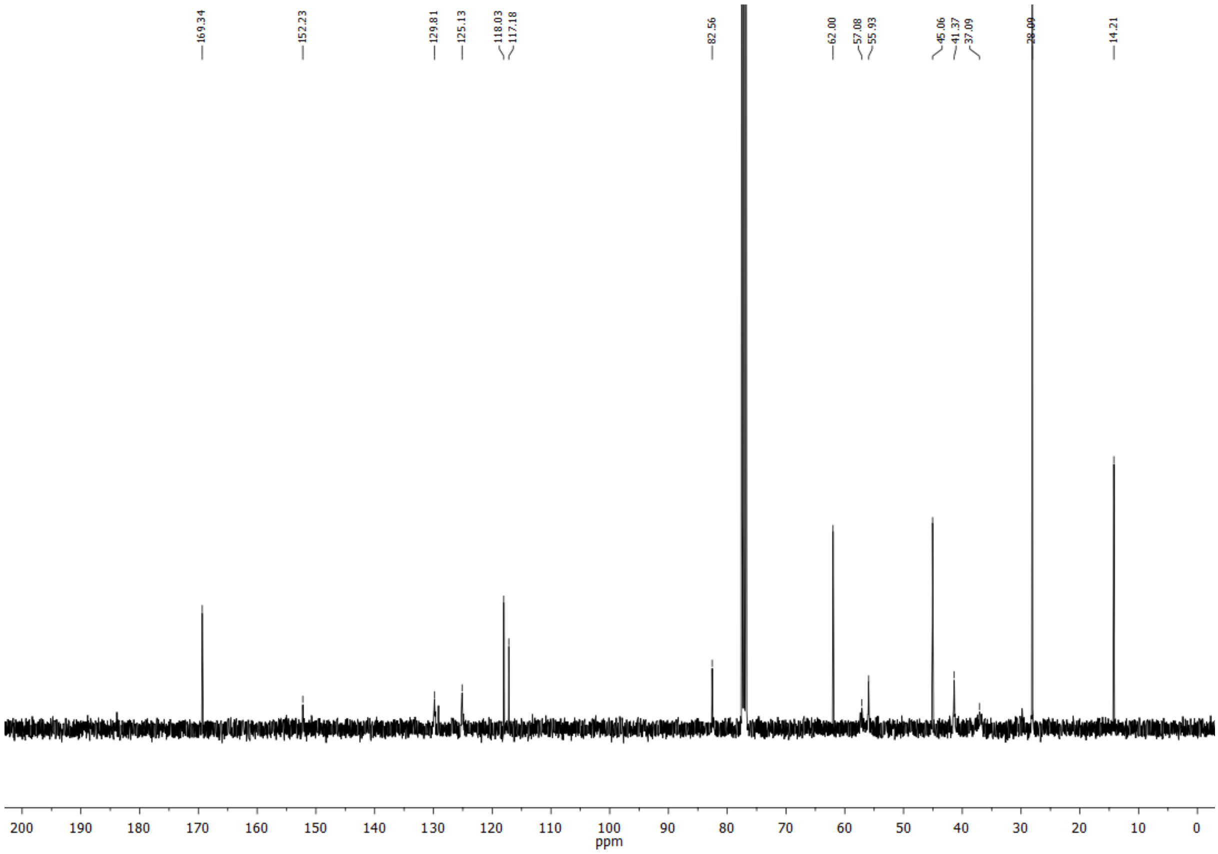

Compound *major and minor* **7j**, ^{1}H NMR and ^{13}C NMR ($CDCl_3$):

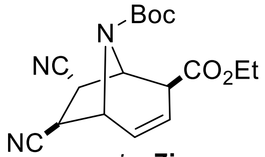

major **7j**

+

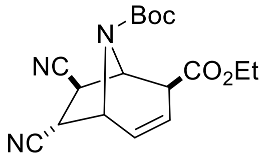

minor **7j**

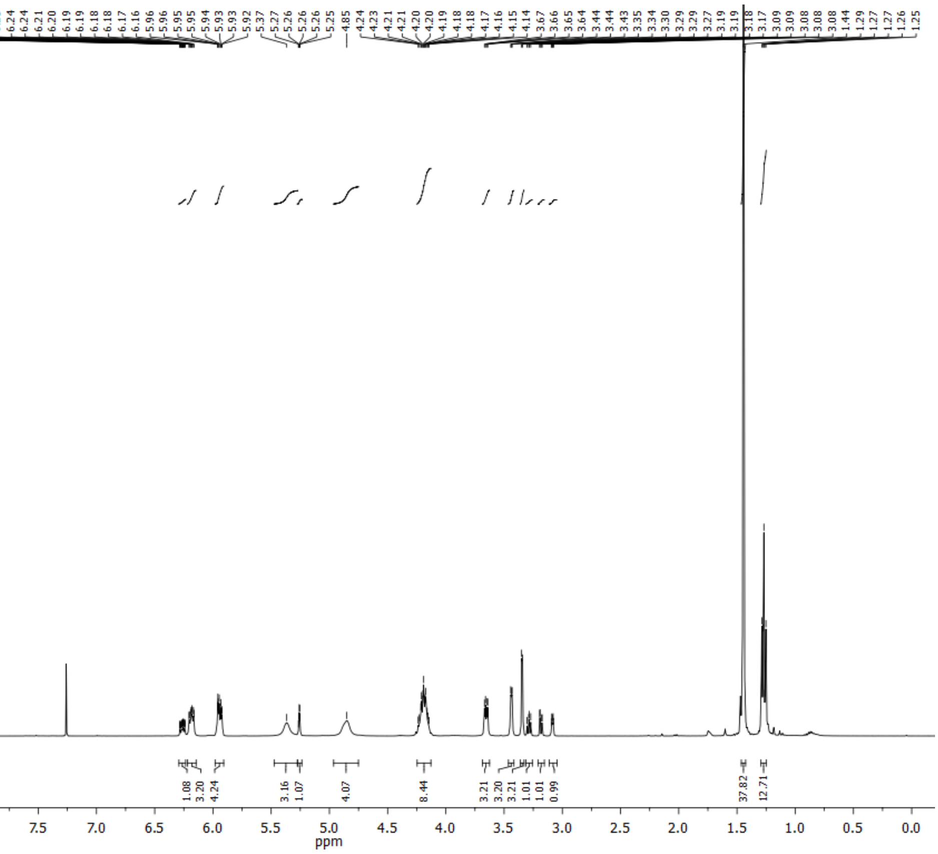

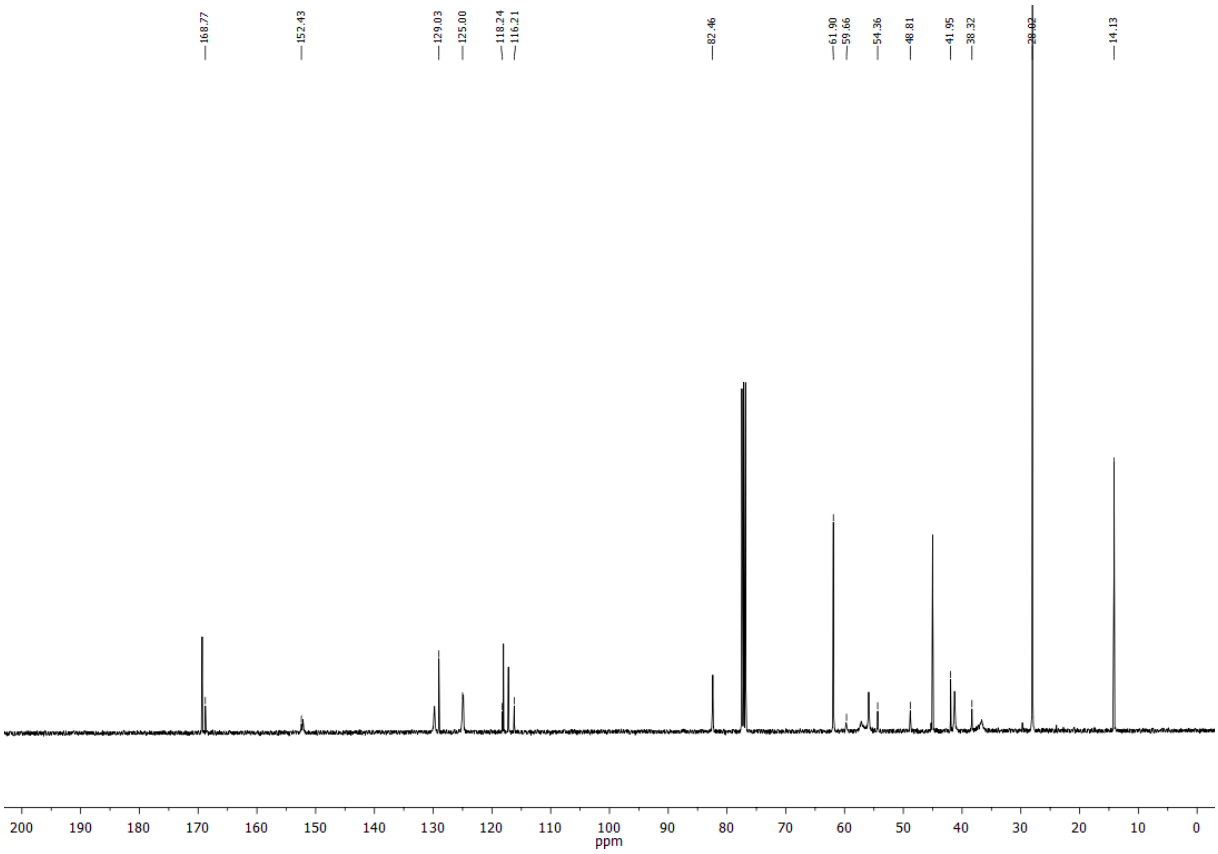

Compound **7k**, ^{1}H NMR and ^{13}C NMR ($CDCl_3$):

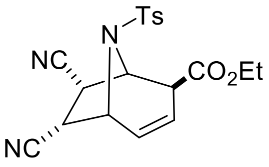

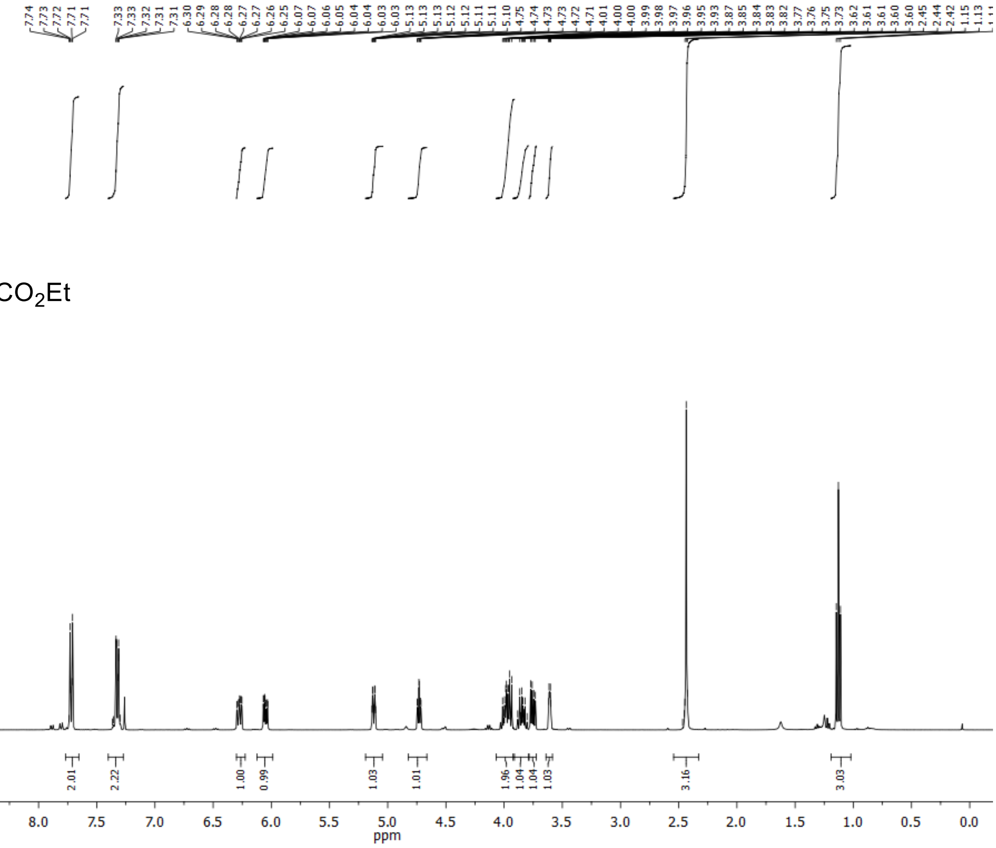

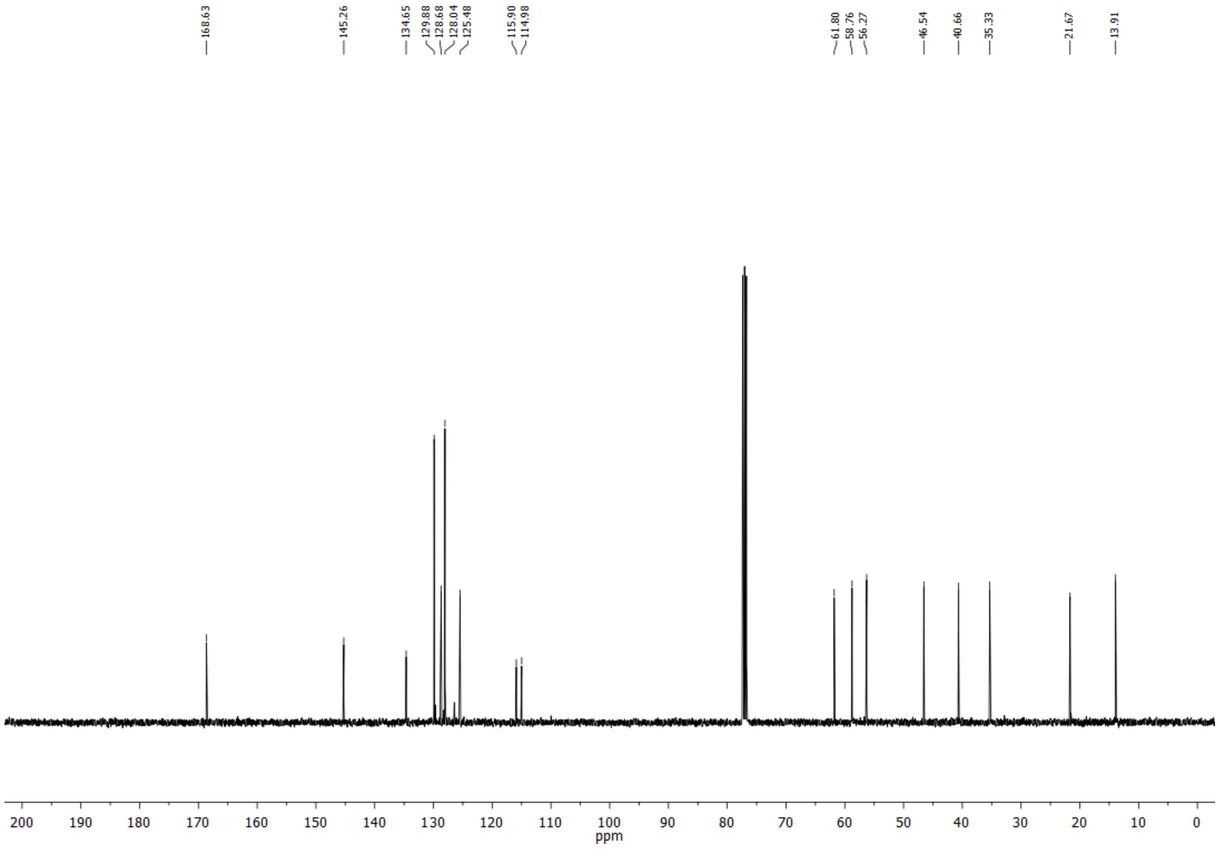

Compound ***endo*** (–)-**7l**, ^{1}H NMR and ^{13}C NMR ($CDCl_3$):

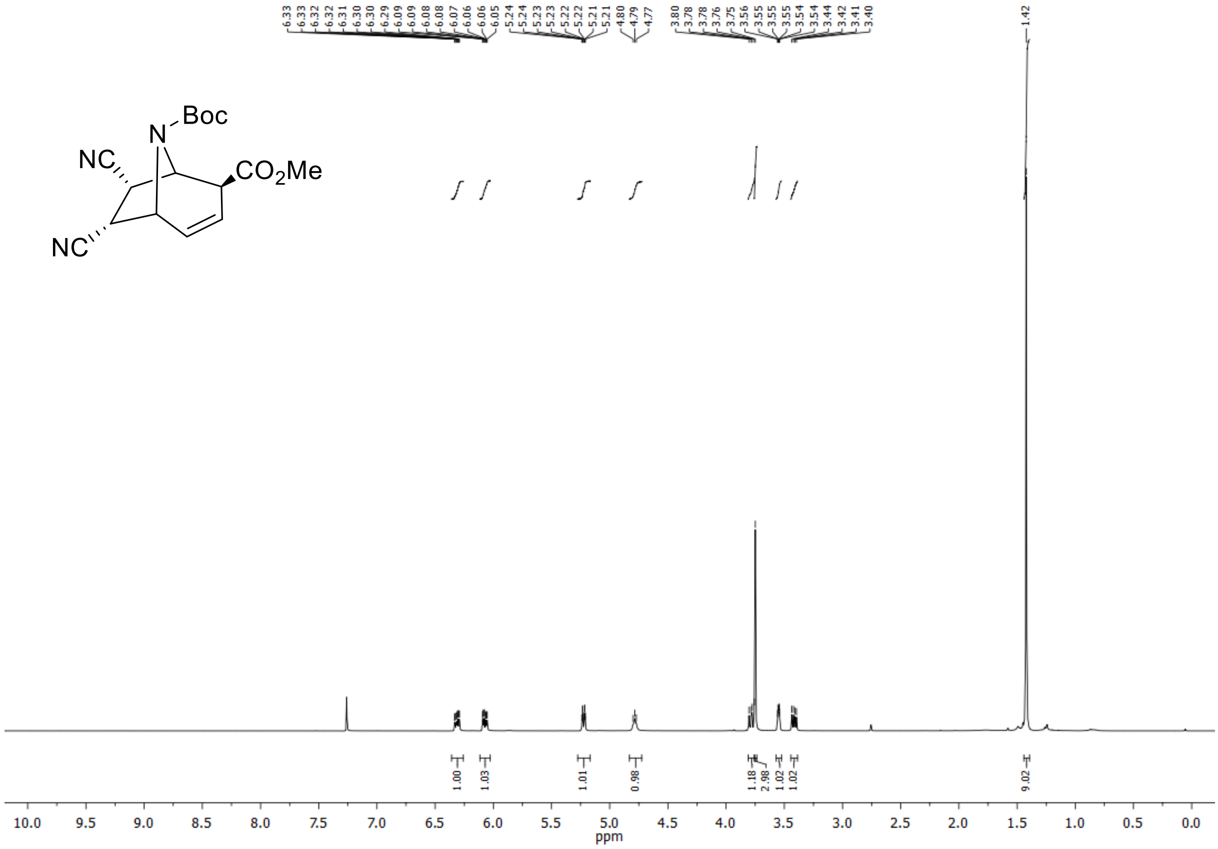

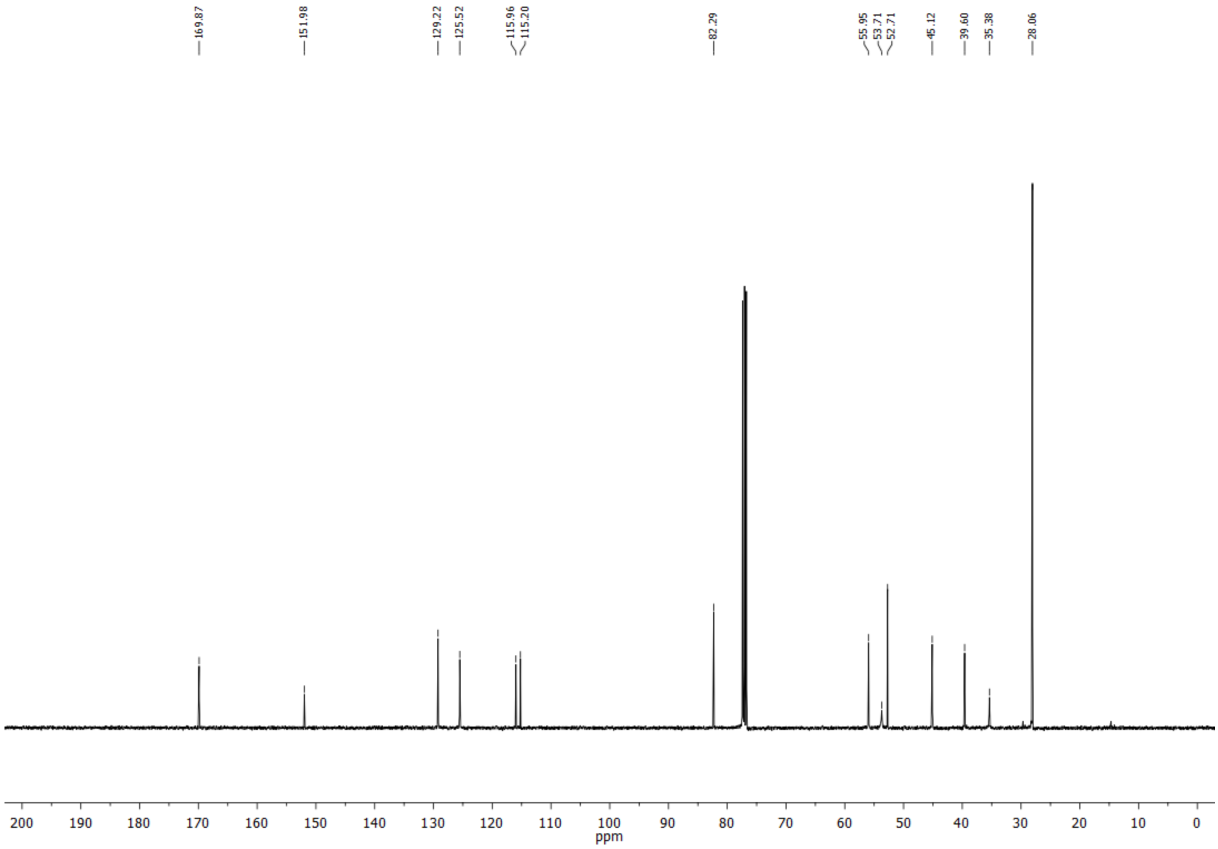

Compound ***exo*** (–)-**7l**, ^{1}H NMR and ^{13}C NMR ($CDCl_3$):

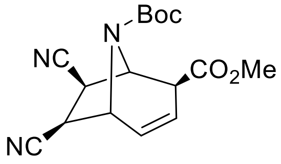

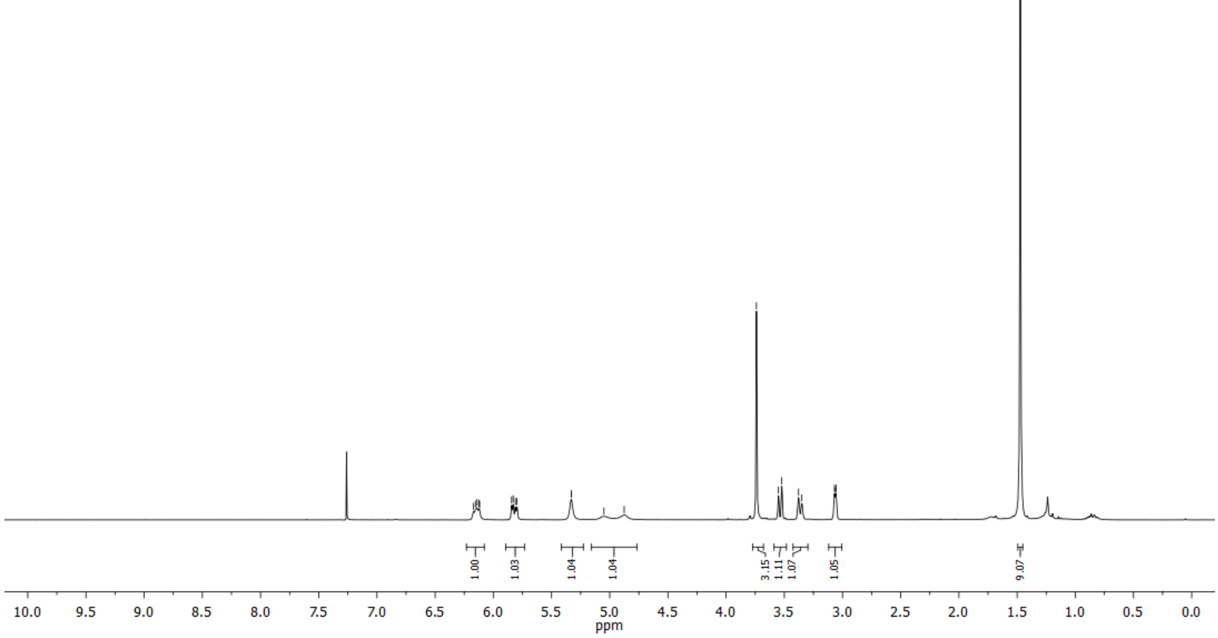

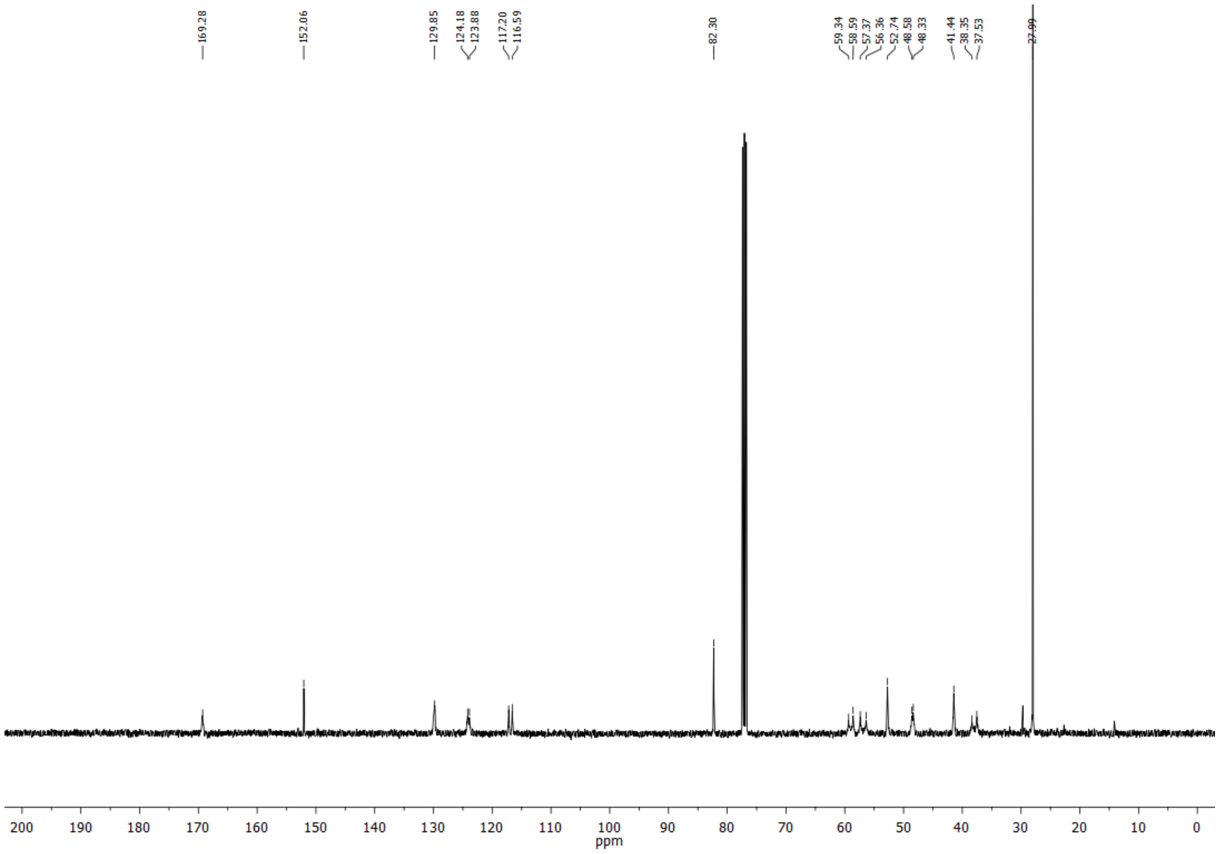

Compound *major* **7m**, ^{1}H NMR and ^{13}C NMR ($CDCl_3$):

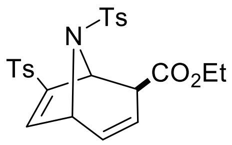

major **7m**

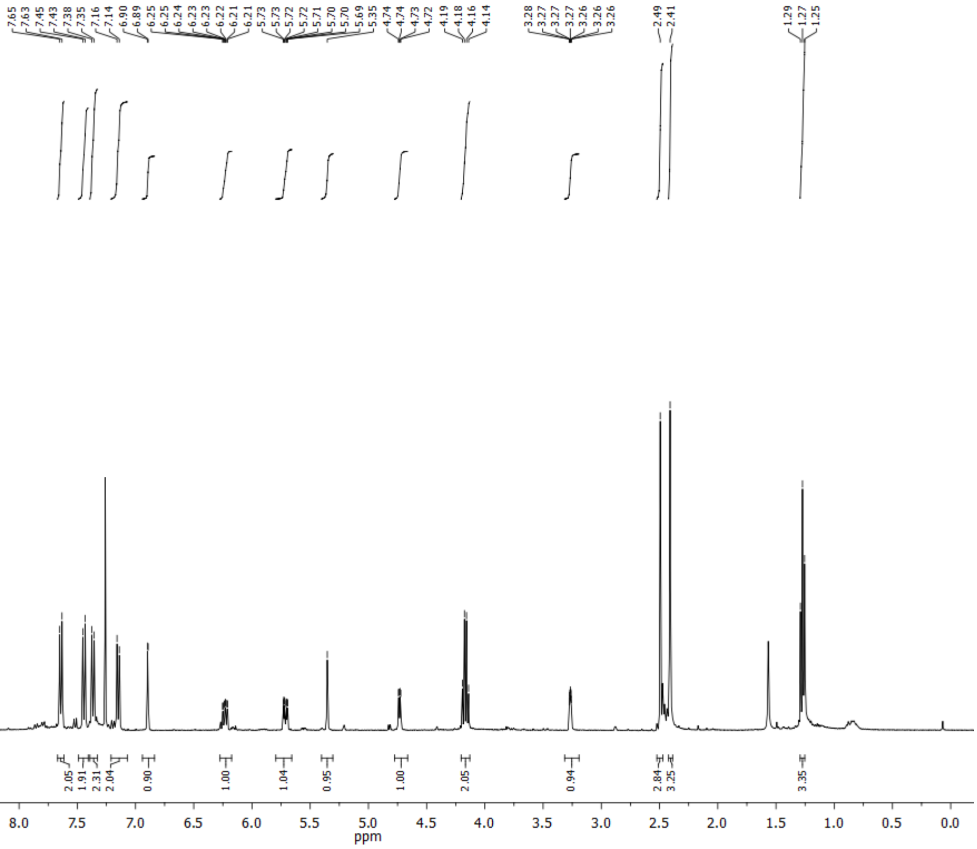

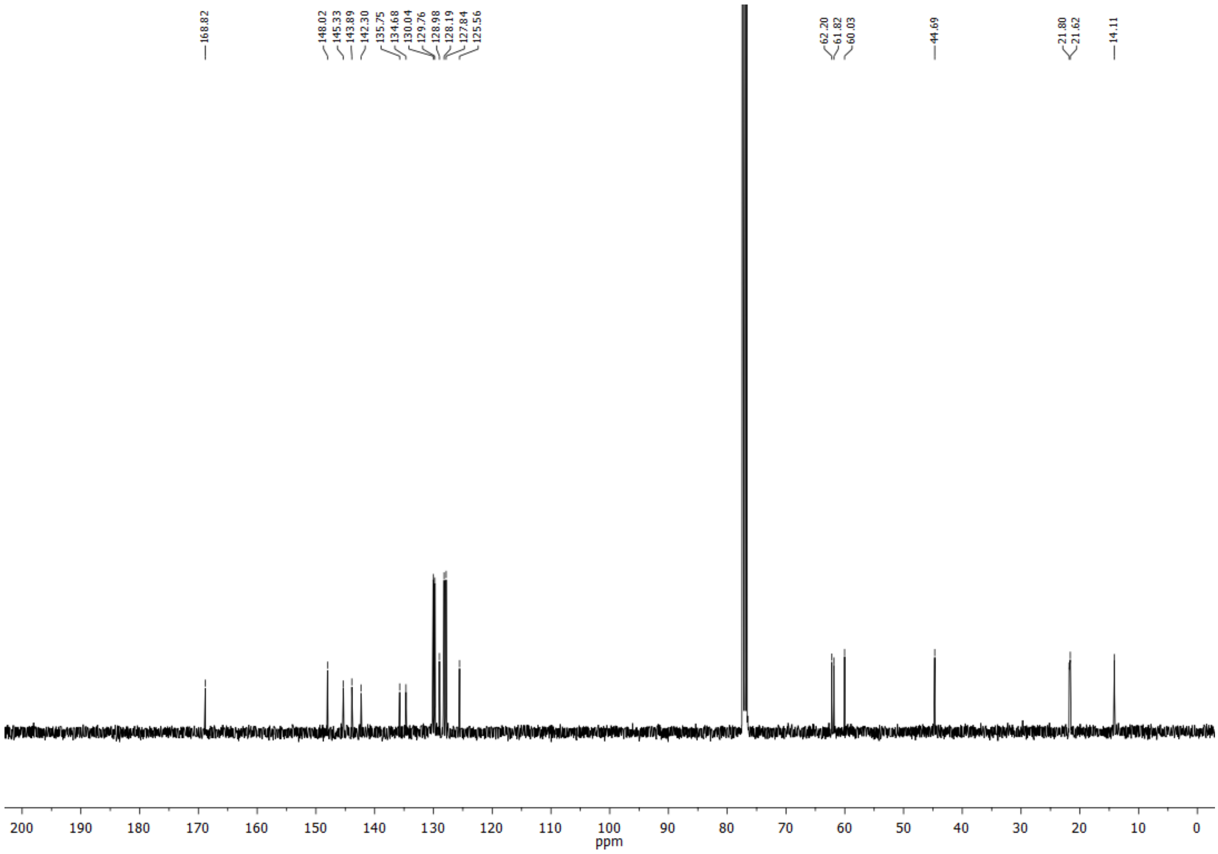

Compound *major* and *minor* **7m**, ^{1}H NMR and ^{13}C NMR ($CDCl_3$):

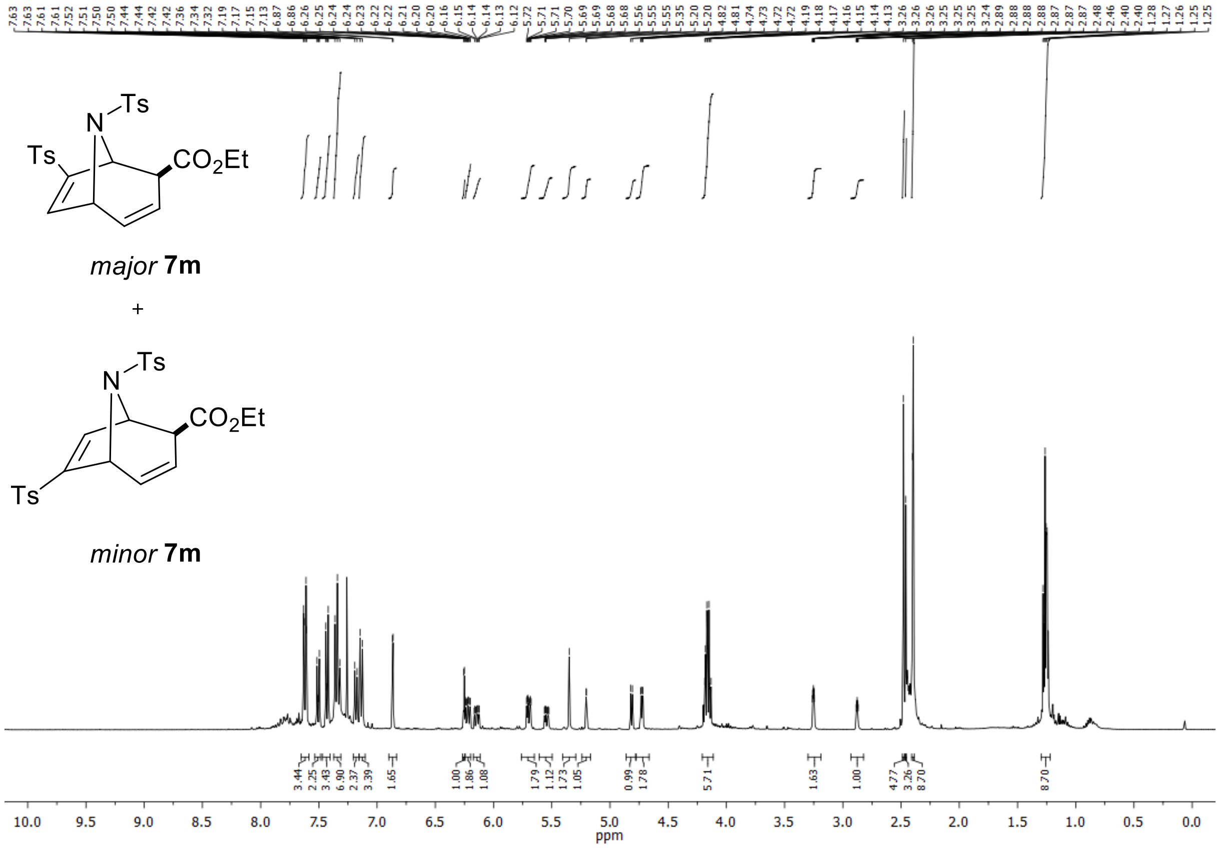

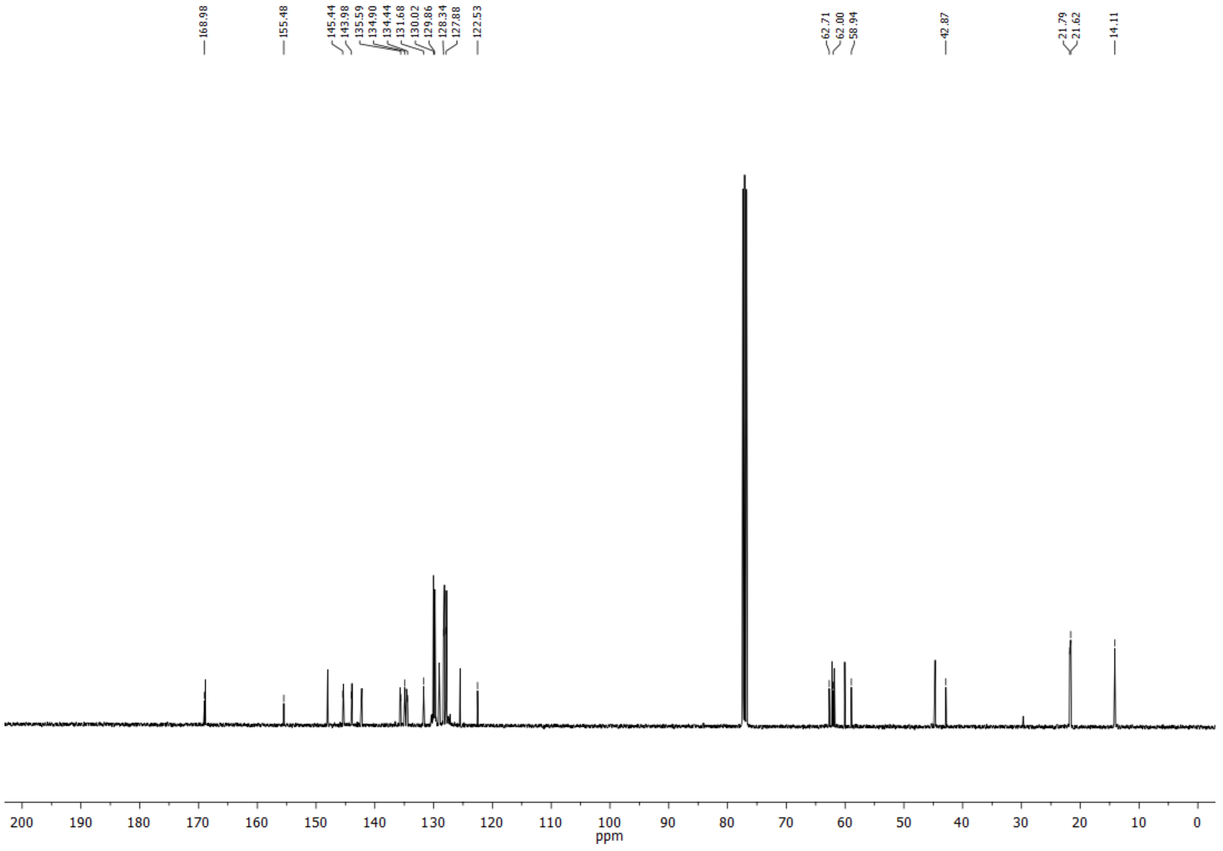

Compound **(+)-8a**, ^{1}H NMR and ^{13}C NMR ($CDCl_3$):

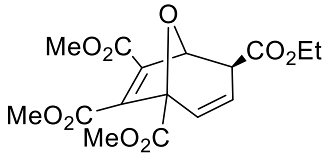

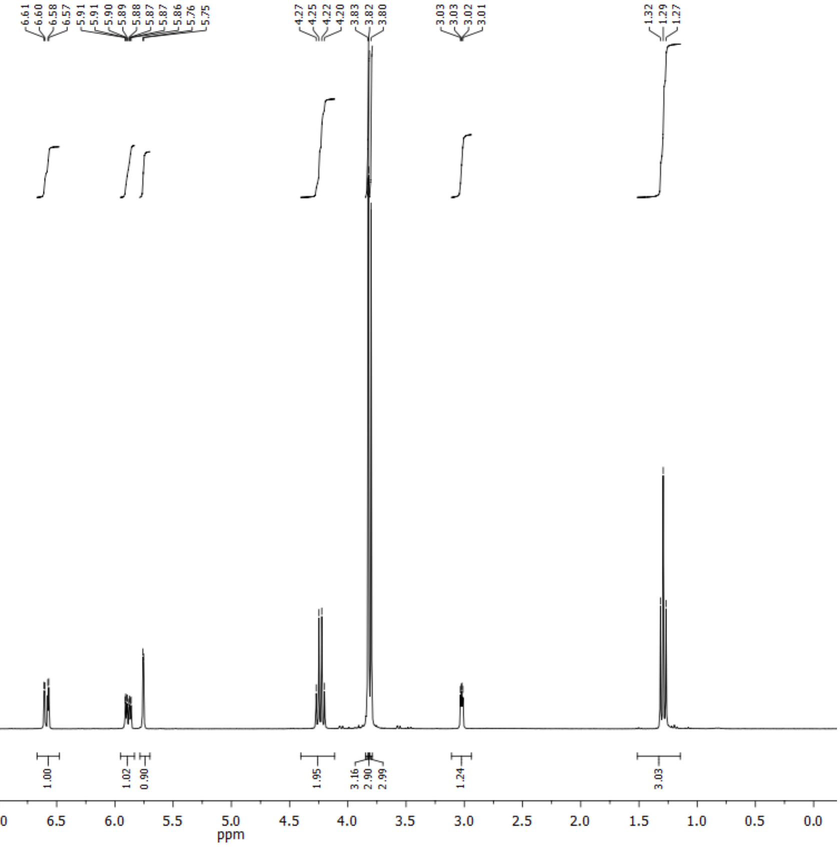

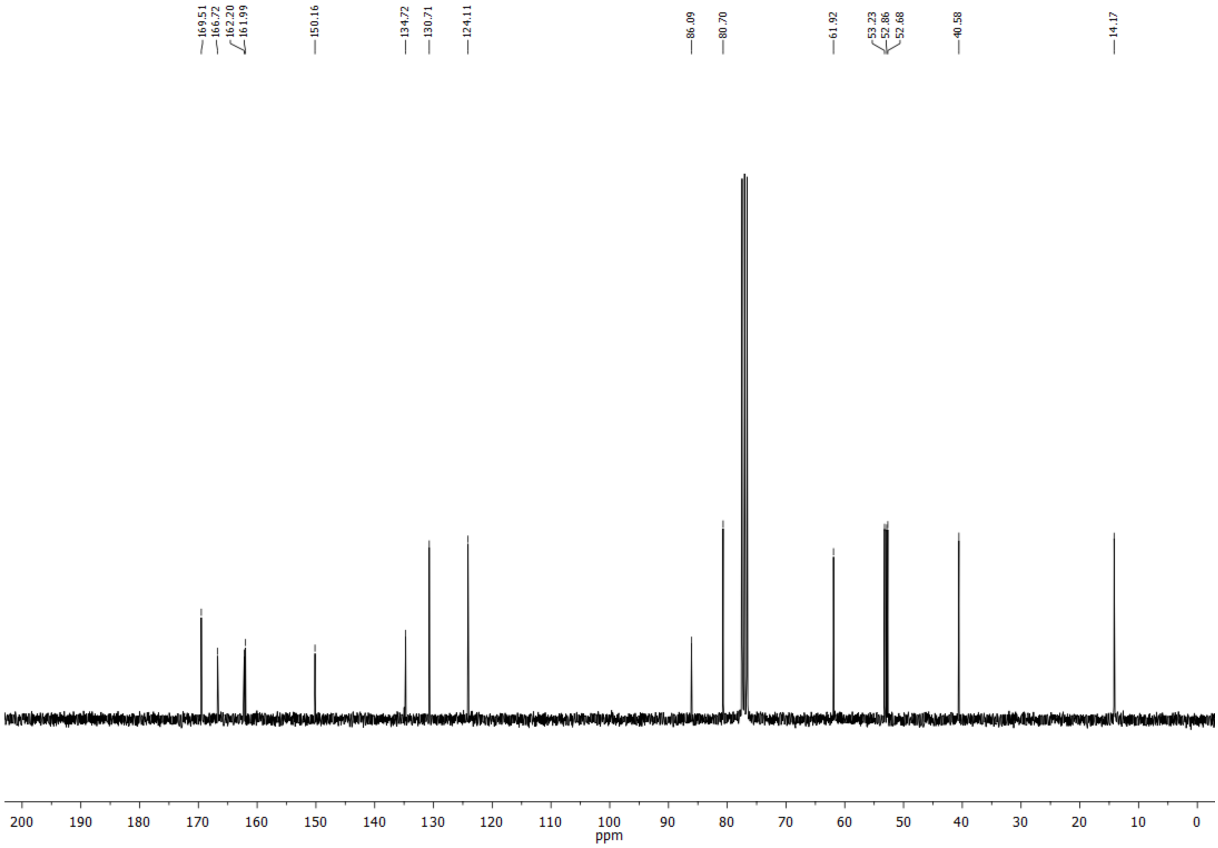

Compound **8b**, ^1H NMR and ^{13}C NMR ($CDCl_3$):

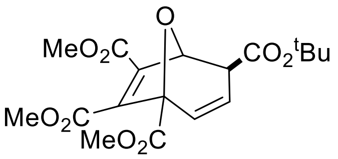

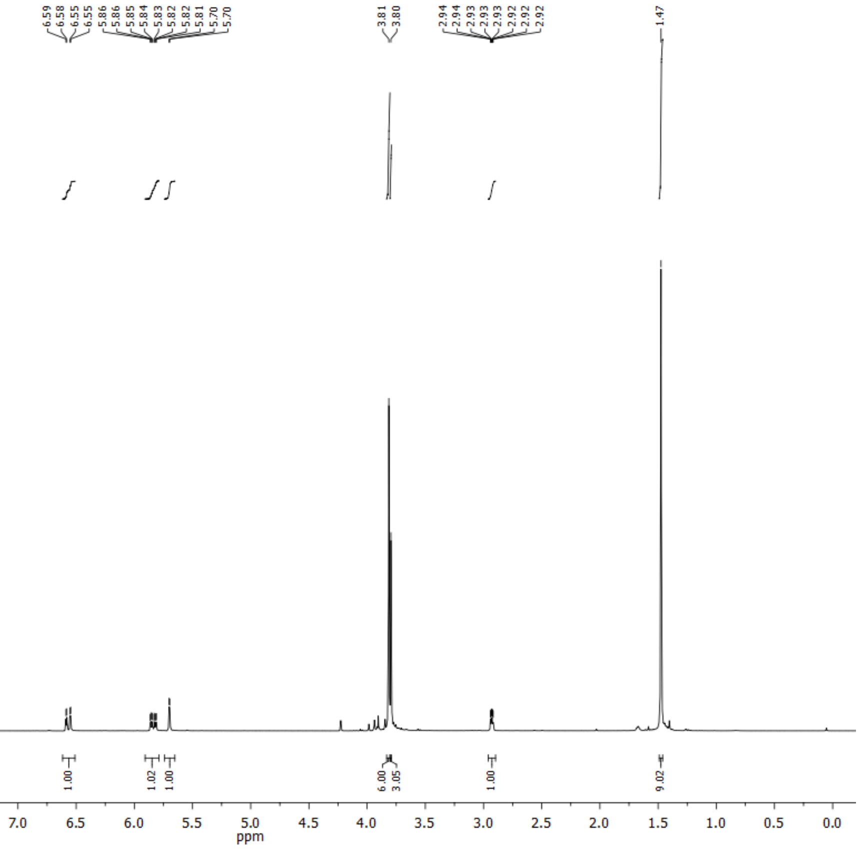

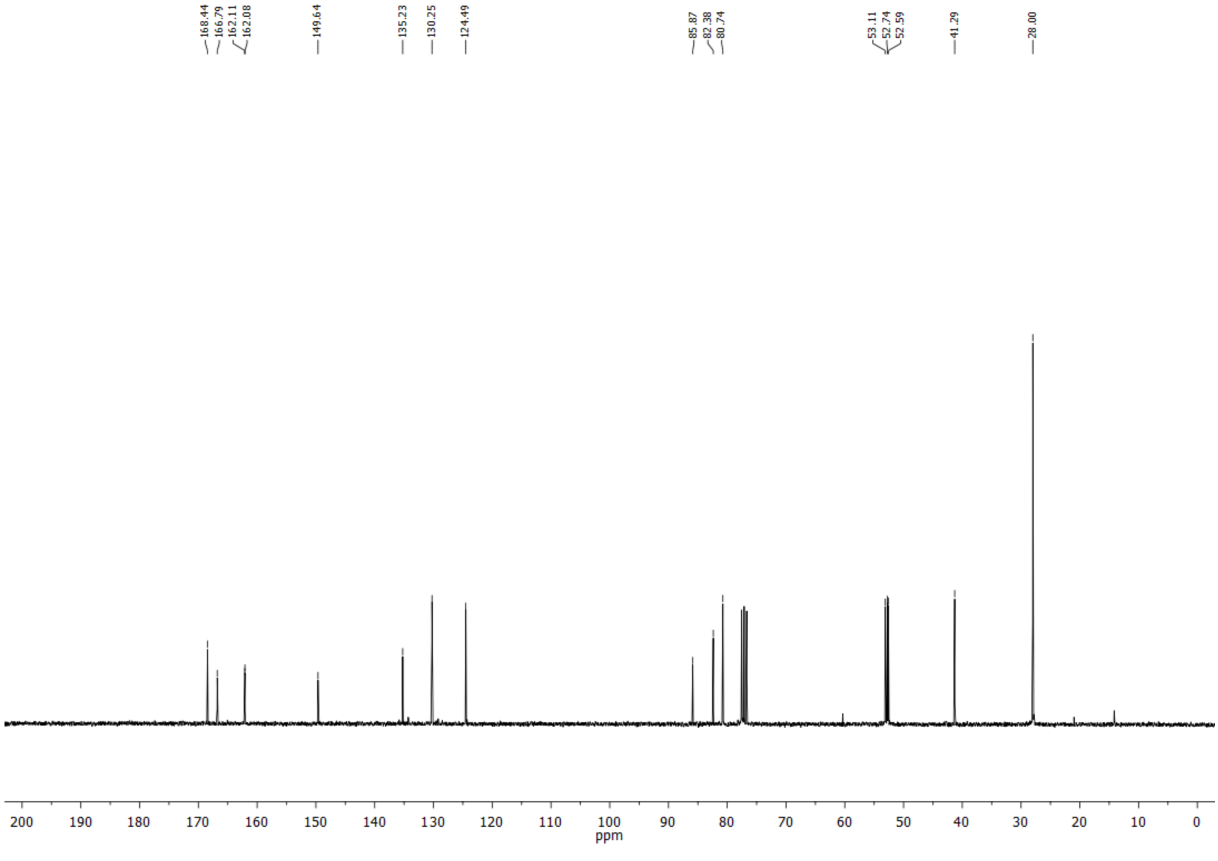

Compound *major* **8c**, ^{1}H NMR and ^{13}C NMR ($CDCl_3$):

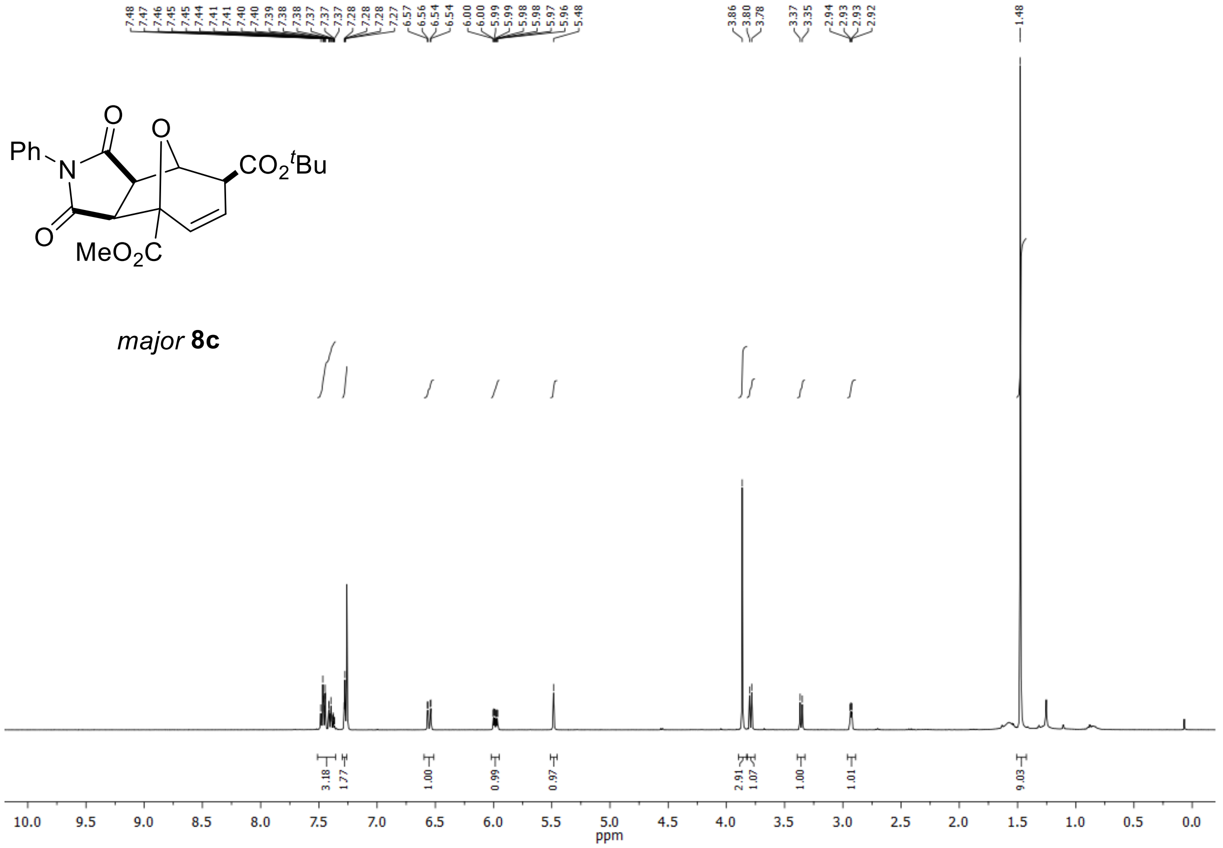

major **8c**

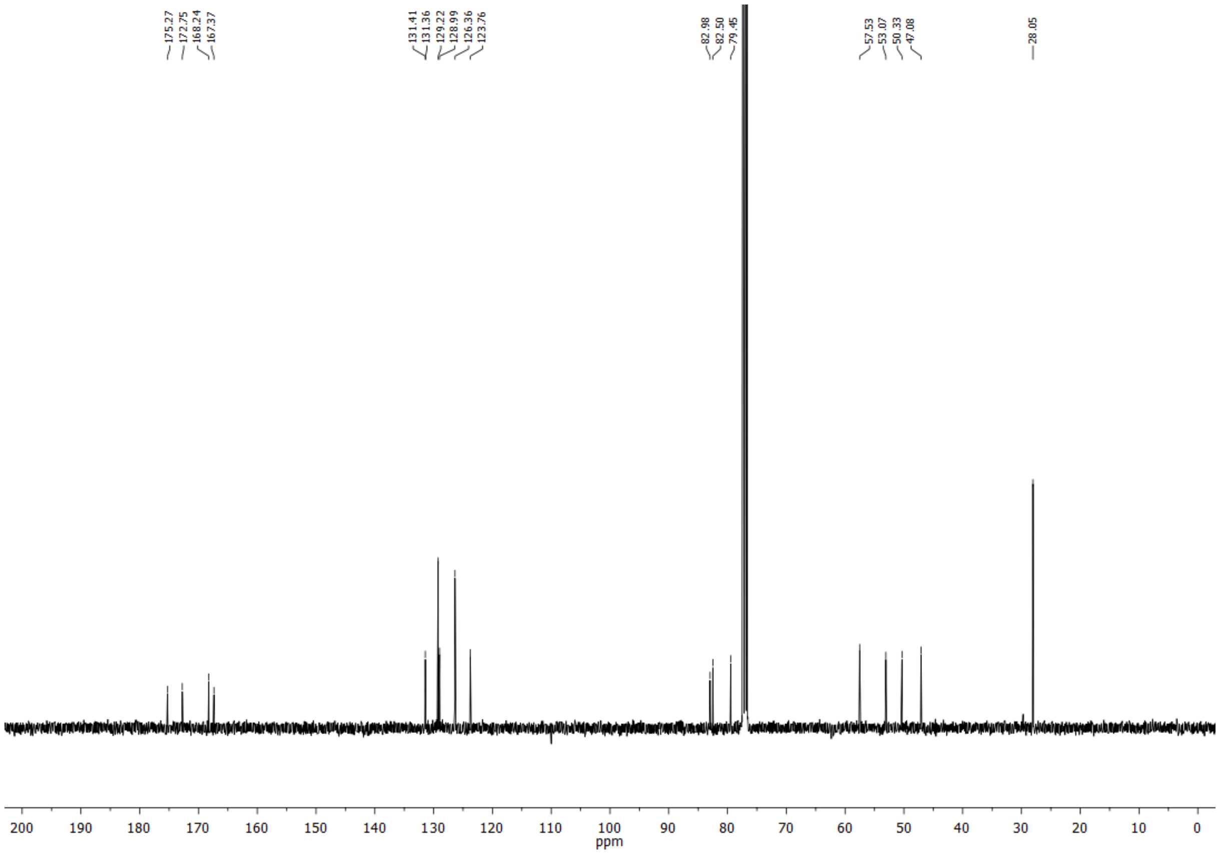

Compound *major* and *minor* **8c**, ^{1}H NMR and ^{13}C NMR ($CDCl_3$):

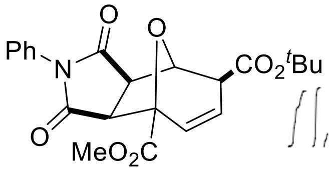

major **8c**

+

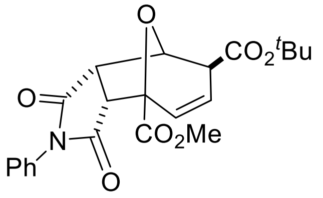

minor **8c**

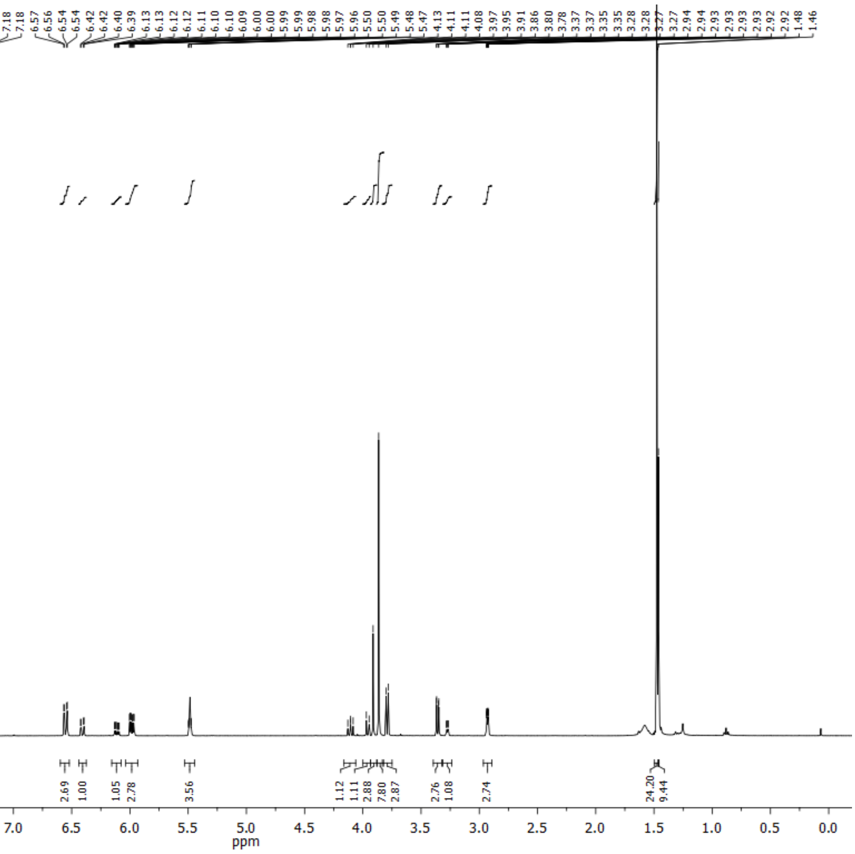

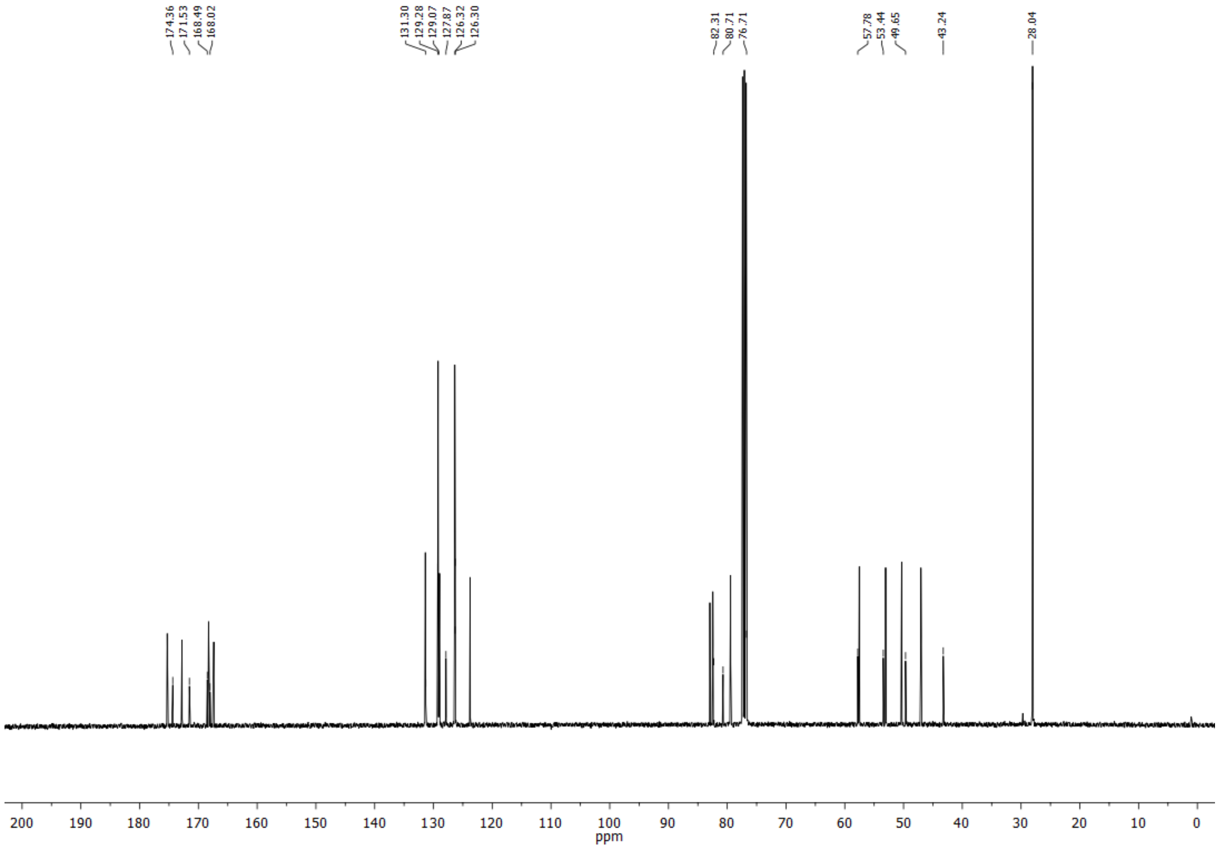

Compound *major* (–)-**8d**, ^{1}H NMR and ^{13}C NMR ($CDCl_3$):

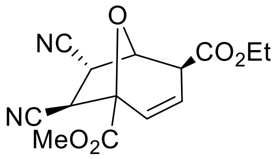

major (-)-**8d**

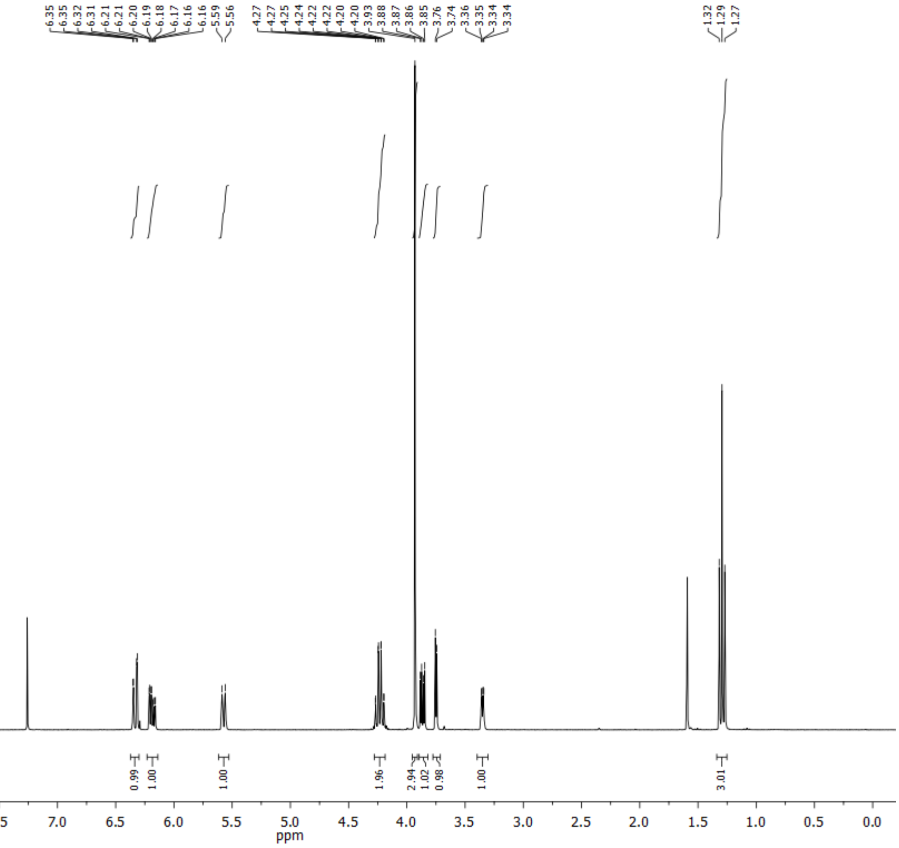

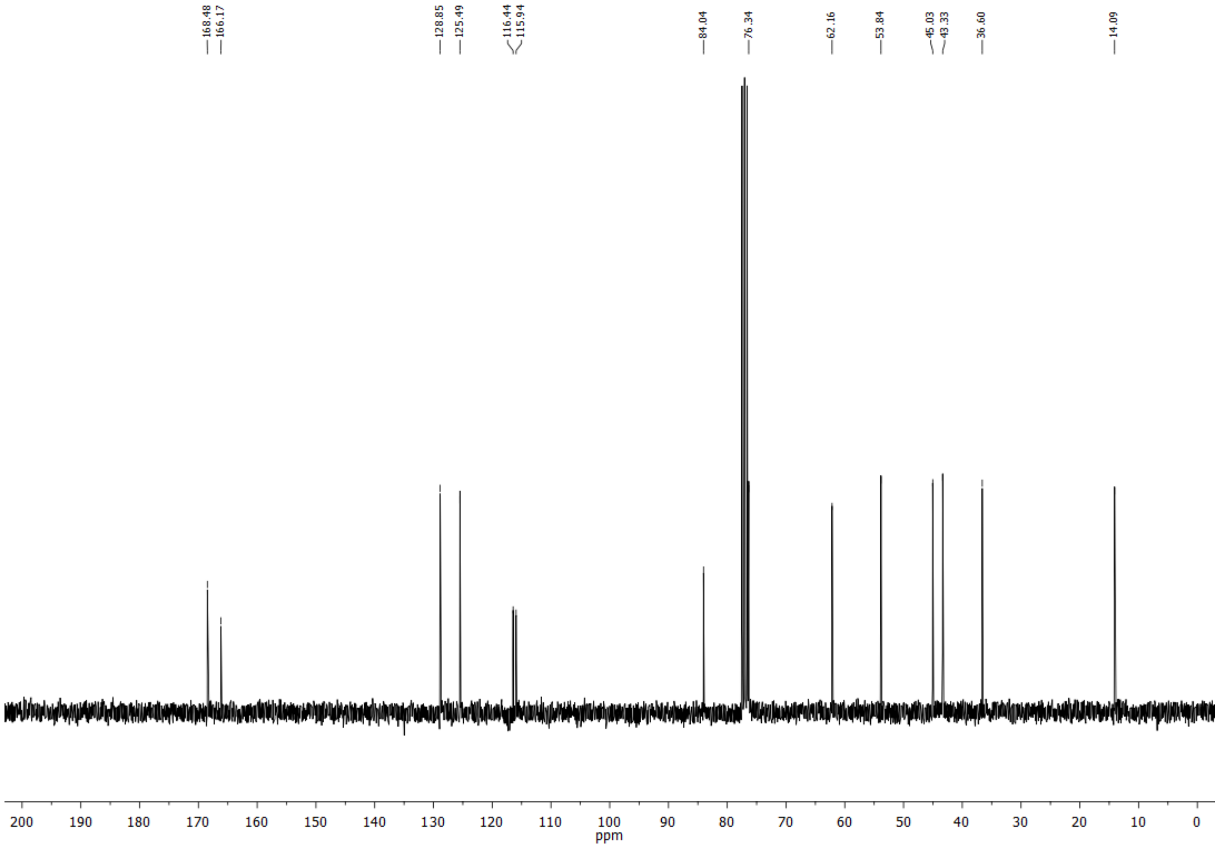

Compound *major* and *minor* **8d**, ^{1}H NMR and ^{13}C NMR ($CDCl_3$):

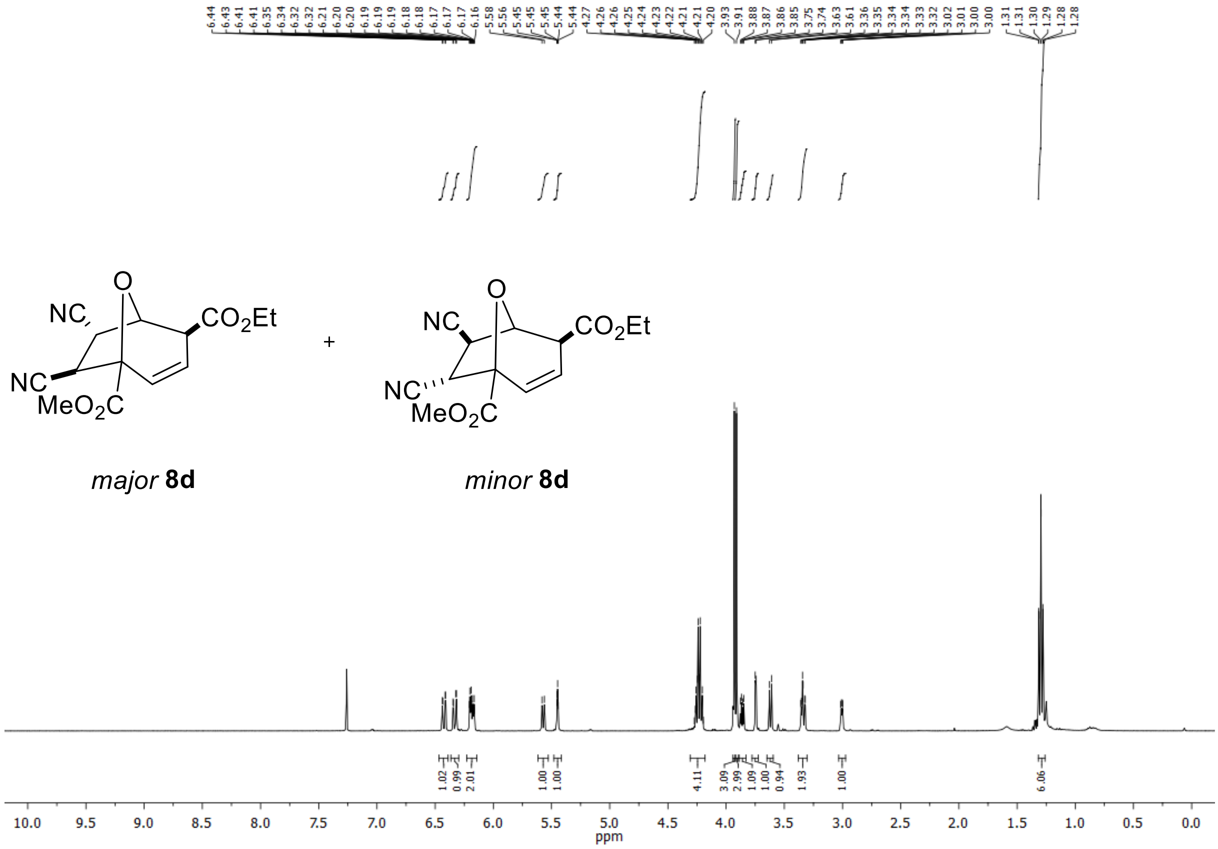

major **8d** *minor* **8d**

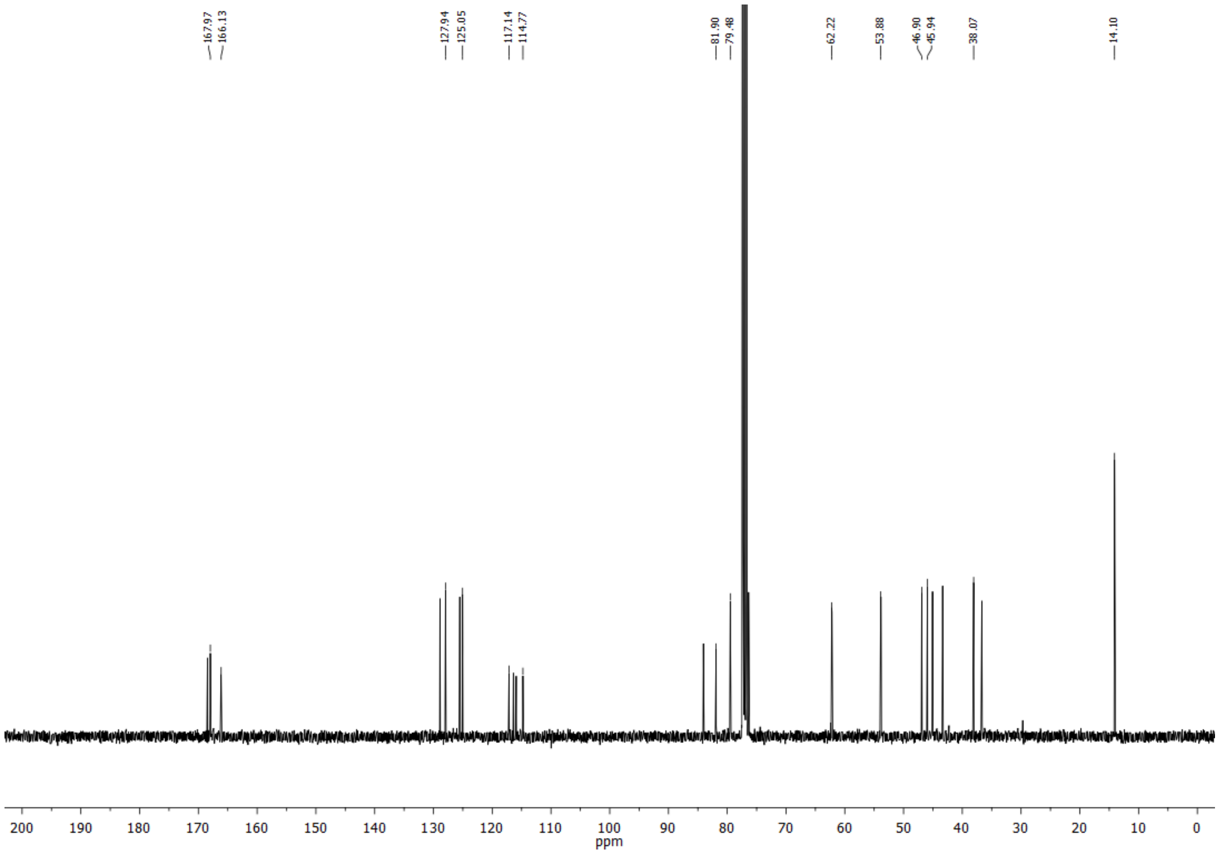

Compound **8e**, ^{1}H NMR and ^{13}C NMR ($CDCl_3$):

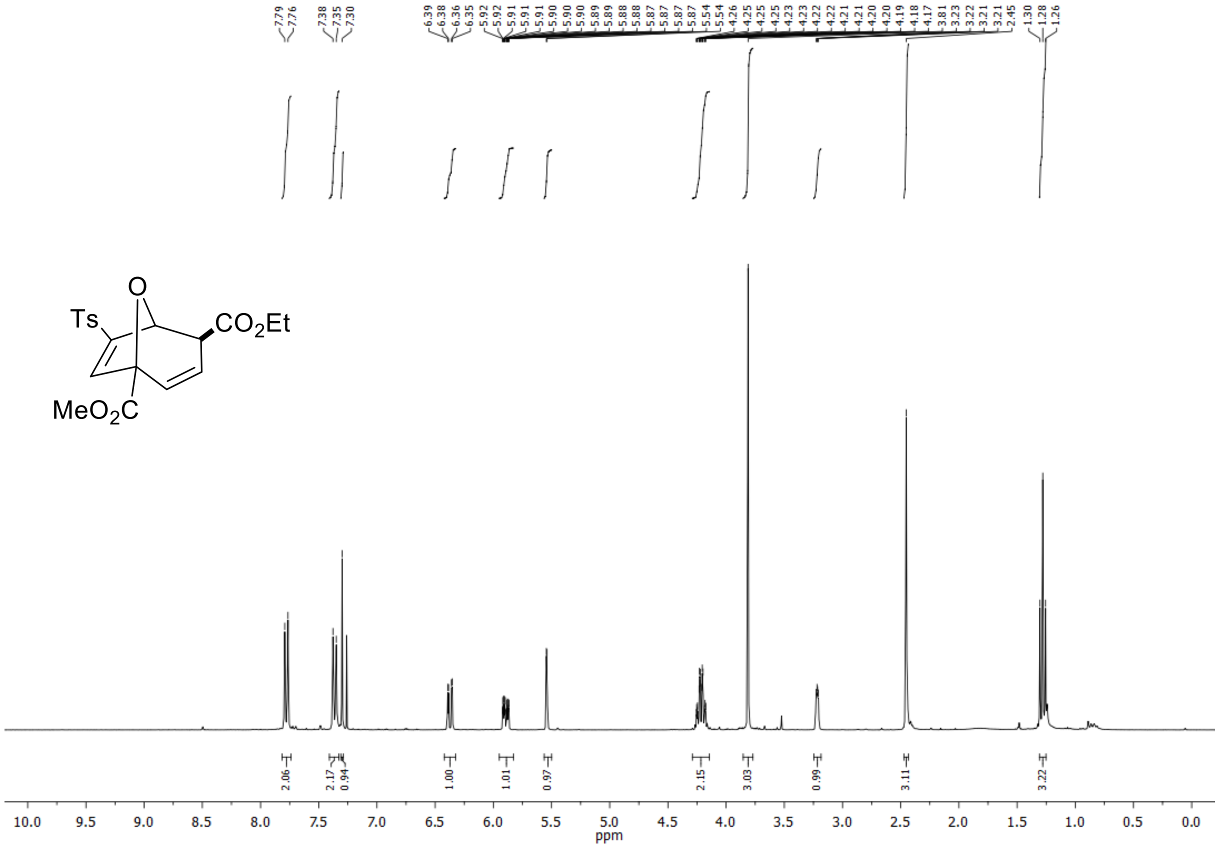

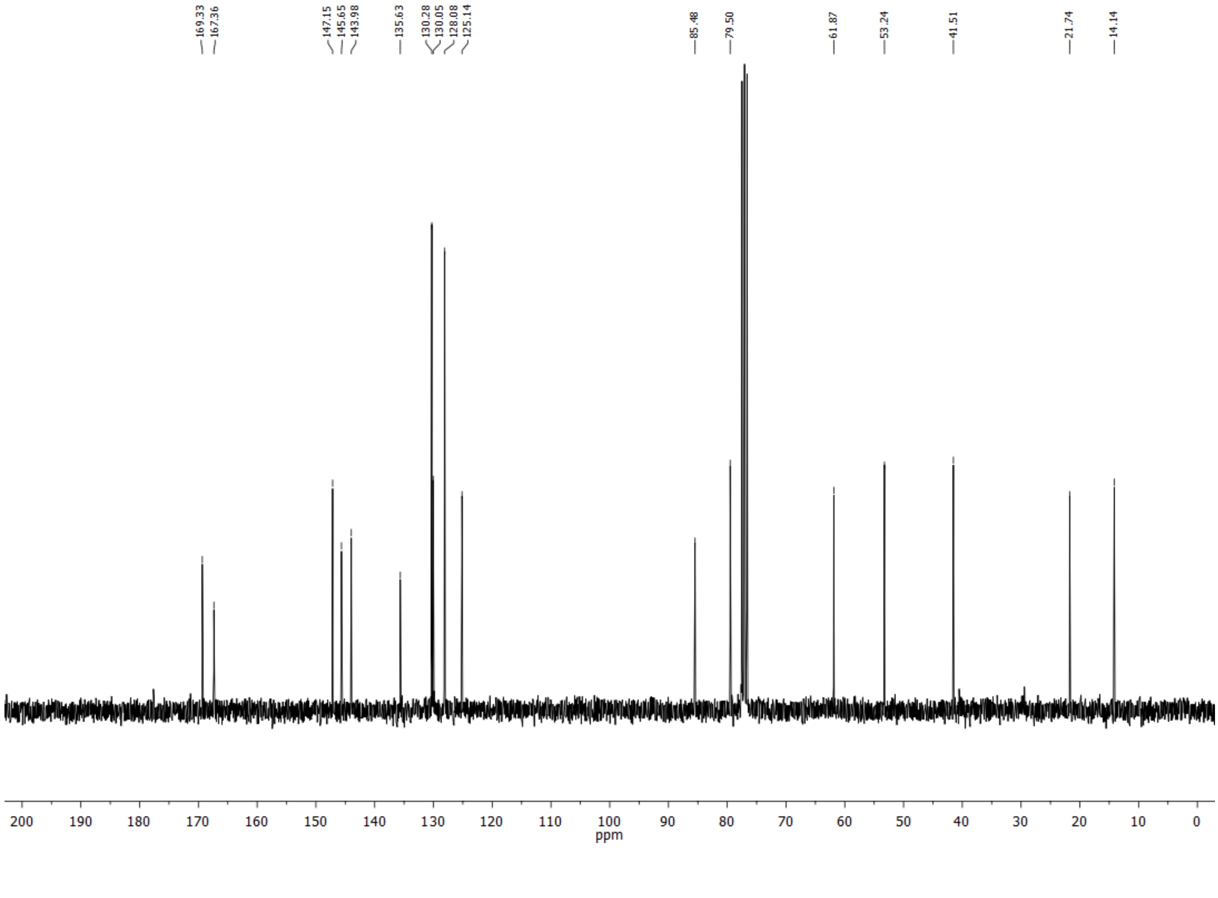

Compound **10**, ^{1}H NMR and ^{13}C NMR ($CDCl_3$):

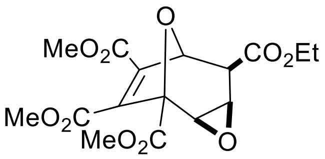

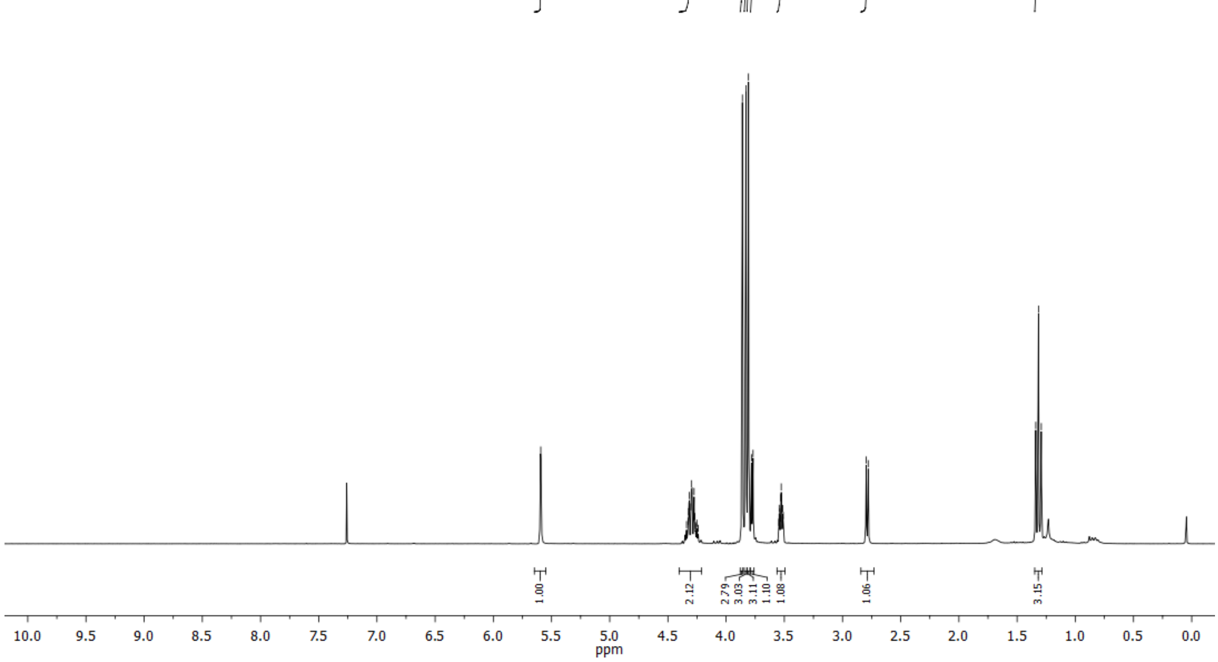

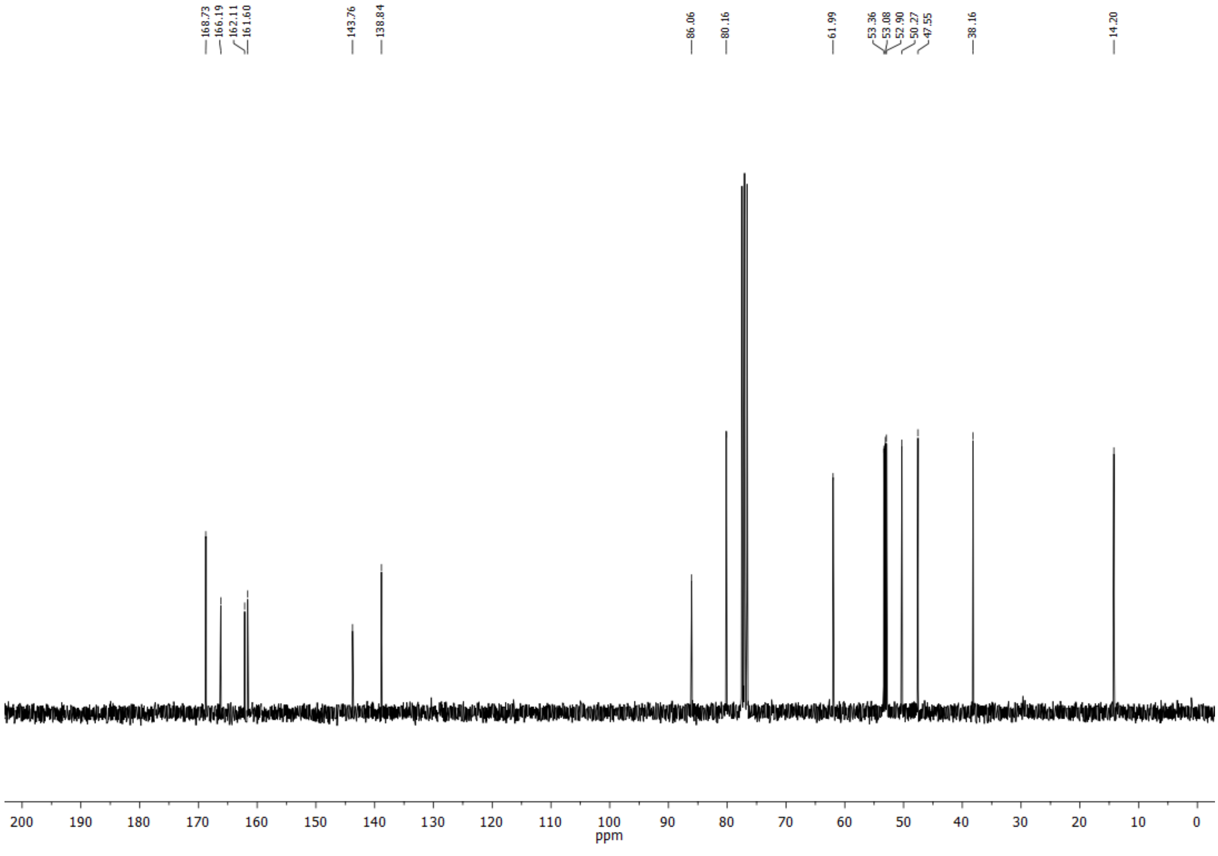

Compound **11**, ^{1}H NMR and ^{13}C NMR ($CDCl_3$):

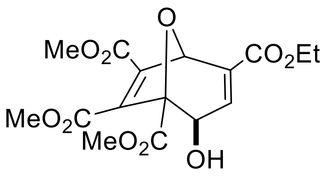

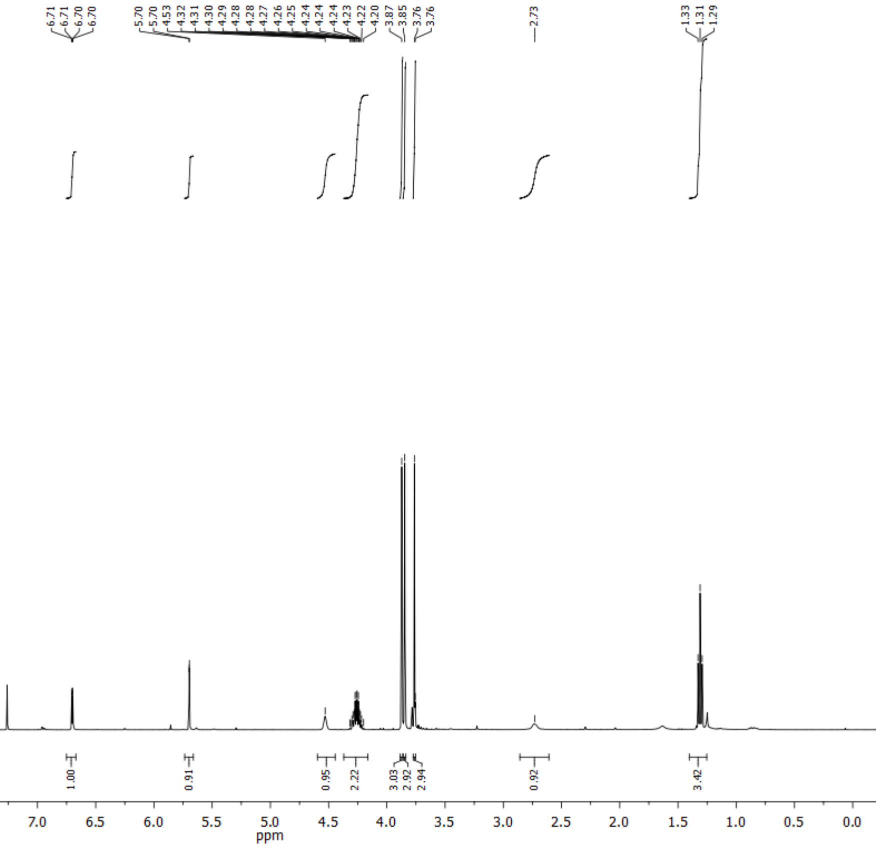

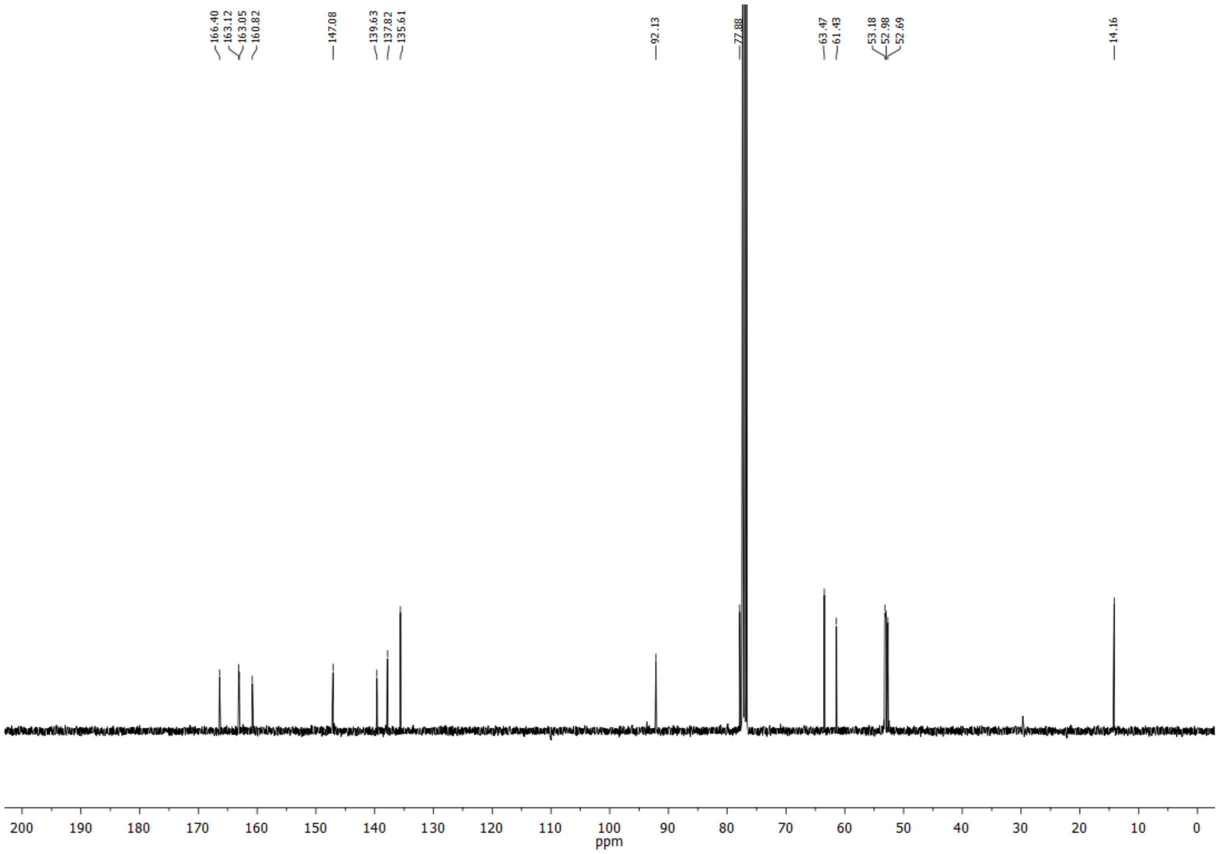

Compound **12**, ^{1}H NMR and ^{13}C NMR ($CDCl_3$):

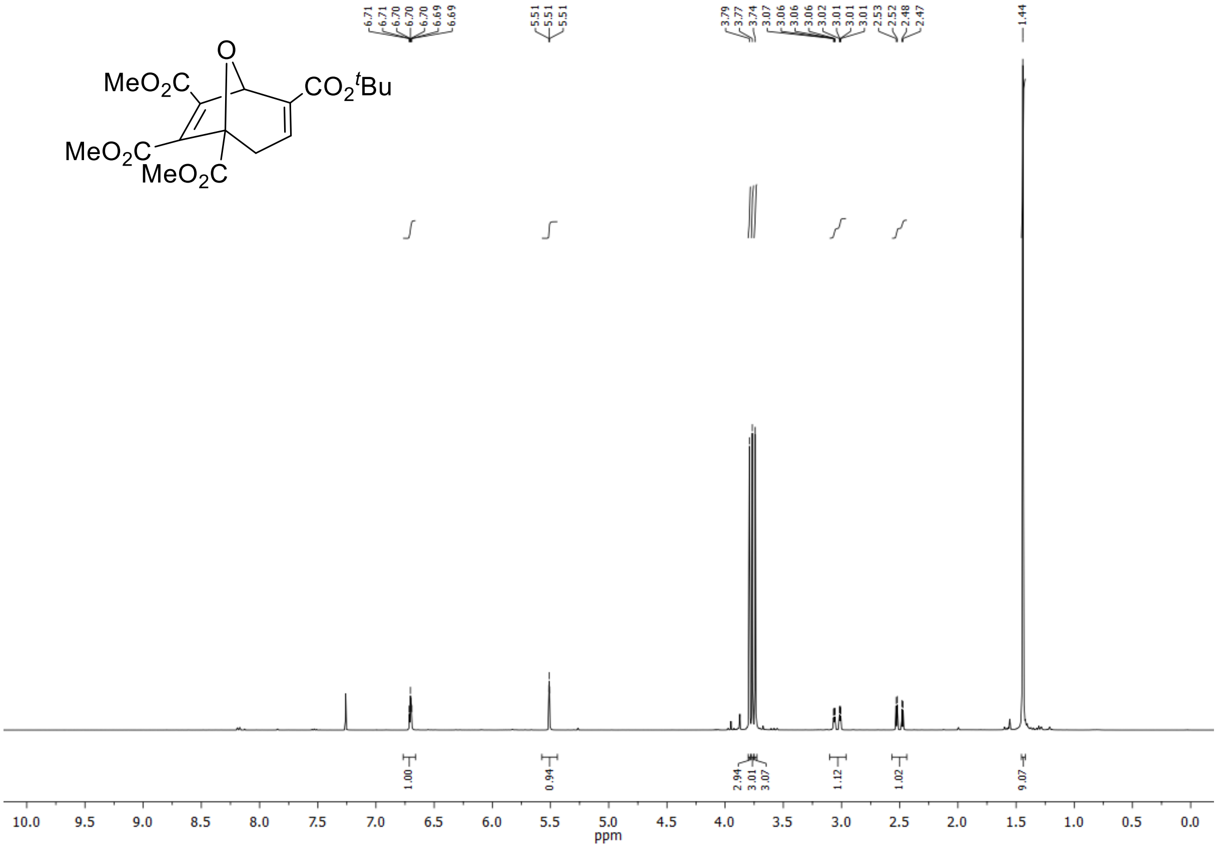

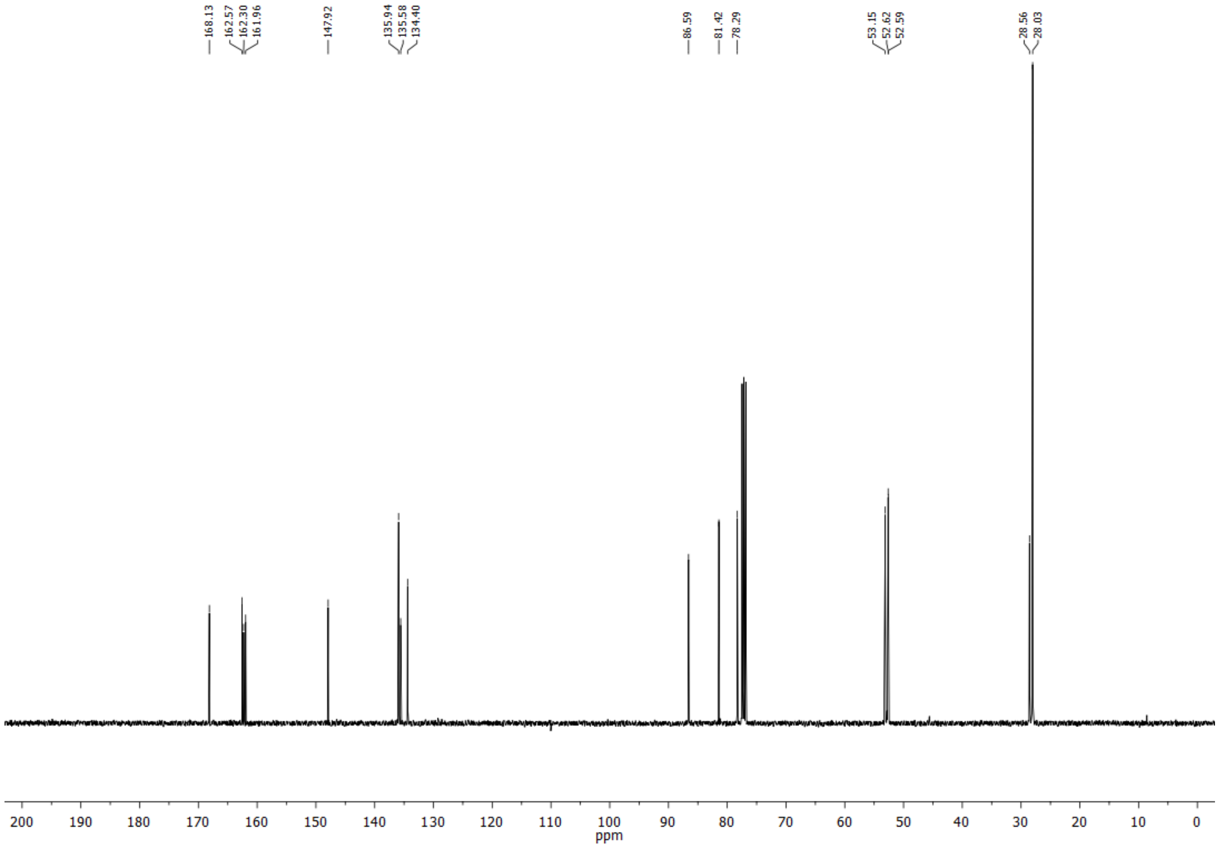

Compound **13**, ^{1}H NMR and ^{13}C NMR ($CDCl_3$):

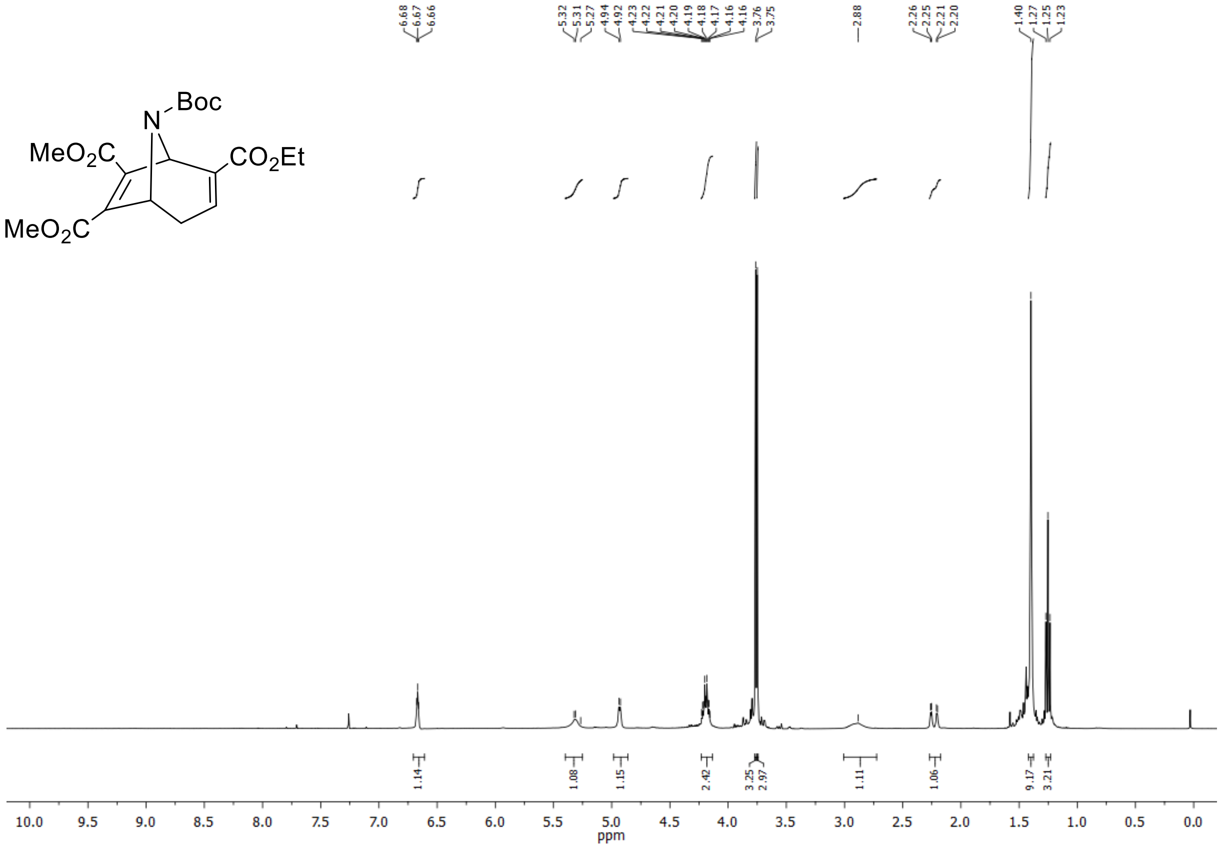

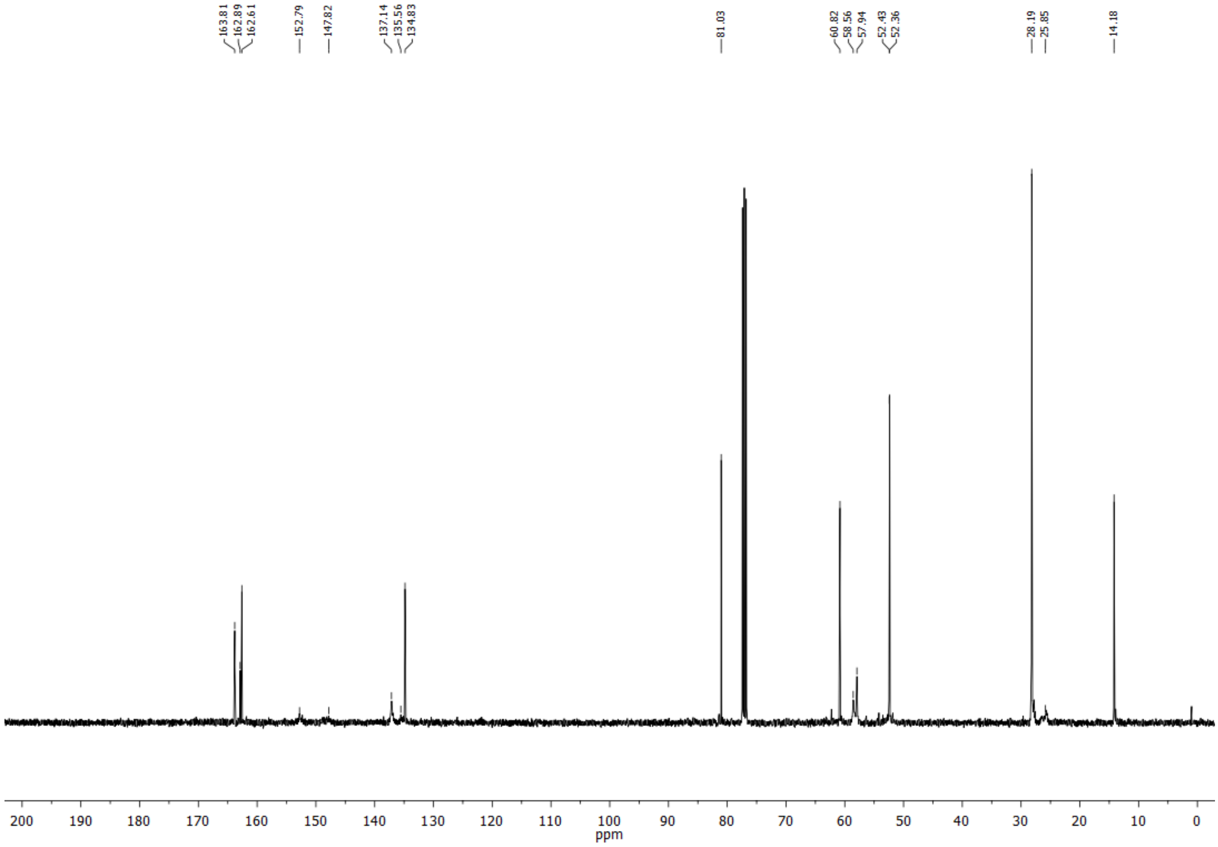

Compound **14**, ^{1}H NMR and ^{13}C NMR ($CDCl_3$):

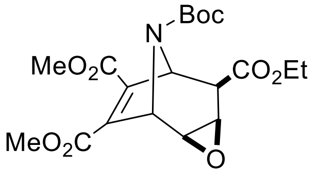

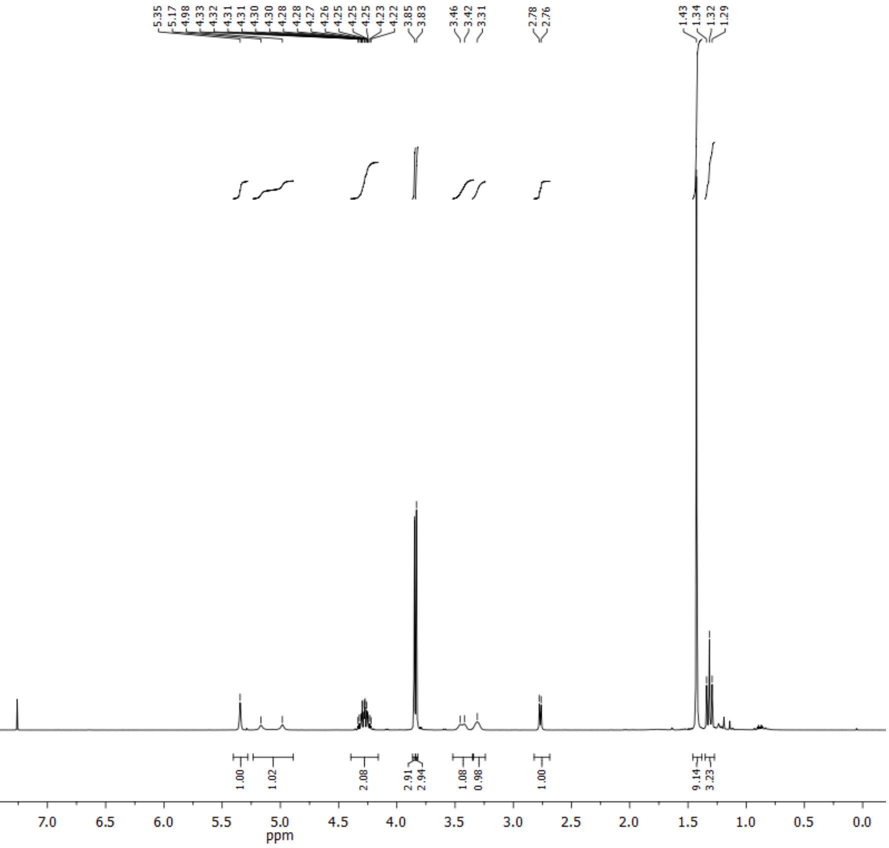

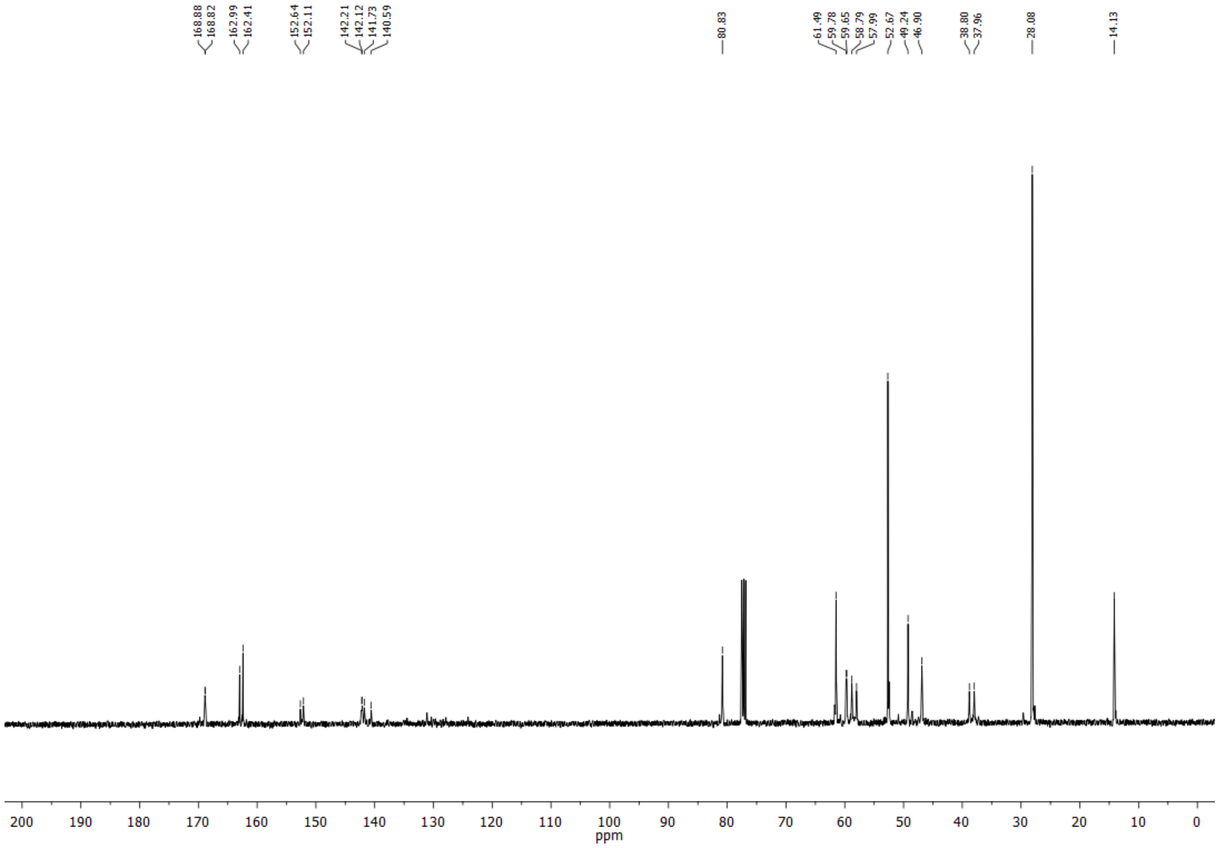

Compound **15**, ^{1}H NMR and ^{13}C NMR ($CDCl_3$):

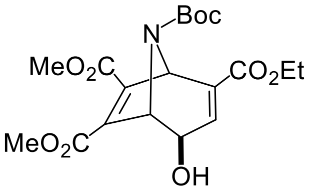

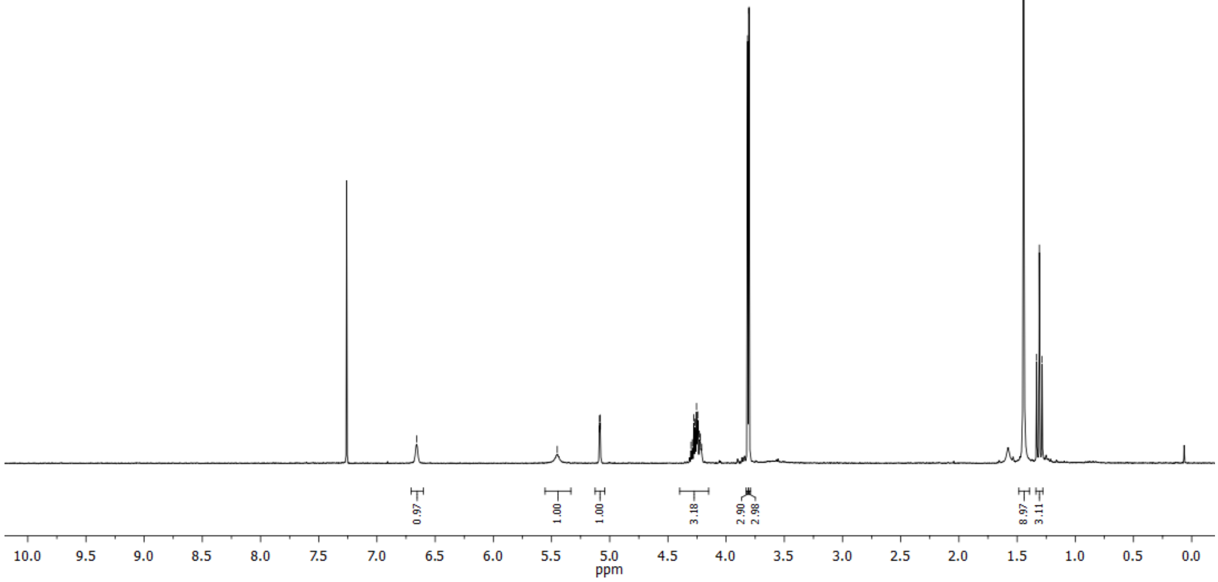

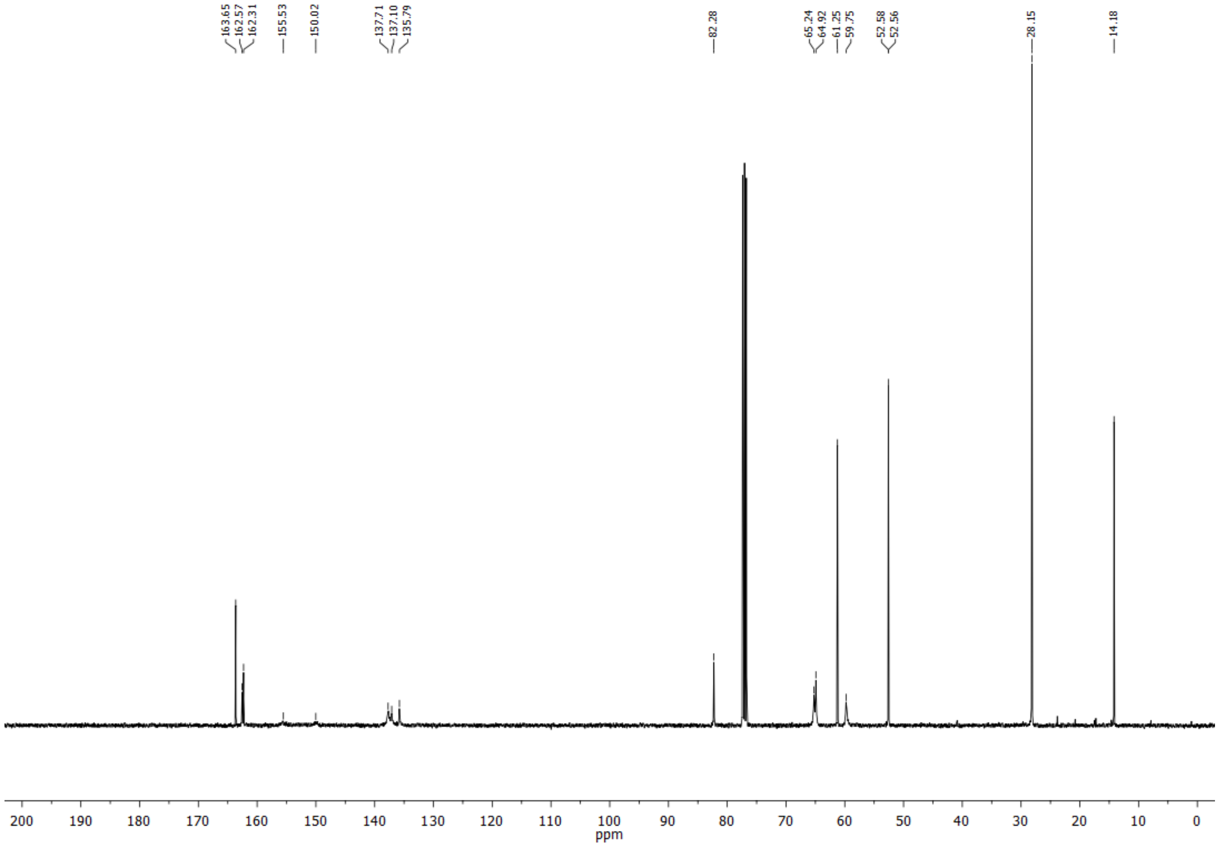

Compound **16**, ^{1}H NMR and ^{13}C NMR ($CDCl_3$):

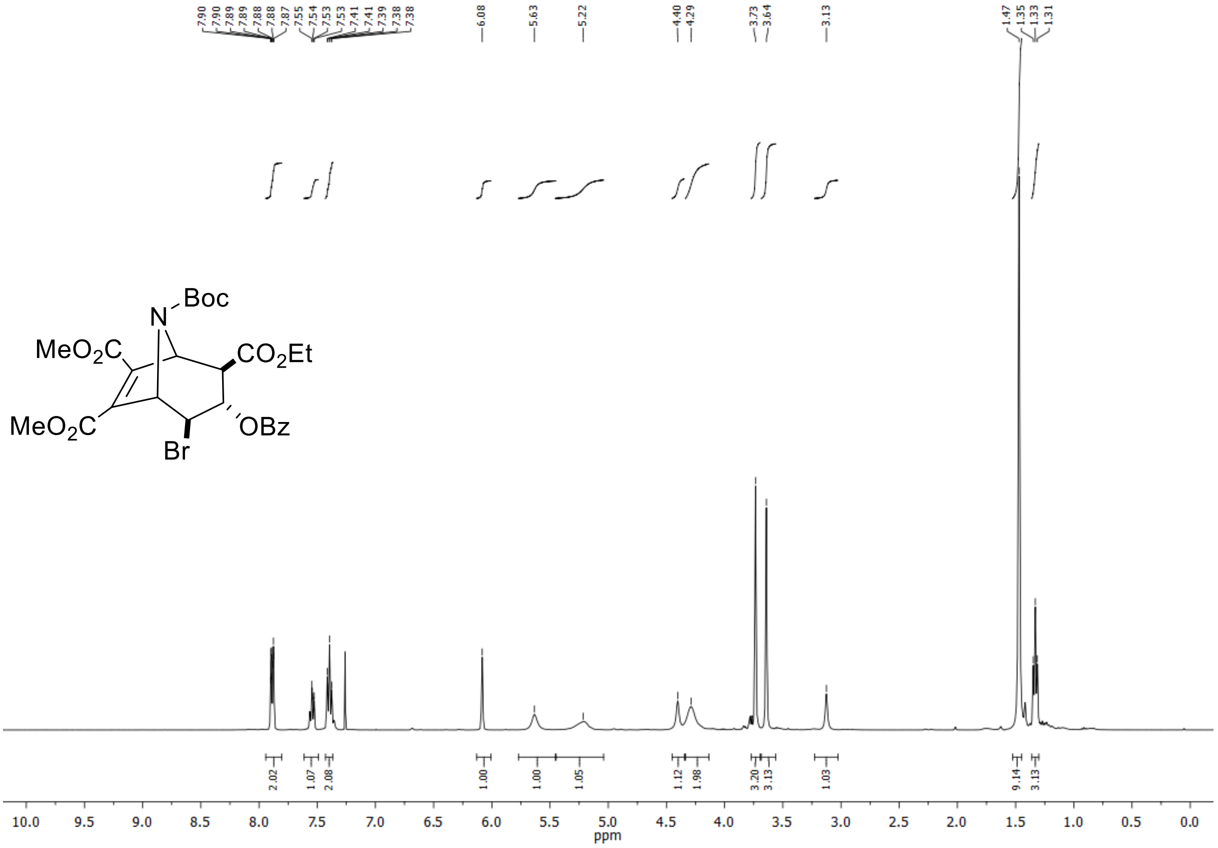

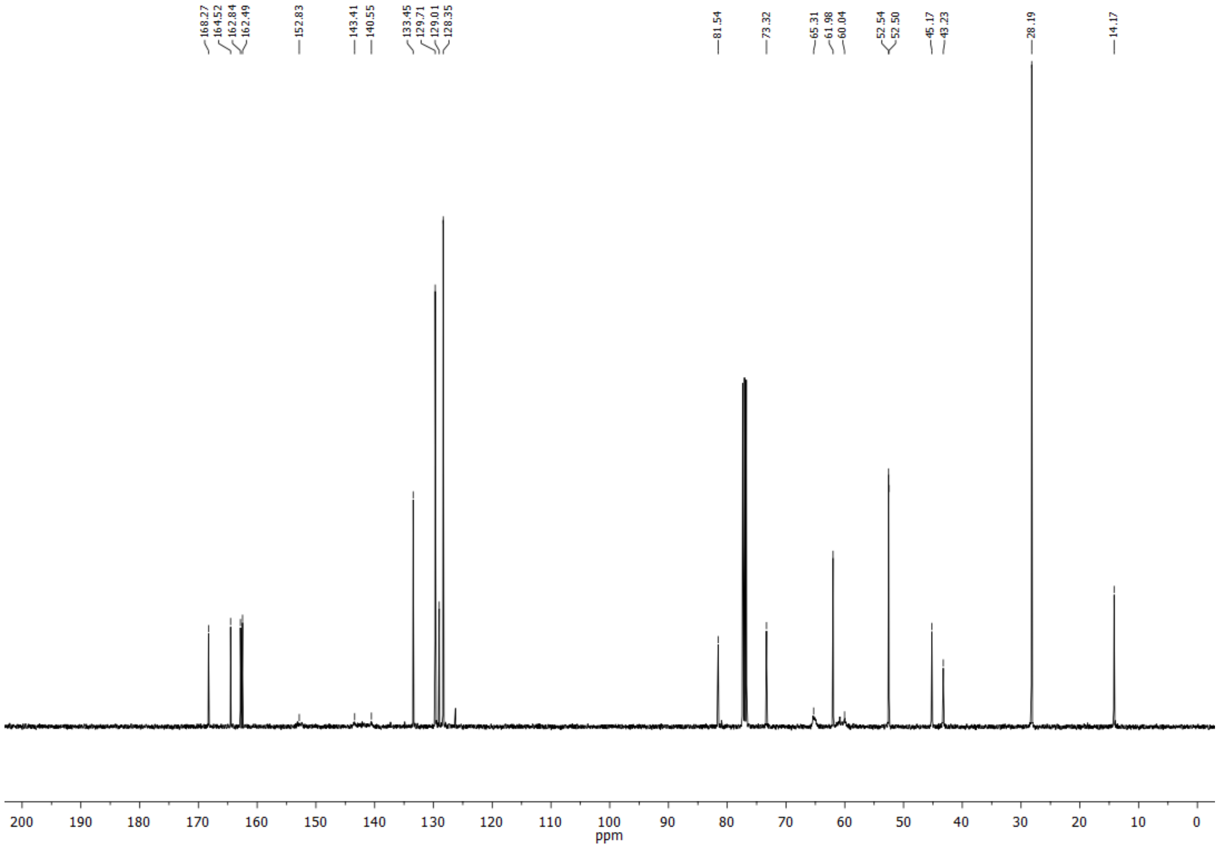

Compound **17**, ^{1}H NMR and ^{13}C NMR ($CDCl_3$):

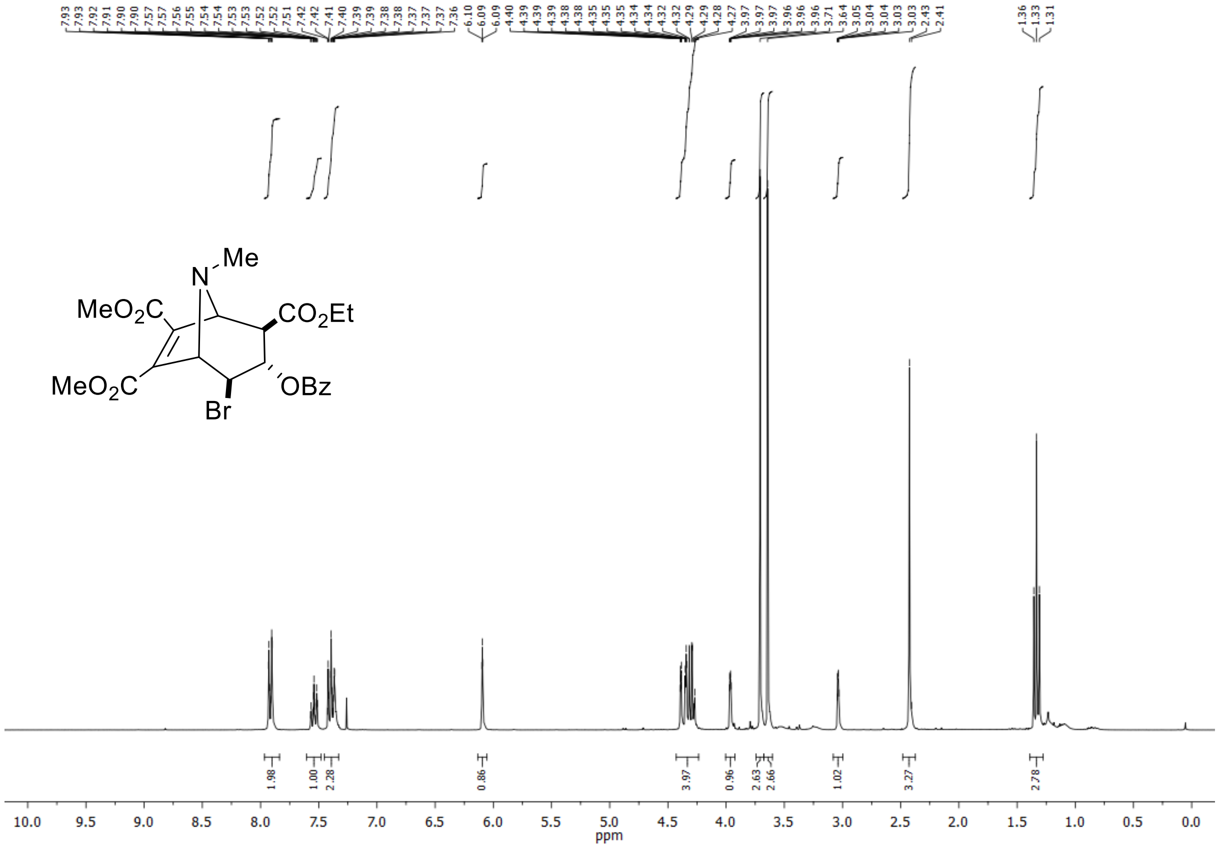

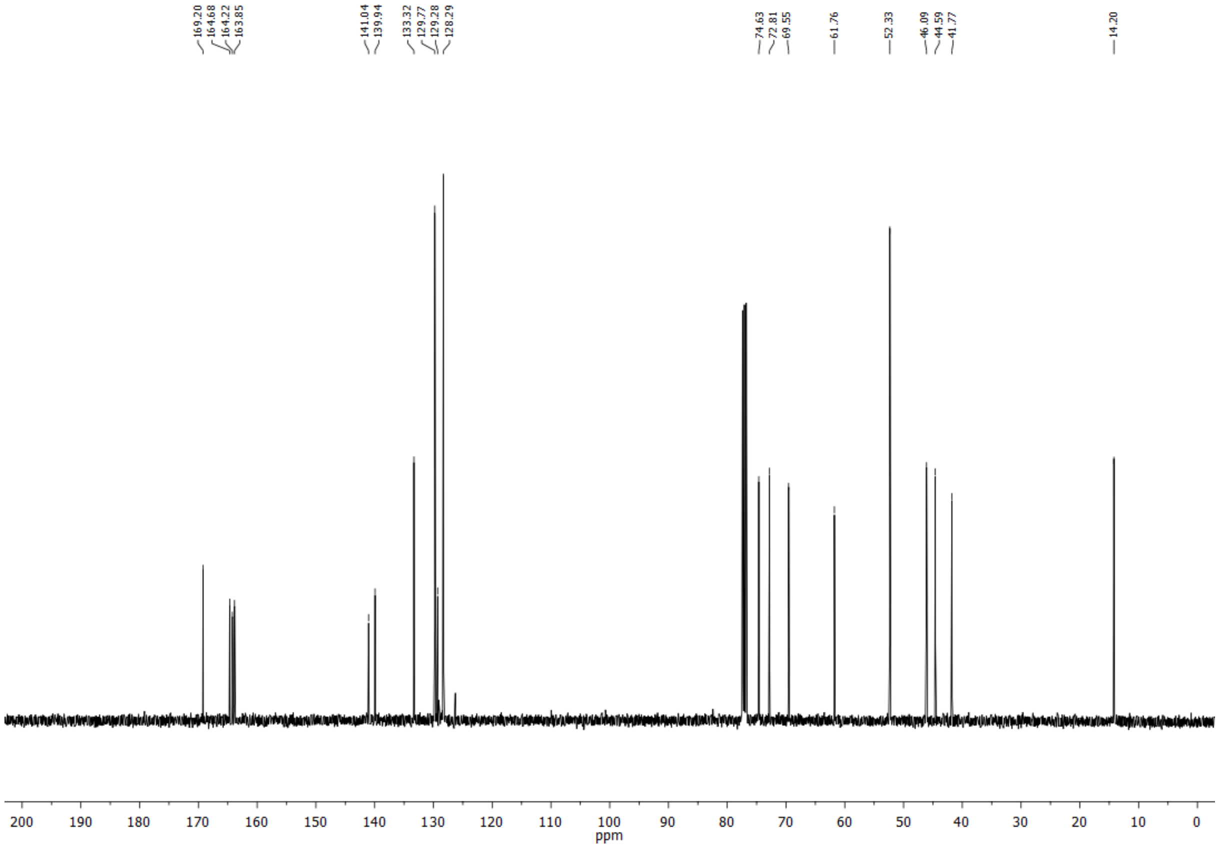

Compound **18**, ^1H NMR and ^{13}C NMR ($CDCl_3$):

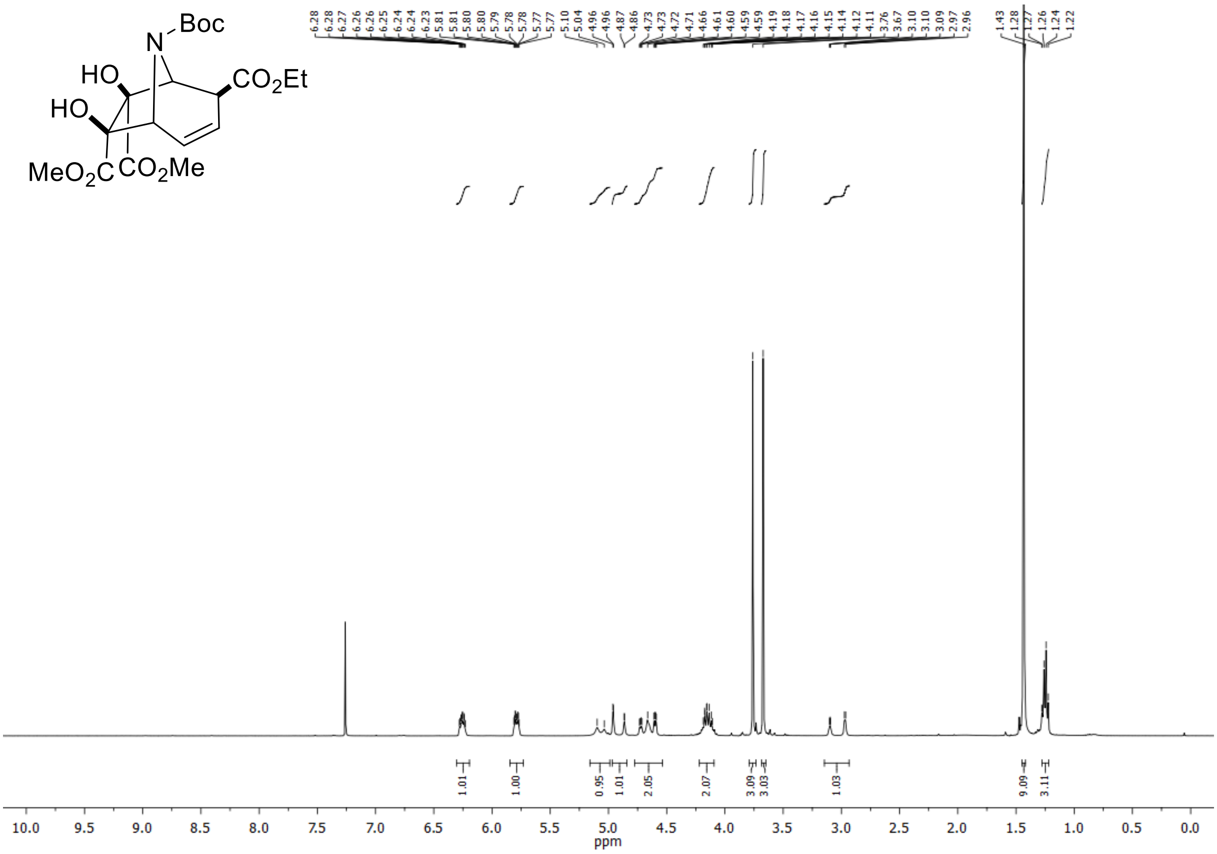

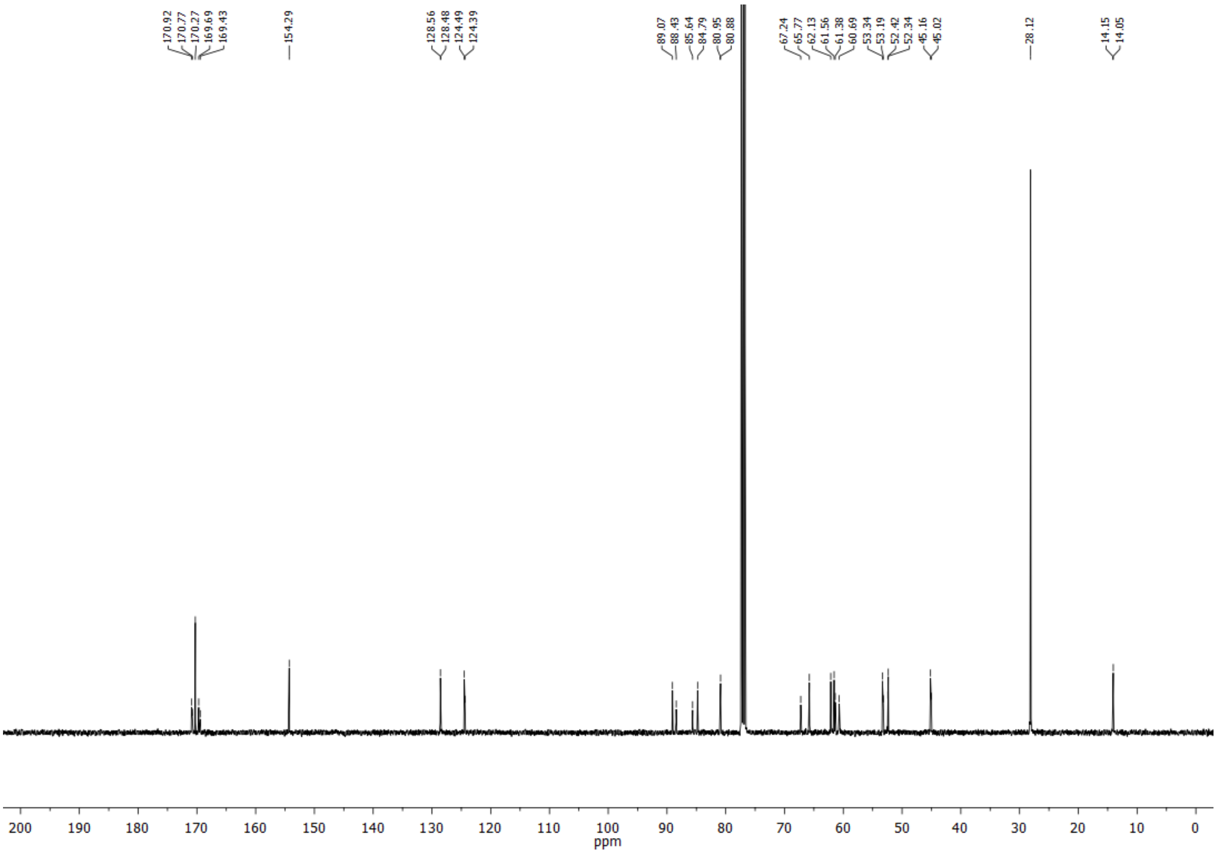

Compound **19**, ^{1}H NMR and ^{13}C NMR ($CDCl_3$):

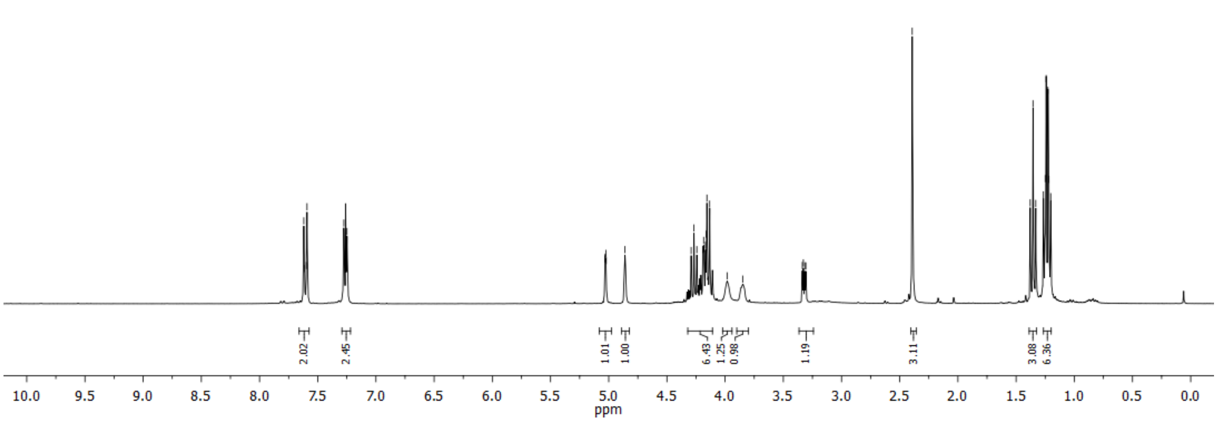

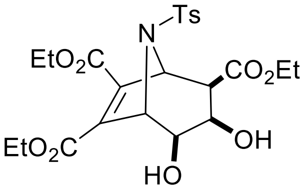

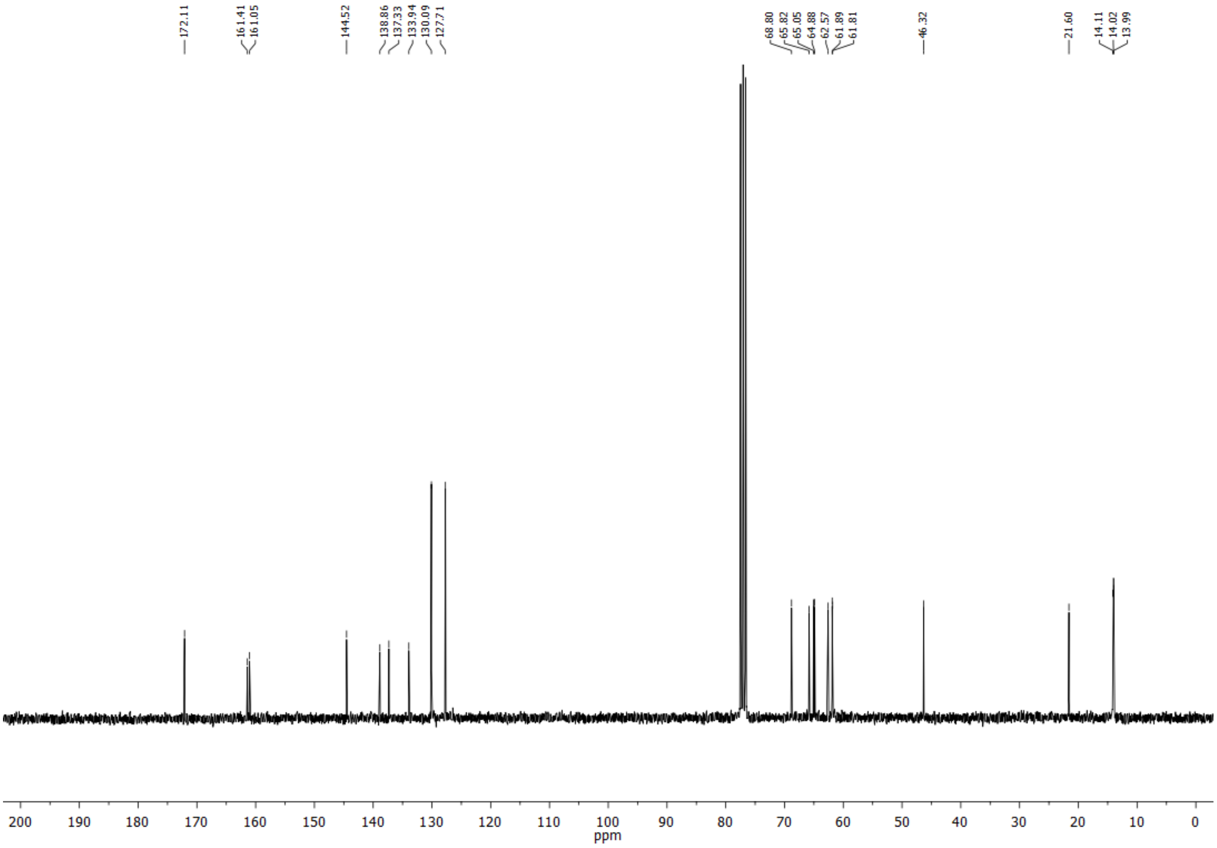

Compound **20**, ^{1}H NMR and ^{13}C NMR ($CDCl_3$):

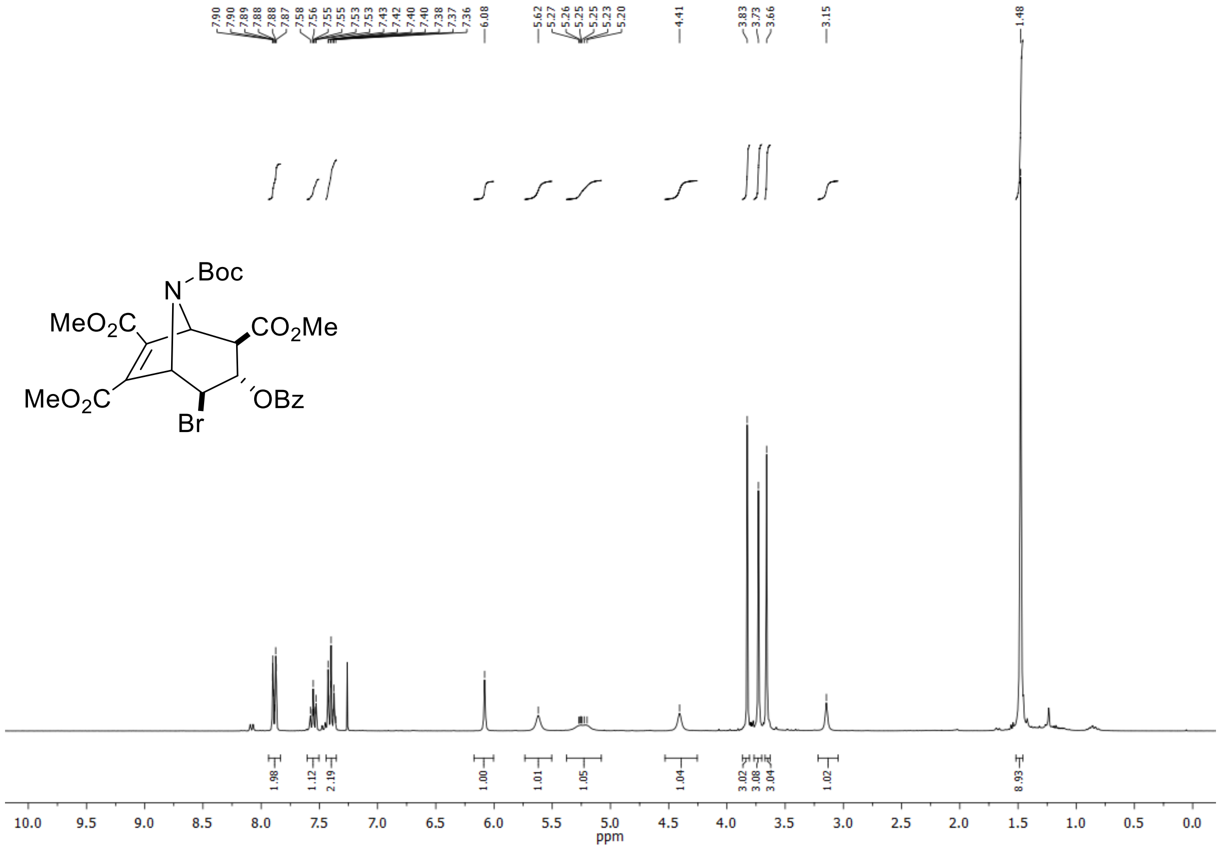

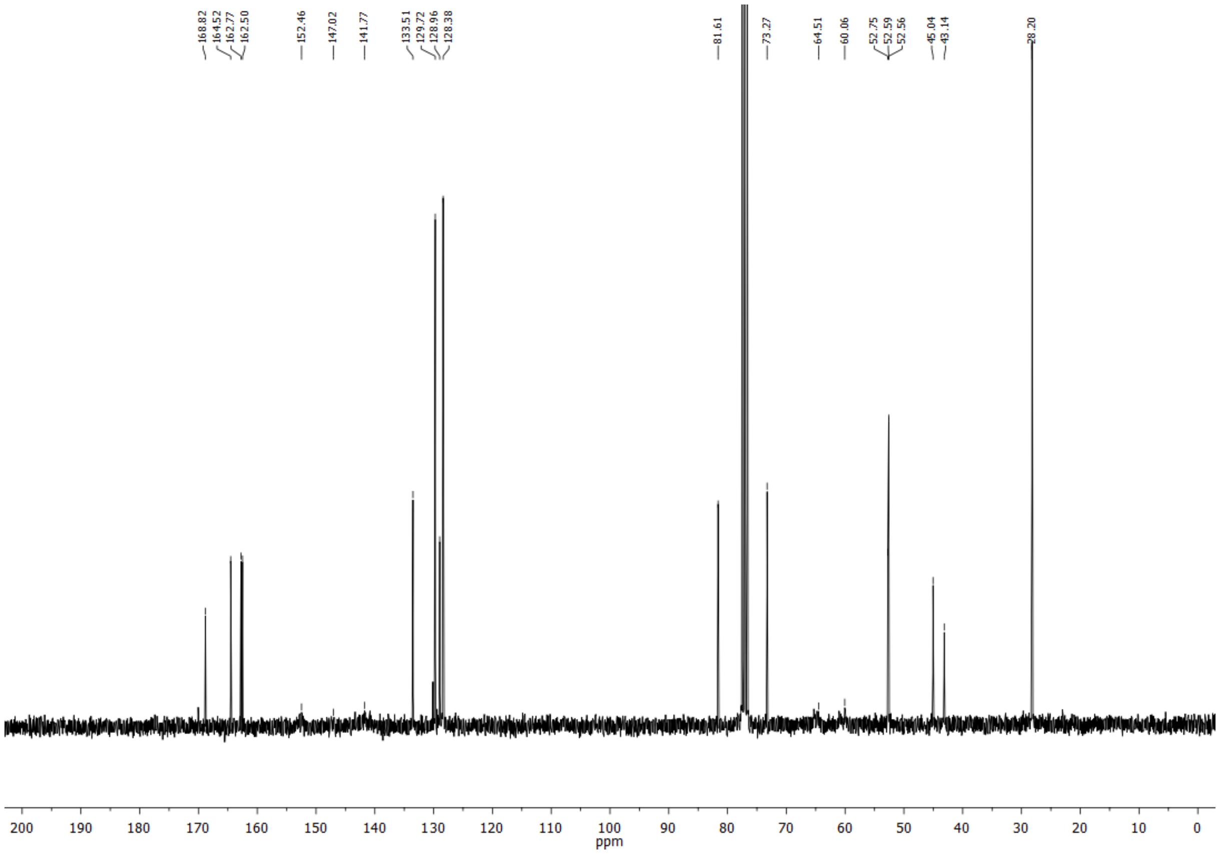

Compound **21**, ^{1}H NMR and ^{13}C NMR ($CDCl_3$):

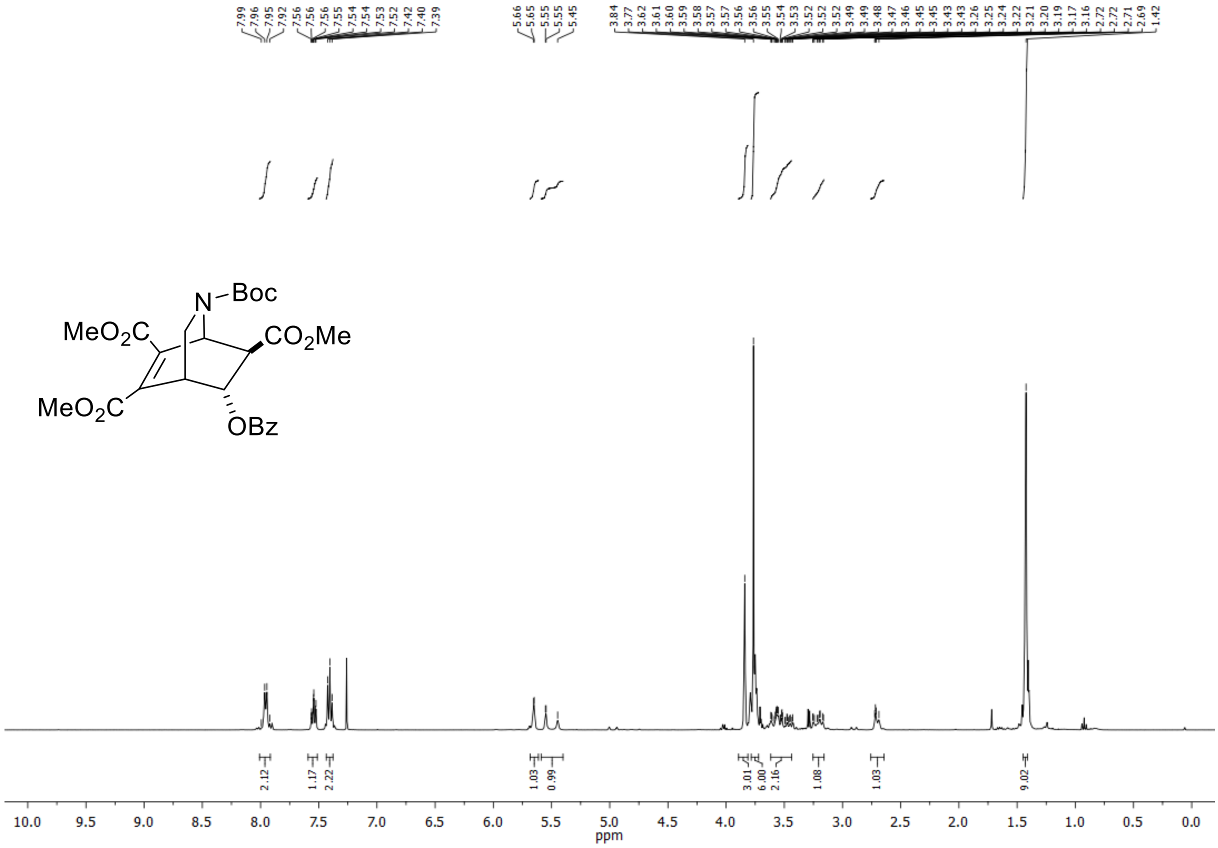

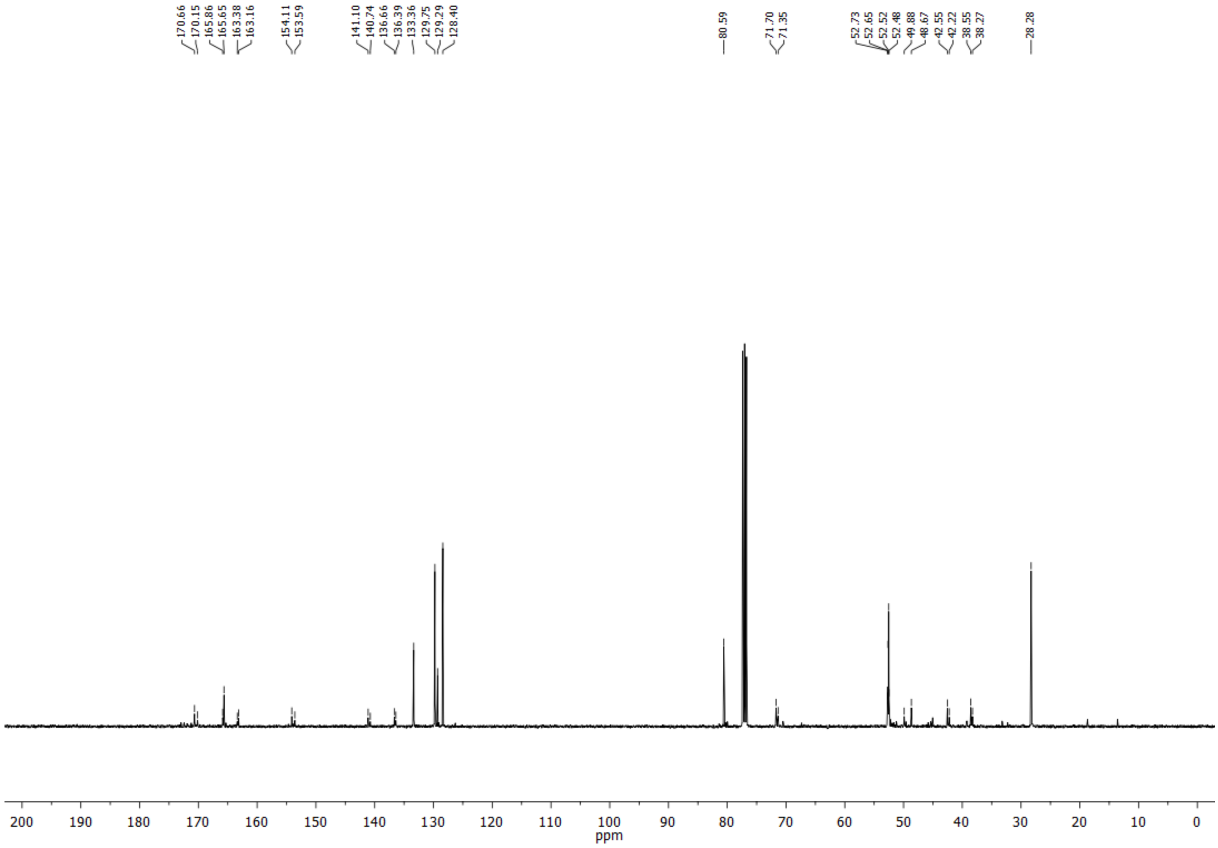

Compound **22**, ^{1}H NMR and ^{13}C NMR ($CDCl_3$):

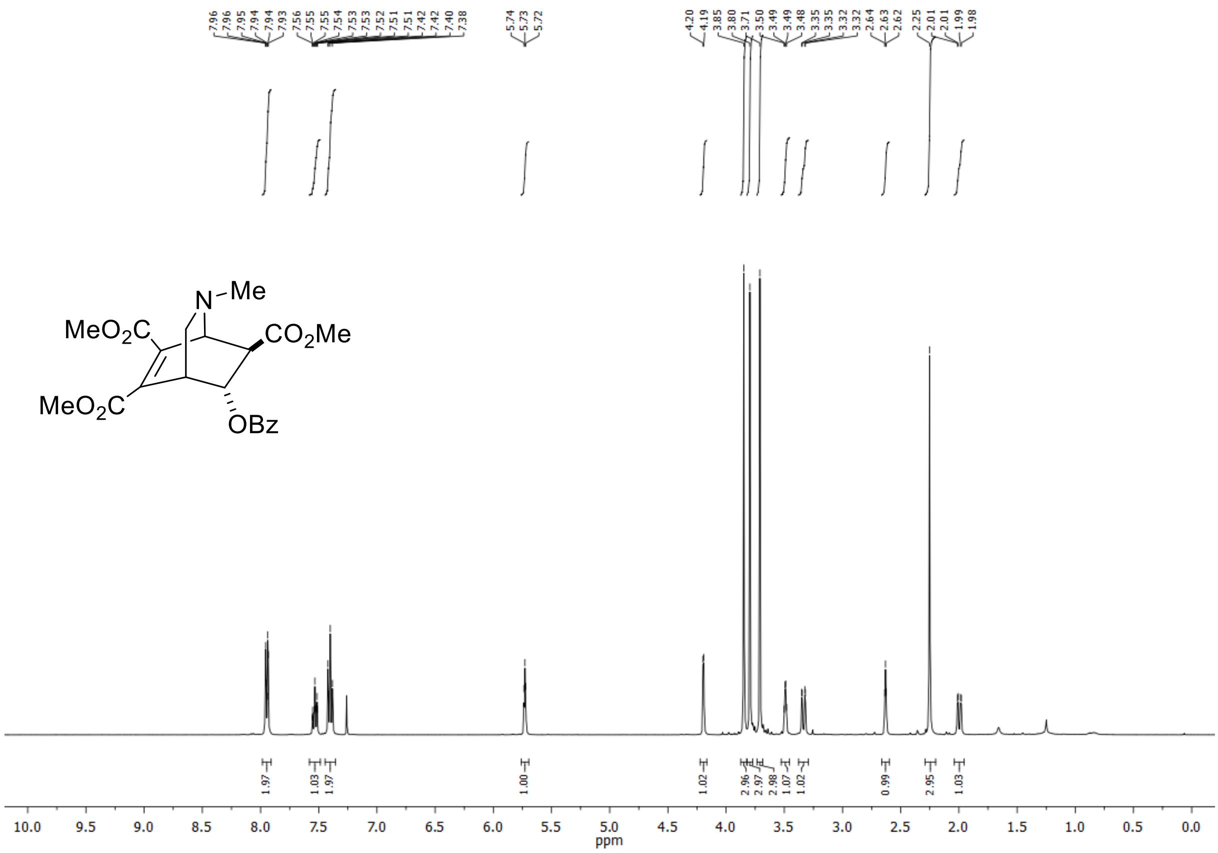

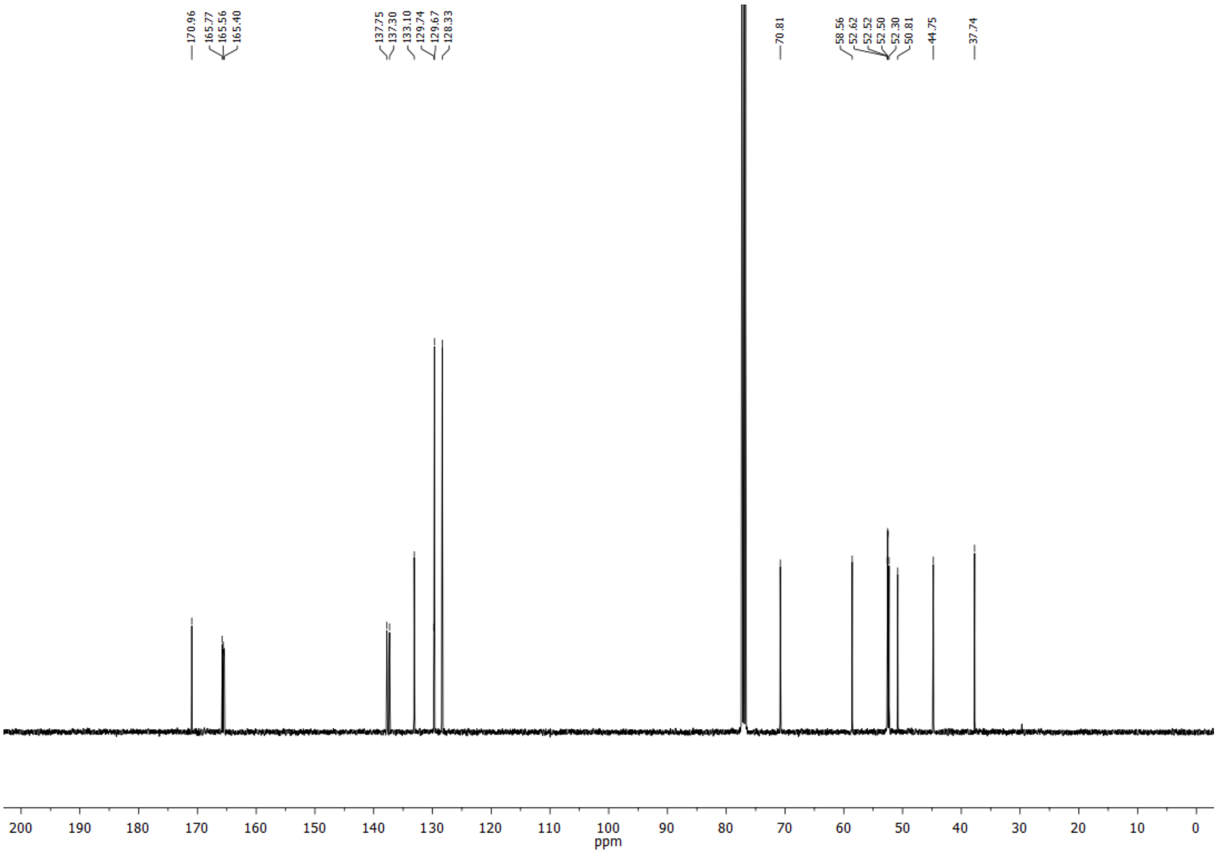

Compound **23**, ^{1}H NMR and ^{13}C NMR ($CDCl_3$):

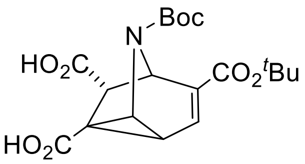

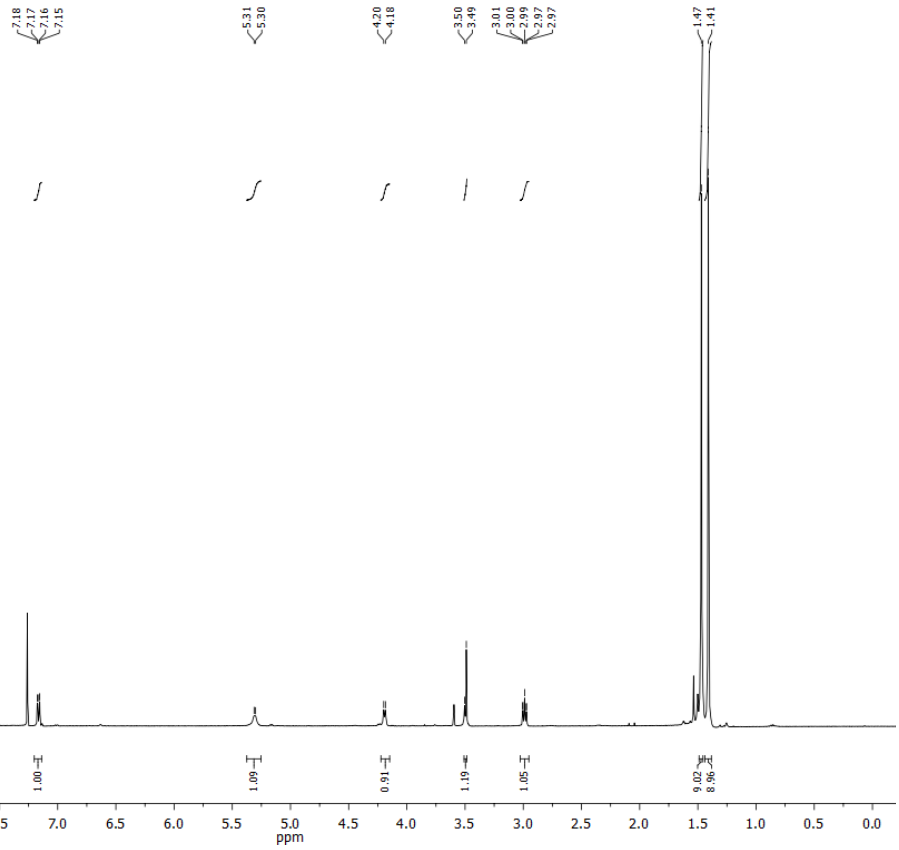

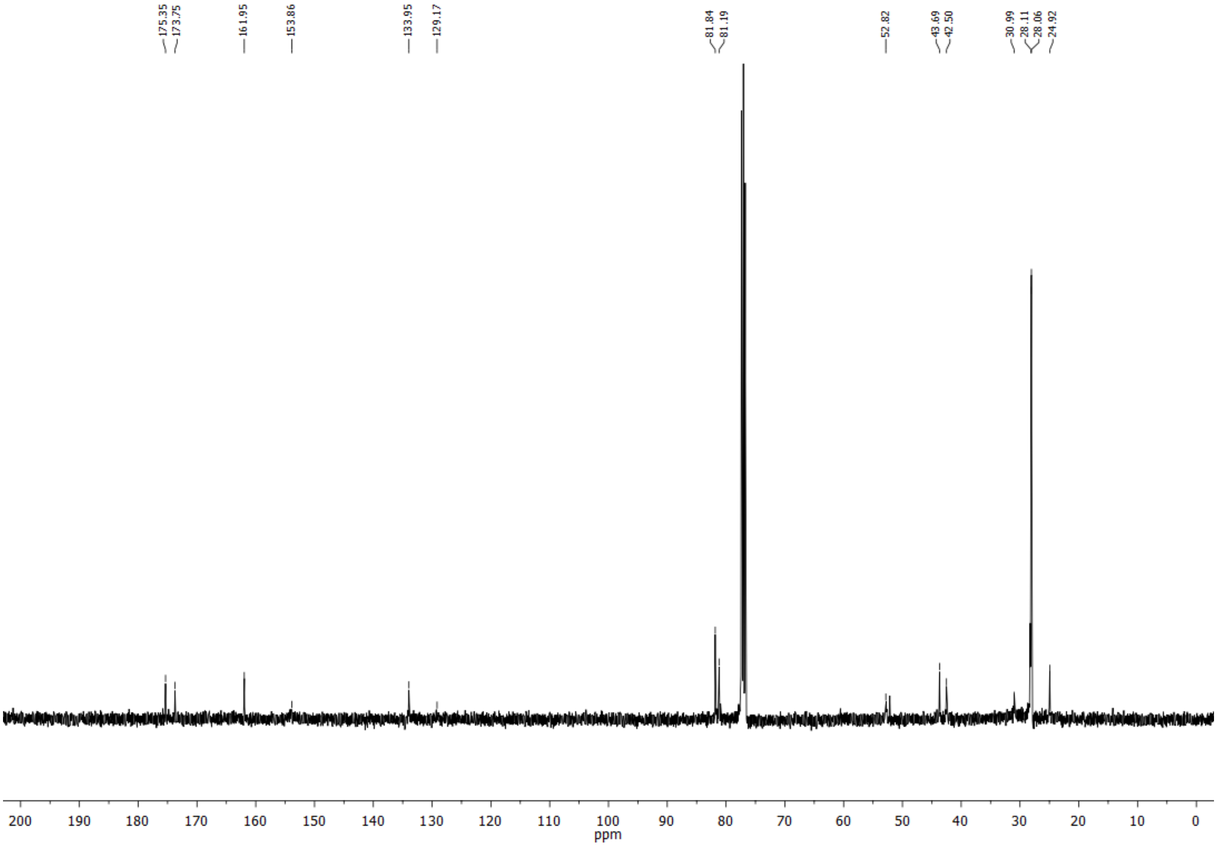

Compound **24**, ^{1}H NMR and ^{13}C NMR ($CDCl_3$):

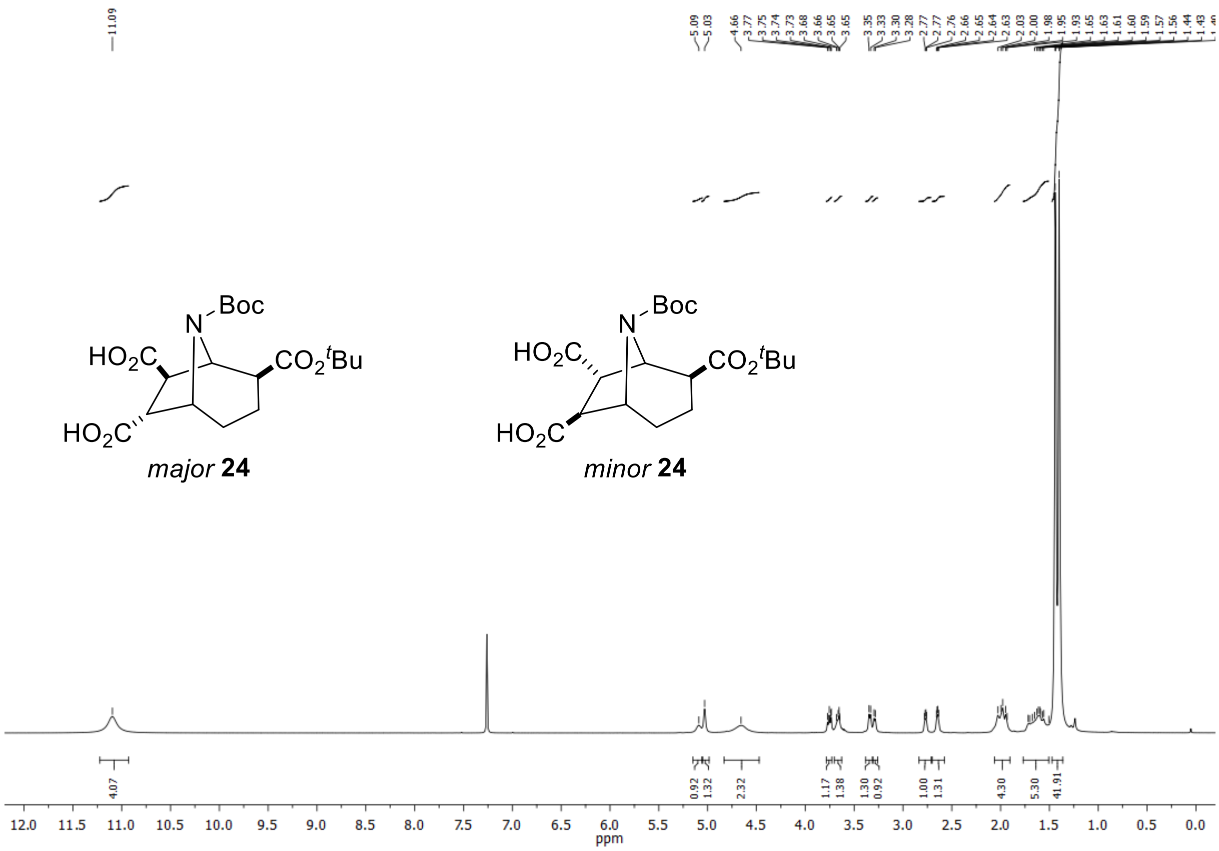

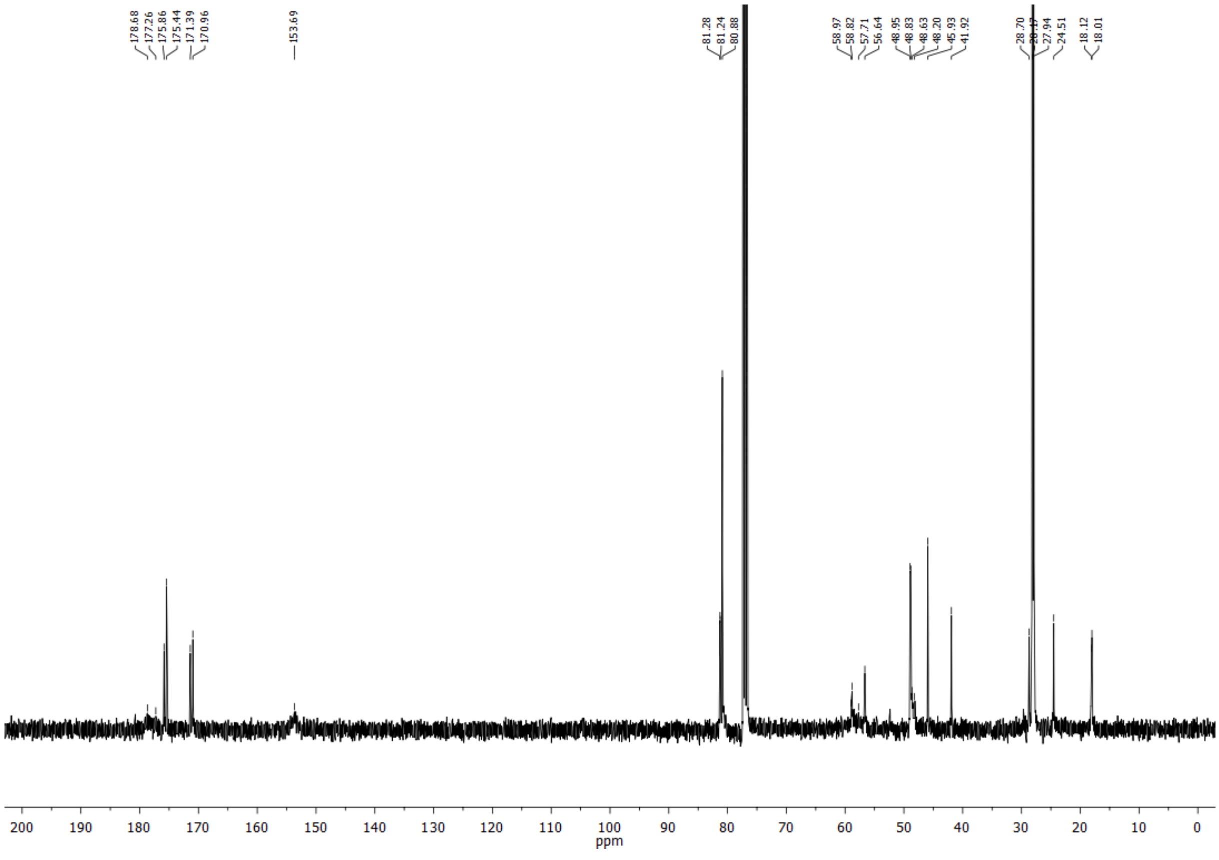

Compound **25**, ^{1}H NMR and ^{13}C NMR (DMF-d_7, 333 K):

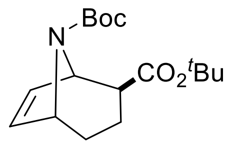

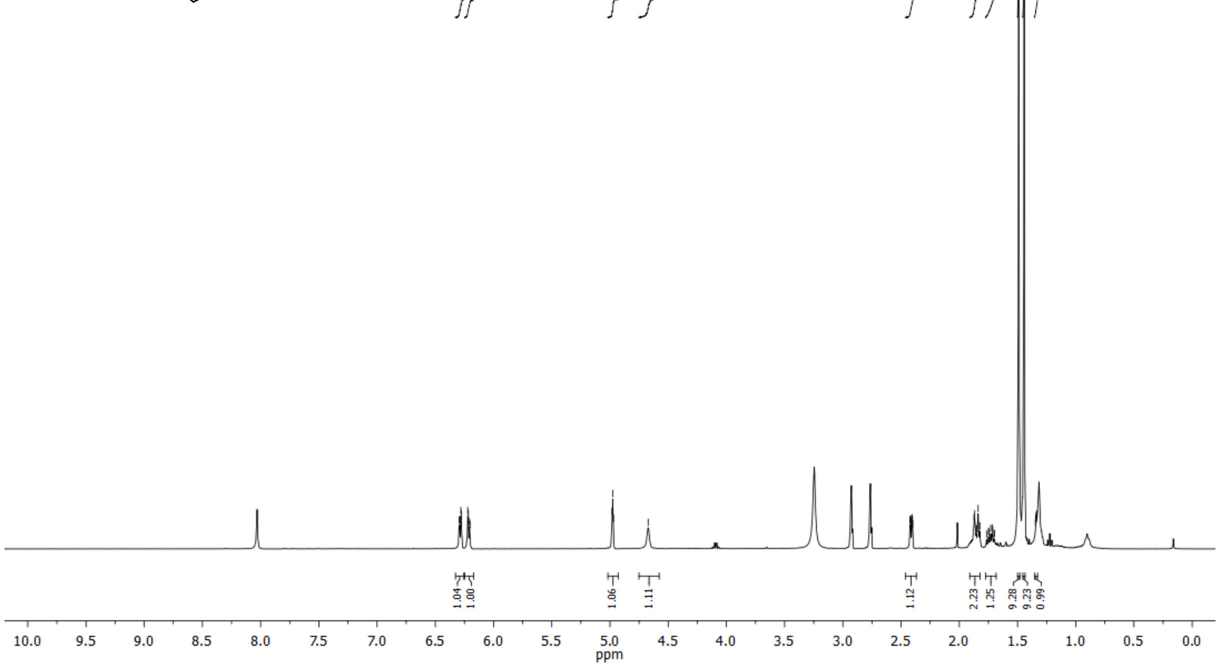

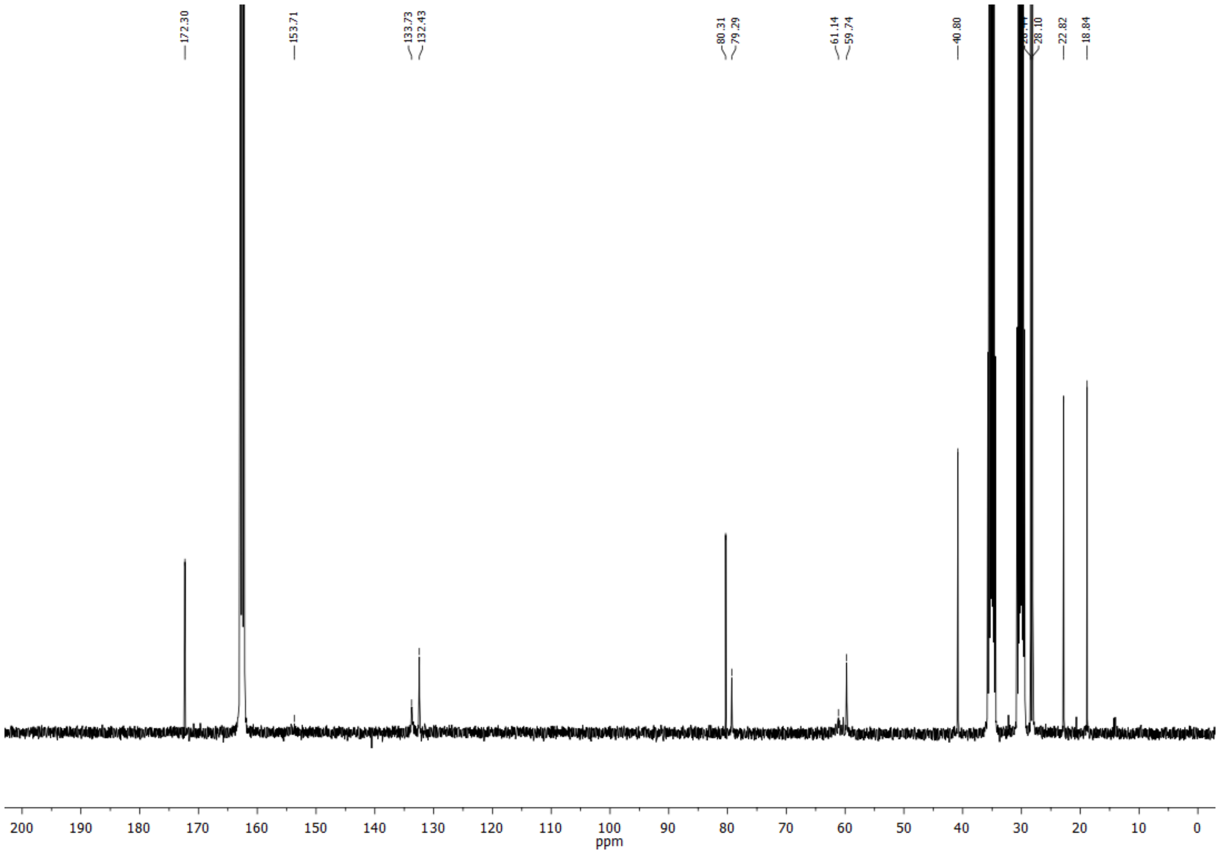

Compound **S2**, ^{1}H NMR and ^{13}C NMR ($CDCl_3$):

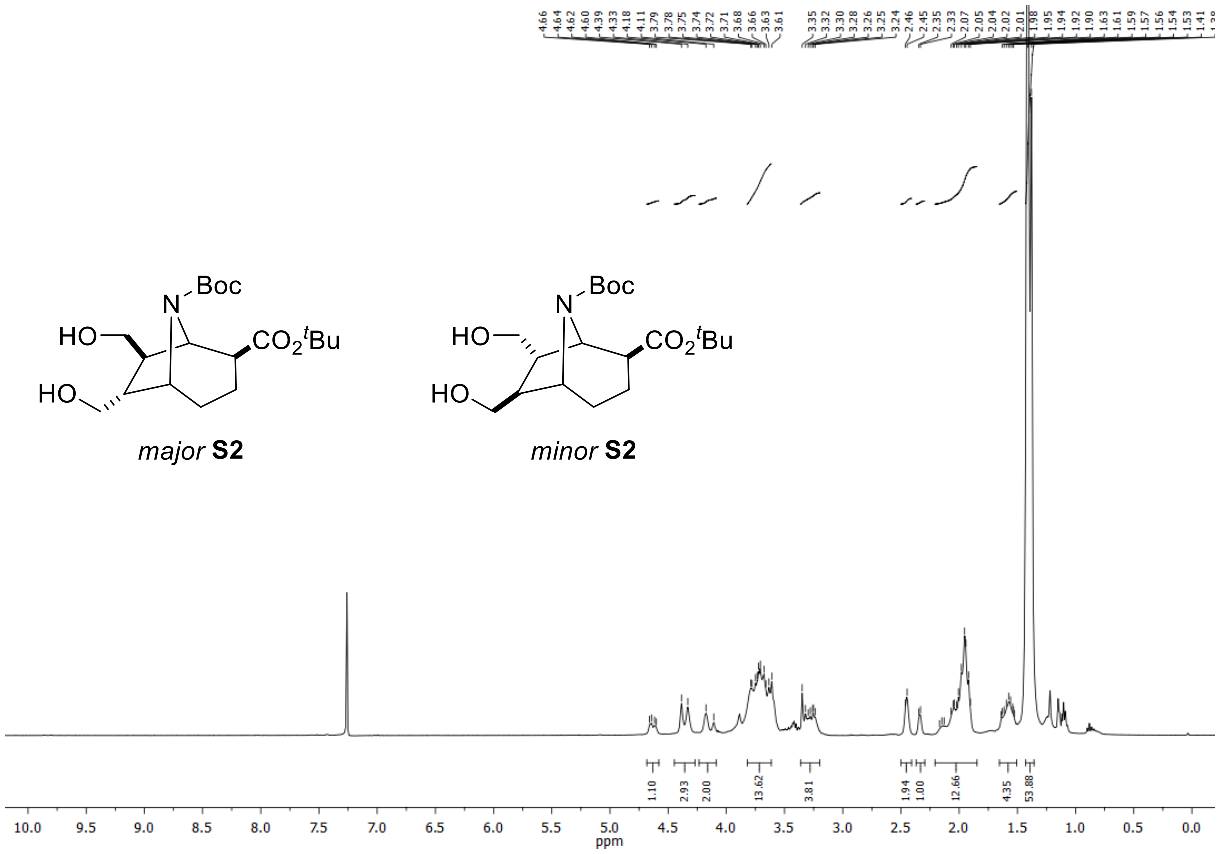

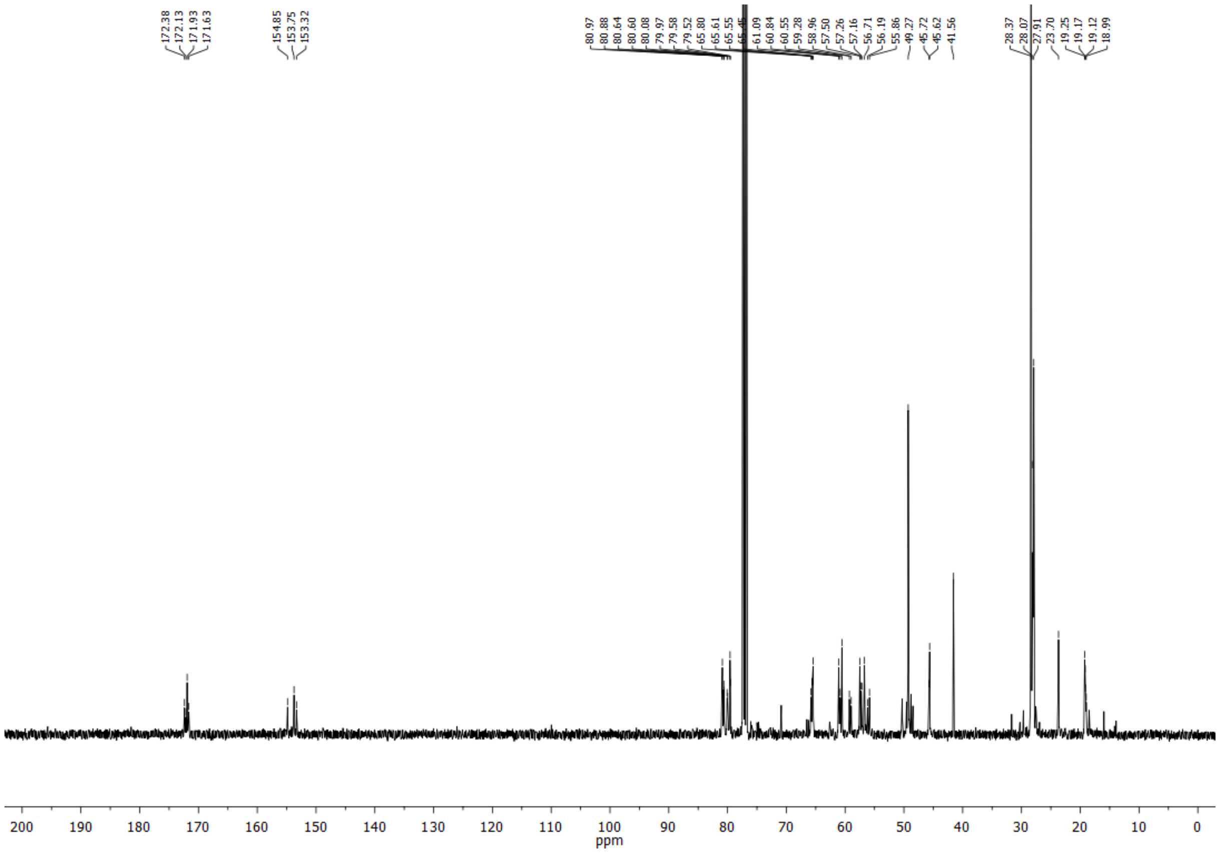

Compound **S3**, ^{1}H NMR and ^{13}C NMR ($CDCl_3$):

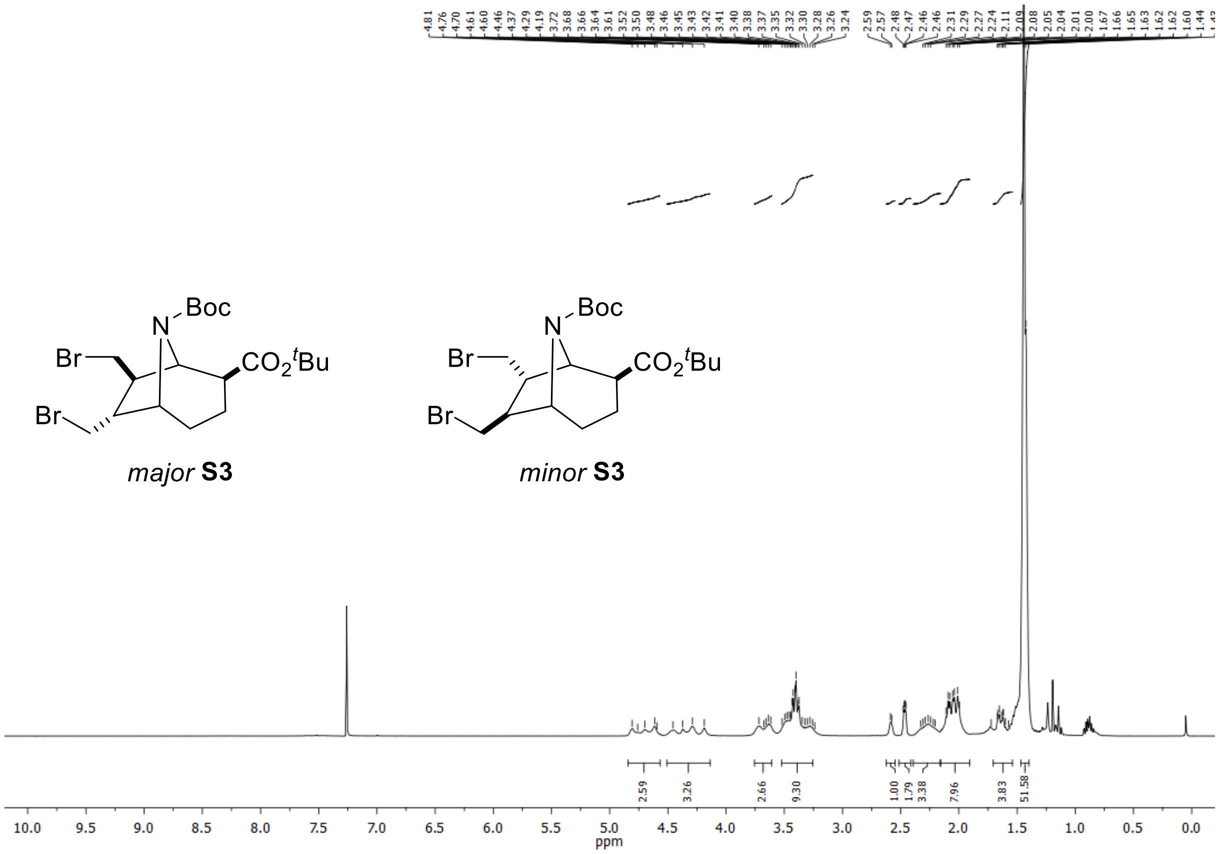

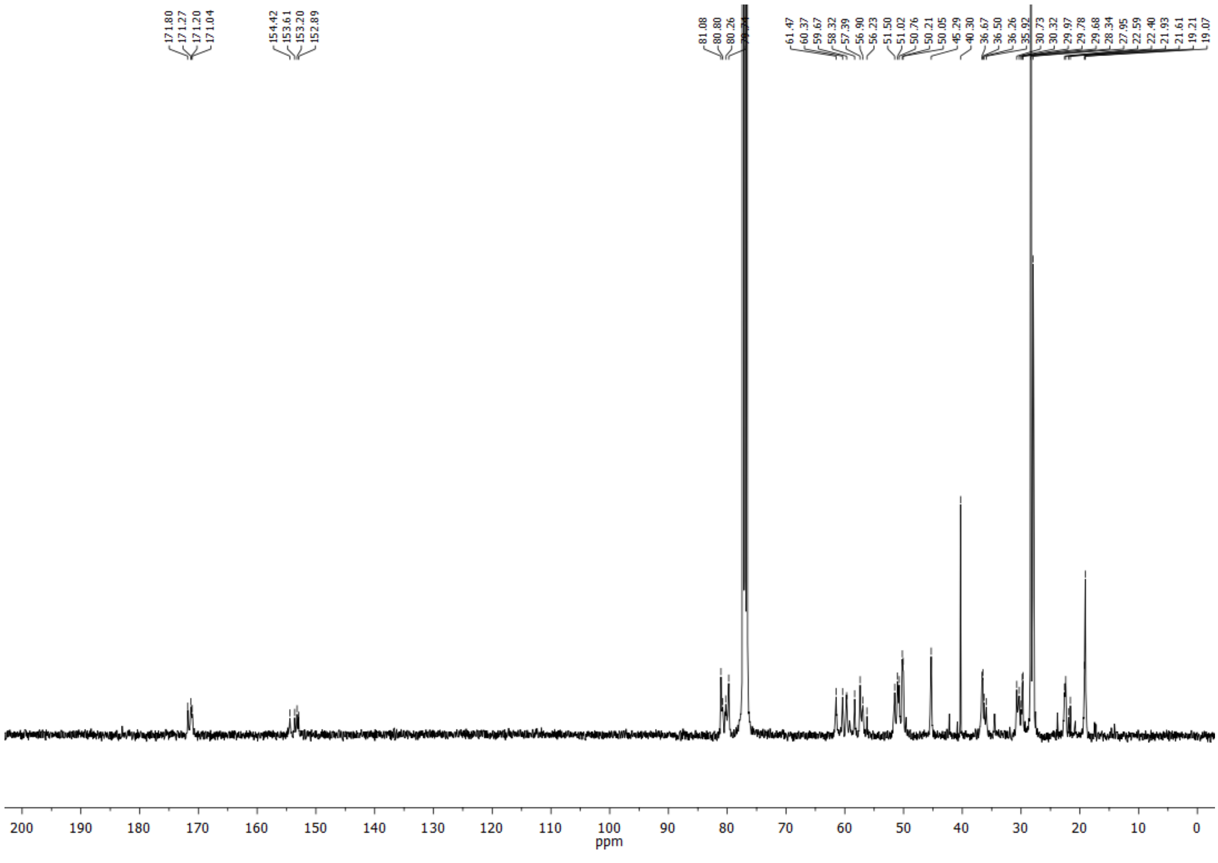

4. X-Ray structures

Ethyl (3a*S*,4*S*,5*S*,8*R*,8a*R*)-1,3-dioxo-9-tosyl-3,3a,4,5,8,8a-hexahydro-1*H*-4,8-epimino-cyclohepta[c]furan-5-carboxylate (7h)

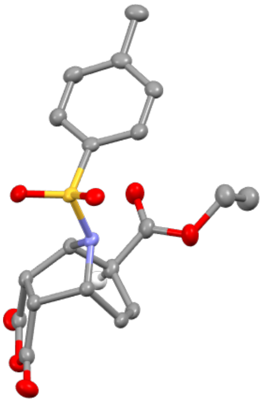

Table 1. Crystal Data and structure refinement for **7h**.

CCDC	1999261
Formula	$C_{19}H_{19}NO_7S$
$D_{calc.}$/ g cm^{-3}	1.458
μ/mm^{-1}	1.947
Formula Weight	405.41
Color	clear colorless
Shape	plate
Size/mm^3	0.24×0.14×0.07
T/K	297.77(10)
Crystal System	triclinic
Space Group	P-1
a/Å	10.9204(3)
b/Å	12.1622(4)
c/Å	14.6939(4)
α/°	94.115(2)
β/°	90.637(2)
γ/°	108.297(3)
V/Å^3	1846.97(10)
Z	4
Z'	2
Wavelength/Å	1.54184
Radiation type	CuK$_\alpha$
Θ_{min}/°	3.840
Θ_{max}/°	73.737
Measured Refl.	48338
Independent Refl.	7407

Reflections Used	6246
R_{int}	0.0885
Parameters	509
Restraints	0
Largest Peak	0.875
Deepest Hole	-0.414
GooF	1.032
wR_2 (all data)	0.1128
wR_2	0.1064
R_1 (all data)	0.0490
R_1	0.0400

4-Ethyl 1,6,7-trimethyl (1*R*,4*S*,5*S*)-8-oxabicyclo[3.2.1]octa-2,6-diene-1,4,6,7-tetracarboxylate ((+)-8a)

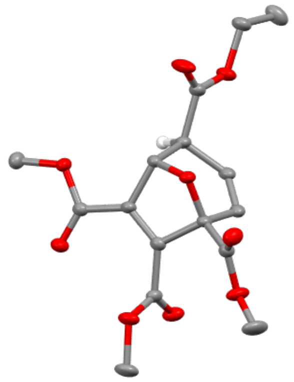

Table 2. Crystal Data and structure refinement for **((+)-8a)**.

CCDC	1999262
Formula	$C_{16}H_{18}O_9$
$D_{calc.}$/ g cm^{-3}	1.417
μ/mm^{-1}	1.008
Formula Weight	354.30
Color	clear colorless
Shape	prism
Size/mm^3	0.32×0.16×0.10
T/K	123.00(10)
Crystal System	monoclinic
Flack Parameter	-0.27(10)
Hooft Parameter	-0.23(8)
Space Group	$P2_1$
a/Å	11.4633(2)
b/Å	10.4467(2)

c/Å	13.8700(2)
α/°	90
β/°	90.5520(10)
γ/°	90
V/Å^3	1660.91(5)
Z	4
Z'	2
Wavelength/Å	1.54184
Radiation type	CuK_α
Θ_{min}/°	3.186
Θ_{max}/°	74.008
Measured Refl.	9844
Independent Refl.	5075
Reflections with I > 2(I)	4841
R_{int}	0.0258
Parameters	584
Restraints	1
Largest Peak	0.235
Deepest Hole	-0.190
GooF	1.039
wR_2 (all data)	0.0836
wR_2	0.0820
R_1 (all data)	0.0342
R_1	0.0319
Creation Method	
Solution	Olex2 1.2-alpha
Refinement	(compiled 2018.07.26 svn.r3523 for OlexSys, GUI svn.r5532)

4-Ethyl 1-methyl (1*R*,4*S*,5*R*,6*R*,7*R*)-6,7-dicyano-8-oxabicyclo[3.2.1]oct-2-ene-1,4-dicarboxylate (*major* (–)-8d)

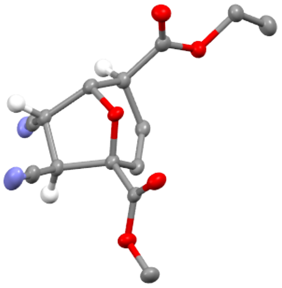

Table 3. Crystal Data and structure refinement for *major* **(–)-8d**.

CCDC	1999263
Formula	$C_{14}H_{14}N_2O_5$
$D_{calc.}$/ g cm^{-3}	1.398
μ/mm^{-1}	0.659
Formula Weight	290.27
Color	clear colorless
Shape	prism
Size/mm^3	0.19×0.13×0.10
T/K	123.00(10)
Crystal System	monoclinic
Flack Parameter	0.03(9)
Hooft Parameter	0.03(8)
Space Group	$P2_1$
a/Å	5.65010(10)
b/Å	10.67929(18)
c/Å	11.4284(2)
α/°	90
β/°	90.9031(16)
γ/°	90
V/Å^3	689.50(2)
Z	2
Z'	1
Wavelength/Å	1.39222
Radiation type	CuK$_\alpha$
Θ_{min}/°	3.493
Θ_{max}/°	74.308
Measured Refl's.	13708
Ind't Refl's	3776
Refl's with I > 2(I)	3653
R_{int}	0.0351
Parameters	192

Restraints	1
Largest Peak	0.311
Deepest Hole	-0.132
GooF	1.061
wR_2 (all data)	0.0813
wR_2	0.0805
R_1 (all data)	0.0314
R_1	0.0302

5-Ethyl 1,7,8-trimethyl (1*S*,2*R*,4*R*,5*R*,6*S*)-3,9-dioxatricyclo[4.2.1.0^{2,4}]non-7-ene-1,5,7,8-tetracarboxylate (10)

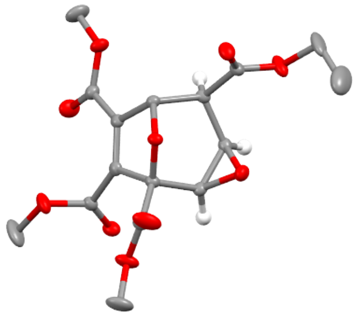

Table 4. Crystal Data and structure refinement for **10**.

CCDC	1999265
Formula	$C_{16}H_{18}O_{10}$
$D_{calc.}$/ g cm^{-3}	1.397
μ/mm^{-1}	1.020
Formula Weight	370.30
Color	clear colorless
Shape	prism
Size/mm^3	0.27×0.12×0.08
T/K	123.00(10)
Crystal System	hexagonal
Flack Parameter	0.01(3)
Hooft Parameter	0.02(3)
Space Group	$P6_5$
a/Å	10.00860(10)
b/Å	10.00860(10)
c/Å	30.4389(3)
α/°	90
β/°	90
γ/°	120
V/Å^3	2640.62(6)

Z	6
Z'	1
Wavelength/Å	1.54184
Radiation type	CuK_α
$\Theta_{min}/^\circ$	5.103
$\Theta_{max}/^\circ$	73.574
Measured Refl.	31431
Independent Refl.	3549
Reflections with I > 2(I)	3501
R_{int}	0.0267
Parameters	239
Restraints	1
Largest Peak	0.343
Deepest Hole	-0.182
GooF	1.039
wR_2 (all data)	0.0684
wR_2	0.0680
R_1 (all data)	0.0264
R_1	0.0260
Creation Method	
Solution	Olex2 1.2-alpha
Refinement	(compiled 2018.07.26 svn.r3523 for OlexSys, GUI svn.r5532)

9-(*Tert*-butyl) 5-ethyl 7,8-dimethyl (1*S*,2*S*,4*R*,5*R*,6*S*)-3-oxa-9-azatricyclo[4.2.1.0^{2,4}]non-7-ene-5,7,8,9-tetracarboxylate (14)

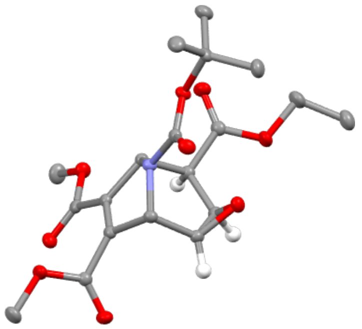

Table 5. Crystal Data and structure refinement for **14**.

CCDC	1999266
Formula	$C_{19}H_{25}NO_9$
$D_{calc.}$/ g cm^{-3}	1.387
μ/mm^{-1}	0.941

Formula Weight	411.40
Color	clear colorless
Shape	prism
Size/mm^3	0.34×0.27×0.20
T/K	123.01(10)
Crystal System	monoclinic
Space Group	$P2_1/c$
a/Å	8.45516(11)
b/Å	24.5268(3)
c/Å	9.65894(13)
α/°	90
β/°	100.4790(13)
γ/°	90
V/Å^3	1969.65(5)
Z	4
Z'	1
Wavelength/Å	1.54184
Radiation type	CuK$_\alpha$
Θ_{min}/°	4.993
Θ_{max}/°	74.260
Measured Refl.	21717
Independent Refl.	3965
Reflections with I > 2(I)	3735
R_{int}	0.0210
Parameters	268
Restraints	0
Largest Peak	0.251
Deepest Hole	-0.294
GooF	1.057
wR_2 (all data)	0.0822
wR_2	0.0805
R_1 (all data)	0.0340
R_1	0.0322
Creation Method	
Solution	Olex2 1.2-alpha
Refinement	(compiled 2018.07.26 svn.r3523 for OlexSys, GUI svn.r5532)

8-(*Tert*-butyl) 2-ethyl 6,7-dimethyl (1*S*,2*R*,3*S*,4*R*,5*S*)-4-bromo-3-hydroxy-8-azabicyclo-[3.2.1]oct-6-ene-2,6,7,8-tetracarboxylate (S1)

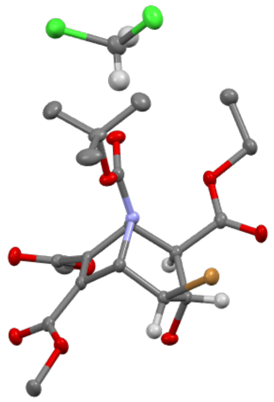

Table 6. Crystal Data and structure refinement for **S1.**

CCDC	1999267
Formula	$C_{20}H_{28}BrCl_2NO_9$
$D_{calc.}$/ g cm^{-3}	1.598
μ/mm^{-1}	4.837
Formula Weight	577.24
Color	dull colorless
Shape	block
Size/mm^3	0.21×0.13×0.10
T/K	123.01(10)
Crystal System	monoclinic
Space Group	$P2_1/c$
a/Å	20.4103(4)
b/Å	9.77236(17)
c/Å	12.09234(16)
α/°	90
β/°	95.9969(14)
γ/°	90
V/Å^3	2398.70(7)
Z	4
Z'	1
Wavelength/Å	1.54184
Radiation type	CuK$_\alpha$
Θ_{min}/°	4.356
Θ_{max}/°	74.175
Measured Refl.	26622
Independent Refl.	4800
Reflections with I > 2(I)	4469
R_{int}	0.0316

Parameters	305
Restraints	0
Largest Peak	0.751
Deepest Hole	-0.570
GooF	1.088
wR_2 (all data)	0.0873
wR_2	0.0857
R_1 (all data)	0.0347
R_1	0.0324
Creation Method	
Solution	Olex2 1.2-alpha
Refinement	(compiled 2018.07.26 svn.r3523 for OlexSys, GUI svn.r5532)

8-(*Tert*-butyl) 2-ethyl 6,7-dimethyl (1*S*,2*S*,5*R*,6*R*,7*S*)-6,7-dihydroxy-8-azabicyclo-[3.2.1]oct-3-ene-2,6,7,8-tetracarboxylate (18)

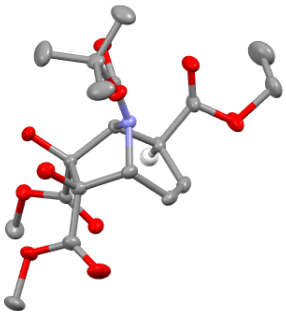

Table 7. Crystal Data and structure refinement for **18**.

CCDC	1999268
Formula	$C_{19}H_{27}NO_{10}$
$D_{calc.}$/ g cm^{-3}	1.358
μ/mm^{-1}	0.940
Formula Weight	429.41
Color	clear colorless
Shape	prism
Size/mm^3	0.25×0.15×0.08
T/K	123.01(10)
Crystal System	monoclinic
Space Group	$P2_1/n$
a/Å	10.16720(10)
b/Å	21.6652(2)
c/Å	19.1977(2)
α/°	90

$\beta/^{\circ}$	96.5370(10)
$\gamma/^{\circ}$	90
V/Å^3	4201.27(7)
Z	8
Z'	2
Wavelength/Å	1.54184
Radiation type	CuK$_{\alpha}$
$\Theta_{min}/^{\circ}$	4.081
$\Theta_{max}/^{\circ}$	74.212
Measured Refl.	43126
Independent Refl.	8419
Reflections with I > 2(I)	7647
R_{int}	0.0295
Parameters	777
Restraints	42
Largest Peak	0.501
Deepest Hole	-0.329
GooF	1.019
wR_2 (all data)	0.1112
wR_2	0.1073
R_1 (all data)	0.0437
R_1	0.0400

Trimethyl (1*S*,4*R*,7*R*,8*R*)-8-(benzoyloxy)-2-methyl-2-azabicyclo[2.2.2]oct-5-ene-5,6,7-tricarboxylate (22)

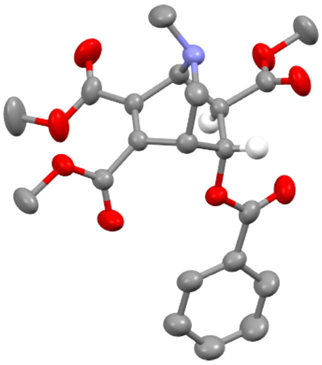

Table 8. Crystal Data and structure refinement for **22**.

CCDC	1999270
Formula	$C_{21}H_{23}NO_8$
$D_{calc.}$/ g cm^{-3}	1.338
μ/mm^{-1}	0.870
Formula Weight	417.40

Color	clear colorless
Shape	prism
Size/mm^3	0.20×0.11×0.06
T/K	293(2)
Crystal System	monoclinic
Space Group	$P2_1/c$
a/Å	19.5386(3)
b/Å	9.90775(17)
c/Å	10.74109(19)
α/°	90
β/°	94.6462(16)
γ/°	90
V/Å^3	2072.46(6)
Z	4
Z'	1
Wavelength/Å	1.54184
Radiation type	CuK$_\alpha$
Θ_{min}/°	4.541
Θ_{max}/°	72.581
Measured Refl's.	22284
Ind't Refl's	4069
Refl's with I > 2(I)	3538
R_{int}	0.0208
Parameters	287
Restraints	151
Largest Peak	0.405
Deepest Hole	-0.241
GooF	1.028
wR_2 (all data)	0.1340
wR_2	0.1269
R_1 (all data)	0.0521
R_1	0.0461

5. HPLC chromatograms

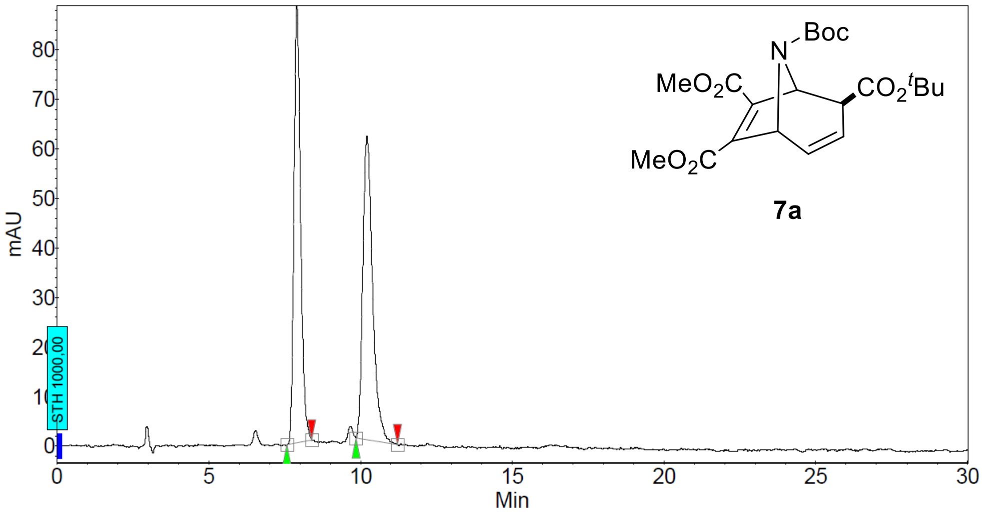

Peak Results:

Index	Time (min)	Area (mAU min)	Area (%)
1	7.90	22.5	49.565
2	10.21	22.9	50.435
Total		45.5	100.00

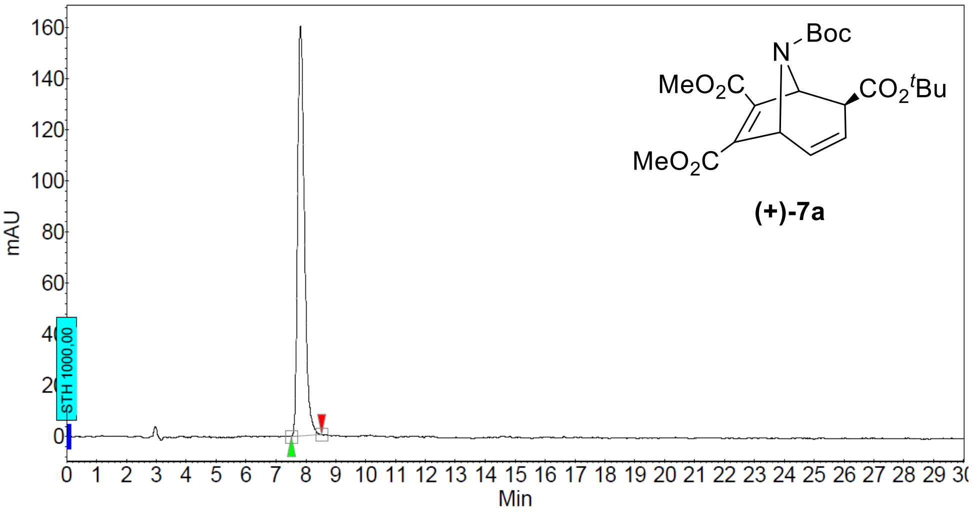

Peak Results:

Index	Time (min)	Area (mAU min)	Area (%)
1	7.82	42.2	100

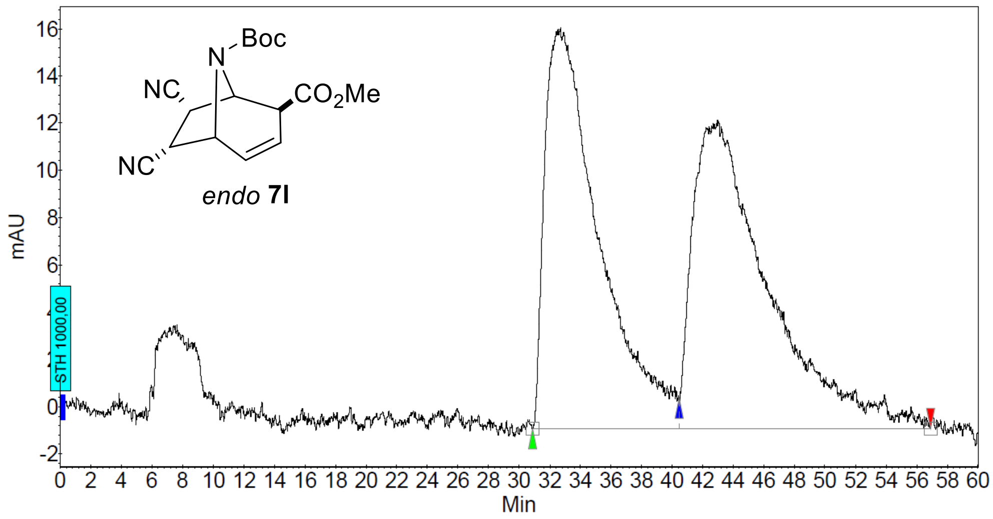

Peak Results:

Index	Time (min)	Area (mAU min)	Area (%)
1	32.72	69.8	48.302
2	42.98	74.7	51.698
Total		144.4	100.00

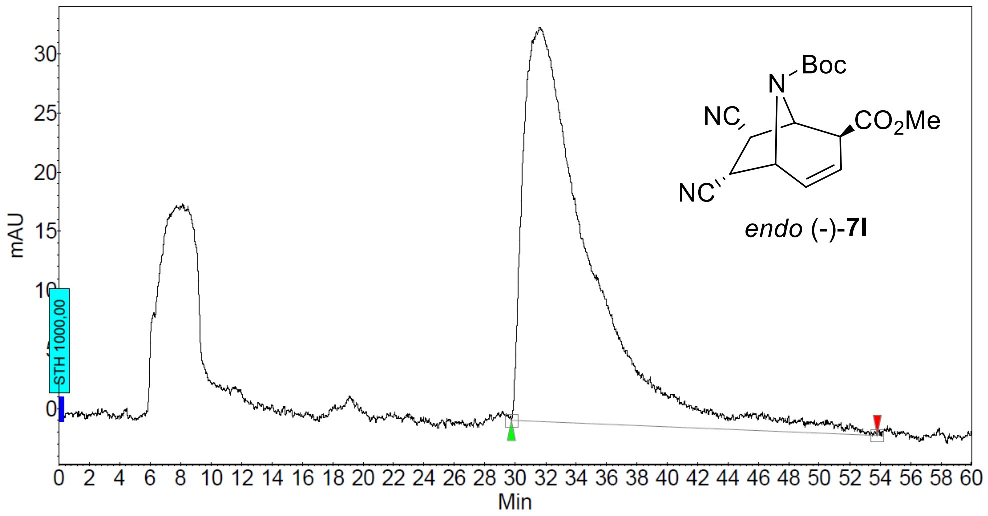

Peak Results:

Index	Time (min)	Area (mAU min)	Area (%)
1	31.61	167.2	100.000

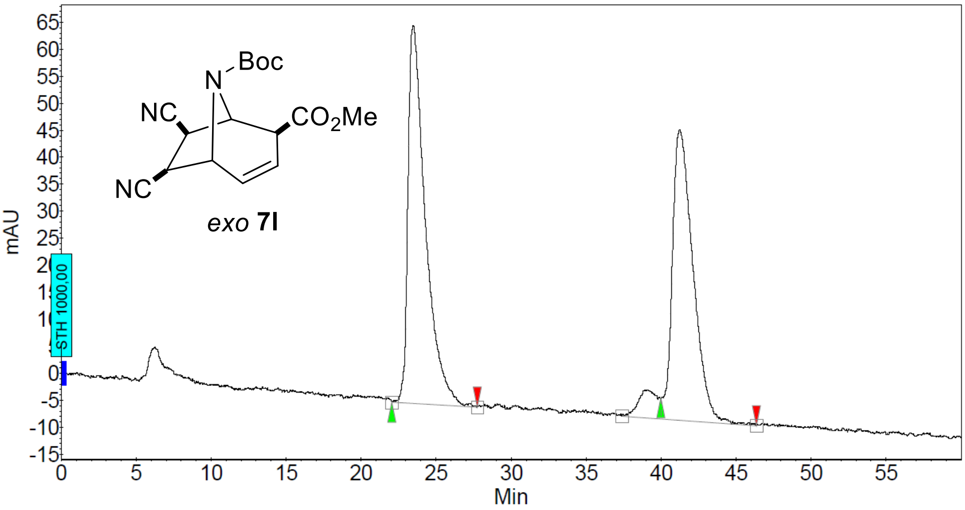

Peak Results:

Index	Time (min)	Area (mAU min)	Area (%)
1	23.45	94.0	52.213
2	41.24	86.1	47.787
Total		180.1	100.00

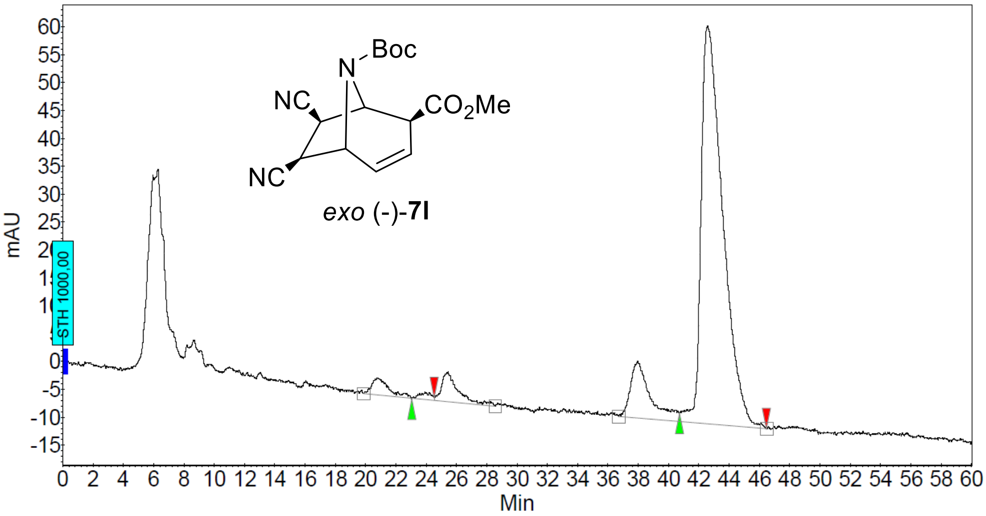

Peak Results:

Index	Time (min)	Area (mAU min)	Area (%)
1	24.21	1.1	0.943
2	42.56	119.0	99.057
Total		120.1	100.00

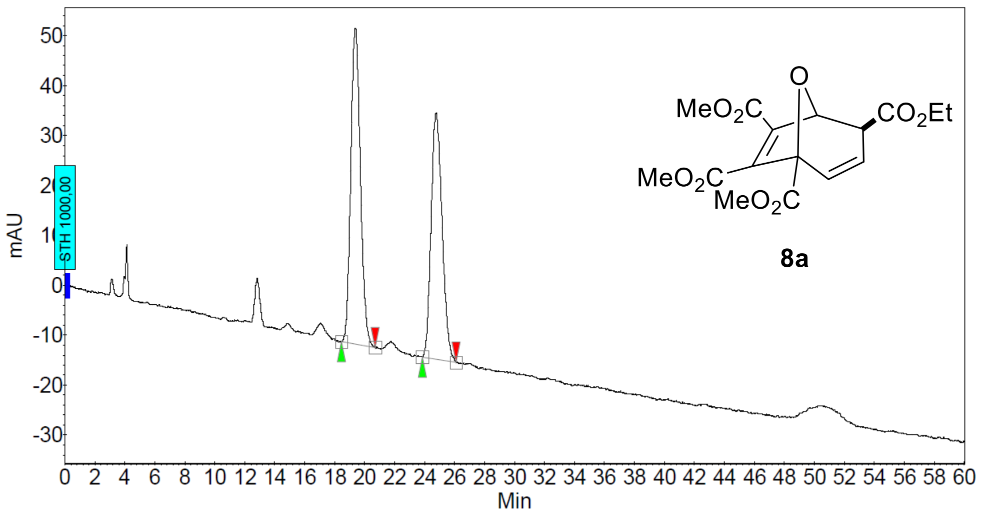

Peak Results:

Index	Time (min)	Area (mAU min)	Area (%)
1	19.38	47.2	52.911
2	24.77	42.0	47.089
Total		89.1	100.00

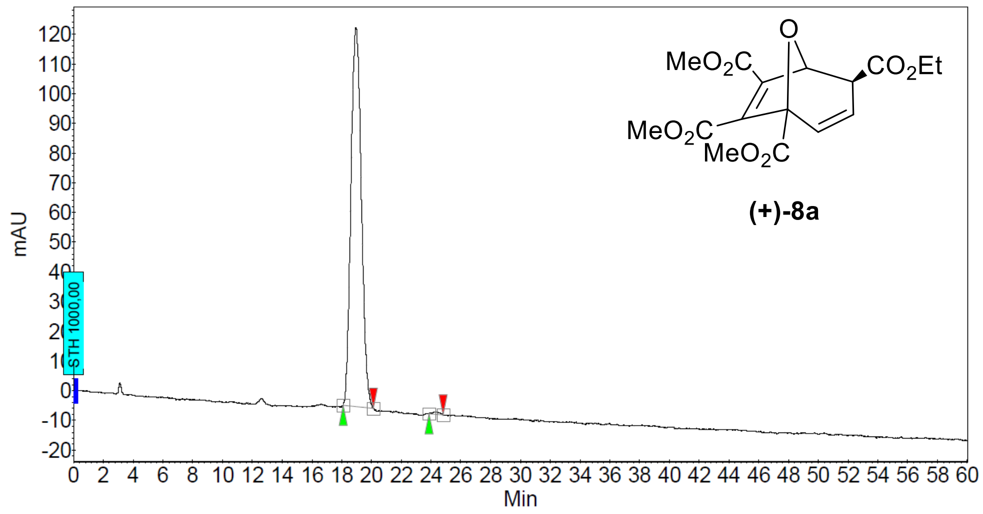

Peak Results:

Index	Time (min)	Area (mAU min)	Area (%)
1	18.96	103.2	99.544
2	24.37	0.5	0.456
Total		103.7	100.00

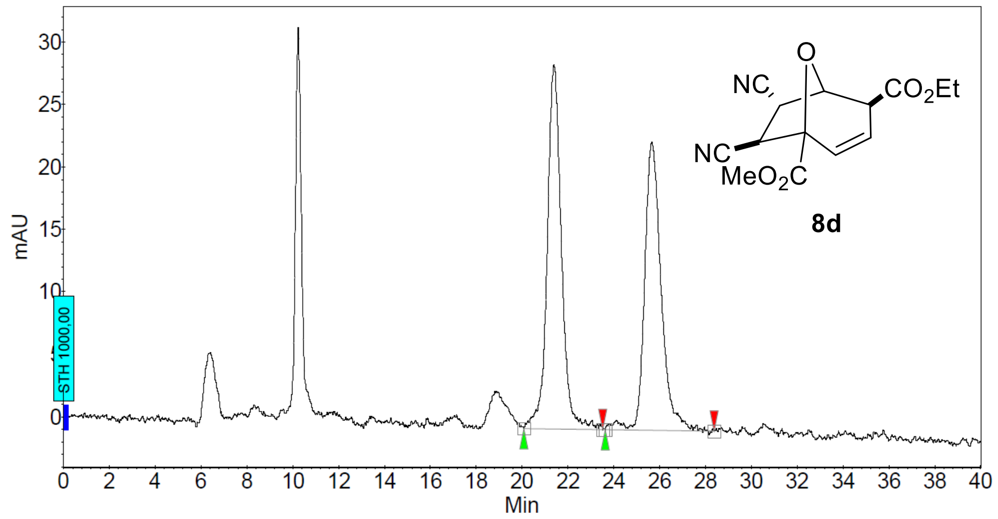

Peak Results:

Index	Time (min)	Area (mAU min)	Area (%)
1	21.39	21.0	51.997
2	25.66	19.4	48.003
Total		40.4	100.00

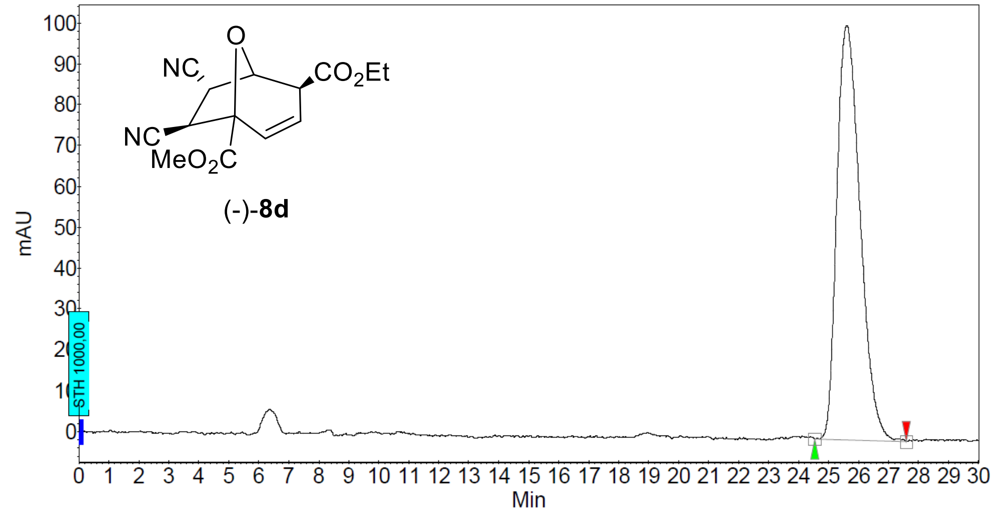

Peak Results:

Index	Time (min)	Area (mAU min)	Area (%)
1	25.61	91.4	100.000

6. References

[1] R. Manfred, H. Jürgen, L. Annemarie, *Org.Synth.* **1968**, *48*, 36.

[2] H. N. C. Wong, X. U. E. Long Hou, *Synthesis* **1985**, 1111.

[3] D. A. Evans, K. A. Woerpel, B. Nosse, A. Schall, Y. Shinde, E. Jezek, E. Mahbubul, M. Haque, R. B. Chhor, O. Reiser, *Org.Synth.* **2006**, *83*, 97.

[4] N. E. Searle, *Org.Synth.* **1956**, *36*.

[5] C. W. Williams, R. Shenje, S. France, *J. Org. Chem.* **2016**, *81*, 8253.

[6] L. Grehn, U. Ragnarsson, *Angew. Chem. Int. Ed.* **1984**, *23*, 296.

[7] L. Waykole, A. L. Paquette, *Org.Synth.* **1989**, *67*.

[8] L. K. A. Pilsl, T. Ertl, O. Reiser, *Org. Lett.* **2017**, *19*, 2754.

[9] R. Beumer, University Regensburg, **2000**.

[10] R. B. Chhor, B. Nosse, S. Sörgel, C. Böhm, M. Seitz, O. Reiser, *Chem. Eur. J.* **2003**, *9*, 260.

[11] T. K. M. Shing, V. W.-F. Tai, E. K. W. Tam, *Angew. Chem. Int. Ed.* **1994**, *33*, 2312.
